# A retrotransposon-derived DNA zip code internalizes myeloma cells through Clathrin-Rab5a-mediated endocytosis

**DOI:** 10.3389/fonc.2024.1288724

**Published:** 2024-02-23

**Authors:** Pavan Kumar Puvvula, Lourdes Martinez-Medina, Munevver Cinar, Lei Feng, Andrey Pisarev, Anthony Johnson, Leon Bernal-Mizrachi

**Affiliations:** ^1^ Kodikaz Therapeutic Solutions, New York, NY, United States; ^2^ Department of Hematology and Medical Oncology, Winship Cancer Institute of Emory University, Atlanta, GA, United States

**Keywords:** myeloma, endocytosis, gene therapy, cancer biology, non-viral vectors, cell-specific targeted delivery

## Abstract

**Introduction:**

We have demonstrated that transposons derived from ctDNA can be transferred between cancer cells. The present research aimed to investigate the cellular uptake and intracellular trafficking of Multiple Myeloma-zip code (MM-ZC), a cell-specific zip code, in myeloma cell lines. We demonstrated that MM-ZC uptake by myeloma cells was concentration-, time- and cell-type-dependent.

**Methods:**

Flow cytometry and confocal microscopy methods were used to identify the level of internalization of the zip codes in MM cells. To screen for the mechanism of internalization, we used multiple inhibitors of endocytosis. These experiments were followed by biotin pulldown and confocal microscopy for validation. Single interference RNA (siRNA) targeting some of the proteins involved in endocytosis was used to validate the role of this pathway in ZC cell internalization.

**Results:**

Endocytosis inhibitors identified that Monensin and Chlorpromazine hydrochloride significantly reduced MM-ZC internalization. These findings suggested that Clathrin-mediated endocytosis and endosomal maturation play a crucial role in the cellular uptake of MM-ZC. Biotin pulldown and confocal microscopic studies revealed the involvement of proteins such as Clathrin, Rab5a, Syntaxin-6, and RCAS1 in facilitating the internalization of MM-ZC. Knockdown of Rab5a and Clathrin proteins reduced cellular uptake of MM-ZC and conclusively demonstrated the involvement of Clathrin-Rab5a pathways in MM-ZC endocytosis. Furthermore, both Rab5a and Clathrin reciprocally affected their association with MM-ZC when we depleted their proteins by siRNAs. Additionally, the loss of Rab5a decreased the Syntaxin-6 association with MMZC but not vice versa. Conversely, MM-ZC treatment enhanced the association between Clathrin and Rab5a.

**Conclusion:**

Overall, the current study provides valuable insights into the cellular uptake and intracellular trafficking of MM-ZC in myeloma cells. Identifying these mechanisms and molecular players involved in MM-ZC uptake contributes to a better understanding of the delivery and potential applications of cell-specific Zip-Codes in gene delivery and drug targeting in cancer research.

## Background and significance

Horizontal gene transfer (HGT) is the mechanism by which genes transfer between cells and impact genome evolution and regulation (1). Transposons play a significant role in HGT in eukaryotes, such as insects and plants (2). Earlier studies suggested that HGT can occur and alter tumor phenotype (3, 4) by circulating tumor DNA (ctDNA) (5). However, the mechanistic details of ctDNA-mediated HGT need to be better understood. Kodikaz’s pioneering research has proved the existence of the HGT mechanism in human cancers. We have identified a unique characteristic of ctDNA that has not been reported previously – its tropism for cells similar to those from which it originated. Identifying the retrotransposons at ctDNA insertion sites, reducing ctDNA insertions by transposition inhibitors, and delivery of genes by the synthetically generated retrotransposons support the notion that the retrotransposons DNA sequences primarily mediate HGT in cancer (1) Kodikaz has leveraged this knowledge of HGT to synthesize an array of transposons sequences (cell-specific Zip-Codes, ZCs) against multiple myeloma cells, hereafter referred to as MM-ZC. These ZCs serve as a molecular vehicle for delivering different payloads without eliciting an immune or DNA damage or stress response. Examination of the ZC internalization by deletion and mutation studies highlighted the significance of AT-rich regions in the cellular uptake of the ZCs. However, nothing is known about the mechanism or molecular players that facilitate the internalization of ZCs in cancer cells.

Preliminary studies demonstrated that cancer cells can engulf exogenous DNA from their environment through endocytosis (6). The DNA is enclosed in vesicles called endosomes (7), which are formed by the invagination of the cell membrane. Once inside the cancer cell, the endosomes can fuse with lysosomes, where the DNA is degraded, or they can release it into the cytoplasm (8). Some cancer cells express specific receptors, such as scavenger and toll-like receptors on their surface, that can bind to DNA molecules and facilitate the uptake of DNA into the cell through receptor-mediated endocytosis (9). In addition, cancer cells can also use macropinocytosis, a form of nonspecific bulk endocytosis, to take up extracellular DNA (10). It is believed that DNA uptake may contribute to the genetic heterogeneity and adaptability of cancer cells, potentially influencing tumor progression, resistance to therapy, and the acquisition of new genetic traits.

Endocytosis is a cellular process by which cells internalize molecules or particles from their external environment. When viral particles come into contact with the surface of a cell, they can exploit macropinocytosis, Clathrin, and Caveolae-mediated endocytosis as a mechanism to gain entry into the cells (11). However, the exact mechanisms vary depending on the virus and its specific lifecycle. Similarly, cells engulfed and internalized nanoparticles through phagocytosis, macropinocytosis, and Clathrin-mediated endocytosis (12). The internalization efficiency of nanoparticles can vary depending on their size, shape, surface charge, and surface modifications. Different cell types may also have distinct preferences for specific endocytic pathways for any given molecule (13, 14). While endocytosis is primarily associated with the uptake of various molecules and particles, such as nutrients, signaling molecules, and viruses, it can also involve the internalization of non-viral DNAs in liposome-mediated transfections. Irrespective of the type, the process of endocytosis is highly complex and dynamic, and various other proteins and regulatory factors such as Clathrin, adaptor proteins, dynamin, Epsin, Rab family proteins, endophilin, and caveolins contribute to its regulation and efficiency (15). However, the cellular machinery responsible for the internalization of DNA in cancer cells by HGT is poorly understood. Multiple myeloma is a type of cancer affecting plasma cells, a specialized type of white blood cells. Plasma cells produce antibodies that help the body fight off infections. The dysregulated growth and proliferation of plasma cells in myeloma cells result in alterations in their cellular processes and biological functions. Although studies have examined general aspects of endocytic processes, specific research on endocytosis of MM-ZCs in myeloma cells is unknown.

Our previous studies indicated that tissue-specific retrotransposons facilitate the process wherein circulating tumor DNA (ctDNA) targets and transmits genetic material to cells resembling their cell of origin. Biochemical studies uncovered that naked ctDNA can function as a vehicle for transferring genes between cancer cells. The enrichment of ctDNA insertion sites with retrotransposons and the reduction of ctDNA insertions by retro transposition inhibitors support the idea that retrotransposons play an essential role in mediating Horizontal Gene Transfer (HGT) in cancer. One such retrotransposons in myeloma-specific ctDNA insertion site belongs to AluSp. We identified its sequence by genome sequencing, chemically synthesized its 300bp DNAs, and tested its properties by employing various cellular models. This DNA sequence was later termed as MM-ZC and its functional characterization revealed that it harbors a cell recognition signal from the middle region to the C-terminus side. The successful ligation of MM-ZC to mCherry mediated the internalization and integration of mCherry into MM1S cells. This observation establishes MM-ZC as a specific vehicle for delivering various cargos into MM1s cells. Considering the implications in clinical studies, Kodikaz Therapeutics explored using MM-ZC as an efficient drug delivery vehicle. Different payloads, including small molecule drugs, peptides, RNAi, and gene cassettes, are being tagged for delivery as a part of the Kodikaz scientific explorations for developing novel, unique hybrid therapeutic anti-cancer molecules.

Despite the unique interest in using these ZCs as delivery vehicles for the payloads in biomedical and clinical research, a clear understanding of the mechanistic details of cellular internalization still needs to be discovered. Hence, the present investigation is to evaluate the cellular uptake and mechanistic understanding of intracellular trafficking of MM-ZC in myeloma cell lines. In addition, we aim to identify the endocytosis mechanism and determine the cellular factors that mediate the internalization of MM-ZC in myeloma cells. Initially, we characterized the cell type dependency, dose, and time requirement of MM-ZC internalization in myeloma cells, and the results clearly showed that the uptake of the MM-ZC correlated positively to both time and concentration. Subsequently, using various endocytosis inhibitors, we identified that monensin and MβCD, the inhibitors of endosomal maturation and Clathrin-mediated endocytosis, significantly reduced the MM-ZC internalization in MM1s and JK6L cells. Streptavidin pulldown assays using MM1S and JK6L cell lysates treated with Biotin labeled MM-ZC showed that Clathrin, Rab5a, Syntaxin-6, and RCAS1 proteins are associated with MM-ZC. Colocalization further confirmed the pulldown results and clearly demonstrated that MM-ZCs internalize through the Clathrin-mediated endocytosis process with the help of Rab5a, Syntaxin-6, and RCAS1. To ensure the observed results are specific to MM-ZC, we amplified five different DNA sequences with the same length of base pairs and tested them on myeloma cell lines. We evaluated their internalization by immunofluorescence and association with the known endosomal marker protein, Rab5a. Our results demonstrated that the cellular uptake of the MM-ZC sequence is significantly higher than the rest of the sequences. The endosomal protein Rab5a is specifically associated with MM-ZC but not with other sequences. The uptake behaviour of the MM-ZC was further evaluated in Rab5a, Clathrin, Syntaxin-6, and caveolin knockdown MM1s and JK6L cells. These results demonstrated that MM-ZC internalization significantly reduced in Rab5a and Clathrin but not in caveolin knockdown cells. Syntaxin-6 knockdown showed a modest effect on MM-ZC uptake. These discoveries reveal that MM-ZC utilizes the Clathrin-mediated Rab5a endocytosis pathway to enter into cells, indicating that this process is a key aspect of ZC-mediated gene delivery.

## Materials and methods

### Cell culture

MM1S, U266B1, and MM1R cell lines were obtained and maintained as per the procedures mentioned in ATCC. JK6L, OPM1, and OPM2 cell lines are available in Bermal lab. All culture media were supplemented with 10% fetal bovine plasma, 1% L-glutamine, 1mM sodium pyruvate, and 50 μg/ml penicillin-streptomycin.

### Antibodies

Na, K-ATPase α1 (Cell Signaling, 23565), ENPP1 (Cell Signaling, 5342), Caveolin (Cell Signaling, 3267), 4F2hc/CD98 (Cell Signaling, 47213), Pan-Cadherin (Cell Signaling, 4073), Clathrin heavy chain (Cell Signaling, 4796), EEA1 (Cell Signaling, 3288), Rab5a (Cell Signaling, 46449), Rab7 (Cell Signaling, 9367), Rab11 (Cell Signaling, 5589), Calnexin (Cell Signaling, 2679), ERp72 (Cell Signaling, 5033), PDI (Cell Signaling, 3501), RCAS1 (Cell Signaling, 12290), Syntaxin-6 (Cell Signaling, 2869), Anti-rabbit IgG-HRP (Cell Signaling, 7074), Anti-mouse IgG-HRP (Cell Signaling, 7076).

Dignam lysates were prepared as per the procedure mentioned earlier (Dignam et al.,1983) with minor modifications. Cell pellets were collected, washed twice in ice-cold phosphate-buffered saline (PBS) and resuspended at one million cells per 0.5 mL of buffer C (0.42 M NaCl, 10% glycerol, 20 mM HEPES [pH 7.9], 1.5 mM MgCl2, 0.2 mM EDTA, 0.5 mM dithiothreitol [DTT], 0.5 mM phenylmethylsulfonyl fluoride [PMSF]) (14), followed by centrifugation for 15 min at 10,000 g.

### Immunoblotting

Briefly, whole-cell lysates or immunoprecipitated samples were resolved by 4–20% precast BioRad gels (BioRad). PAGE separated proteins were transferred onto a PVDF membrane at 15V overnight at 4C in a Tris-Glycine Transfer Buffer (25 mM Tris, 192 mM glycine, 10% Methanol) by wet transfer procedure in Mini Trans-Blot cell as per the manufacturer protocol. Protein-bound PVDF membranes were incubated for 2 hr at RT with 5% skimmed milk in TBST buffer (20 mM Tris, 150 mM NaCl, 0.1% tween20). Blocked membranes were incubated overnight with primary antibodies. Later, three washes of 5 minutes each in TBST were performed followed by 2 hr incubation at RT with the 1:5000 or 1:10000 diluted horseradish peroxidase conjugated secondary antibodies. Protein bands were visualized with ECL western blotting reagents (GE Health care, Cat no # RPN2106).

### Co-immunoprecipitation

It was performed as previously described (16). Briefly, lysates were prepared using the Dignam buffer. Cleared lysates were incubated for 4 hr in the cold chamber with specific primary antibodies followed by an additional 2 hr of incubation with the pre-equilibrated Dynabeads Protein G (Invitrogen) at 4C. Immunoprecipitated beads were washed five times with lysis buffer and resuspended in 4X SDS-loading buffer. Immunoprecipitated proteins were subjected to SDS-PAGE analysis followed by immunoblotting with specific antibodies. Co-immunoprecipitations and immunoblotting results were confirmed in 3 replicates.

Note: in figures, immunoprecipitation antibodies are listed at top, and immunoblot antibodies are listed at the left or bottom of the panels. Input lanes contain 5% of protein lysate used for IP; 25% of IP’d material was loaded and subjected to immunoblotting.

Immunofluorescence was performed as described previously (17–19). Cells were fixed with 1% PFA for 10 mins and subsequently permeabilized with 0.5% triton-x100 in 1xPBS. Later, cells were washed with PBS and incubated with 5% blocking buffer for 4 hr before primary antibody incubation. Cells were stained with antibodies against Rab5a, Clathrin, Syntaxin-6, RCAS1, CD98 and phalloidin in the cold room for 4 hr. Cells were washed with PBS and subjected to incubation with conjugated secondary antibodies. The secondary antibodies used to detect the staining of proteins were Alexa Fluor 488, 594, and 647, respectively. DNA counterstaining was performed using DAPI (4′,6-diamidino-2-phenylindole). The cell droplets were placed onto coverslips and air-dried to evaporate all the liquid. Semi-dried cells on the coverslips were mounted on the glass slides. Cells were visualized under Zeiss confocal microscope Z007, and digital images were acquired using a × 63 numerical apertures (NA)=1.46 oil objective for high magnification images and a × 20 NA=0.16 objective for low magnification images. High-resolution images were acquired as a z-stack with an ~0.2 μm z-interval. 3D projections were further processed offline using Image J software (20).

3D images from quadruplicate experiments were acquired using a Leica SP8 LIGHTING confocal microscope housed in the Cell Imaging and Microscopy Shared Resource, Winship Cancer Institute of Emory University. The 3D visualization and colocalization were performed using surfaces created in Bitplane Imaris 10. Colocalization of DNA and proteins was measured using overlapped volume to surface measurement, and colocalization of two proteins was measured by Pearson correlation in the area of interest.

### Biotin pulldown assay

One million cells are treated with 500ng of biotin labeled DNA sequences and incubated for 4 hr in a standard cell culture incubator. Post-treatment, cells were crosslinked with 1% formaldehyde for 5 minutes, followed by quenching with 125mM glycine. The cell pellet was washed with cold PBS and resuspended in 500 μL lysis buffer (50 mM HEPES-KOH pH 7.5, 140 mM NaCl, 1 mM EDTA, 1% Triton X-100, 0.1% sodium deoxycholate and protease inhibitors). 1 ml of cleared lysate with 5mg of total protein was incubated overnight with 100 μl of streptavidin magnetic beads in the cold chamber. Immunoprecipitated complexes were collected by magnetic bar and, washed three times with lysis buffer and resuspended in 4X SDS-loading buffer. Biotin labeled MM-ZC1 associated proteins were subjected to SDS-PAGE analysis followed by immunoblotting with specific antibodies. Input lanes contain 5% of protein lysate used for immunoprecipitation (IP); 25% of IP’d material was loaded and subjected to immunoblotting.

### Cell viability analysis

Myeloma cell lines were seeded at a density of 5,000 cells/well in a 96-well plate and cultured in RPMI medium at 37°C. After 24 hr, endocytosis inhibitors, were added to the cells and incubated for a specified time, as mentioned in the figure legends, at 37°C. Cell viability was measured by CellTiter-Blue Cell viability Assay (Cat.No: G8080) and CellTiter-Glo 2.0 Cell Viability Assay (Cat.No: G9241 (Promega Corporation, Madison, WI, USA) as per the procedure mentioned in the manufacture report. Each experiment was performed in triplicates. Fluorescent labeling of DNAs: Various DNA sequences as mentioned in the figure legends, were specifically amplified by Taq polymerase in PCR amplification. 5μg of purified DNA was utilized for labeling with either Cx-Rhodamine or Biotin using the Label IT^®^ Nucleic Acid Labeling kit (Mirus Bio LLC, WI).

### siRNA preparation

The siRNA pellets were resuspended in nuclease-free water to make a stock of 100 µM. A working solution of 50μM siRNA was made fresh before electroporating the cells. lucsiRNAs were used as negative controls.

SiRNA Knockdown: Myeloma cell lines are transfected with 100nM concentration of control (SC-37007) or Rab5a (SC-36344) or Syntaxin-6 (SC-41333) or Clathrin (SC-35067) or caveolin (SC-29241) specific siRNAs using Neon transfection system as per manufacturer’s instructions.

### Transfections

The electroporation of all myeloma cell lines was carried out using the Neon Transfection System from Invitrogen, following the manufacturer’s instructions. The Neon Transfection System, 100 µL kit, was employed for transfecting myeloma cell lines. Cells were subcultured 48 hr before electroporation and harvested when they reached nearly 80% confluency. The cells, at a density of 2 × 10^5^, were washed with PBS and resuspended in 100 µl Resuspension buffer R. Within 10 minutes of resuspension, the cells were mixed with 100nM siRNA duplexes in a separate tube and immediately proceeded to the Neon Transfection System. Electroporation was performed by applying two pulses at 1400 Volts for 20 milli seconds to all cell lines. Following electroporation, the cells were seeded in a 6-well plate containing 2.5 mL media of RPMI (ATCC) with 10% FBS. Transfected cells were cultured for 48–72 hr and subsequently proceeded for further analysis. Western Blot was implemented to verify the downregulation of proteins.

PCR reactions are performed with *taq* DNA polymerase as per the manufacturer’s suggestion. Primer details are mentioned below.

Ctr: FP: GTGAACCGCATCGAGCTGAAGGGC;
RP: TCGCGCTTCTCGTTGGGGTCTTTGC
MM-ZC: FP: ACCCGGCCTTGGACACGCCA;
RP: GGTGGGCAGATCATGAGGT
MM1: FP: GGTCAGTCGCGGTGGCTC;
RP: GAGACGGAGTCTTGCTCTGTCACC
MM3: FP: TGATATGGCTTGGCTGTGTCCC;
RP: TGTATTAGTCCATTTTCATGCTGCTG
MM4: FP: GAGACAGAGTCTGCTCTCGTTGC;
RP: GGCTGAGTGCGGTGGCT
PC1: FP: AGCCAGGTGCAGTGGCTC;
RP: GAGACGGATTCTCGCTCTCTTGC
Vector: FP: GTGCCACCTGACGTCTAAGAAACC;
RP: GAGCTCACCCCAATTCACTGGCCGTC
PC3: FP: TGTGTTAGTCAGGATAGGCTAACCTCTG;
RP: TGTGTTAGTCAGGATAGGCTAACCTCTG

### Inhibitor studies

Cells were seeded at a density of 1,000,000 cells per well in the 6 well plate one day prior to the treatments. The endocytic pathway inhibitors Chlorpromazine hydrochloride, Filipin III, Amiloride Hydrochloride, Hydroxy dynasore, Monensis, Pitstop 2, and Methyl beta cyclodextrin (MβCD) were used at the concentrations mentioned in the figure legends for 2 hr. Later, cell suspensions containing inhibitors were incubated with fluorescence labeled MM-ZC or control DNAs at the concentration of 100ng/ml of million cells for an additional 2 hr. At the end of the treatment, inhibitor-treated cells were removed from the incubator and fixed the cells with 1% paraformaldehyde for 10 mins at room temperature. Subsequently, a permeabilizing solution (0.5% tritionX100 in PBS) was applied and cells were incubated for 10 mins at room temperature. Nuclei were stained with DAPI in PBS solution for an additional 5 mins. Cells were layered on coverslips and fluorescence signals were recorded using Zeiss700 microscope. Mean intensity of MM-ZC signals were calculated using ImageJ software and results were plotted as bar graphs.

### Quantification and statistical analysis

Number of biological replicates (BR) is stated in each legend. Error bars represent SD. The significance of difference p values for cellular assays was determined using the unpaired t-test (*p < 0.05; **p < 0.01). To determine the significant difference between samples, we used the Unpaired T-test.

## Results and discussion

We have previously demonstrated the HGT between human cancer cells via ctDNAs by employing multiple myeloma cell line models. Using chemically synthesized DNA sequence, we demonstrated the myeloma cell targeting by a specific sequence referred to as MM-ZC. This sequence belongs to the class of AluSp based on its sequence feature. We concluded that this type of ZCs could be efficient carriers of different chemotherapeutic payloads. These ZCs were also found to have a positive effect on treating cancer cells (results not published), suggesting that these carries have several advantages in drug delivery applications apart from their simple preparation from commercial starting materials. In order to better understand the effects of these MM-ZC in cancer cells, we have conducted a detailed and careful investigation of the MM-ZC uptake and intracellular trafficking.

### Time and concentration-dependence of MM-ZC uptake

**Figure 1:**
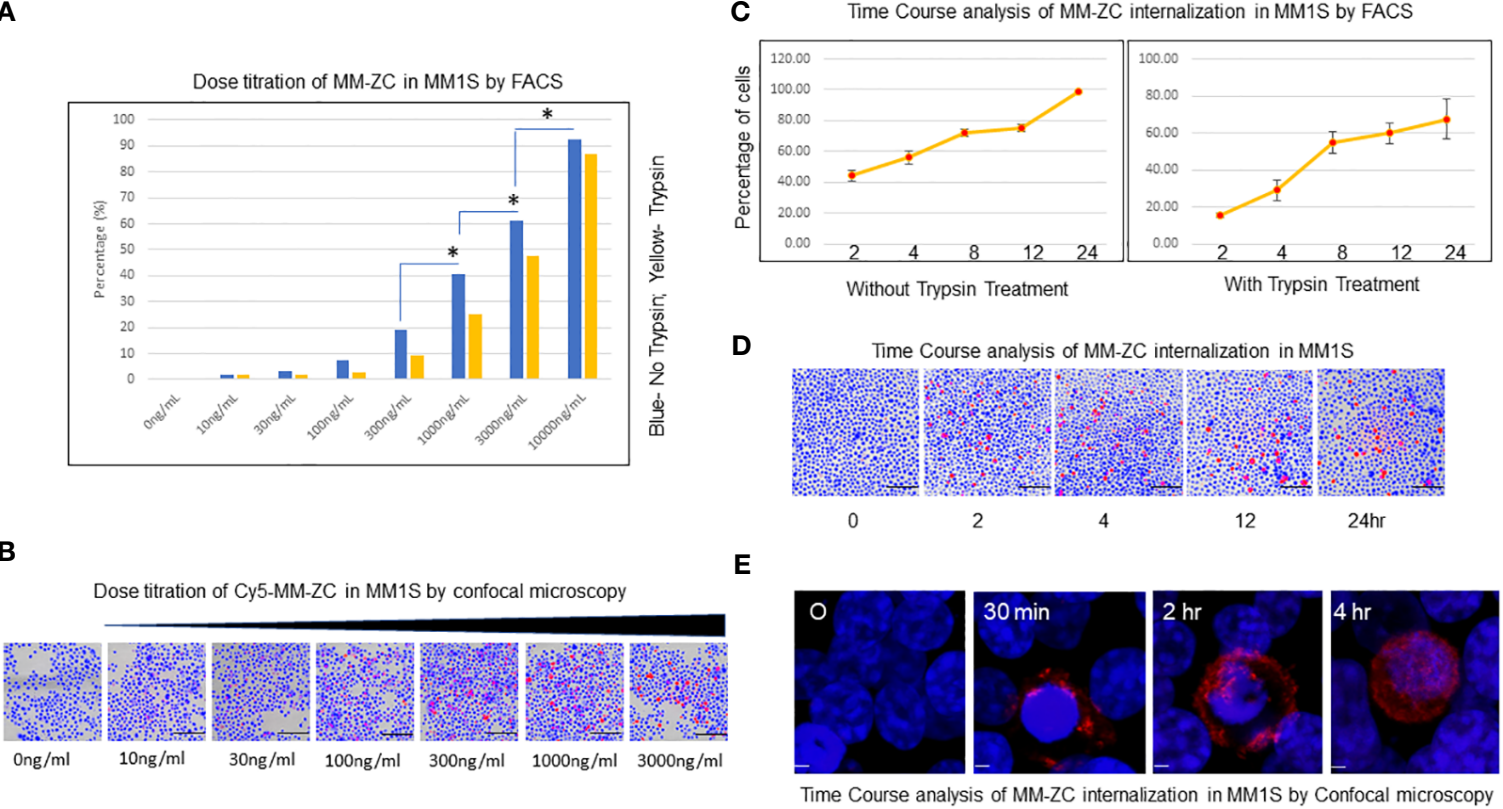
Dose titration and time course analysis of MM-ZC internalization in myeloma cells. **(A)** Dose titration of MM-ZC internalization by FACS analysis. MM1S cells were treated with various concentrations (as mentioned on X-axis) of Cy5 labeled MM-ZC DNA for 4 hr. The percentage of Cy5 positive cells is plotted on Y-axis. Trypsin-treated and untreated cells are represented as yellow and blue bars, respectively. * Indicates p<0.05 relative to the lesser concentration. The sample size for each point is 30 to 50K cells. **(B)** Representative microscopic image (selected from the ten random different field of view) of MM1S cells after 24 hr of incubation with various Cy5 labeled MM-ZC DNA concentrations. Scale bar 150 μm. Images are obtained using the ImageXpress micro confocal high-content imaging system. **(C)** Quantification of MM1S cells showed positive signals for Cy5 labeled MM-ZC at various time points. The left and right panels indicate the percent positive cells for Cy5 signal in trypsin untreated and treated MM1S cells. Percent positive cells for Cy5 signal is on Y-axis. The time points of the treatments are on X-axis. Error bars represent the standard error of mean (SEM) from 3 replicates. Each time point reflects the analysis of at least 10 x10^3^ cells. **(D)** Representative microscopic images (selected from ten random different field of view) of MM1S cells treated with 1μg/ml concentration of Cy5 labeled MM-ZC for various time points as mentioned below each panel. Scale bar 150 μm. Images are obtained by using the ImageXpress micro confocal high-content imaging system. **(E)** Representative 3D projection of Z-stack immunofluorescence images of MM1S cells showing the different uptake patterns of Rhodamine-labeled MM-ZC. The red fluorescence signal represents the MM-ZC, and the blue indicates the DAPI stained Nuclei. Images were taken after 0, 30 mins, 2, and 4 hr of treatment with 1 μg/ml of the MM-ZC. Scale bar 5 μm. Images are captured by using Zeiss 700 confocal microscopy.

The cellular uptake of MM-ZC was assessed using a multiple myeloma cell line (MM1S) to identify the characteristics of this unique non-viral transposon-based sequence. Intracellular uptake of MM-ZC in JK6L cells was detected by FACS analysis using various concentrations ranging from 0 to 10000ng/ml. The highest concentration employed for MM-ZC treatments did not affect the cells viability (**Supplementary Figure 1A**) or IFNγ expression (**Supplementary Figure 1B**). As shown in **Figure 1A**, the uptake of MM-ZC increased in a concentration-dependent manner. Alternatively, cells were treated briefly with trypsin in order to remove the loosely or non-specifically bound MM-ZC to the outside of the cell membrane to assess the percentage of cells that showed positive for the internalization of MM-ZC in MM1S cells. Significant internalization of MM-ZC (>60% cells showed MM-ZC uptake) was detected at 3000 and 10000 ng per ml of a million cells compared to no trypsin-treated cells (**Figure 1**, compare blue and yellow bars). At higher concentrations, both the samples showed a similar pattern (**Figure 1**, compare blue and yellow bars at 10 μg/ml) indicating the most of the MM-ZC is effectively internalized in MM1S cells. Similar response was observed in dose-dependent triplicate study in JK6L cells (**Supplementary Figure 1E**). These quantitative results are further confirmed by microscopic analysis. As shown in **Figure 1B**, images from “the ImageXpress micro confocal high-content imaging system” revealed that 100 to 3000ng/ml concentration of MM-ZC showed cellular uptake in MM1S cells. Higher concentrations of MM-ZC resulted in specific internalization in both quantitative and qualitative analysis. Next, the effects of time course on MM-ZC uptake were investigated. As shown in **Figures 1C, D**, the uptake of MM-ZC increased in a time-dependent manner. At 4 and 12 hr, MM1S cells showed significant cellular uptake of MM-ZC compared to 0 time points in both FACS and immunofluorescence analysis. As DNAs are negatively charged molecules, their affinity towards negatively charged cell membranes is low. However, MM-ZC overcomes these hurdles and effectively internalizes to cells indicating that the sequence might possess unique features, or the proteins that bind to the MM-ZC DNA sequence might be involved in reducing the negative repulsion between DNA and cellular membrane and activating the specific pathways that aid to the effective internalization of MM-ZC to MM1S cells. However, at the higher concentrations and prolonged exposure, MM1S cells demonstrated nonspecific uptake of any given DNA indicating that the identification of the optimal amount and time course are crucial for the development of MM-ZC as a deliverable vehicle. The internalization of MM-ZC by cells was further confirmed by confocal microscopy. **Figure 1E** shows a 3D projection of a Z-stack image of the cells treated with 100 ng/mL of MM-ZC for 30 min, 2 and 4 hr, which confirms the MM-ZC association with the membrane starts at early time points and internalization takes place the later time points. Red fluorescence indicates that the MM-ZC was associated with the outer membrane region as early as 30 min and completely internalized by 4 hr after the treatment. Thus, a 4 or 8 hr incubation time and a 100 or 300 ng/mL concentration were selected as the parameters for further studies. Next, we tested the effect of DNase1 on MM-ZC association with MM1S and JK6L. As shown in **Supplementary Figures 1C, D**, DNase1 treatment drastically reduced the Rhodamine signals of MM-ZC in both MM1S and JK6 cells. Overall intensity, membrane-bound and internalized MM-ZC signals were reduced in DNase1 treatment compared to the control, suggesting that MM-ZC behaves as expected as DNA molecule.

### Evaluation of MM-ZC uptake by different cell lines

**Figure 2:**
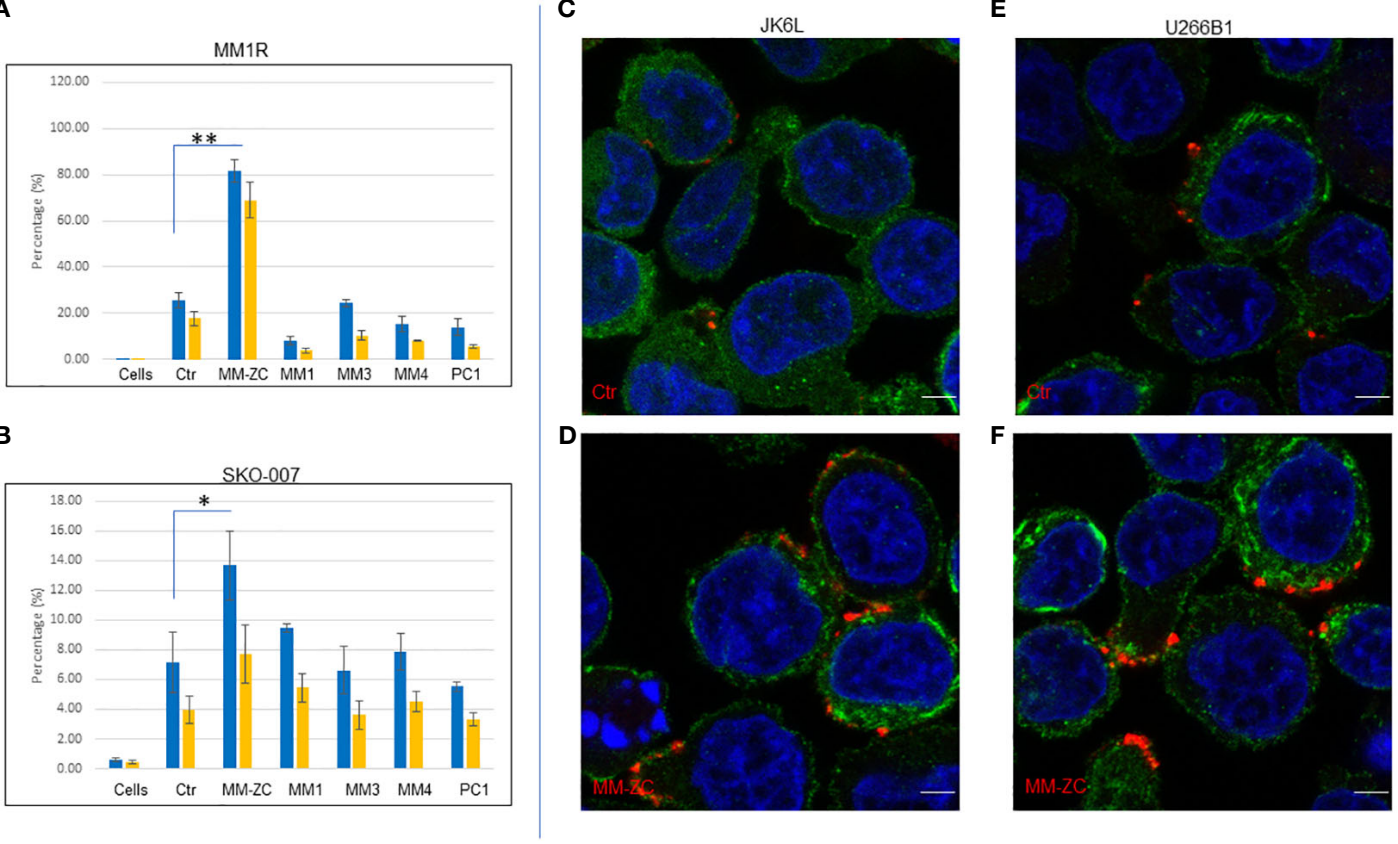
Cell type-specific uptake of various DNA molecules by FACS analysis. **(A, B)** Quantification of percent positive cells treated with six different Cy5 labeled DNA sequences (Ctr, MM-ZC, MM1, MM3, MM4, PC1) after 4hr of the treatments. Percent positive cells are on Y-axis. DNA treatments are on X-axis. * Indicates p<0.05 and ** indicates p<0.01 relative to control (cells only). Trypsin-treated and untreated cells are represented as yellow and blue bars, respectively. A = MM1R; B = SKO-007. Error bars represent the standard error of means (SEM) from 3 replicates. Sample size for each dose point is at least 10K. Unpaired T-test is used to determine the significant difference. **(C-F)** Representative confocal images (selected from ten random different field of view) of JK6L **(C, D)** and U266B1 **(E, F)** cells showing the different uptake amounts of Rhodamine-labeled Ctr (top panel) MM-ZC (bottom panel). The red and green fluorescence signal represents the DNA and phalloidin (membrane) staining respectively; the blue indicates the DAPI stained Nuclei. Images were taken after 4 hr of treatment with 100 ng/ml of the MM-ZC. Scale bar 10 μm. Images are captured by using Zeiss 700 confocal microscopy.

In order to extend the scope of this work, we decided to test the efficacy of two different cell types from the myeloma origin for their internalization preference for different non-specific DNA sequences after 4 hr of treatment with a dosing concentration of 1000 ng/ml cells. Direct comparison of MM-ZC with other DNA sequences demonstrated the specific and significant internalization of MM-ZC in MM1R, other widely used myeloma cell lines. This result is similar to that of MM1S cells whose data is present in elsewhere (1) and demonstrated a significant uptake of MM-ZC compared to the other DNA sequences (**Figure 2A**, compare MM-ZC bar graph with other DNA sequences). Both trypsin-treated and untreated cells showed similar patterns. However, SKO-007 cells showed the lowest uptake of MM-ZC DNA compared to other myeloma cells. However, compared to control sequence, they did show significant uptake of MM-ZC (**Figure 2B**) compared to other DNAs. This is probably a result of the low expression of specific factors or proteins that bind or mediate the MM-ZC internalization process and/or non-specific uptake of DNAs due to the phagocytosis process in which cells engulf foreign particles. Next, we tested the localization pattern of MM-ZC in two different myeloma cell types by using confocal microscopy. As shown in **Figures 2C, D**, JK6L cells demonstrated a distinct punctate pattern of MM-ZC in the surrounding outer membrane regions compared to the control sequence (**Figure 2C**). U266B1 cells showed a similar internalization pattern for MM-ZC (**Figure 2F**) compared to Ctr (**Figure 2E**). However, MM1S cells showed a uniformly distributed MM-ZC signal in cytosolic and membrane regions (**Figure 1E**). These variations in the cellular uptake for the same type of MM-ZC in different cell lines have been reported previously and suggested that the membrane composition, receptor expressions, and endocytosis mechanisms significantly contribute to the intake and cellular trafficking of the external molecules to the internal destinations (21–23).

### Studies on the mechanisms of MM-ZC uptake

**Figure 3:**
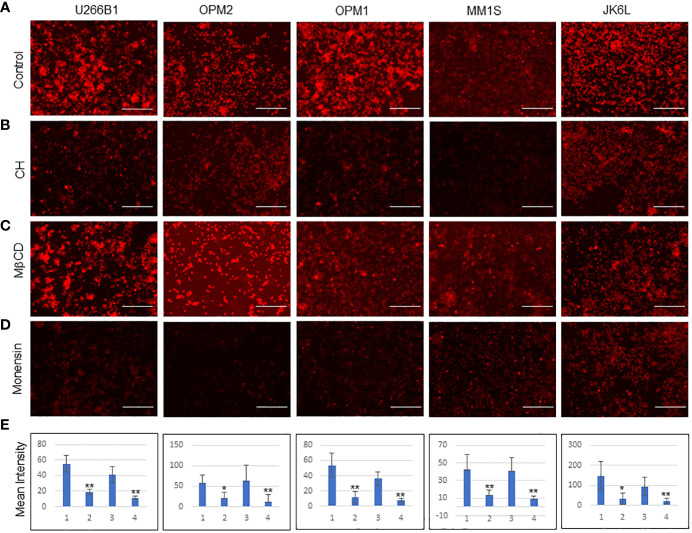
Effect of endocytosis inhibitors on the cellular uptake of MMZC in different myeloma cell types **(A-D)** Representative immunofluorescence images (selected from five random different field of view) of U266B1, OPM2, OPM1, MM1S, and JK6L cells after 2 hr of the Rhodamine-labeled MM-ZC treatment. Scale bar, 150 μm. Inhibitors (**A** = control cells; **B** = Chlorpromazine hydrochloride (CH), **C** = Methyl beta cyclodextrin (MβCD), **D** = Monensin) treatments are mentioned on the left side of the panels. Images were taken by Invitrogen EVOS M5000 microscope. **(E)** Quantifying the mean intensity of the Rhodamine signal from 50 randomly selected cells from five different fields of view indicates the reductions of cellular uptake of Rhodamine-labeled MM-ZC in response to the inhibitor’s treatments. * Indicates p<0.05 and ** indicates p<0.01 relative to control. Error bars represent the standard deviation. Sample size corresponds to 5% of the total cell seeded on the coverslip. 1= Untreated, 2= Chlorpromazine hydrochloride, 3= MβCD and 4= Monensin treatments in bar graphs. Error bars represent the standard deviation of three independent experiments.

As a first step towards an understanding of how MM-ZC internalizes, we examined in an unbiased fashion by performing an inhibitory analysis. We have selected eight known inhibitors that were proven to be effective against major endocytosis pathways. All the inhibitors were used according to the manufacturer’s protocols, which are also reported from various research groups for different cell lines to achieve transport inhibition (24, 25). One important requirement when using endocytosis inhibitors is that they should not affect cell viability or survival, as a side effect may impact the uptake processes of MM-ZC, thereby interfering with the data and leading to the pleiotropic effects occurring simultaneously. Cellular uptake of molecules known to internalize cells via a specific pathway were used as positive controls when possible. However, this was not possible in myeloma cells due to the lack of established molecules or thorough investigations of endocytosis mechanisms. The second caveat for the usage of inhibitors is short exposure times. Because it has been known that blocking one pathway for prolonged exposure can result in the activation or suppression of other alternative endocytic mechanisms, which again confounds the results. Based on this, it is essential to test the toxicity of each inhibitor on MM1S cells. **Supplementary Figures 2A, B** shows the cell viability of MM1S cells after 8 and 4 hrs of treatment with each inhibitor at various concentrations. Results indicated that higher concentrations of the inhibitors significantly reduced the cell viability as assessed by the cell titer blue assay. However, test concentrations implemented for inhibitors study in our experiments, as marked by the green arrow, did not show much effect on the viability of the cells (**Supplementary Figure 2B**). Next, we treated MM1S cells with the above-mentioned eight inhibitors for 2 hr followed by an additional 2 hr incubation with Rhodamine-labeled MM-ZC and assessed the effect of each inhibitor on MM-ZC signal in MM1S cells. Monensin Sodium and Chlorpromazine hydrochloride (CH) decreased the overall intensity of MM-ZC signal in MM1S cells compared to non-treated cells (**Figure 3**; **Supplementary Figures 3.1, 3.2**). Hydroxy dynarose and Filipin III reagents showed no effect on MM-ZC internalization (**Supplementary Figure 3.2** panels), indicating that dynamin and caveola-dependent endocytosis pathways are not potential routes for the internalization of MM-ZC. Similarly, Amiloride hydrochloride, which is used to define the role of micropinocytosis, and Pitstop, which is an inhibitor of the Clathrin-dependent and independent endocytosis pathways, had a modest effect on the MM-ZC internalization in MM1S cells (**Supplementary Figures 3.2C, H**), suggesting the possibility of other pathways influencing MM-ZC internalization. These preliminary results encouraged us to test the direct effect of selective inhibitors on five different myeloma cell lines (U266B1, OPM1, OPM2, MM1S and JK6L) to address if MM-ZC utilizes the same pathway or not. Based on the previous results, we selected Monensin, CH and MβCD to test their effect on MM-ZC internalization. Equal number (10x^6^) of cells were treated with each of the inhibitors and analyzed their cellular uptake of MM-ZC after two hr of the post-treatment. **Supplementary Figure 3.1** represents the bright field images of **Figure 3**. As shown in **Figures 3B, D** panels, immunofluorescence analysis revealed that CH and Monensin treatments significantly diminished the intensity of MM-ZC signal in all the five cell types compared to control (**Figure 3A**) or MβCD treatment (**Figure 3C**). These results conclude that endosomal maturation and Clathrin-mediated endocytosis are the primary mechanisms that aid MM-ZC to internalize in myeloma cells. In addition, the components of cholesterol in the cell membrane also play an important role in cell-type specific manner in the translocation processes of MM-ZC. To quantify the effect of MM-ZC internalization inhibition, we measure the mean intensity of the MM-ZC signal by image J analysis. **Figure 3E** showed that a significant reduction of mean intensity of MM-ZC was observed in CH and Monensin treatments. Therefore, both qualitative and quantitative results provide ample evidence that MM-ZC internalization may be dependent on the Clathrin-mediated endocytosis and endosomal maturation mechanisms in myeloma cells.

### Intracellular trafficking of MM-ZC with the aid of cell membrane and endocytosis proteins

**Figure 4:**
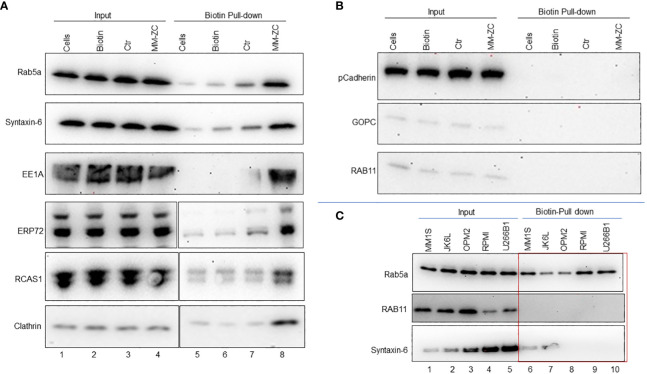
Endosomal markers and other membrane-associated factors interact with MM-ZC but not with Ctr or biotin in JK6L cells. **(A, B)** Streptavidin pulldown of biotin-labeled control (Ctr) and MM-ZC followed by immunoblotting to examine the interaction of MM-ZC with Rab5a, Syntaxin-6, EE1A, ERP72, RCAS1, Clathrin, Cadherin, GOPC, and Rab11 in JK6L cells. Lane 1-4 indicates Input samples. Lanes 5, 6, 7, and 8 indicate pulldown samples from untreated cells, biotin alone, biotin-labeled Ctr, and biotin-labeled MM-ZC treated JK6L cells. Compare lanes 7 and 8 to see the enrichment of different proteins in MM-ZC pulldown samples relative to Ctr. Input lanes show the expressions of relevant proteins, as mentioned on the left side of each panel in JK6L cells. We used 1000 μg of total isolate for each pulldown experiment. **(C)** Immunoblots for Rab5a, Rab11 and Syntaxin-6 proteins after pulldown by streptavidin beads from MM1S, JK6L, OPM2 and U266B1 cell lysates treated with biotin labeled MM-ZC for 4 hr. Cell lines are listed at top of panel and immunoblotting antibodies are left. Lanes 1-5 represent the expression of proteins in the whole cell lysate used as a input material for the pulldowns. The red box highlights the cell type specific interactions of MM-ZC with Rab5a, RCAS1 and CD98 but not with Rab11 and Rab7. We used 1000 μg of total isolate for each pulldown experiment.

Endocytosis is an orchestrated process executed by a plethora of factors in a spatiotemporal manner. The invagination of the plasma membrane forms endosomes to shuttle the factors across the membrane (26). Clathrin and caveolins are essential proteins of the plasma membrane and in the formation of endosomes (27, 28). A unique set of proteins marks each stage of endosome maturation. EEA1 is an early endosome marker essential for membrane fusion and trafficking (29). Members of small Rab GTPases, specifically Rab5a, Rab7, and Rab11, are markers of the early, late, and recycling endosomes (30). We reasoned that identifying MM-ZC interaction with such endosomal marker proteins might hold the key to understanding the mechanical process of MM-ZC intracellular trafficking in myeloma cells. To this end, we first determined the association between biotin-labeled MM-ZC and known endosomal markers such as Rab5a, 11, Clathrin, and EEA1 in biotin pulldown samples using whole-cell lysates prepared from JK6L cells treated with biotin labeled MM-ZC for eight hr (**Figure 4**). The eluted proteins were subjected to immunoblotting with specific antibodies against the above mentioned proteins, revealing that MM-ZC is associated with Clathrin, Rab5a, and EEA1 (**Figure 4A**, lane 8) proteins but not Rab11 (**Figure 4B**, lane 8). Ctr sequence or biotin alone treated sample failed to correlate with Rab5a or Clathrin (**Figure 4A**, lane 6 and 7). Next, we asked if MM-ZC is part of any other membrane-associated complexes, for which we utilized Cadherins, which are transmembrane glycoproteins that mediate calcium-dependent cell-cell adhesion (31) and Ectonucleotide pyrophosphatase-phosphodiesterase 1 (ENPP1) which is a type II transmembrane protein primarily involved in ATP hydrolysis at the plasma membrane (32). As shown in **Figure 4A**, Immunoblotting analysis of Biotin-labeled MM-ZC pulldown samples revealed that MM-ZC failed to associate with Cadherin (**Figure 4B**) and ENPP1 (data not shown), which also serves as a negative control to demonstrate the specificity of MM-ZC association with specific proteins. These results thus suggest that MM-ZC is part of membrane-associated Clathrin and Rab family proteins.

Several proteins act as mediators between the outside membrane and internal organelle and aid in the shuttling of external factors. Because of their direct involvement, recent studies have uncovered several bidirectional transport routes between endosomes and the Golgi complex (33). Similarly, endosomes and Golgi complex are also in contact with ER, ensuring the coordination of molecular activities between these compartments (34). Based on these associations, we hypothesized that MM-ZC might also be associated with some ER and Golgi network factors. We employed antibodies against known ER and Golgi apparatus proteins such as the ER stress protein 72 (ERp72) (35), the tumor-associated antigen RCAS1 (36), and Syntaxin-6 (37). Immunoblot analysis of biotin-labeled MM-ZC pulldown samples revealed a stronger association of MM-ZC with RCA1, ERP72 and Syntaxin-6 (**Figure 4A**, lane 8). Next, we sought to identify if these observed interactions are consistent in other myeloma cell types. We performed biotin pulldown assays using lysates from MM1S, JK6L, OPM2, RPMI and U266B1 cells treated with biotin labeled MM-ZC. Immunoblotting with antibodies against Rab5a showed a consistent association of Rab5a with MM-ZC in all the myeloma cell types (**Figure 4C**). Interestingly, GOPC and Rab11 failed to show an association with MM-ZC in either of the cell types tested (**Figure 4C**, lower and middle panel). To our surprise, syntaxin-6 showed a positive association with MM-ZC in MM1S and JK6L cells (**Figure 4C**, bottom panel) but not in OPM2, RPMI and U266B1, suggesting that some of the organelle-specific proteins interact with MM-ZC in cell-type specific manner. These findings further support the hypothesis that MM-ZC interacts with a ubiquitous set of proteins at the membrane levels and with different sets of proteins in downstream organelles in a cascade manner before integrating into the genome. Secondly these data also indicates that the cell type-specific associations are the causative factor for the cellular differences of MM-ZC internalization in different myeloma cell types.

**Figure 5:**
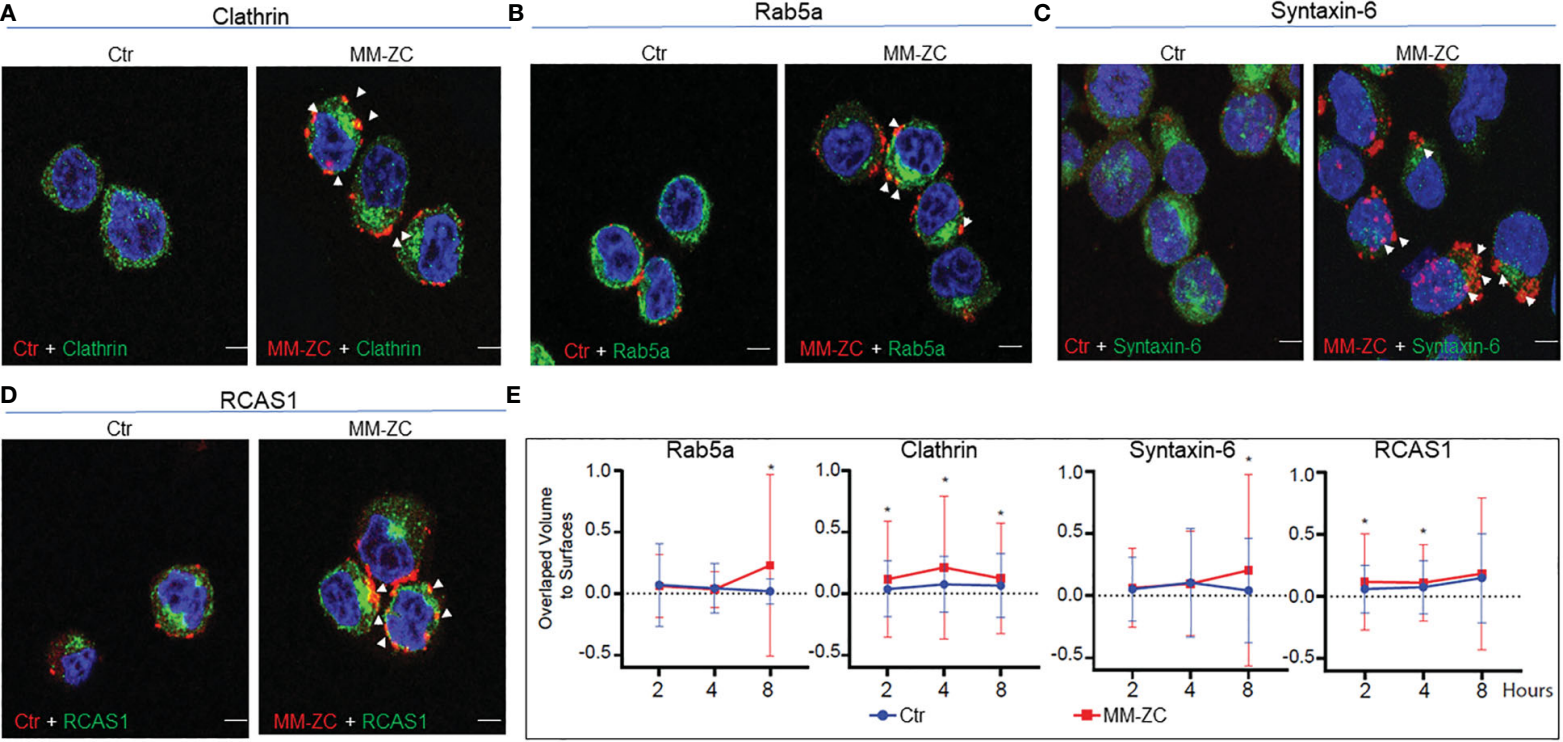
Colocalization of MM-ZC with various endosomal markers and other membrane-associated factors in JK6L cells. **(A-D)** Representative (selected from ten random different field of view) anti-Clathrin, Rab5a, Syntaxin-6 and RCAS1 immunofluorescence signal of JK6L cells treated with Rhodamine-labeled MM-ZC for 8 hr. Images are captured by using Zeiss 700 confocal microscopy. All the proteins showed colocalization with MM-ZC. The left panel represents the Ctr signal (red), and the right panel corresponds to MM-ZC (red). In both panels, Alexa Flour 488 corresponds to the antibody specific to the protein mentioned on top of the image panels (green); Nuclei is stained with DAPI (blue). Arrows indicate the yellow colored colocalized areas of MM-ZC (red) with endogenous proteins (green). Scale bars for all the images = 10 μm. **(E)** Quantitative analysis of MM-ZC colocalization with Rab5a, Clathrin, Syntaxin-6 and RCAS1 in JK6L cells. 1 x 10^6^ cells were incubated with Rhodamine-labeled DNA (MMZC or Ctr sequence) at different time points and performed the immunofluorescence analysis as mentioned in the methods. Images were acquired using a Leica SP8 LIGHTING confocal microscope from quadruplicate experiments. The 3D visualization and colocalization was performed using surfaces were created in Bitplane Imaris 10. Colocalization of DNA and proteins was measured using overlapped volume to surface measurement (plotted on Y-axis; Treatment time on x-axis). * indicates the significant difference between ctr and MM-ZC. Unpaired T-test is used to determine the significant difference. Error bars represent the standard deviation from quadruplicate experiments.

To extend the physiological relevance of this interaction, we carried out immunofluorescence between endogenous proteins and Rhodamine-labeled MM-ZC. As shown in **Figures 5A, B**, high colocalization of MM-ZC with Clathrin and Rab5a (yellow color) was observed when compared to the control sequence. These results argue that not all the DNA sequences follow the same uptake route as that of MM-ZC, but for some undiscovered reason, MM-ZC effectively associates with Clathrin and early endosomal protein, Rab5a (Zoom in images and 3D projection videos are provided in **Supplementary Figures 5.2A, B, E, F** respectively). Furthermore, ER and Golgi organelle associated proteins interaction with MM-ZC was studied to confirm the *in vitro* biotin pulldown results hold the truth *in vivo*. As demonstrated in **Figures 5C, D**, MM-ZC in MM1S cells was associated with Syntaxin-6 and RCAS1 (Zoom in images and 3D projection videos are provided in **Supplementary Figure 5.2C, D, G, H** respectively). We did not find much colocalization between the Ctr sequence and the above-mentioned proteins.

Next, we investigated the time-dependent association MM-ZC with Clathrin, Rab5a, Syntaxin-6, and RCAS1 in JK6L cells. One million cells were incubated with Rhodamine-labeled DNA (MM-ZC or Ctr sequence) for 2, 4, and 8 hr and subjected to immunofluorescence analysis with specific antibodies, as mentioned in the figure labels. Images from quadruplicate experiments were acquired using a Leica SP8 LIGHTING confocal microscope, and the colocalization DNA and proteins were measured using overlapped volume-to-surface measurement. As shown in **Figure 5E**, MM-ZC associates with Clathrin, a membrane-associated protein, as early as 2 hr of treatment, peaking its association by 4 hr and subsequently reduced by 8 hr. Unlike Clathrin, cytosolic proteins Rab5a and Syntaxin-6 demonstrated negligible association at early time points and stronger association at 8 hr of the treatment. These results highlight the dynamic process of how different proteins associate with MM-ZC at distinctive time points and based on their localization in various cellular compartments. Being a cell membrane protein, Clathrin gets early access to associate with MM-ZC and facilitates its membrane association, followed by internalization to the downstream molecules. Therefore, by 8 hr, Clathrin-associated MM-ZC is reduced. At the same time, MM-ZC association with internal endosomal protein and Golgi-apparatus proteins increased, indicating the dynamic stepwise internalization process aid of cellular proteins. We thus conclude that MM-ZC targets different sets of proteins during its internalization process, and we envisage that these associations are cell-specific, time- and sequence-dependent.

To conclusively address that these interactions are sequence-dependent, we carried out an experiment to test the colocalization between Rab5a and four different DNA sequences by immunofluorescence analysis. As shown in **Supplementary Figure 5.1A**, Rab5a effectively colocalized with MM-ZC but not with other DNAs such as Ctr, pancreatic cancer 1 (Pc1) or vector sequence (compare panels among A-D). These confocal results were further confirmed by biotin-pulldown analysis. As demonstrated in **Supplementary Figure 5.1E**, Rab5a only associates with MM-ZC but not any other DNA sequences that we tested in biotin-pull down assay followed by immunoblotting with specific antibodies. Collectively the above results support the notion that MM-ZC takes advantage of early endosomal proteins to internalize the cells effectively. We leave open the possibility that other sequences may associate with different sets of proteins through additional mechanisms. We also tested the association of Rab7 (**Supplementary Figure 5.1F**) with different DNA sequences; interestingly, none of the DNAs interacted with Rab7 in biotin-pull down assay. It also served as a negative control to show the specificity of MM-ZC towards Rab5a protein but not with any other Rab family proteins. A well-known bottleneck in drug or gene delivery into the cytosol of cells is the lysosomal escape of vehicles. Since MM-ZC is not associated with Rab7, a lysosomal protein, we observed an effective internalization of MM-ZC to MM1S cells. Based on these results, we inferred that cellular uptake and trafficking of MM-ZC is mainly dependent on the specific set of membrane and endosomal trafficking proteins in a spatial-temporal manner.

### Loss of Clathrin, Rab5a and Syntaxin-6 reduces MM-ZC internalization in myeloma cells

**Figure 6:**
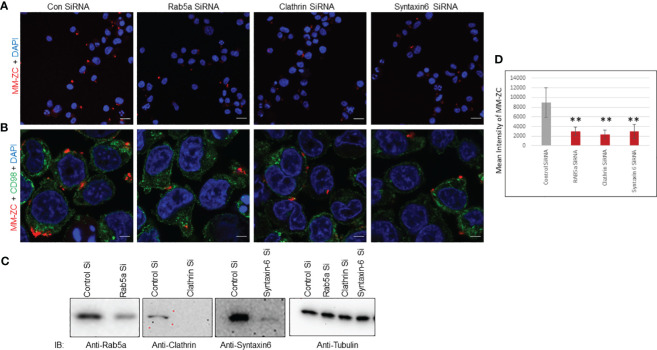
Rab5a, Clathrin, and Syntaxin-6 knockdown reduce MM-ZC internalization in JK6L cells. **(A, B)** Immunofluorescence analysis of Rhodamine-labeled MM-ZC in JK6L cells transfected with control (luc) and Rab5a, Clathrin, and Syntaxin-6 siRNAs. All the images in **(A)** represent the 20x zoom images with a scale bar of 150 μm. **(B)** Depicts the 60X zoom with a scale bar of 10 μm. Rhodamine MM-ZC signal = Red, CD98 as a membrane marker = green, Nuclei DAPI = blue. Images are captured by using Zeiss 700 confocal microscopy. **(C)** Immunoblots showing knock down of Rab5a, Clathrin, and Syntaxin-6 in JK6L cells. Tubulin is loading control. **(D)** Quantification of mean intensity of MM-ZC signal from 50 randomly selected cells in each field in response to the knockdown of different proteins. * Indicates p<0.05 and ** indicates p<0.01 relative to control. Error bars indicate the standard deviation from all the 50 randomly selected cells from three replicates. Unpaired T-test is used to determine the significance. Sample size corresponds to 1% of total cells spotted on the cover slip for the microscopic analysis.

Several studies have shown that Clathrin and Rab5a proteins play vital role in the endocytosis of viruses, nanoparticles, and peptides. These proteins function independently or in complex and participate in the safe passage of nutrients from outside to inside the cell. Hence, the silencing of either Rab5a or Clathrin proved to be a roadblock for the uptake of many external factors. Therefore, we began investigating whether the depletion of these proteins could influence the internalization of MM-ZC in myeloma cells. We knock down the Clathrin, Rab5a, and Syntaxin-6 proteins by neon transfection in JK6L cells with specific pool of siRNAs corresponding to each protein under investigation. **Figure 6C** panels show the effective knockdown of Clathrin, Rab5a, and Syntaxin-6 proteins compared to the negative control (luc siRNA). Furthermore, silencing of Clathrin, Rab5a, and Syntaxin-6 decreased the cellular uptake of MM-ZC in JK6L cells as assayed by Immunofluorescence analysis (compare the Rhodamine signals of MM-ZC in proteins knockdown cells and luc siRNA transfected cell in both **Figure 6A** (20X confocal image field), 6B (60X confocal image field). Staining of the cells with CD98 marker (green channel) in **Figure 6B** aided us in demarcating the cell membrane and visualizing how MM-ZC associates with the membrane of myeloma cells. The mean intensity of the MM-ZC signal was measured from a pool of 50 cells in each siRNA-treated sample using imageJ software analysis. Consistent with the confocal observations, the mean intensity of MM-ZC fluorescence signal was significantly decreased in Clathrin, Rab5a, and Syntaxin-6 knockdown myeloma cells (**Figure 6D**). MM-ZC internalization in an additional myeloma cell line MM1S also showed a similar reduction by loss of Rab5a and Clathrin (**Supplementary Figures 6A-D**, compare the fluorescence signal of MM-ZC between control and protein knockdown panels). These results argue that Clathrin, Rab5a and Syntaxin-6 actively and specifically contribute to the cellular uptake of MM-ZC in myeloma cells.

### Interdependency among Rab5a, Clathrin and Syntaxin-6 on MM-ZC internalization

**Figure 7:**
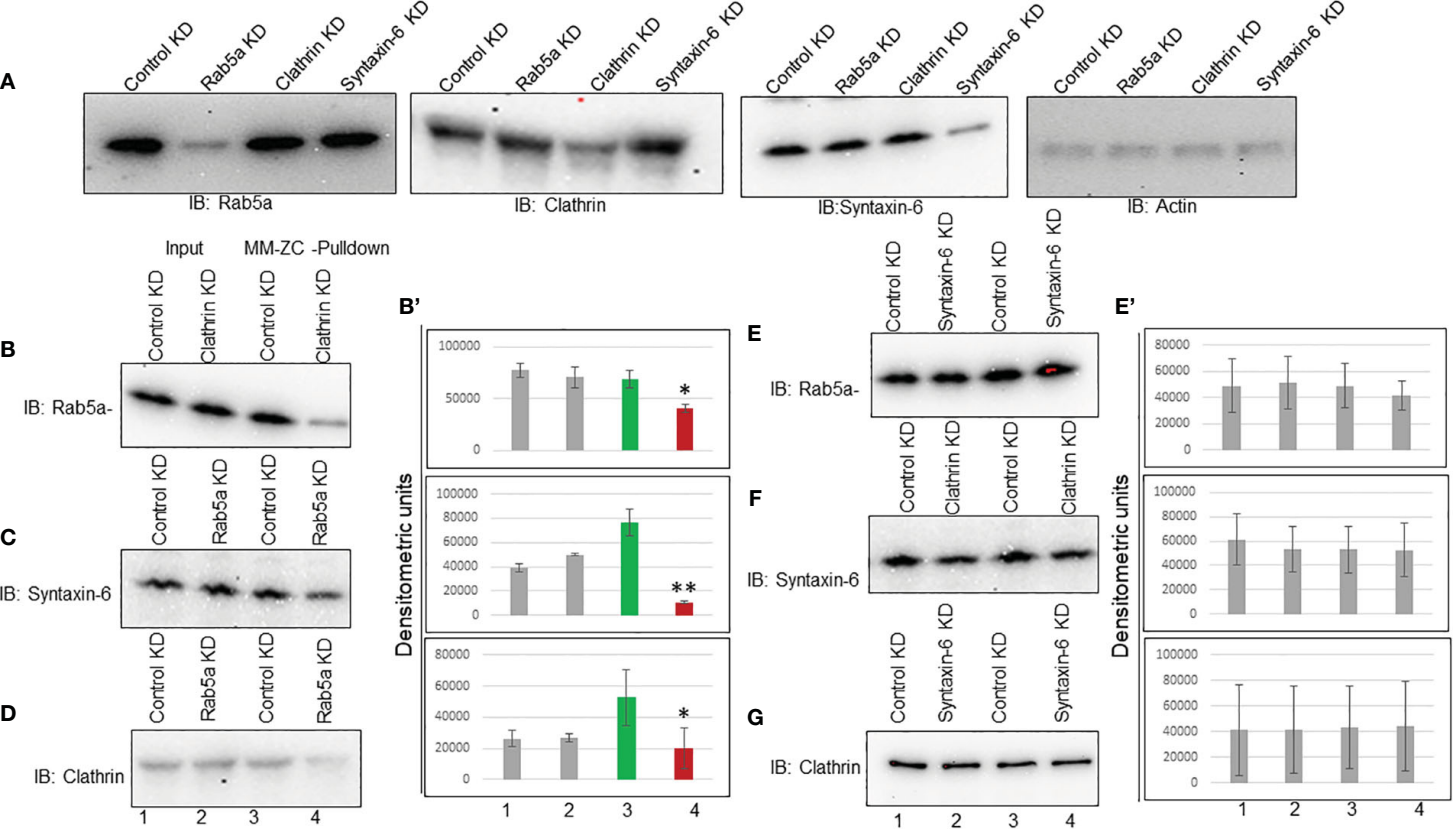
Interdependency among Rab5a, Clathrin, and Syntaxin-6 in MM-ZC internalization in JK6L cells. **(A)** Immunoblots showing Rab5a, Clathrin, and Syntaxin-6 knockdown in JK6L cells. **(B-G)** Immunoblots for Rab5a, Syntaxin-6, and Clathrin proteins on biotin pulldown samples from Luc, Rab5a, Clathrin, and Syntaxin-6 siRNAs transfected JK6L cells. Knockdown of protein names are listed at the panel’s top and immunoblotting antibodies are at the left. Lanes 1 and 2 represent the input material; Lanes 3 and 4 illustrate the biotin pulldown samples. **(B’, E’)** Bar graphs representing the densitometric analysis of **(B–G)** immune blots. Error bars represent the standard deviation of three independent experiments. * Indicates p<0.05 and ** indicates p<0.01 relative to control.

The above results demonstrated that MM-ZC internalization depends on Rab5a, Clathrin, and Syntaxin-6 expression. Previous studies showed that Rab5a is essential in Clathrin vesicle formation (38). Therefore, Brucella abortus entry and early intracellular trafficking depend on the cooperation between Clathrin and Rab5a (39). Even though Syntaxin-6 is implicated in the trans-Golgi network, its association with endosome trafficking is well established by demonstrating its interaction with Rab5a effector protein EE1A (40). Hence, these proteins might function in a stepwise signaling pathway that facilitates MM-ZC traveling from the membrane to the nucleus. If that’s the case, the knockdown of one protein could destabilize the association of MM-ZC with the other downstream proteins. First, we tested the knockdown effect of each protein on the expression levels of all other proteins. Immunoblotting showed that transfection with Rab5a siRNAs decreased Rab5a expression levels but not Clathrin or syntaxin-6 (**Figure 7A**, Rab5a Panel). A similar pattern was obtained for Clathrin and syntaxin-6 (**Figure 7A**, Clathrin and syntaxin-6 panel). The knockdown of each protein reduced its expression but did not affect other proteins’ expression. Actin immunoblot served as a loading control. Transfection of control siRNA had no apparent effect on either the expression of Rab5a, Clathrin, or syntaxin-6. Next, we assessed the functional consequence of knock down of one protein on the other proteins’ association with MM-ZC. We started with Clathrin depletion and asked if it influences the interaction of Rab5a or syntaxin-6 with MM-ZC. To this end, we prepared the lysates from MM-ZC treated and Clathrin siRNAs transfected JK6L cells and performed the biotin-pulldown analysis. As shown in **Figure 7**, Clathrin depletion disrupted the MM-ZC association with Rab5a (**Figure 7B**, compare lane 3 and 4) but not Syntaxin-6 (**Figure 7F**, compare lane 3 and 4), suggesting that Clathrin functions upstream to the Rab5a but not to the syntaxin-6. Next, we tested the effect of Rab5a depletion on Syntaxin-6’s association with MM-ZC. Results show that the association of syntaxin-6 with MM-ZC is partly dependent on Rab5a knockdown (**Figure 7C**, compare lane 3 and 4) cells, indicating that Rab5a is acting upstream to the Syntaxin-6. Taking the results from Clathrin and Rab5a knockdown cells, we conclude that Clathrin and Rab5a act upstream to Syntaxin-6 as entry points to MM-ZC trafficking. Next, we asked if the knockdown of Syntaxin-6 has any effect on the MM-ZC association with Rab5a or Clathrin. Biotin-pulldown results indicate that the reduction of Syntaxin-6 did not affect the Rab5a (**Figure 7E**, compare lanes 3 and 4) or Clathrin (**Figure 7G**, compare lanes 3 and 4) association with MM-ZC, highlighting that the Rab5a or Clathrin proteins associate with MM-ZC independent of Syntaxin-6 expression, suggesting that these two proteins act upstream to the trans-Golgi network processes. As Rab5 and Clathrin are mutually involved in the vesicle formation and early endosomal processes, we asked if the knockdown of Rab5a regulates the Clathrin association with MM-ZC. As shown in **Figure 7**, Clathrin interaction with MM-ZC is reduced in Rab5a knockdown cells (**Figure 7D**, compare lanes 3 and 4), indicating that both proteins influence the association of both the proteins with MM-ZC, possibly due to functioning as a complex at the membrane invagination, vesicle formation, and early endosomal maturation. Densitometric analysis of biotin-pull down from triplicates reconfirmed the immunoblotting assay results (**Figures 7B’, 7E’**). To test whether the observed binding of Clathrin, Rab5a and Syntaxin-6 with MM-ZC can take place *in vivo* under the knockdown of different proteins, we performed double staining of Rab5a/Clathrin/Syntaxin-6 with Rhodamine-labeled MM-ZC in cells transfected with Clathrin (**Supplementary Figure 7B**) and Rab5a siRNAs (**Supplementary Figures 7D, F**). As seen in **Supplementary Figures 7A, C, E**, Rab5a, Clathrin, and Syntaxin-6 are colocalized with MM-ZC. MM-ZC is concentrated in outer membrane regions and is also present at lower concentrations in the cytoplasm. Furthermore, because the association of Rab5a and Clathrin with MM-ZC is mutually interdependent, we tested whether the knockdown of one protein affects the association of other proteins with MM-ZC. We found that knockdown of Clathrin (compare **Supplementary Figure 7A** with **7B**) or Rab5a (compare **Supplementary Figures 7C** with **7D**) reduced the internalization of MM-ZC; as a result, we could not detect any colocalization between these proteins and MM-ZC in knockdown cells. Similar results were observed with Syntaxin-6 in Rab5 knockdown cells (compare **Supplementary Figures 7E** with **7F**). Therefore, these results can partially support the loss of association between Rab5a and Syntaxin-6 proteins with MM-ZC by demonstrating that less MM-ZC is available to internalize. Hence, based on both coimmunoprecipitation and colocalization results, we conclude that the knockdown of Clathrin and Rab5a profoundly affects MM-ZC internalization, thereby affecting the association of downstream proteins with MM-ZC molecules.

### MM-ZC enhances the interaction between Rab5a and Clathrin

**Figure 8:**
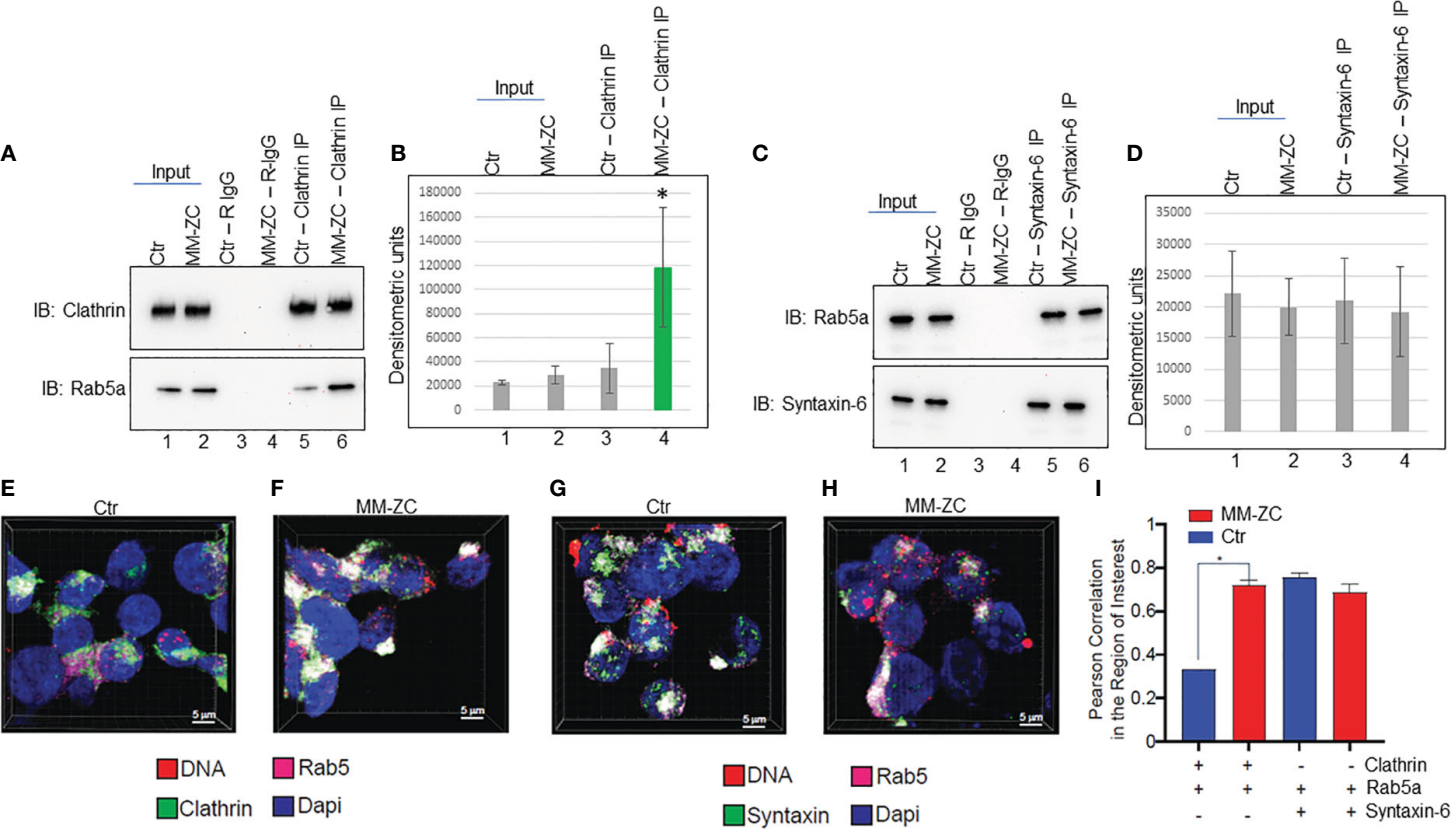
MM-ZC treatment increases the association between Rab5a and Clathrin. **(A, C)** Co-immunoprecipitation to assay Rab5a interaction with Clathrin and Syntaxin-6 in ctr or MM-ZC treated MM1S cells. Lanes 1 and 2 represent 20% of the Input sample. Lanes 3 and 4 indicate the rabbit-IgG immunoprecipitated samples from control and MM-ZC treated cell lysates. Lanes 5 and 6 represent antibody-specific immunoprecipitated samples. Antibodies used in immunoprecipitation and immunoblotting have been mentioned on the top and left side of the panels, respectively. **(B, D)** Bar graphs representing the densitometric analysis of **(A, C)** immune blots. Error bars represent the standard deviation of three independent experiments. * Indicates p<0.05 and ** indicates p<0.01 relative to control. **(E–H)** Representative immunofluorescence images (selected from ten different randomly captured field of views) of Rab5 colocalization with Clathrin **(E, F)** and Syntaxin-6 **(G, H)** in MM1C cells treated with Ctr **(E, G)** and MM-ZC **(F, H)** for 8 hr. Individual channels (Rab5a = Cy5, Magenta, Clathrin/Syntaxin-6 = Alexa fluor 488, Green). Rab5a is probed with anti-Rab5a and Alexa Fluor 647-conjugated secondary mouse IgG, whereas Clathrin and Syntaxin-6 are probed with their respective individual specific primary antibodies followed by Alexa Fluor 488 -conjugated secondary rabbit IgG. Scale bar: 5μm. **(I)** Quantitative analysis of Rab5a colocalization with Clathrin and Syntaxin-6 MM1S cells. Images from quadruplicate experiments were acquired using a Leica SP8 LIGHTING confocal microscope. The 3D visualization and colocalization was performed using Bitplane Imaris 10. The colocalization of two proteins was measured by Pearson correlation in the area of interest (plotted on the Y-axis). * Indicates the significant difference between ctr and MM-ZC. Unpaired T-test is used to determine the significant difference.

The aggregate of the above results prompted us to investigate whether MM-ZC regulates the interaction between Clathrin and Rab5a. To this end, we carried out co-immunoprecipitation between endogenous Rab5a and Clathrin in whole cell lysates prepared from MM1S cells ectopically treated with either control or MM-ZC sequence. As shown in **Figure 8A** top panel, Clathrin specific antibody immunoprecipitated equal amount of Clathrin in both the treatments. Input samples indicate that treatment of cells with ectopic DNAs did not alter the protein amount of Clathrin or Rab5a. Interestingly, immunoblotting of Clathrin immunoprecipitated samples with Rab5a specific antibody revealed a significant increase of interaction between Clathrin and Rab5a in MM-ZC treated cells but not in control sequence treated cells (compare lanes 5 and 6 in **Figure 8A** bottom panel). Densitometric analysis of both the protein bands reconfirmed the significant increase of interaction between Clathrin and Rab5a in MM-ZC treated cells (**Figure 8B**). Similarly, we tested the interaction between Rab5a and Syntaxin-6 in MM-ZC treated cells and found that MM-ZC did not alter the association between the latter two proteins (compare lanes 5 and 6 in **Figure 8C** bottom panel, densitometric analysis in **Figure 8D**). Together, these results provide evidence that MM-ZC enhances the Clathrin-Rab5a complex formation and depends on the complex to traffic into the cells. Intrigued by our findings on the effect of MM-ZC on enhancing the interaction between Rab5a and Clathrin by immunoprecipitation analysis, we set out to explore whether this effect was observed *in vivo* by fluorescence imaging for Rab5a and Clathrin colocalization using confocal microscopy on MM1S cells treated with Ctr and MM-ZC sequences. As expected, strong colocalization (as indicated by the white areas that resulted from merging green and magenta dyes) of Rab5a and Clathrin was observed in MM-ZC treated MM1S cells (**Figure 8F**; **Supplementary Figures 8F-J**) where minimal association was detected in Ctr treated cells (**Figure 8E**; **Supplementary Figures 8A-E**) as evident by the segregated green and magenta areas. In contrast, colocalization of Rab5a and Syntaxin-6 failed to show strong enrichment in MM-ZC compared to Ctr-treated cells (compare **Figures 8G, H**). Both treatments demonstrated the similar colocalized spots of green and magenta fluorescence signals in MM1S cells. (compare **Supplementary Figure 8K-O, P-T**). These *in vivo* colocalization patterns between Rab5a-Clathrin and Rab5a-Syntaxin-6 were quantified in an unbiased manner. Images from quadruplicate experiments were acquired using a Leica SP8 LIGHTING confocal microscope. The 3D visualization and colocalization were performed using Bitplane Imaris 10. The co-localization of Rab5a with Clathrin and Syntaxin proteins was measured by Pearson correlation in the area of interest in both treatments, and results are plotted on a bar graph. As demonstrated in **Figure 8I**, MM-ZC treatments significantly increased the association between Rab5a and Clathrin compared to Ctr treatment. As expected, MM-ZC treatment plays a no or minor role in enhancing the association between Rab5a and Syntaxin-6. Therefore, these results reconfirm the earlier coimmunoprecipitation data and indicate that the novel protein-protein and protein-DNA associations facilitate MM-ZC internalization in myeloma cells.

## Conclusion

Previously we have demonstrated a significant discovery related to horizontal gene transfer (HGT) in the context of cancer, explicitly involving cell-free DNA circulating tumor DNA (ctDNA) as a vehicle for transferring genes between cancer cells. Our pioneering study revealed that retrotransposons, specific mobile elements within the ctDNA, play a central role in mediating HGT in cancer (1). Retrotransposons are sequences capable of moving within the genome, and their reactivation in cancer cells may facilitate their integration into the cancer cell genome. We showed that ctDNA-derived sequences could serve as a vehicle for transferring genetic material between cancer cells by using various reporters as a delivery cargo to target cancer cells (1). However, the specific cellular mechanism by which ctDNA is recognized and internalized by target cancer cells is unknown. Hence, the present investigation focuses on a mechanistic understanding of intracellular trafficking and how different pathways contribute to the cellular uptake of MM-ZC in myeloma cells. The study also aims to identify the proteins involved in MM-ZC uptake and to determine which endocytic routes contribute most to effective gene delivery based on ZIP code sequences. Understanding how cells take up and process MM-ZC delivery systems is crucial for enhancing their efficiency and specificity. Our results also highlight the careful design of experiments to optimize conditions and minimize potential confounding factors. Time-resolved experiments provided valuable kinetic information about the uptake process. Furthermore, attention was given to maintaining cell viability, as demonstrated by including viability tests. Myeloma cells uptake MM-ZC in a concentration-dependent and time-dependent manner. Comparative analysis of MM-ZC with other DNA sequences suggested that MM-ZC specifically internalizes certain types of myeloma cell lines. Various mechanisms for cellular uptake are known, and immunofluorescence analysis is used to assess the impact of these inhibitors on the internalization of MM-ZC. Our results confirmed that the uptake of MM-ZC mainly depends on Clathrin and endosomal maturation mechanisms. These results are in unison with the previous studies that suggested that Clathrin-mediated endocytosis is the primary mechanism of lipoplexes (liposomes carrying DNA) entry into cells (41). In addition, our findings are also partially similar to the endocytosis process observed for viruses. Different endocytic pathways mediate viral entry and utilize early endosomes marked by Rab5. Some viruses progress to late endosomes marked by Rab7 (42). Outward sorting virions are mediated by Rab11, which is part of the recycling endosome. Therefore, as MM-ZC is derived from retrotransposons, it’s tempting to speculate that MM-ZC might exploit the same pathway proteins, such as Rab5a that aid the virus to internalize inside the cells. However, we envisage that MM-ZC might also internalize through a non-Clathrin dependent pathway depending on the cell type. To further understand the process, we investigated MM-ZC’s interaction with membrane receptors, endosomal proteins, and organelle-associated trafficking proteins. We conclude that several proteins are associated with MM-ZC, including Clathrin, Rab5a, and Syntaxin-6. Loss of these proteins further reduced MM-ZC internalization in myeloma cells, highlighting their importance in the uptake process. Interestingly, we showed the interdependencies among Rab5a, Clathrin, and syntaxin-6 on MM-ZC internalization, suggesting that these proteins function in a coordinated and stepwise signaling pathway for MM-ZC trafficking. Our results are in accordance with the retrograde trafficking mechanism where internalizing particles trafficking from endosomes to the trans-Golgi network aim to divert away from lysosomal degradation (43). Next, we compared our observations with the previously reported DNA endocytosis mechanistic process. Rosazza et al., reported that the endocytosis and intracellular trafficking of electroporated DNA into CHO cells forms stable clusters at the cell membrane and internalizes through different mechanisms. Macropinocytosis, Clathrin- and Caveolin/raft-mediated endocytosis independently contribute 25%, 30%, and 50% of the internalization of the DNA, respectively (7). Furthermore, DNA aggregates were observed around early (Rab5a), recycling (Rab11), late endosomes (Rab9), and lysosomes. Finally, most DNA is lost in lysosomes, which can limit the overall gene expression efficiency achieved through DNA electrotransfer (7). Unlike the electroporated DNA endocytosis mechanism, MM-ZC internalizes predominantly through Clathrin and Rab5a molecules avoiding the recycling or lysosomal pathway protein association. These mechanistic differences bestow the specificity and stability of the MM-ZC and efficient internalization and cellular uptake. Though we showed the process of MM-ZC internalization in myeloma cells we still have to address how MM-ZC is associated with the cell membrane or internalized to the nucleus. As we know that MM-ZC is a DNA sequence, we speculate that specific DNA-binding factors might function as receptors at the membrane surface on the myeloma cells and bind to it and form the protein-nucleic acid complex, which might be engaging with the Clathrin-mediated vesicle formations and undergoing through the known endocytosis process. Therefore, future investigations are needed to define the binding receptors for MM-ZC and how those proteins specifically mediate the MM-ZC internalization process in myeloma cells but not in other cell types. Overall, the study provided valuable insights into the endocytic pathways involved in MM-ZC endocytosis and shed light on the specific mechanisms of MM-ZC uptake and intracellular trafficking in myeloma cells. The findings contribute to developing novel gene delivery systems and potential therapeutic applications.

## Data availability statement

The original contributions presented in the study are included in the article/**Supplementary Material**. Further inquiries can be directed to the corresponding authors.

## Ethics statement

Ethical approval was not required for the studies on animals in accordance with the local legislation and institutional requirements because only commercially available established cell lines were used.

## Author contributions

PP: Conceptualization, Methodology, Project administration, Supervision, Validation, Writing – original draft, Writing – review & editing. LM-M: Data curation, Methodology, Writing – review & editing. MC: Data curation, Writing – review & editing. LF: Data curation, Writing – review & editing. AP: Data curation, Writing – review & editing. AJ: Funding acquisition, Writing – review & editing. LB-M: Conceptualization, Data curation, Writing – review & editing.

## Supplementary material

Supplementary Figure 1 Effect of MM-ZC on cell viability and IFN gamma expression in MM1S cells. **(A)** CellTiter-blue Cell viability assay of MM1S cells treated with Ctr and MM-ZC sequences at the concentration of 1 μg per ml for 0 and 24 hr. Error bars represent the standard deviation from 3 replicates. No significant difference was observed between the Ctr and MM-ZC after 24 hr of treatment. Unpaired T-test is used to determine the significant difference. **(B)** qRT-PCR analysis of IFNγgene expression in total RNA isolated after 24 hr of Ctr and MM-ZC treatment. **(C, D)** Representative microscopic images (selected from ten random different field of view) of MM1S **(C)** and JK6L **(D)** cells treated with Rhodamine-labeled MM-ZC for 8 hr. Top panel represents no treatment, and the bottom panel represents the DNase1 treatment. MM-ZC is represented by Rhodamine red channel (left side panel) Cells were visualized by bright field (right side panel). Scale bar, 150 μm. **(E)** Dose titration of MM-ZC internalization in JK6L cells by FACS analysis. JK6L cells were treated with four different concentrations (as mentioned on X-axis) of Cy5 labeled MM-ZC DNA for 24 hr. The percentage of Cy5 positive cells is plotted on Y-axis. * Indicates p<0.05 relative to the lesser concentration. Sample size for each dose point is at least 10K. Error bars represent the standard error of mean from 3 replicates.

Supplementary Figure 2 Effect of different concentrations of the inhibitors on the cellular viability of MM1s cells **(A)** Quantification of percent cell viability of MM1S cells after 8 hr of incubation with different inhibitors. Two concentrations of each inhibitor were tested, as mentioned on the X-axis. **(B)** The lowest concentration of each inhibitor from **(A)** has been tested further for a shorter period of treatment. Quantification of percent cell viability of MM1s cells after 4 hr of incubation in different inhibitors was plotted on Y-Axis; X-Axis indicates the various inhibitors. Sample size is one million cells for each treatment. Error bars represent the standard deviation from 3 replicates.

Supplementary Figure 3.1
**(A-D)** Representative bright field images corresponding to the immunofluorescence images of . Scale bar, 150 μm. Images were taken by Invitrogen EVOS M5000 microscope.

Supplementary Figure 3.2
**(A-H)** Representative immunofluorescence (top panel) and bright field images (bottom panel) of MM1S cells after 2 hr of inhibitors treatment followed by an additional 2 hr of treatment with Rhodamine-labeled MM-ZC. Inhibitors are mentioned on top of the panels. Scale bar for all the images is 150 μm. Images were taken by Invitrogen EVOS M5000 microscope.

Supplementary Figure 5.1 Colocalization of Rab5a with MM-ZC but not with Ctr, vector sequence or PC1 DNAs in JK6L cells. **(A-D)** Representative Z-stacked immunofluorescence images of anti-Rab5a (green) in JK6L cells treated with Rhodamine-labeled MM-ZC, Ctr, Vector and Pc1 DNA (red) for 8 hr. Rab5a showed colocalization with MM-ZC but not with other DNAs. Images are captured by using Zeiss 700 confocal microscopy Scale bars for all the images = 10 μm. Arrows indicate the colocalized signal between MM-ZC and Rab5a **(E, F)**. Pulldown analysis of biotin-labeled control (Ctr), Vector, PC1, PC3 and MM-ZC followed by immunoblotting to examine the association of Rab5a **(E)** and Rab7 **(F)** with different DNA sequences. Lanes 1-6 indicate Input samples and lanes 7-12 show pulldown samples. Compare lanes 7-11 and 12 to see the enrichment of Rab5a in MM-ZC pulldown samples.

Supplementary Figure 5.2 Colocalization of MM-ZC with various endosomal markers and other membrane-associated factors in JK6L cells. **(A-D)** Zoom in images of to show the colocalization of MM-ZC with Clathrin, Rab5a, Syntaxin-6 and RCAS1 proteins in JK6L cells. **(E–H)** Video demonstrating the 3D projection of Z-stack panels corresponding to images. Arrows indicate the yellow colored colocalized areas of MM-ZC (red) with endogenous proteins (green).

Supplementary Figure 6 Rab5a and Clathrin knockdown reduce MM-ZC internalization in MM1S cells. **(A)** Representative immunofluorescence images of anti-CD98 (green) in Rab5a, Clathrin and Rab5a + Clathrin knockdown MM1S cells treated with Rhodamine-labeled MM-ZC for 8 hr. CD98 is used as membrane marker to visualize the cell surface. Compare the red signals between **(A, B)** or **(C, D)**. Red fluorescence signals were reduced in MM1S cells transfected with RAB5A or Clathrin or Rab5a + Clathrin siRNAs. Scale bar: 10 μm.

Supplementary Figure 7 Interdependency among Rab5a, Clathrin, and Syntaxin-6 in MM-ZC association in MM1S cells. **(A, C, E)** Representative immunofluorescence images (selected from ten different randomly captured field of views) of MM-ZC (red) in control cells stained with anti-Rab5a (Green in **A**), Clathrin (Green in **C**) and Syntaxin-6 (Green in **E**). Similarly, **B, D, F** show the staining of Rab5a in Clathrin knockdown cells **(B)** Clathrin in Rab5a knockdown cells **(D)** and Syntaxin-6 in Rab5a knockdown cells. Scale bar: 10 μm. Rab5a is probed with anti-Rab5a and Alexa Fluor 488-conjugated secondary mouse IgG, whereas Clathrin and Syntaxin-6 are probed with their respective individual specific primary antibodies followed by Alexa Fluor 488 -conjugated secondary rabbit IgG. MM-ZC is labeled with Rhodamine fluorescent dye. Images are captured by using Zeiss 700 confocal microscopy.

Supplementary Figure 8 Representative immunofluorescence images (acquired by Zeiss 700 confocal microscope) (selected from 5 different randomly captured field of views) of Rab5 colocalization with Clathrin **(A, J)** and Syntaxin-6 **(K, T)** in MM1S cells treated with Ctr **(E, O)** and MM-ZC **(F, P)** for 8 hr. Individual channels (Rab5a = Cy5, Magenta, Clathrin/Syntaxin-6 = Alexa fluor 488, Green). **A-D** are the individual channels corresponding to **(E)**; **(G-J)** are for **(F)**; **(K-M)** are for **(O)**, and **(Q-T)** are for panel P. Scale bar for all the images: 10μm. Individual channels (Rab5a = Cy5, Magenta, Clathrin/Syntaxin-6 = Alexa fluor 488, Green, Ctr or MM-ZC = Rhodamine Red, Nuclei = Hoechst, Blue). Images are captured by using Zeiss 700 confocal microscopy.

Supplementary Figure 9 All the original immunoblots are provided with their specific figure numbers.

Supplementary Figure 10 Coimmunoprecipitations from **Figures 7**, **8** are performed in three replicates. Images from one replicate have been incorporated in the main figures. The remaining two replicates are provided here as **Supplementary Data**. All the three replicates are utilized for the densitometric analysis in **Figures 7**, **8**.
