# Supplementary figures and images for: A retrotransposon-derived DNA zip code internalizes myeloma cells through Clathrin-Rab5a-mediated endocytosis
Source: Front Oncol. 2024 Feb 23;14:1288724. doi: 10.3389/fonc.2024.1288724 (PMC10920344; doi:10.3389/fonc.2024.1288724)

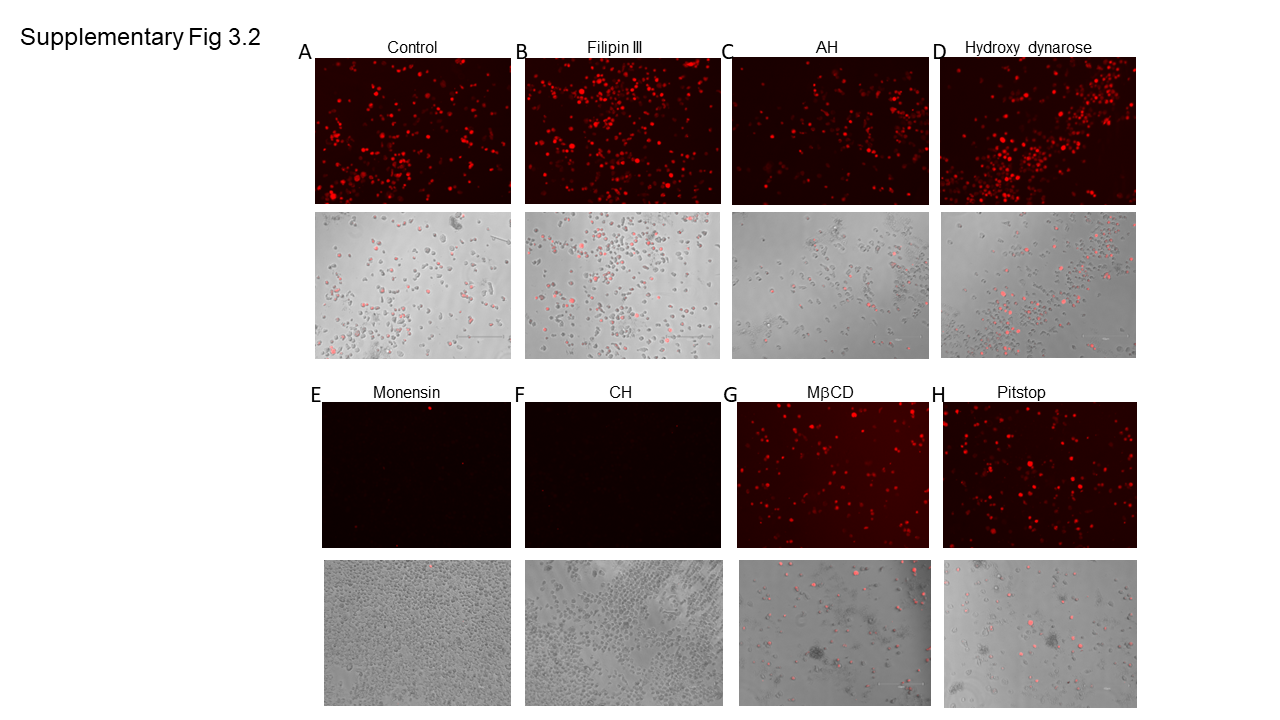

Supplement: Supplementary Figure 1 — Effect of MM-ZC on cell viability and IFN gamma expression in MM1S cells. (A) CellTiter-blue Cell viability assay of MM1S cells treated with Ctr and MM-ZC sequences at the concentration of 1 μg per ml for 0 and 24 hr. Error bars represent the standard deviation from 3 replicates. No significant difference was observed between the Ctr and MM-ZC after 24 hr of treatment. Unpaired T-test is used to determine the significant difference. (B) qRT-PCR analysis of IFNγgene expression in total RNA isolated after 24 hr of Ctr and MM-ZC treatment. (C, D) Representative microscopic images (selected from ten random different field of view) of MM1S (C) and JK6L (D) cells treated with Rhodamine-labeled MM-ZC for 8 hr. Top panel represents no treatment, and the bottom panel represents the DNase1 treatment. MM-ZC is represented by Rhodamine red channel (left side panel) Cells were visualized by bright field (right side panel). Scale bar, 150 μm. (E) Dose titration of MM-ZC internalization in JK6L cells by FACS analysis. JK6L cells were treated with four different concentrations (as mentioned on X-axis) of Cy5 labeled MM-ZC DNA for 24 hr. The percentage of Cy5 positive cells is plotted on Y-axis. * Indicates p<0.05 relative to the lesser concentration. Sample size for each dose point is at least 10K. Error bars represent the standard error of mean from 3 replicates. [file Image_1.tif]

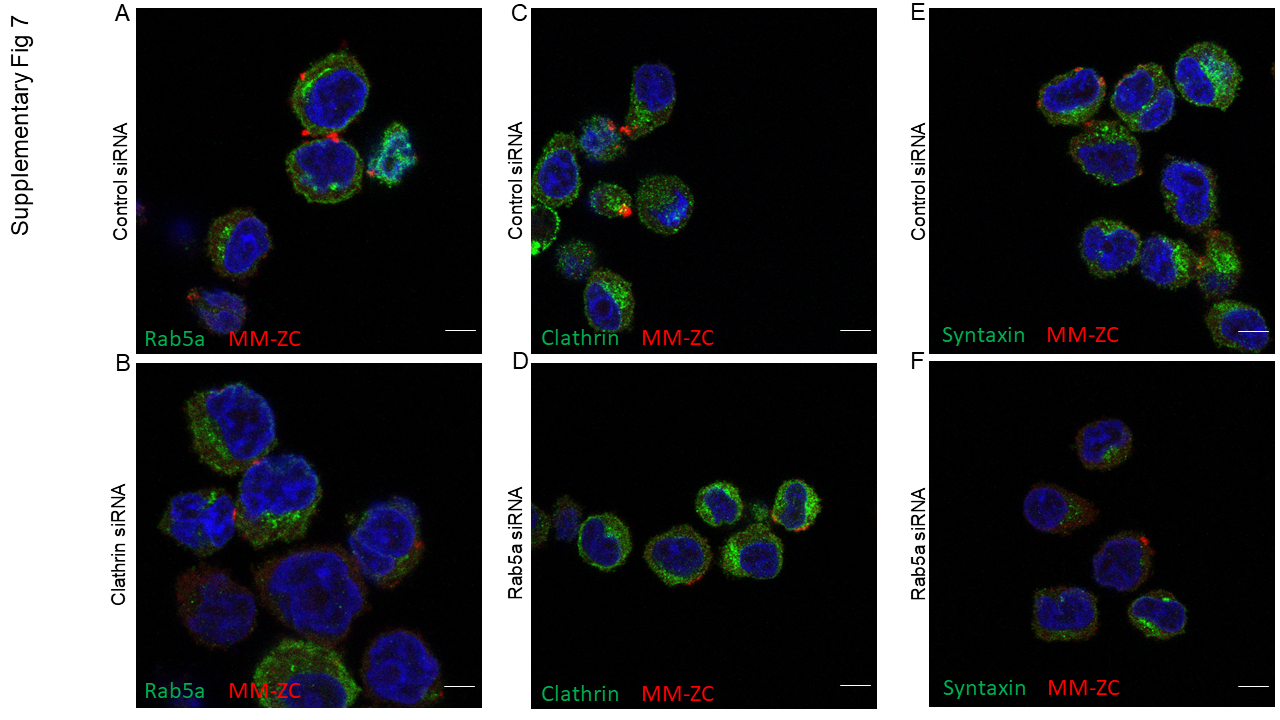

Supplement: Supplementary Figure 2 — Effect of different concentrations of the inhibitors on the cellular viability of MM1s cells (A) Quantification of percent cell viability of MM1S cells after 8 hr of incubation with different inhibitors. Two concentrations of each inhibitor were tested, as mentioned on the X-axis. (B) The lowest concentration of each inhibitor from (A) has been tested further for a shorter period of treatment. Quantification of percent cell viability of MM1s cells after 4 hr of incubation in different inhibitors was plotted on Y-Axis; X-Axis indicates the various inhibitors. Sample size is one million cells for each treatment. Error bars represent the standard deviation from 3 replicates. [file Image_2.tif]

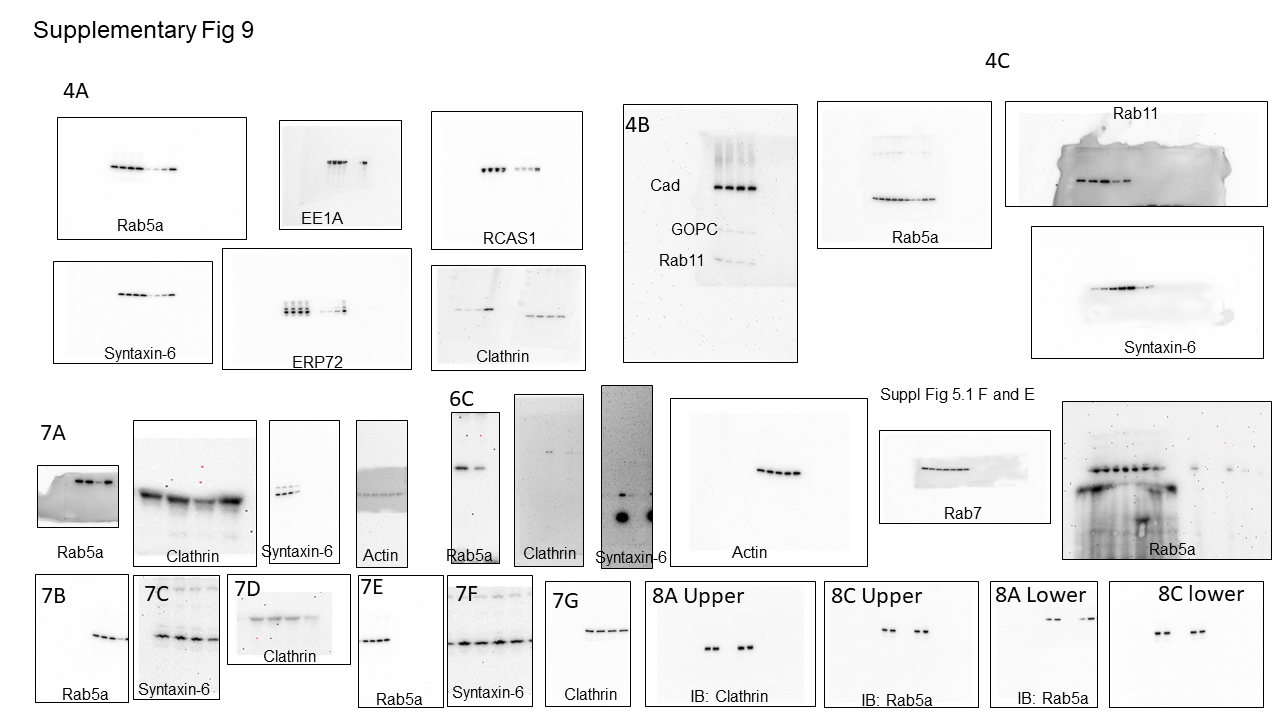

Supplement: Supplementary Figure 3.1 — (A-D) Representative bright field images corresponding to the immunofluorescence images of . Scale bar, 150 μm. Images were taken by Invitrogen EVOS M5000 microscope. [file Image_3.tif]

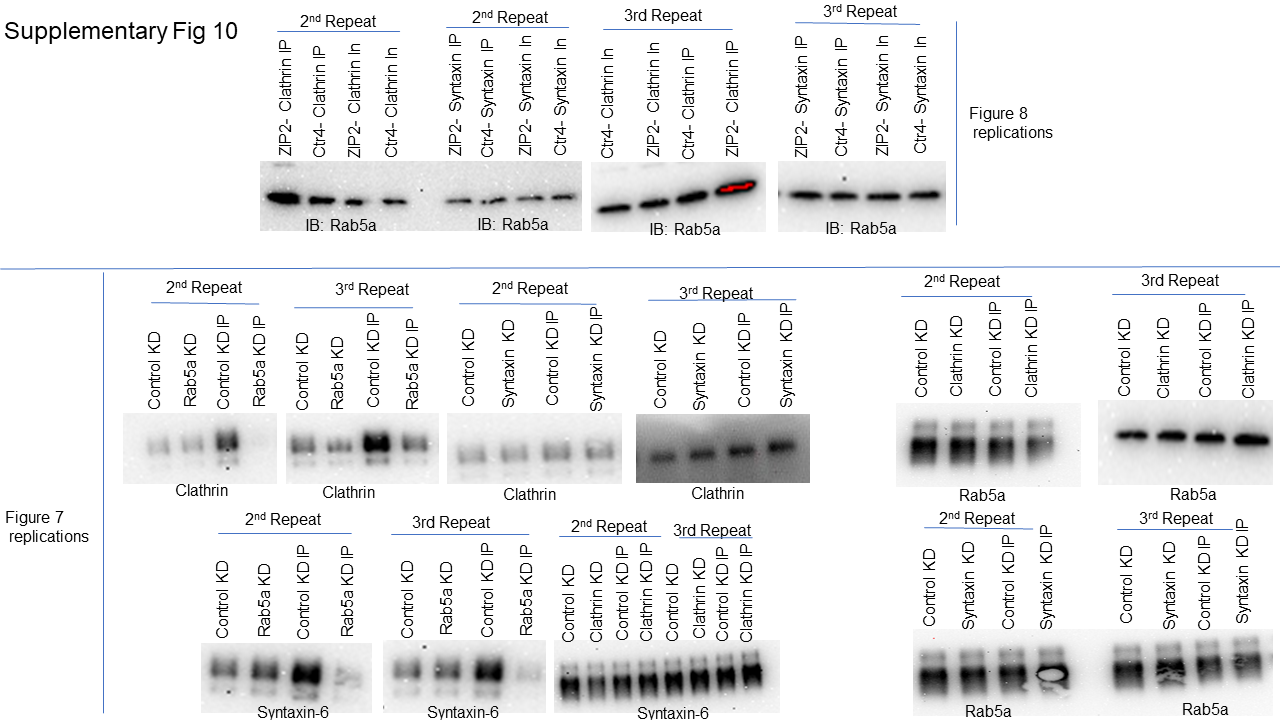

Supplement: Supplementary Figure 3.2 — (A-H) Representative immunofluorescence (top panel) and bright field images (bottom panel) of MM1S cells after 2 hr of inhibitors treatment followed by an additional 2 hr of treatment with Rhodamine-labeled MM-ZC. Inhibitors are mentioned on top of the panels. Scale bar for all the images is 150 μm. Images were taken by Invitrogen EVOS M5000 microscope. [file Image_4.tif]

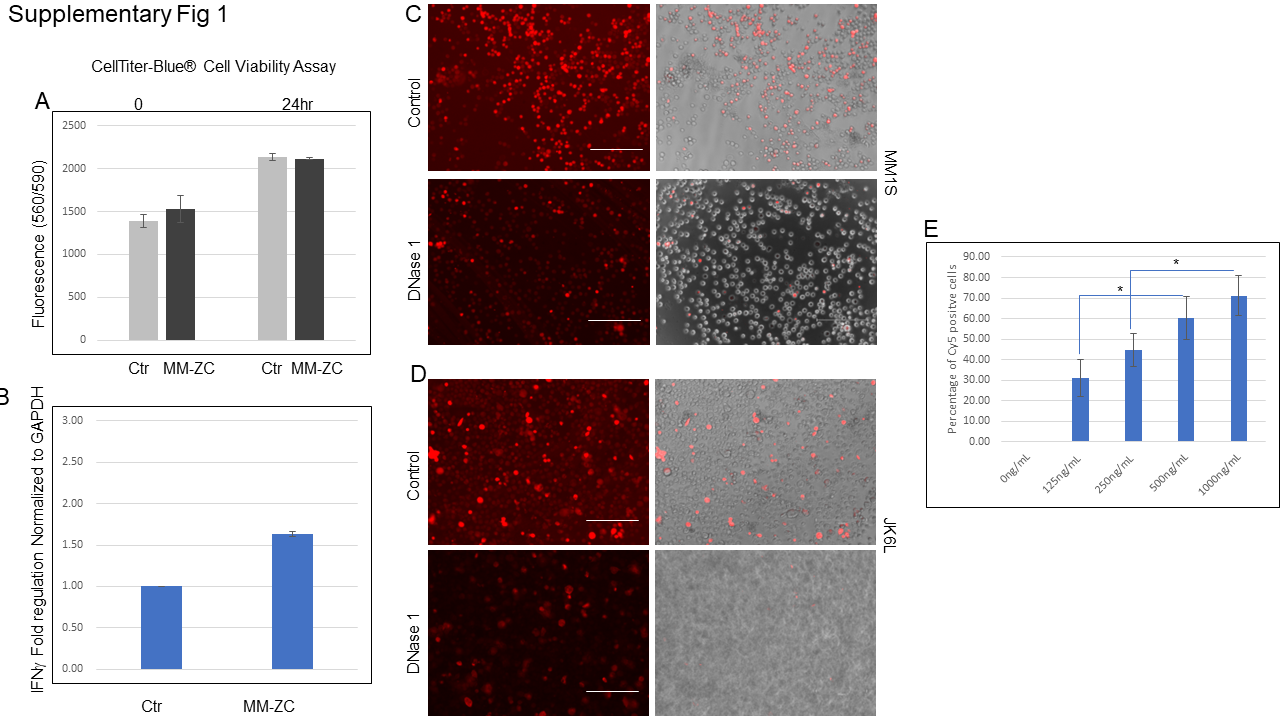

Supplement: Supplementary Figure 5.1 — Colocalization of Rab5a with MM-ZC but not with Ctr, vector sequence or PC1 DNAs in JK6L cells. (A-D) Representative Z-stacked immunofluorescence images of anti-Rab5a (green) in JK6L cells treated with Rhodamine-labeled MM-ZC, Ctr, Vector and Pc1 DNA (red) for 8 hr. Rab5a showed colocalization with MM-ZC but not with other DNAs. Images are captured by using Zeiss 700 confocal microscopy Scale bars for all the images = 10 μm. Arrows indicate the colocalized signal between MM-ZC and Rab5a (E, F). Pulldown analysis of biotin-labeled control (Ctr), Vector, PC1, PC3 and MM-ZC followed by immunoblotting to examine the association of Rab5a (E) and Rab7 (F) with different DNA sequences. Lanes 1-6 indicate Input samples and lanes 7-12 show pulldown samples. Compare lanes 7-11 and 12 to see the enrichment of Rab5a in MM-ZC pulldown samples. [file Image_5.tif]

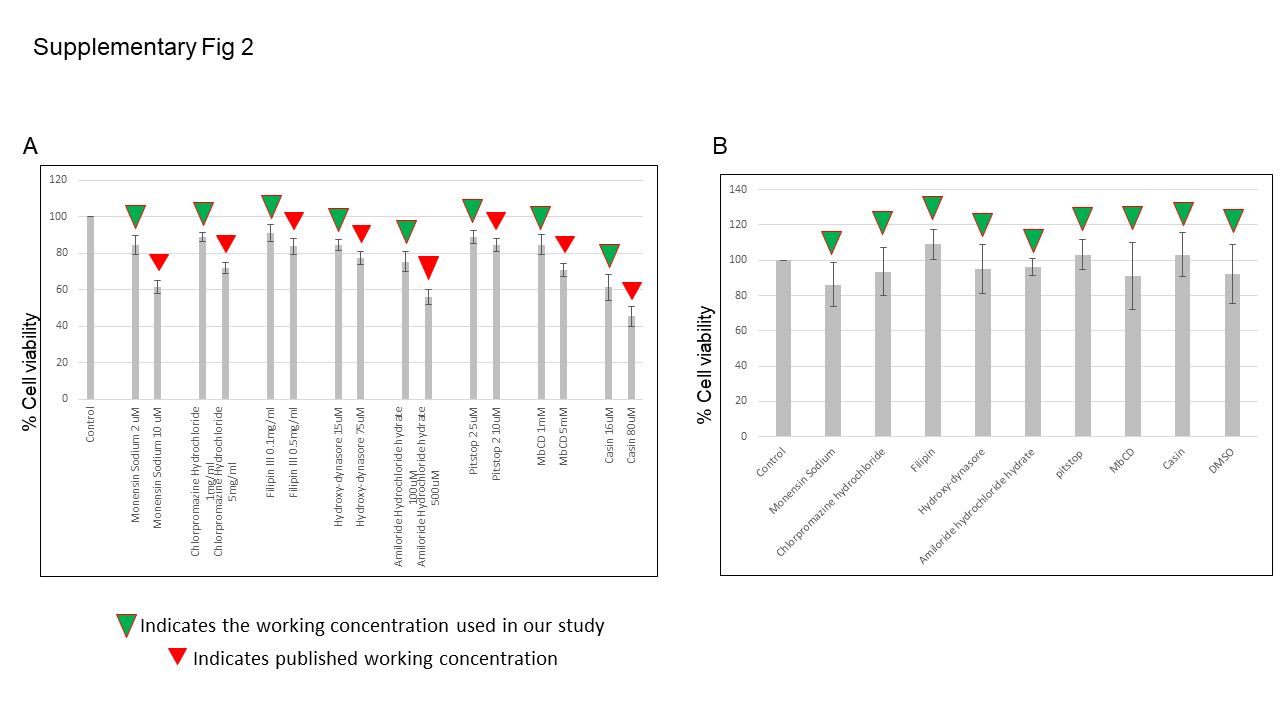

Supplement: Supplementary Figure 5.2 — Colocalization of MM-ZC with various endosomal markers and other membrane-associated factors in JK6L cells. (A-D) Zoom in images of to show the colocalization of MM-ZC with Clathrin, Rab5a, Syntaxin-6 and RCAS1 proteins in JK6L cells. (E–H) Video demonstrating the 3D projection of Z-stack panels corresponding to images. Arrows indicate the yellow colored colocalized areas of MM-ZC (red) with endogenous proteins (green). [file Image_6.tif]

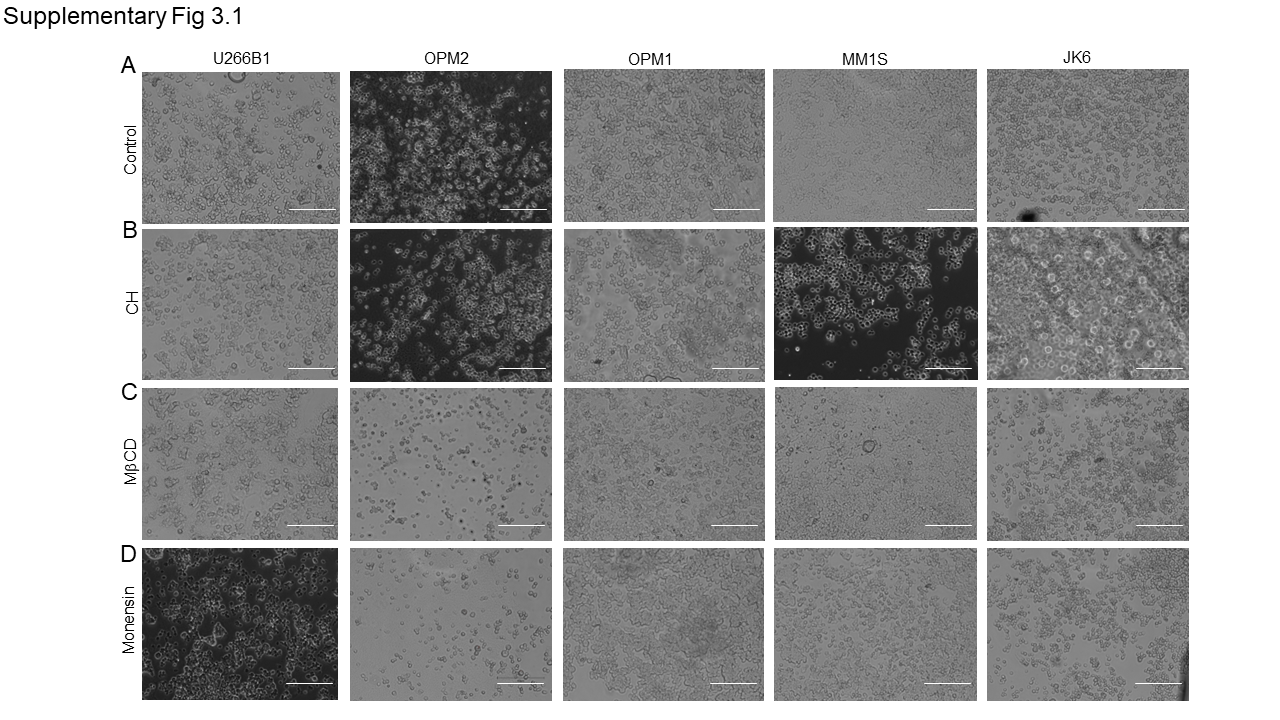

Supplement: Supplementary Figure 6 — Rab5a and Clathrin knockdown reduce MM-ZC internalization in MM1S cells. (A) Representative immunofluorescence images of anti-CD98 (green) in Rab5a, Clathrin and Rab5a + Clathrin knockdown MM1S cells treated with Rhodamine-labeled MM-ZC for 8 hr. CD98 is used as membrane marker to visualize the cell surface. Compare the red signals between (A, B) or (C, D). Red fluorescence signals were reduced in MM1S cells transfected with RAB5A or Clathrin or Rab5a + Clathrin siRNAs. Scale bar: 10 μm. [file Image_7.tif]

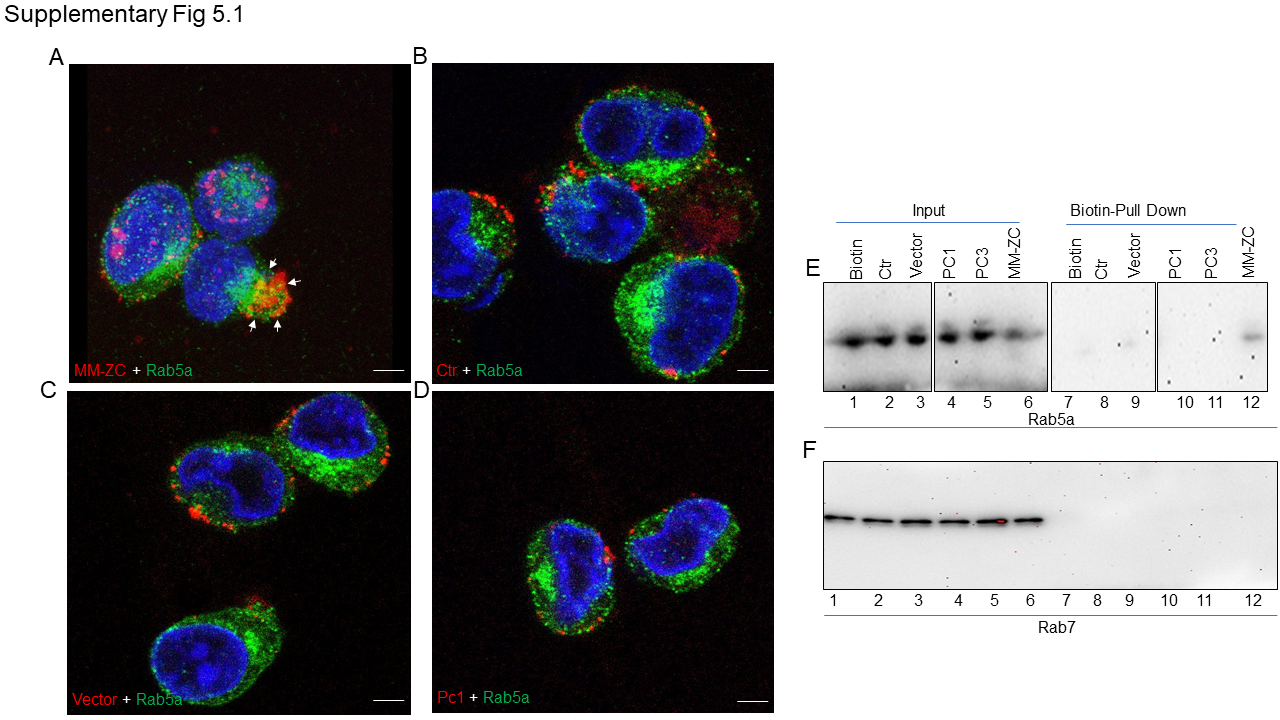

Supplement: Supplementary Figure 7 — Interdependency among Rab5a, Clathrin, and Syntaxin-6 in MM-ZC association in MM1S cells. (A, C, E) Representative immunofluorescence images (selected from ten different randomly captured field of views) of MM-ZC (red) in control cells stained with anti-Rab5a (Green in A), Clathrin (Green in C) and Syntaxin-6 (Green in E). Similarly, B, D, F show the staining of Rab5a in Clathrin knockdown cells (B) Clathrin in Rab5a knockdown cells (D) and Syntaxin-6 in Rab5a knockdown cells. Scale bar: 10 μm. Rab5a is probed with anti-Rab5a and Alexa Fluor 488-conjugated secondary mouse IgG, whereas Clathrin and Syntaxin-6 are probed with their respective individual specific primary antibodies followed by Alexa Fluor 488 -conjugated secondary rabbit IgG. MM-ZC is labeled with Rhodamine fluorescent dye. Images are captured by using Zeiss 700 confocal microscopy. [file Image_8.tif]

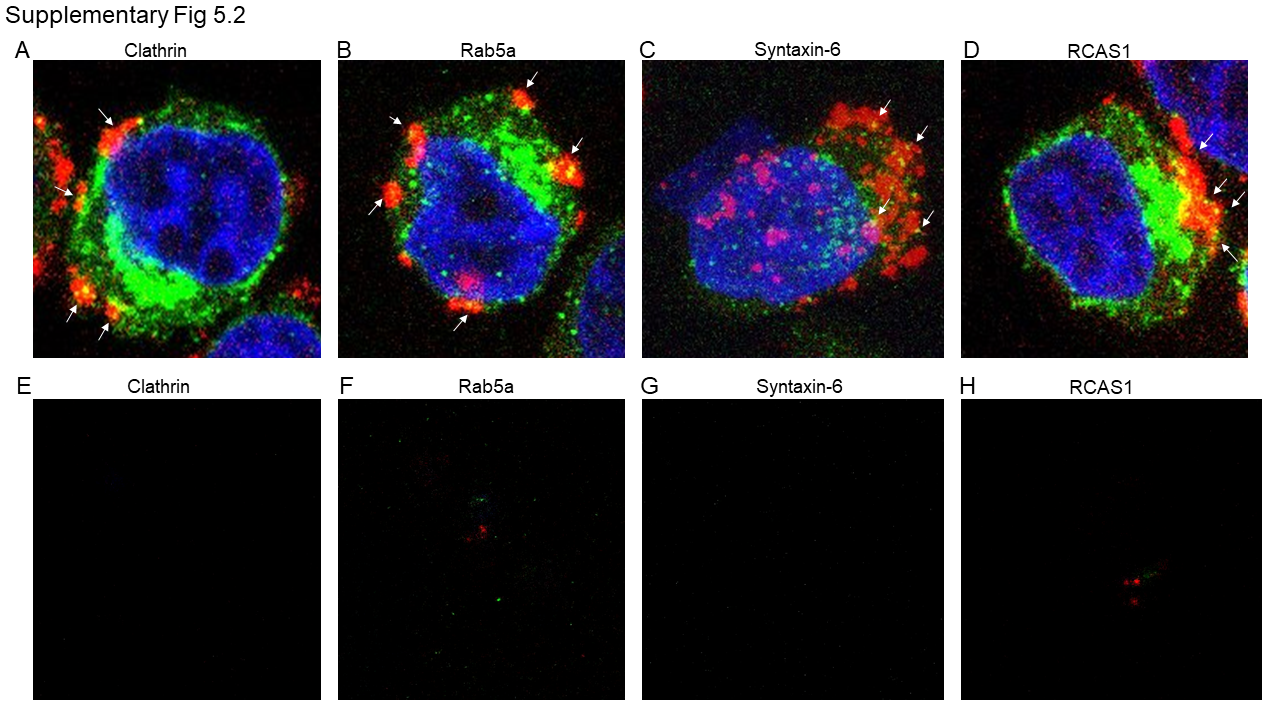

Supplement: Supplementary Figure 8 — Representative immunofluorescence images (acquired by Zeiss 700 confocal microscope) (selected from 5 different randomly captured field of views) of Rab5 colocalization with Clathrin (A, J) and Syntaxin-6 (K, T) in MM1S cells treated with Ctr (E, O) and MM-ZC (F, P) for 8 hr. Individual channels (Rab5a = Cy5, Magenta, Clathrin/Syntaxin-6 = Alexa fluor 488, Green). A-D are the individual channels corresponding to (E); (G-J) are for (F); (K-M) are for (O), and (Q-T) are for panel P. Scale bar for all the images: 10μm. Individual channels (Rab5a = Cy5, Magenta, Clathrin/Syntaxin-6 = Alexa fluor 488, Green, Ctr or MM-ZC = Rhodamine Red, Nuclei = Hoechst, Blue). Images are captured by using Zeiss 700 confocal microscopy. [file Image_9.tif]

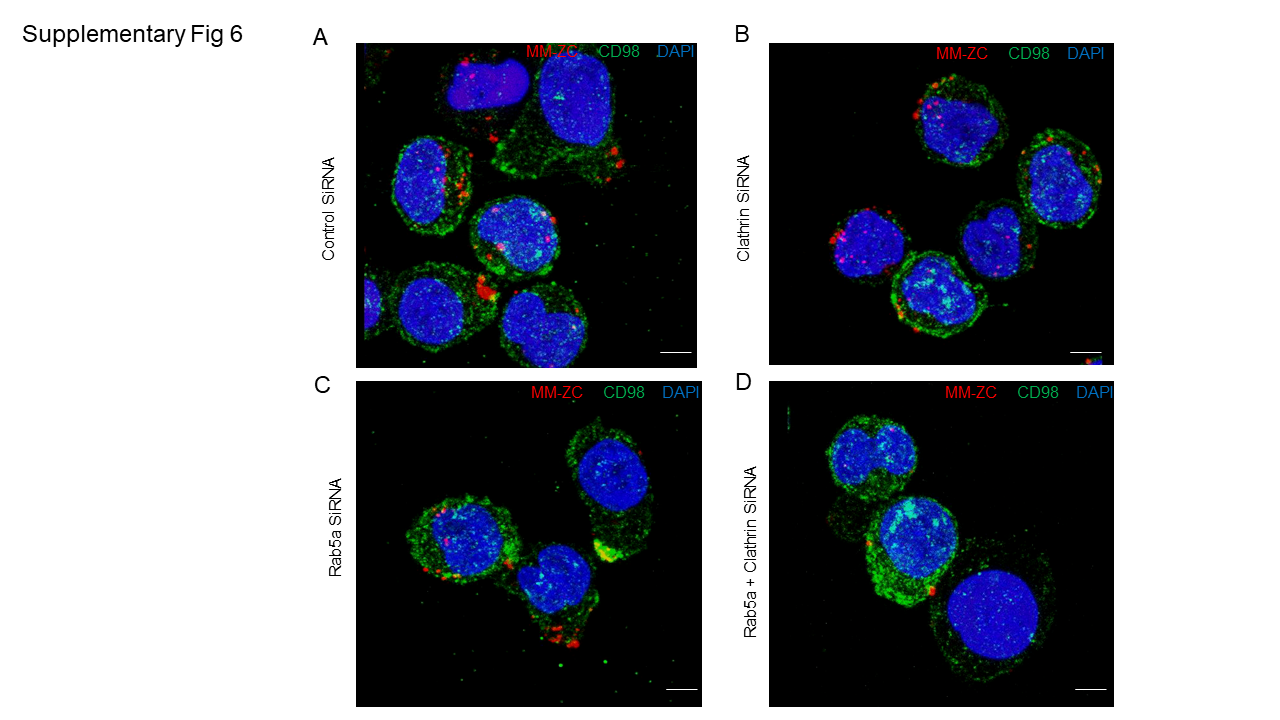

Supplement: Supplementary Figure 9 — All the original immunoblots are provided with their specific figure numbers. [file Image_10.tif]

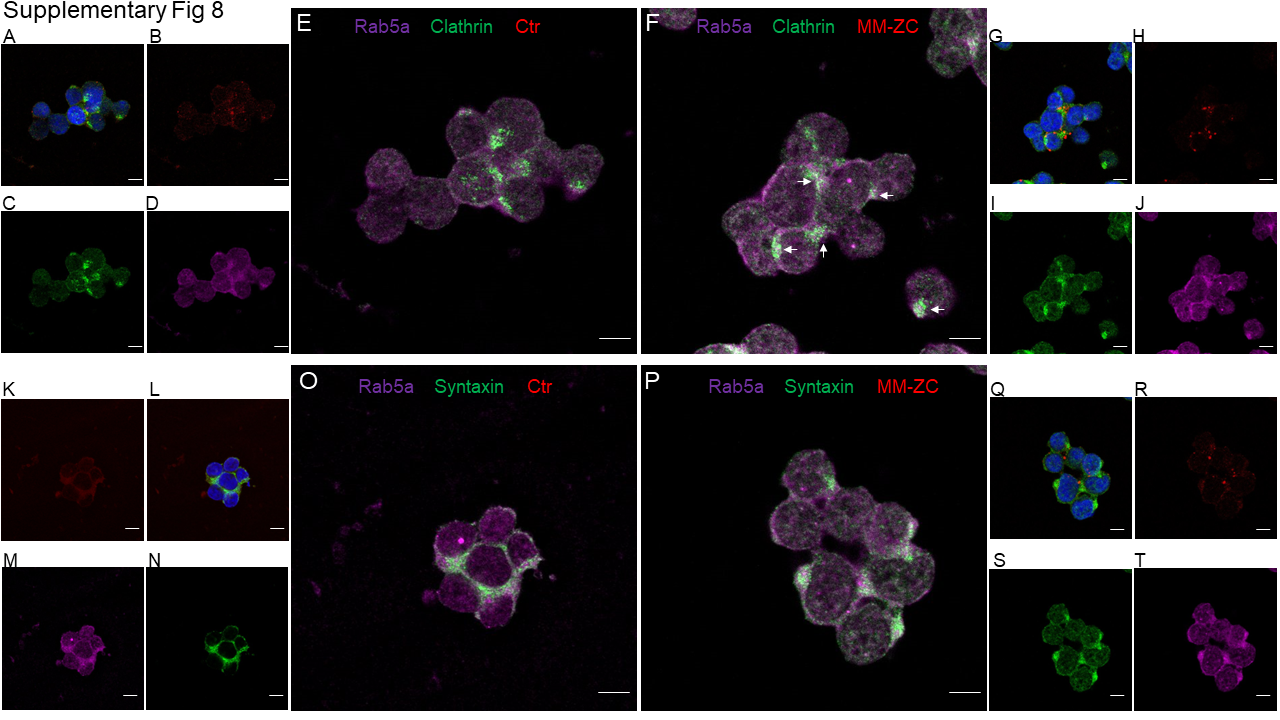

Supplement: Supplementary Figure 10 — Coimmunoprecipitations from Figures 7, 8 are performed in three replicates. Images from one replicate have been incorporated in the main figures. The remaining two replicates are provided here as Supplementary Data . All the three replicates are utilized for the densitometric analysis in Figures 7, 8. [file Image_11.tif]
