# Supplementary material for: A retrotransposon-derived DNA zip code internalizes myeloma cells through Clathrin-Rab5a-mediated endocytosis
Source: Front Oncol. 2024 Feb 23;14:1288724. doi: 10.3389/fonc.2024.1288724 (PMC10920344; doi:10.3389/fonc.2024.1288724)
Supplement: Supplementary file 12 [file DataSheet_1.zip › Raw data/Fig 1A, MM1S Dose titration/20220511 MM1S Dose titration MMZC Cy5 4 hrs.pdf]

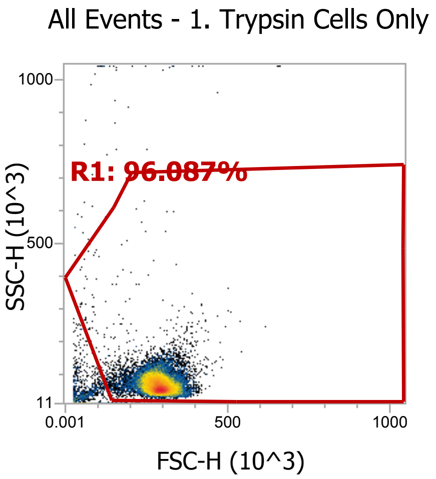

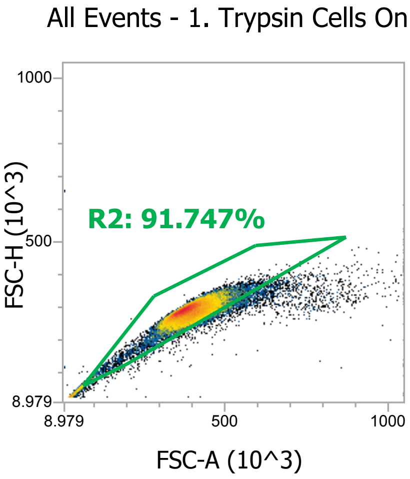

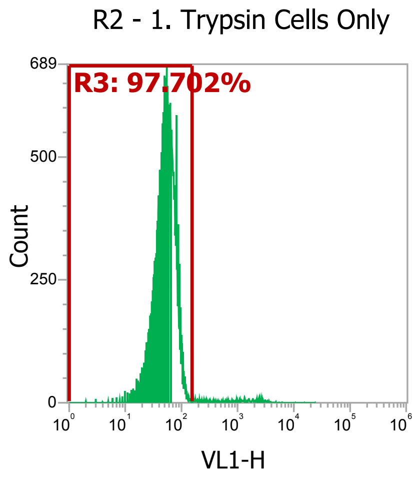

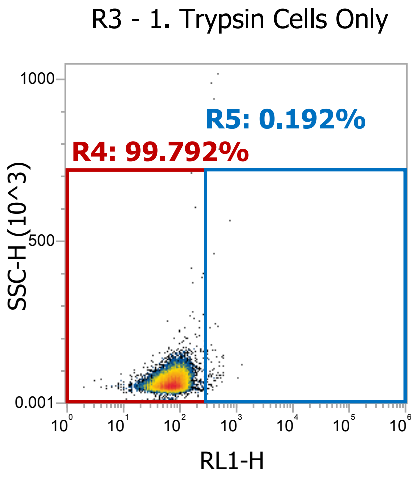

Experiment: **20220511**
Sample: **1. Trypsin Cells Only**
Time Recorded: **14:44:16**

Name	Gate	X Parameter	Y Parameter	Count	%Total	%Gated
All Events	All Events	N/A	N/A	34,907	100.000	100.000
R1	R1	FSC - FSC-H	SSC - SSC-H	33,541	96.087	96.087
R2	R2	FSC - FSC-A	FSC - FSC-H	32,026	91.747	91.747
R3	R3	VL1 - VL1-H		31,290	89.638	97.702
R4	R4	RL1 - RL1-H	SSC - SSC-H	31,225	89.452	99.792
R5	R5	RL1 - RL1-H	SSC - SSC-H	60	0.172	0.192

All Events - 2. Trypsin Cells+ 10ng per...

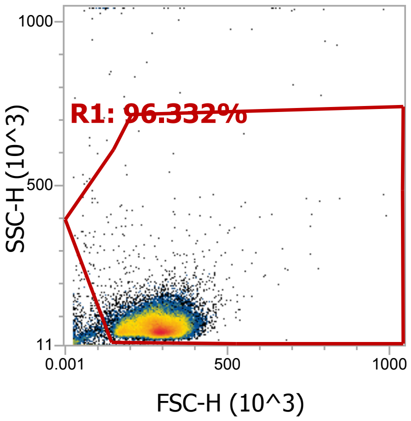

All Events - 2. Trypsin Cells+ 10ng per...

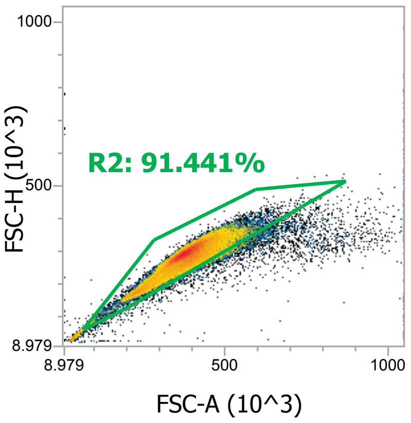

R2 - 2. Trypsin Cells+ 10ng per mL SEQ...

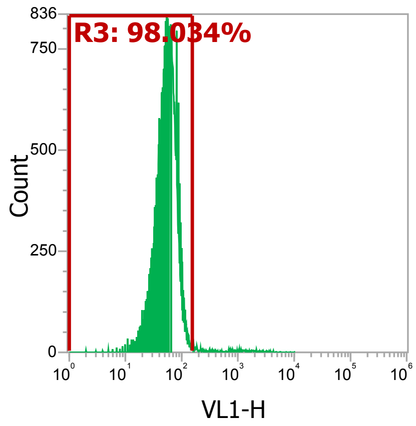

R3 - 2. Trypsin Cells+ 10ng per mL SEQ...

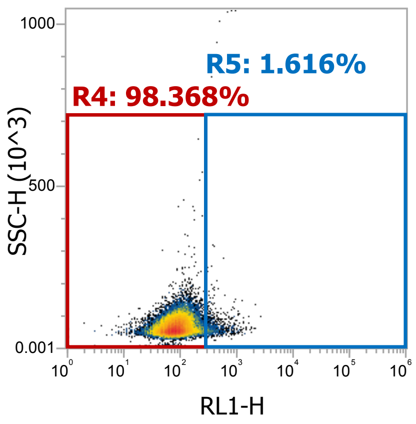

Experiment: **20220511**
Sample: **2. Trypsin Cells+ 10ng per mL SEQ2 Cy5**
Time Recorded: **14:45:45**

Name	Gate	X Parameter	Y Parameter	Count	%Total	%Gated
All Events	All Events	N/A	N/A	46,946	100.000	100.000
R1	R1	FSC - FSC-H	SSC - SSC-H	45,224	96.332	96.332
R2	R2	FSC - FSC-A	FSC - FSC-H	42,928	91.441	91.441
R3	R3	VL1 - VL1-H		42,084	89.643	98.034
R4	R4	RL1 - RL1-H	SSC - SSC-H	41,397	88.180	98.368
R5	R5	RL1 - RL1-H	SSC - SSC-H	680	1.448	1.616

All Events - 3. Trypsin Cells+ 30ng per...

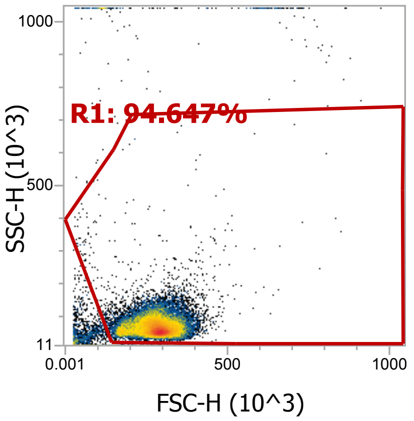

All Events - 3. Trypsin Cells+ 30ng per...

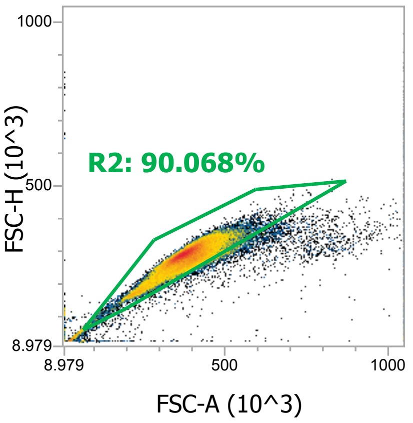

R2 - 3. Trypsin Cells+ 30ng per mL SEQ...

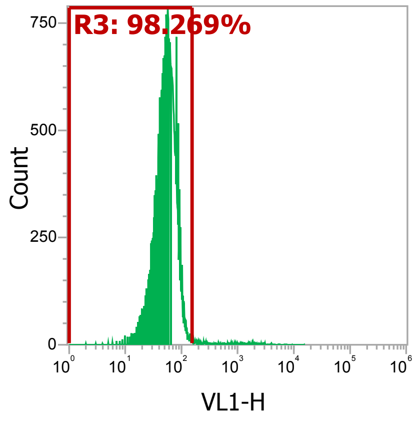

R3 - 3. Trypsin Cells+ 30ng per mL SEQ...

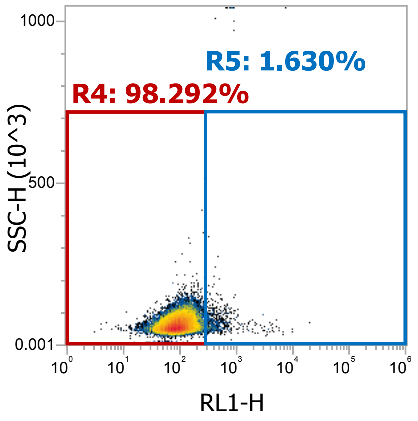

Experiment: **20220511**
Sample: **3. Trypsin Cells+ 30ng per mL SEQ2 Cy5**
Time Recorded: **14:47:14**

Name	Gate	X Parameter	Y Parameter	Count	%Total	%Gated
All Events	All Events	N/A	N/A	42,409	100.000	100.000
R1	R1	FSC - FSC-H	SSC - SSC-H	40,139	94.647	94.647
R2	R2	FSC - FSC-A	FSC - FSC-H	38,197	90.068	90.068
R3	R3	VL1 - VL1-H		37,536	88.510	98.269
R4	R4	RL1 - RL1-H	SSC - SSC-H	36,895	86.998	98.292
R5	R5	RL1 - RL1-H	SSC - SSC-H	612	1.443	1.630

All Events - 4. Trypsin Cells+ 100ng per...

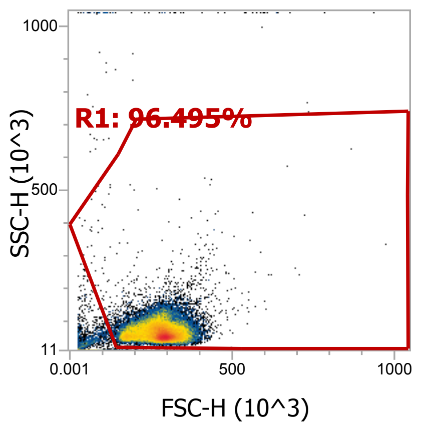

All Events - 4. Trypsin Cells+ 100ng per...

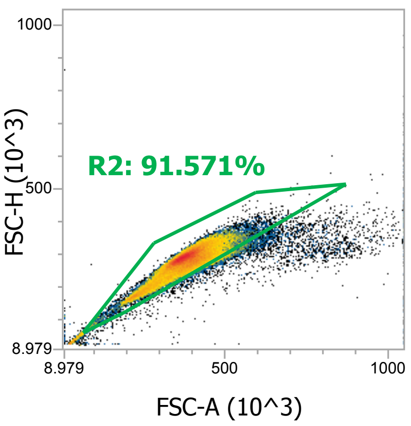

R2 - 4. Trypsin Cells+ 100ng per mL SE...

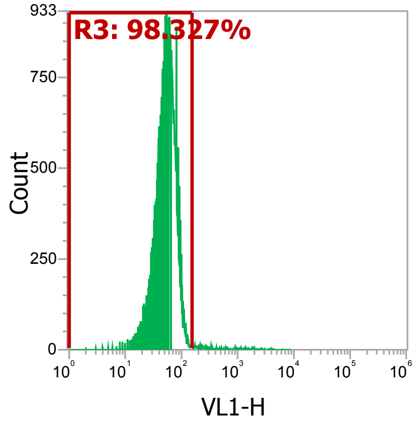

R3 - 4. Trypsin Cells+ 100ng per mL SE...

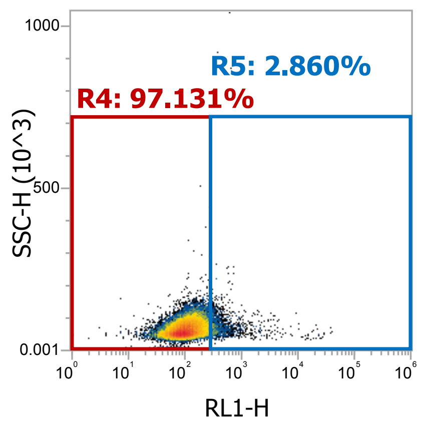

Experiment: **20220511**
Sample: **4. Trypsin Cells+ 100ng per mL SEQ2 Cy5**
Time Recorded: **14:48:42**

Name	Gate	X Parameter	Y Parameter	Count	%Total	%Gated
All Events	All Events	N/A	N/A	49,933	100.000	100.000
R1	R1	FSC - FSC-H	SSC - SSC-H	48,183	96.495	96.495
R2	R2	FSC - FSC-A	FSC - FSC-H	45,724	91.571	91.571
R3	R3	VL1 - VL1-H		44,959	90.039	98.327
R4	R4	RL1 - RL1-H	SSC - SSC-H	43,669	87.455	97.131
R5	R5	RL1 - RL1-H	SSC - SSC-H	1,286	2.575	2.860

All Events - 5. Trypsin Cells+ 300ng per…

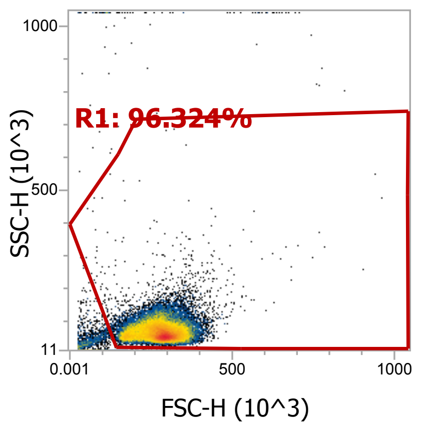

All Events - 5. Trypsin Cells+ 300ng per…

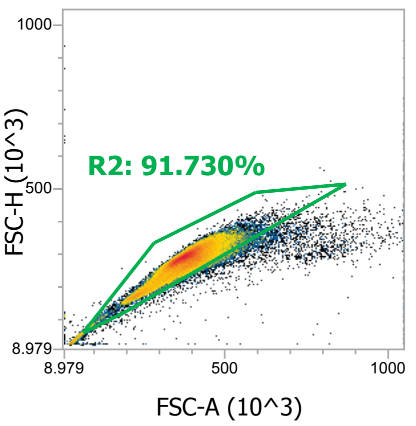

R2 - 5. Trypsin Cells+ 300ng per mL SE…

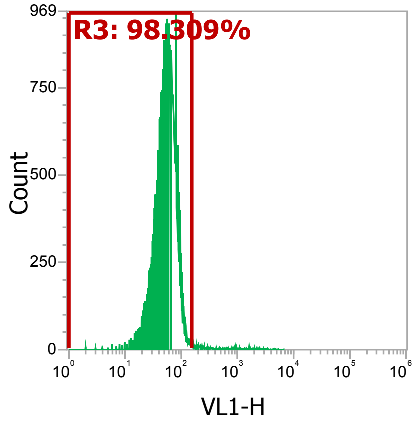

R3 - 5. Trypsin Cells+ 300ng per mL SE…

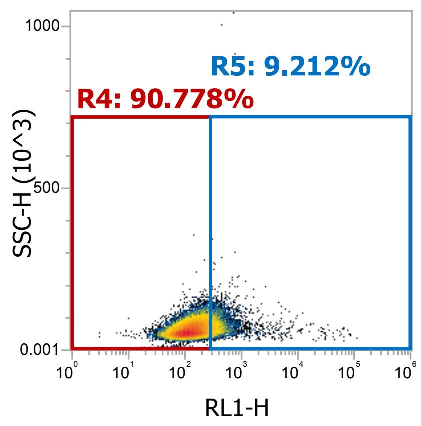

Experiment: **20220511**
Sample: **5. Trypsin Cells+ 300ng per mL SEQ2 Cy5**
Time Recorded: **14:50:11**

Name	Gate	X Parameter	Y Parameter	Count	%Total	%Gated
All Events	All Events	N/A	N/A	51,499	100.000	100.000
R1	R1	FSC - FSC-H	SSC - SSC-H	49,606	96.324	96.324
R2	R2	FSC - FSC-A	FSC - FSC-H	47,240	91.730	91.730
R3	R3	VL1 - VL1-H		46,441	90.178	98.309
R4	R4	RL1 - RL1-H	SSC - SSC-H	42,158	81.862	90.778
R5	R5	RL1 - RL1-H	SSC - SSC-H	4,278	8.307	9.212

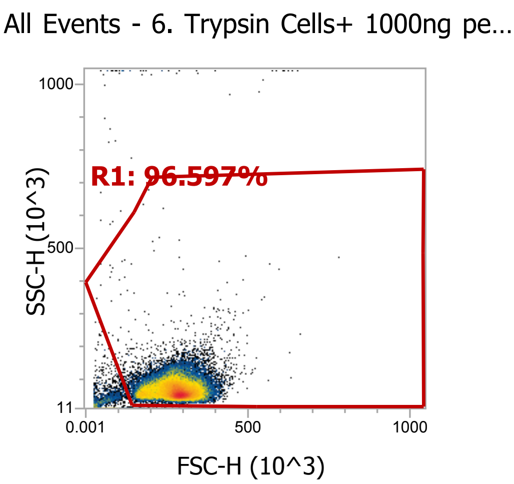

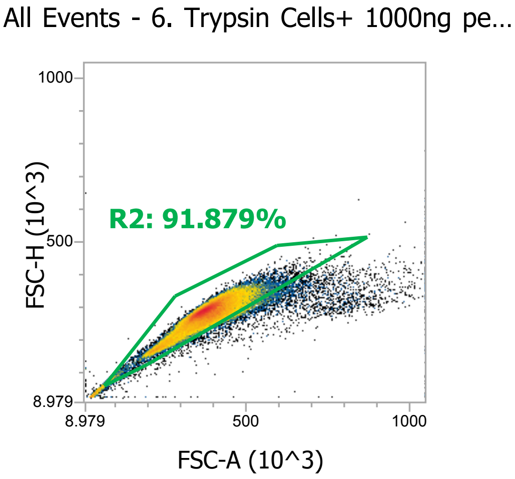

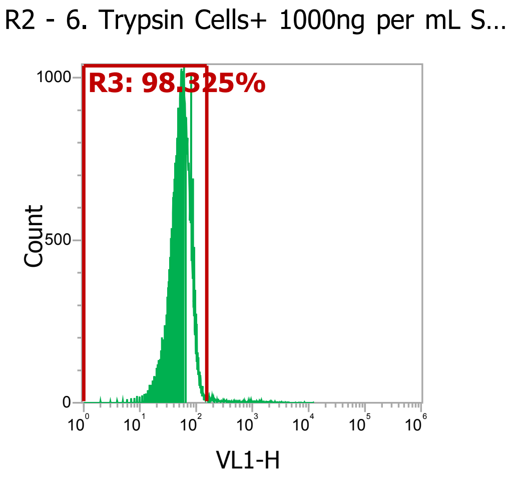

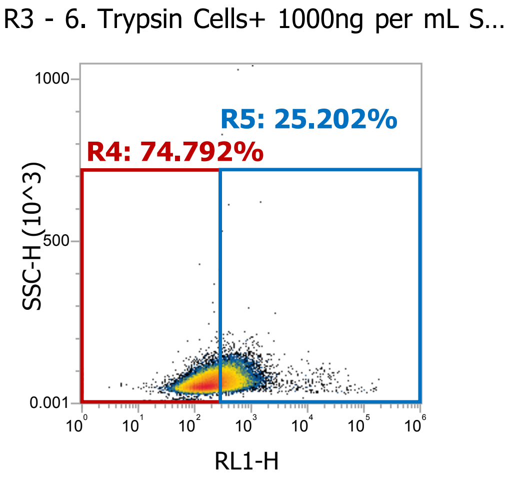

Experiment: **20220511**
Sample: **6. Trypsin Cells+ 1000ng per mL SEQ2 Cy5**
Time Recorded: **14:51:40**

Name	Gate	X Parameter	Y Parameter	Count	%Total	%Gated
All Events	All Events	N/A	N/A	55,179	100.000	100.000
R1	R1	FSC - FSC-H	SSC - SSC-H	53,301	96.597	96.597
R2	R2	FSC - FSC-A	FSC - FSC-H	50,698	91.879	91.879
R3	R3	VL1 - VL1-H		49,849	90.341	98.325
R4	R4	RL1 - RL1-H	SSC - SSC-H	37,283	67.567	74.792
R5	R5	RL1 - RL1-H	SSC - SSC-H	12,563	22.768	25.202

All Events - 7. Trypsin Cells+ 3000ng pe...

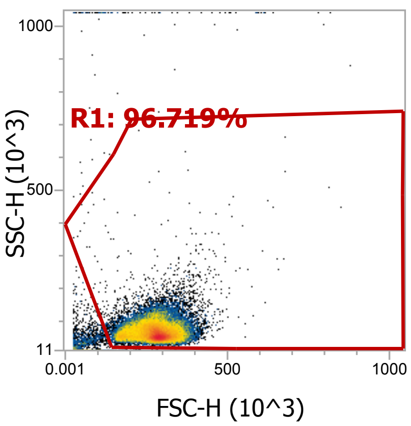

All Events - 7. Trypsin Cells+ 3000ng pe...

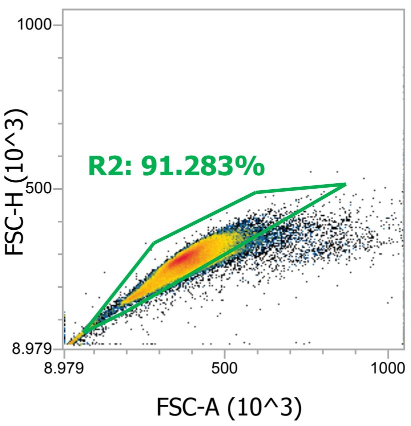

R2 - 7. Trypsin Cells+ 3000ng per mL S...

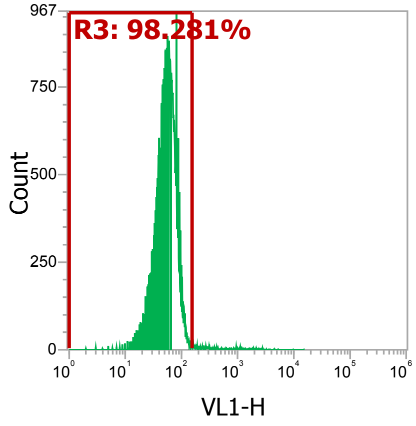

R3 - 7. Trypsin Cells+ 3000ng per mL S...

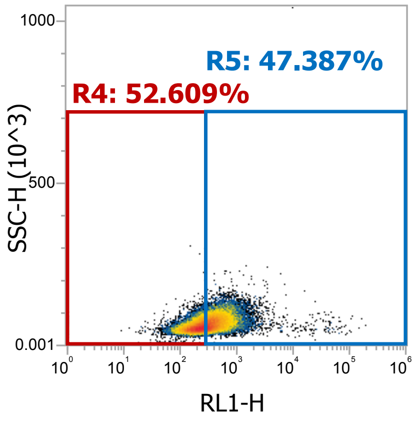

Experiment: **20220511**

Sample: **7. Trypsin Cells+ 3000ng per mL SEQ2 Cy5**

Time Recorded: **14:53:09**

Name	Gate	X Parameter	Y Parameter	Count	%Total	%Gated
All Events	All Events	N/A	N/A	49,136	100.000	100.000
R1	R1	FSC - FSC-H	SSC - SSC-H	47,524	96.719	96.719
R2	R2	FSC - FSC-A	FSC - FSC-H	44,853	91.283	91.283
R3	R3	VL1 - VL1-H		44,082	89.714	98.281
R4	R4	RL1 - RL1-H	SSC - SSC-H	23,191	47.198	52.609
R5	R5	RL1 - RL1-H	SSC - SSC-H	20,889	42.513	47.387

All Events - 8. Trypsin Cells+ 10000ng p...

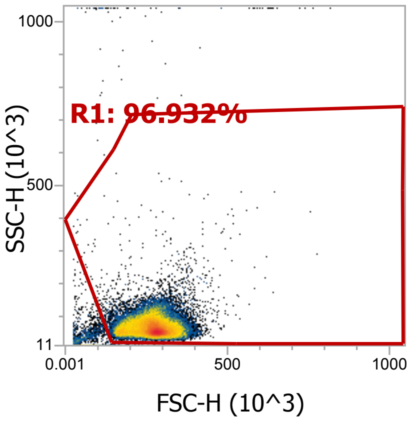

All Events - 8. Trypsin Cells+ 10000ng p...

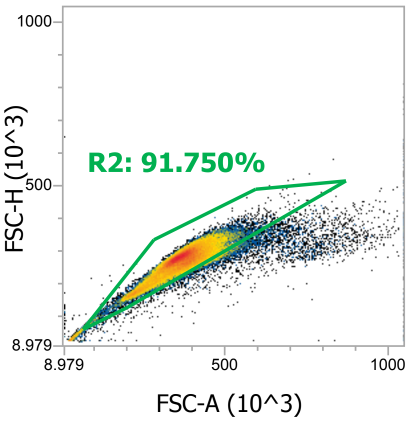

R2 - 8. Trypsin Cells+ 10000ng per mL...

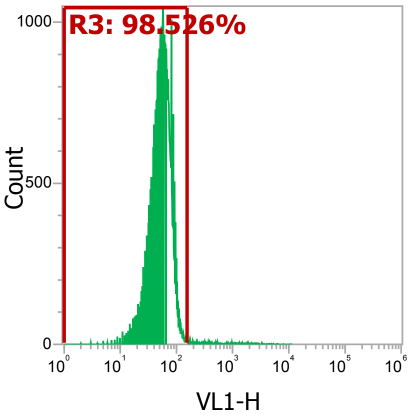

R3 - 8. Trypsin Cells+ 10000ng per mL...

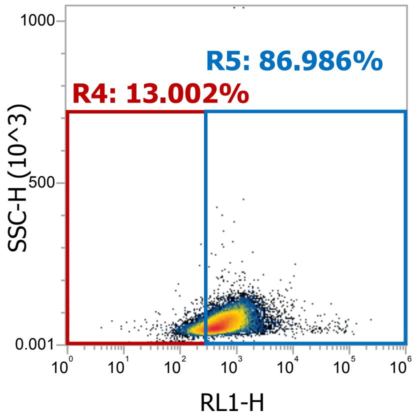

Experiment: **20220511**
Sample: **8. Trypsin Cells+ 10000ng per mL SEQ2 Cy5**
Time Recorded: **14:54:38**

Name	Gate	X Parameter	Y Parameter	Count	%Total	%Gated
All Events	All Events	N/A	N/A	55,021	100.000	100.000
R1	R1	FSC - FSC-H	SSC - SSC-H	53,333	96.932	96.932
R2	R2	FSC - FSC-A	FSC - FSC-H	50,482	91.750	91.750
R3	R3	VL1 - VL1-H		49,738	90.398	98.526
R4	R4	RL1 - RL1-H	SSC - SSC-H	6,467	11.754	13.002
R5	R5	RL1 - RL1-H	SSC - SSC-H	43,265	78.634	86.986

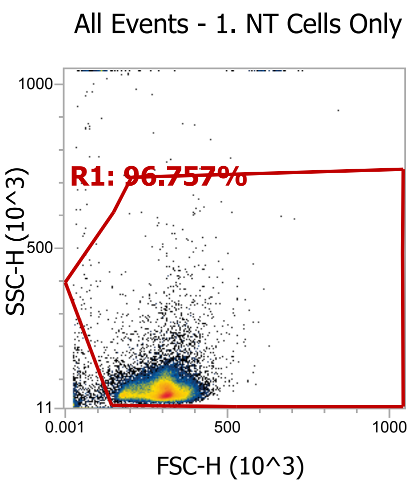

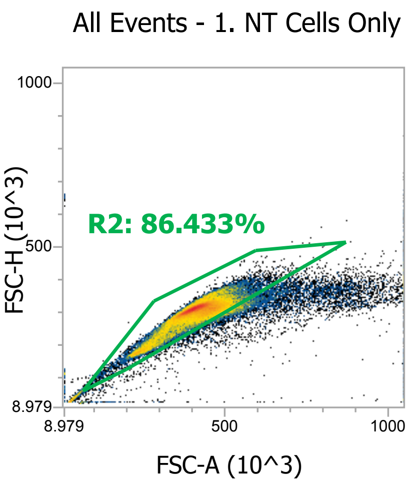

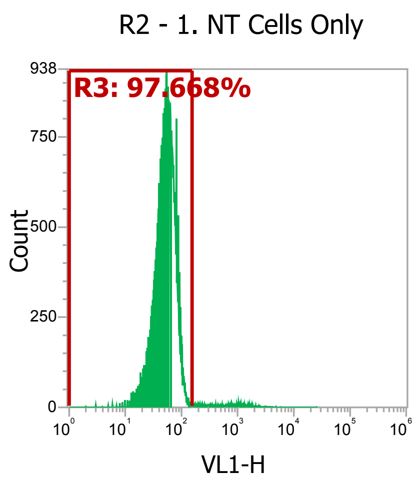

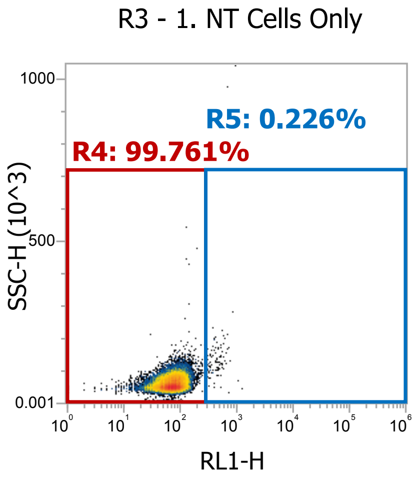

Experiment: **20220511**
Sample: **1. NT Cells Only**
Time Recorded: **14:59:04**

Name	Gate	X Parameter	Y Parameter	Count	%Total	%Gated
All Events	All Events	N/A	N/A	51,440	100.000	100.000
R1	R1	FSC - FSC-H	SSC - SSC-H	49,772	96.757	96.757
R2	R2	FSC - FSC-A	FSC - FSC-H	44,461	86.433	86.433
R3	R3	VL1 - VL1-H		43,424	84.417	97.668
R4	R4	RL1 - RL1-H	SSC - SSC-H	43,320	84.215	99.761
R5	R5	RL1 - RL1-H	SSC - SSC-H	98	0.191	0.226

All Events - 2. NT Cells+ 10ng per mL S...

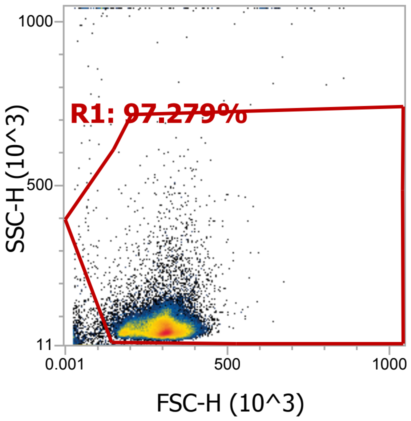

All Events - 2. NT Cells+ 10ng per mL S...

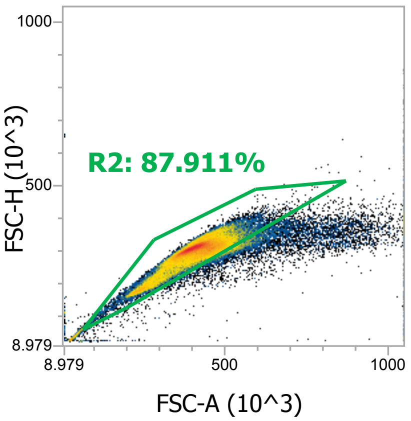

R2 - 2. NT Cells+ 10ng per mL SEQ2 Cy5

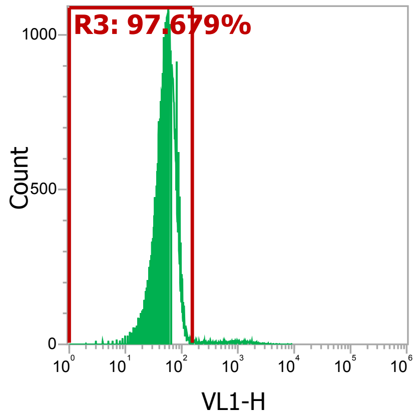

R3 - 2. NT Cells+ 10ng per mL SEQ2 Cy5

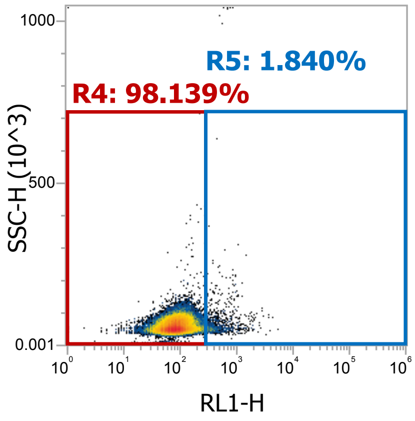

Experiment: **20220511**
Sample: **2. NT Cells+ 10ng per mL SEQ2 Cy5**
Time Recorded: **15:00:33**

Name	Gate	X Parameter	Y Parameter	Count	%Total	%Gated
All Events	All Events	N/A	N/A	60,079	100.000	100.000
R1	R1	FSC - FSC-H	SSC - SSC-H	58,444	97.279	97.279
R2	R2	FSC - FSC-A	FSC - FSC-H	52,816	87.911	87.911
R3	R3	VL1 - VL1-H		51,590	85.870	97.679
R4	R4	RL1 - RL1-H	SSC - SSC-H	50,630	84.272	98.139
R5	R5	RL1 - RL1-H	SSC - SSC-H	949	1.580	1.840

All Events - 3. NT Cells+ 30ng per mL S...

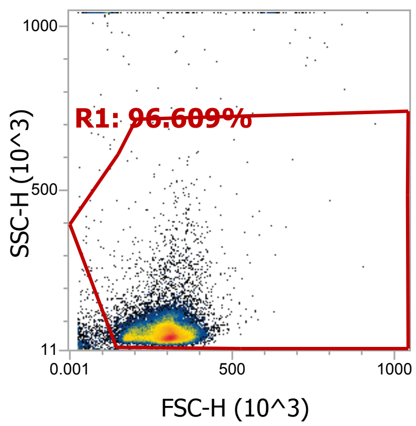

All Events - 3. NT Cells+ 30ng per mL S...

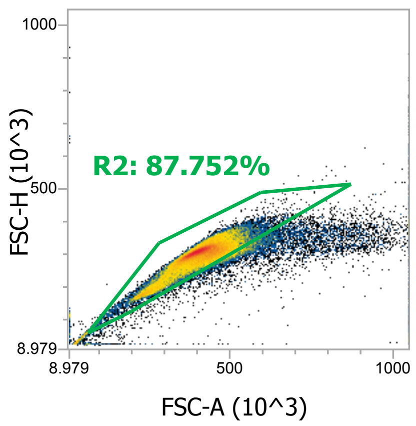

R2 - 3. NT Cells+ 30ng per mL SEQ2 Cy5

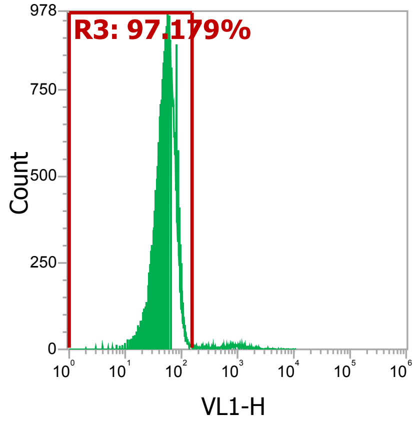

R3 - 3. NT Cells+ 30ng per mL SEQ2 Cy5

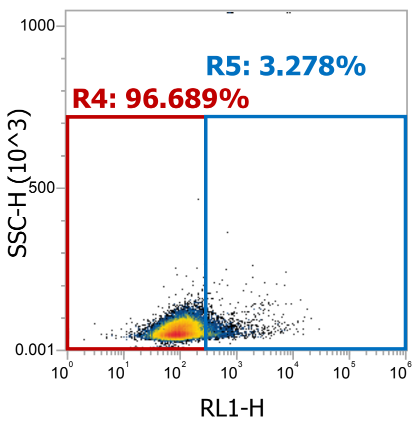

Experiment: **20220511**
Sample: **3. NT Cells+ 30ng per mL SEQ2 Cy5**
Time Recorded: **15:02:03**

Name	Gate	X Parameter	Y Parameter	Count	%Total	%Gated
All Events	All Events	N/A	N/A	53,517	100.000	100.000
R1	R1	FSC - FSC-H	SSC - SSC-H	51,702	96.609	96.609
R2	R2	FSC - FSC-A	FSC - FSC-H	46,962	87.752	87.752
R3	R3	VL1 - VL1-H		45,637	85.276	97.179
R4	R4	RL1 - RL1-H	SSC - SSC-H	44,126	82.452	96.689
R5	R5	RL1 - RL1-H	SSC - SSC-H	1,496	2.795	3.278

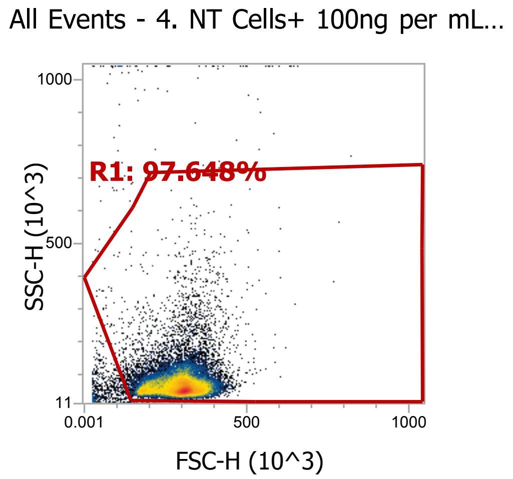

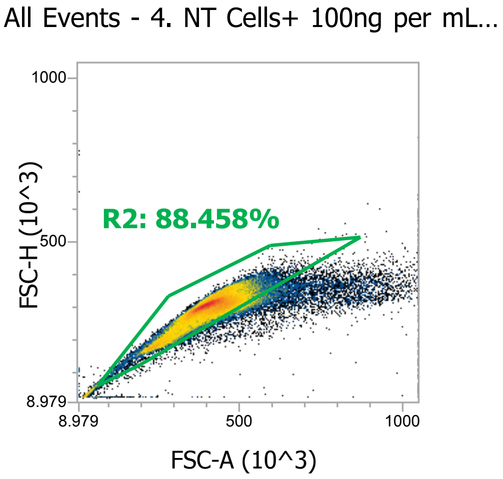

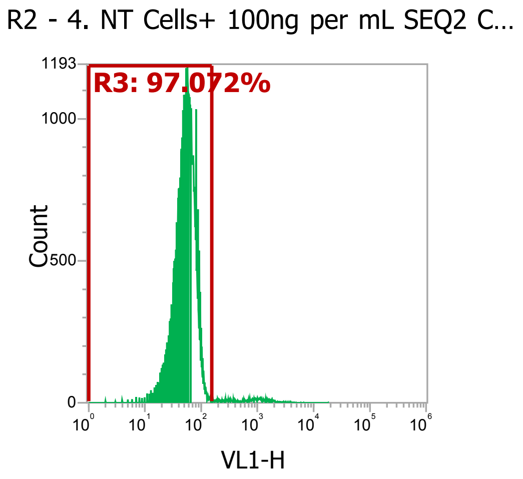

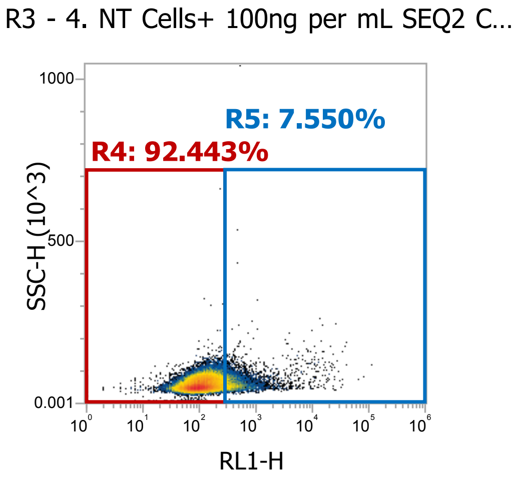

Experiment: **20220511**
Sample: **4. NT Cells+ 100ng per mL SEQ2 Cy5**
Time Recorded: **15:03:32**

Name	Gate	X Parameter	Y Parameter	Count	%Total	%Gated
All Events	All Events	N/A	N/A	64,445	100.000	100.000
R1	R1	FSC - FSC-H	SSC - SSC-H	62,929	97.648	97.648
R2	R2	FSC - FSC-A	FSC - FSC-H	57,007	88.458	88.458
R3	R3	VL1 - VL1-H		55,338	85.869	97.072
R4	R4	RL1 - RL1-H	SSC - SSC-H	51,156	79.379	92.443
R5	R5	RL1 - RL1-H	SSC - SSC-H	4,178	6.483	7.550

All Events - 5. NT Cells+ 300ng per mL...

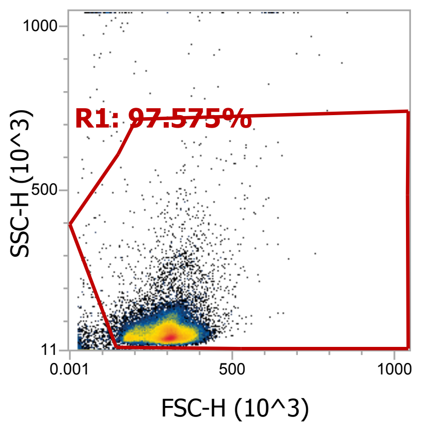

All Events - 5. NT Cells+ 300ng per mL...

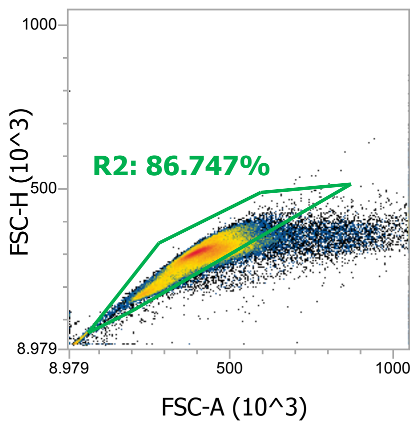

R2 - 5. NT Cells+ 300ng per mL SEQ2 C...

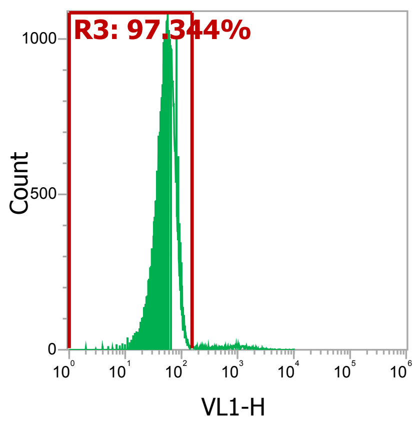

R3 - 5. NT Cells+ 300ng per mL SEQ2 C...

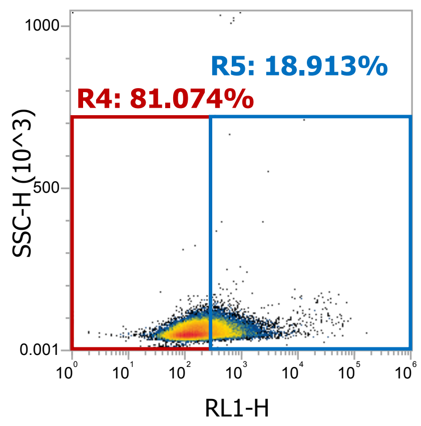

Experiment: **20220511**
Sample: **5. NT Cells+ 300ng per mL SEQ2 Cy5**
Time Recorded: **15:05:01**

Name	Gate	X Parameter	Y Parameter	Count	%Total	%Gated
All Events	All Events	N/A	N/A	60,380	100.000	100.000
R1	R1	FSC - FSC-H	SSC - SSC-H	58,916	97.575	97.575
R2	R2	FSC - FSC-A	FSC - FSC-H	52,378	86.747	86.747
R3	R3	VL1 - VL1-H		50,987	84.444	97.344
R4	R4	RL1 - RL1-H	SSC - SSC-H	41,337	68.461	81.074
R5	R5	RL1 - RL1-H	SSC - SSC-H	9,643	15.971	18.913

All Events - 6. NT Cells+ 1000ng per mL...

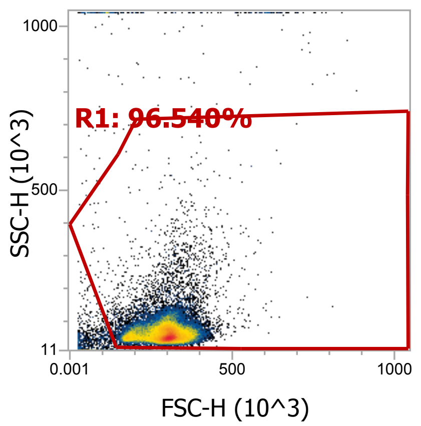

All Events - 6. NT Cells+ 1000ng per mL...

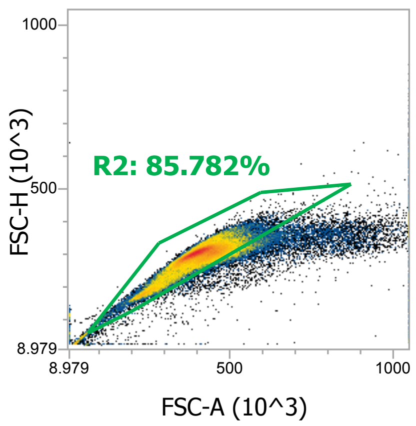

R2 - 6. NT Cells+ 1000ng per mL SEQ2...

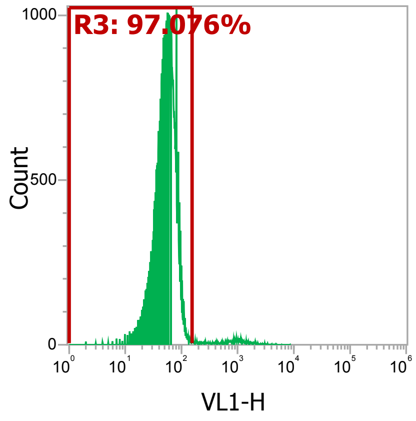

R3 - 6. NT Cells+ 1000ng per mL SEQ2...

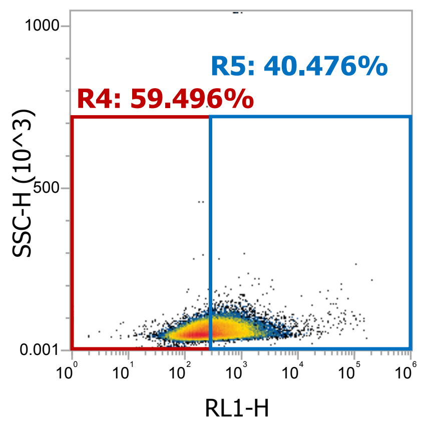

Experiment: **20220511**
Sample: **6. NT Cells+ 1000ng per mL SEQ2 Cy5**
Time Recorded: **15:06:30**

Name	Gate	X Parameter	Y Parameter	Count	%Total	%Gated
All Events	All Events	N/A	N/A	59,770	100.000	100.000
R1	R1	FSC - FSC-H	SSC - SSC-H	57,702	96.540	96.540
R2	R2	FSC - FSC-A	FSC - FSC-H	51,272	85.782	85.782
R3	R3	VL1 - VL1-H		49,773	83.274	97.076
R4	R4	RL1 - RL1-H	SSC - SSC-H	29,613	49.545	59.496
R5	R5	RL1 - RL1-H	SSC - SSC-H	20,146	33.706	40.476

All Events - 7. NT Cells+ 3000ng per mL...

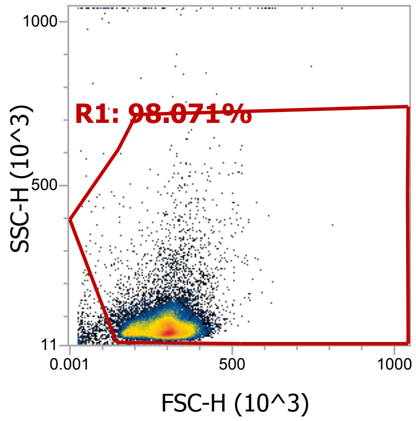

All Events - 7. NT Cells+ 3000ng per mL...

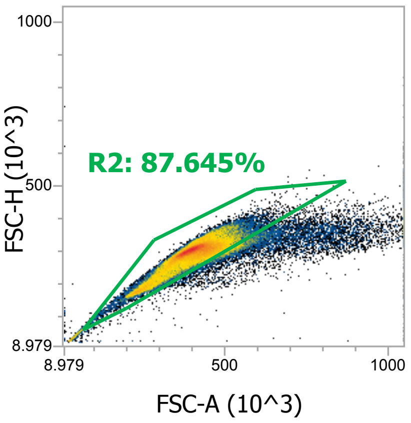

R2 - 7. NT Cells+ 3000ng per mL SEQ2...

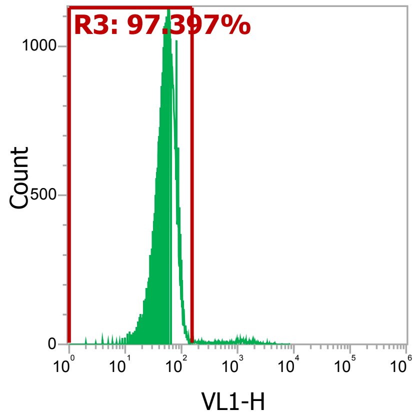

R3 - 7. NT Cells+ 3000ng per mL SEQ2...

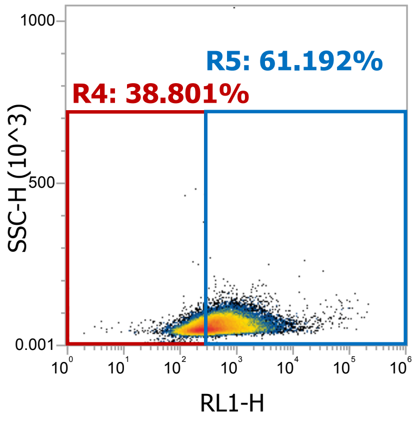

Experiment: **20220511**
Sample: **7. NT Cells+ 3000ng per mL SEQ2 Cy5**
Time Recorded: **15:07:58**

Name	Gate	X Parameter	Y Parameter	Count	%Total	%Gated
All Events	All Events	N/A	N/A	63,333	100.000	100.000
R1	R1	FSC - FSC-H	SSC - SSC-H	62,111	98.071	98.071
R2	R2	FSC - FSC-A	FSC - FSC-H	55,508	87.645	87.645
R3	R3	VL1 - VL1-H		54,063	85.363	97.397
R4	R4	RL1 - RL1-H	SSC - SSC-H	20,977	33.122	38.801
R5	R5	RL1 - RL1-H	SSC - SSC-H	33,082	52.235	61.192

All Events - 8. NT Cells+ 10000ng per m...

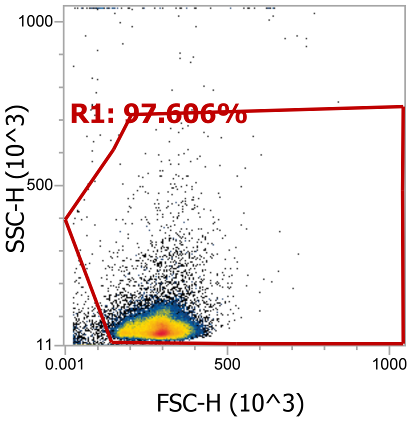

All Events - 8. NT Cells+ 10000ng per m...

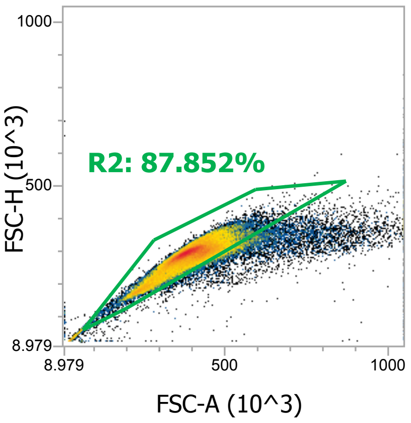

R2 - 8. NT Cells+ 10000ng per mL SEQ...

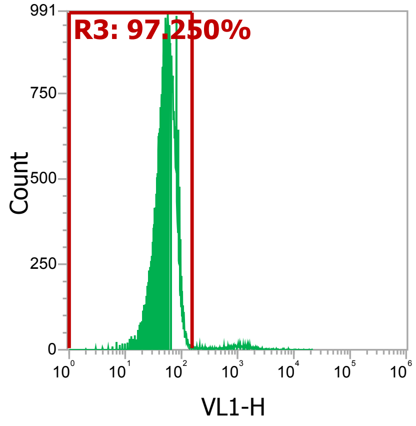

R3 - 8. NT Cells+ 10000ng per mL SEQ...

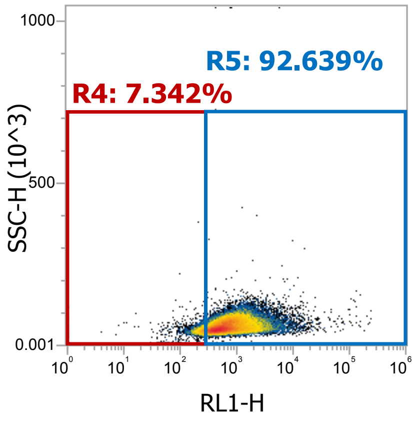

Experiment: **20220511**
Sample: **8. NT Cells+ 10000ng per mL SEQ2 Cy5**
Time Recorded: **15:09:27**

Name	Gate	X Parameter	Y Parameter	Count	%Total	%Gated
All Events	All Events	N/A	N/A	55,226	100.000	100.000
R1	R1	FSC - FSC-H	SSC - SSC-H	53,904	97.606	97.606
R2	R2	FSC - FSC-A	FSC - FSC-H	48,517	87.852	87.852
R3	R3	VL1 - VL1-H		47,183	85.436	97.250
R4	R4	RL1 - RL1-H	SSC - SSC-H	3,464	6.272	7.342
R5	R5	RL1 - RL1-H	SSC - SSC-H	43,710	79.148	92.639
