# Supplementary material for: A retrotransposon-derived DNA zip code internalizes myeloma cells through Clathrin-Rab5a-mediated endocytosis
Source: Front Oncol. 2024 Feb 23;14:1288724. doi: 10.3389/fonc.2024.1288724 (PMC10920344; doi:10.3389/fonc.2024.1288724)
Supplement: Supplementary file 12 [file DataSheet_1.zip › Raw data/Fig 1C, MM1S Timepoints/12hr NT Cells+MMZC Cy5.pdf]

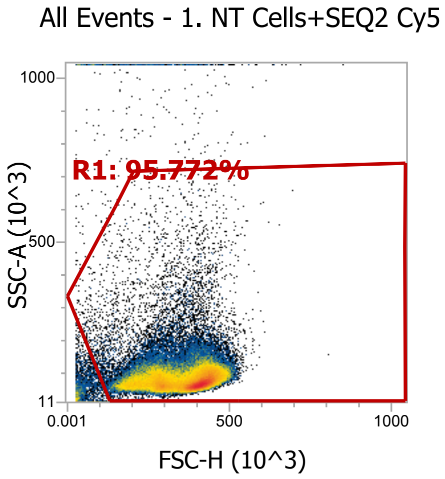

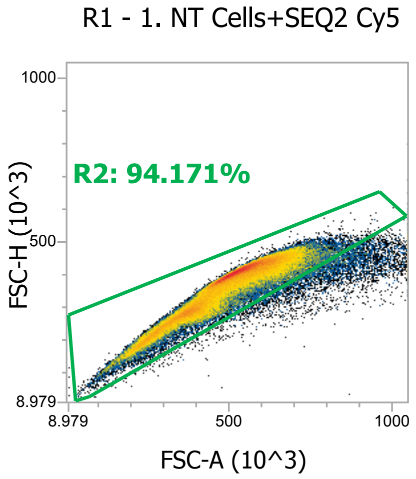

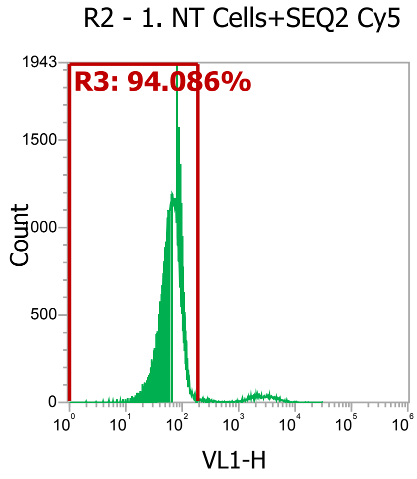

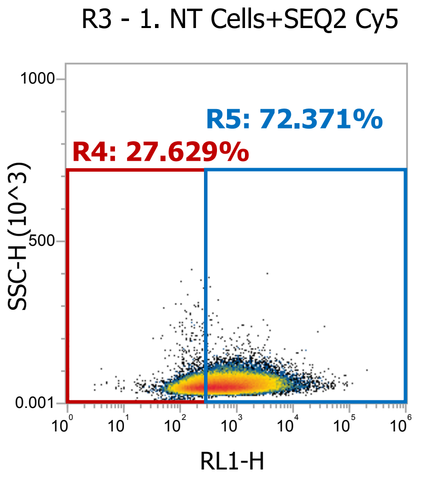

Experiment: **20210901**
Sample: **1. NT Cells+SEQ2 Cy5**
Time Recorded: **20:28:29**

Name	Gate	X Parameter	Y Parameter	Count	%Total	%Gated
All Events	All Events	N/A	N/A	82,757	100.000	100.000
R1	R1	FSC - FSC-H	SSC - SSC-A	79,258	95.772	95.772
R2	R2	FSC - FSC-A	FSC - FSC-H	74,638	90.189	94.171
R3	R3	VL1 - VL1-H		70,224	84.856	94.086
R4	R4	RL1 - RL1-H	SSC - SSC-H	19,402	23.445	27.629
R5	R5	RL1 - RL1-H	SSC - SSC-H	50,822	61.411	72.371

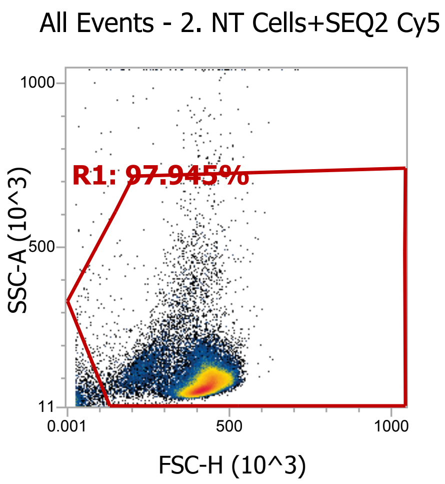

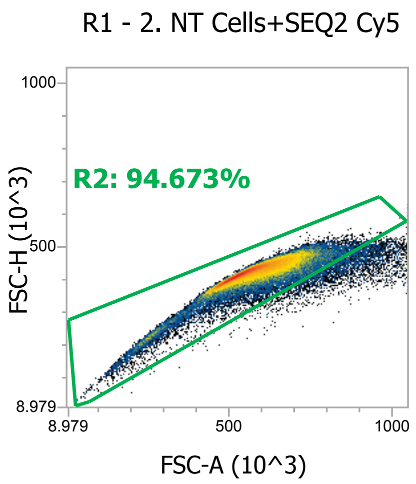

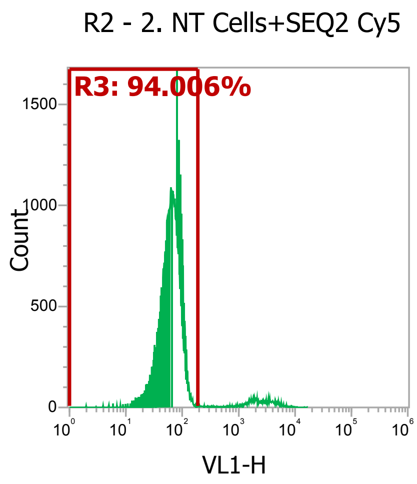

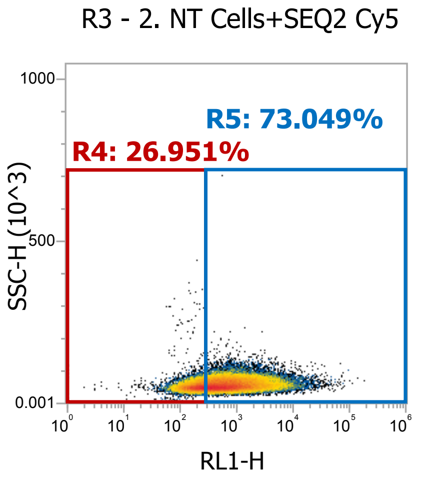

Experiment: **20210908**
Sample: **2. NT Cells+SEQ2 Cy5**
Time Recorded: **21:35:34**

Name	Gate	X Parameter	Y Parameter	Count	%Total	%Gated
All Events	All Events	N/A	N/A	70,911	100.000	100.000
R1	R1	FSC - FSC-H	SSC - SSC-A	69,454	97.945	97.945
R2	R2	FSC - FSC-A	FSC - FSC-H	65,754	92.728	94.673
R3	R3	VL1 - VL1-H		61,813	87.170	94.006
R4	R4	RL1 - RL1-H	SSC - SSC-H	16,659	23.493	26.951
R5	R5	RL1 - RL1-H	SSC - SSC-H	45,154	63.677	73.049

All Events - 3. NT Cells+SEQ2 Cy5

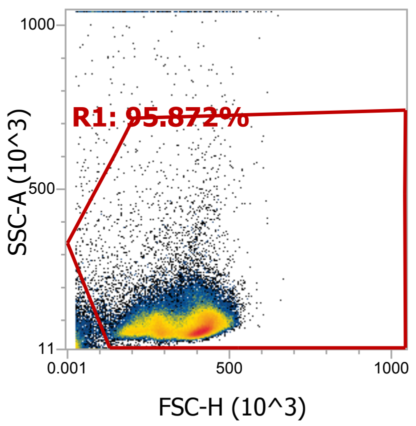

R1 - 3. NT Cells+SEQ2 Cy5

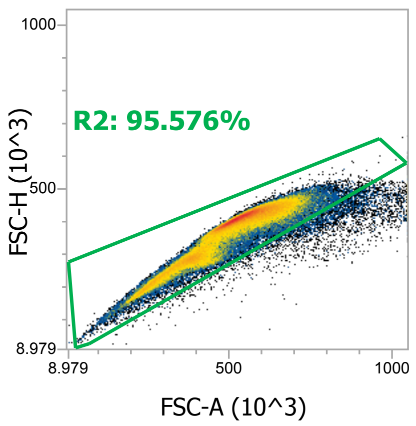

R2 - 3. NT Cells+SEQ2 Cy5

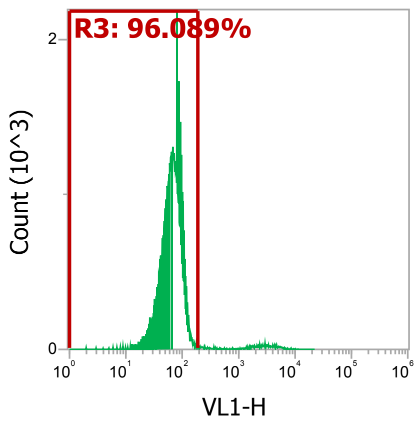

R3 - 3. NT Cells+SEQ2 Cy5

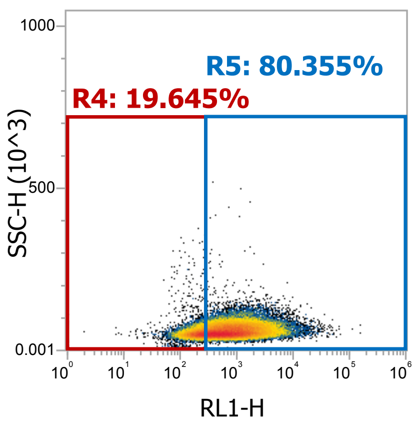

Experiment: **20210917**
Sample: **3. NT Cells+SEQ2 Cy5**
Time Recorded: **19:20:40**

Name	Gate	X Parameter	Y Parameter	Count	%Total	%Gated
All Events	All Events	N/A	N/A	85,165	100.000	100.000
R1	R1	FSC - FSC-H	SSC - SSC-A	81,649	95.872	95.872
R2	R2	FSC - FSC-A	FSC - FSC-H	78,037	91.630	95.576
R3	R3	VL1 - VL1-H		74,985	88.047	96.089
R4	R4	RL1 - RL1-H	SSC - SSC-H	14,731	17.297	19.645
R5	R5	RL1 - RL1-H	SSC - SSC-H	60,254	70.750	80.355
