# Supplementary material for: A retrotransposon-derived DNA zip code internalizes myeloma cells through Clathrin-Rab5a-mediated endocytosis
Source: Front Oncol. 2024 Feb 23;14:1288724. doi: 10.3389/fonc.2024.1288724 (PMC10920344; doi:10.3389/fonc.2024.1288724)
Supplement: Supplementary file 12 [file DataSheet_1.zip › Raw data/Fig 1C, MM1S Timepoints/12hr Trypsin Cells+MMZC Cy5.pdf]

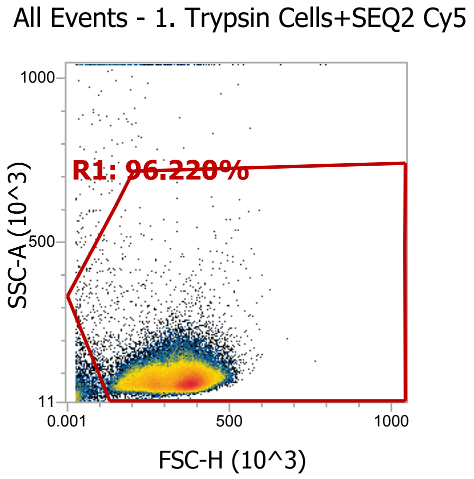

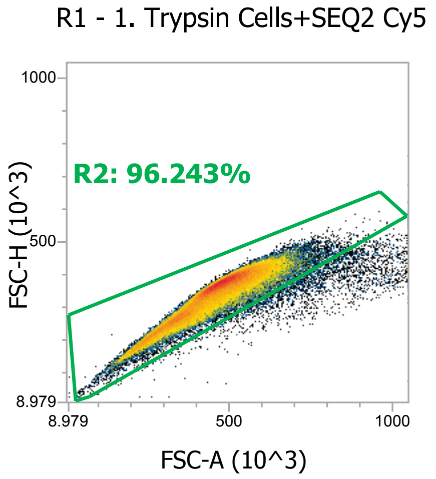

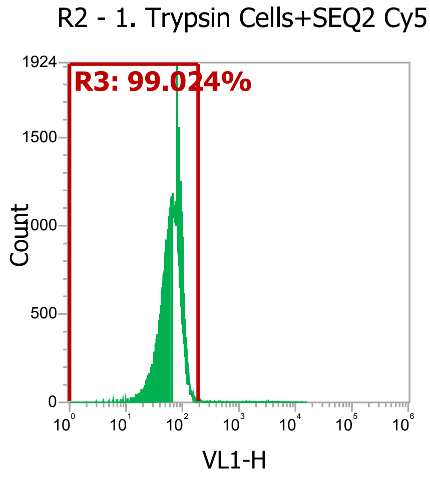

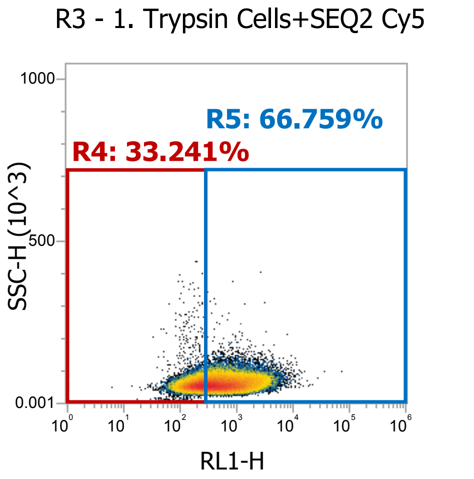

Experiment: **20210901**
Sample: **1. Trypsin Cells+SEQ2 Cy5**
Time Recorded: **20:26:57**

Name	Gate	X Parameter	Y Parameter	Count	%Total	%Gated
All Events	All Events	N/A	N/A	72,889	100.000	100.000
R1	R1	FSC - FSC-H	SSC - SSC-A	70,134	96.220	96.220
R2	R2	FSC - FSC-A	FSC - FSC-H	67,499	92.605	96.243
R3	R3	VL1 - VL1-H		66,840	91.701	99.024
R4	R4	RL1 - RL1-H	SSC - SSC-H	22,218	30.482	33.241
R5	R5	RL1 - RL1-H	SSC - SSC-H	44,622	61.219	66.759

All Events - 2. Trypsin Cells+SEQ2 Cy5

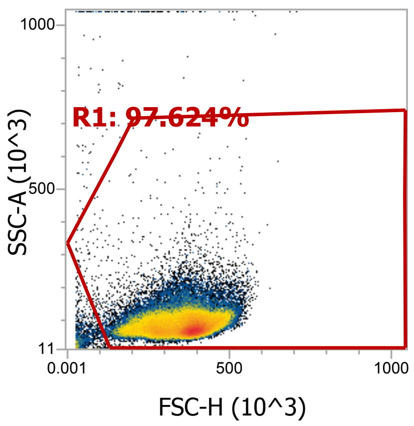

R1 - 2. Trypsin Cells+SEQ2 Cy5

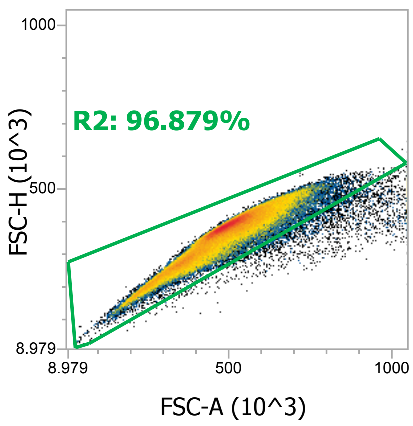

R2 - 2. Trypsin Cells+SEQ2 Cy5

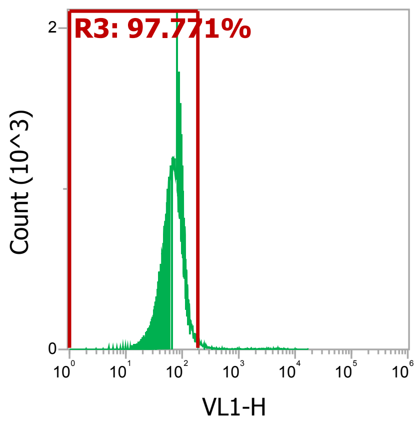

R3 - 2. Trypsin Cells+SEQ2 Cy5

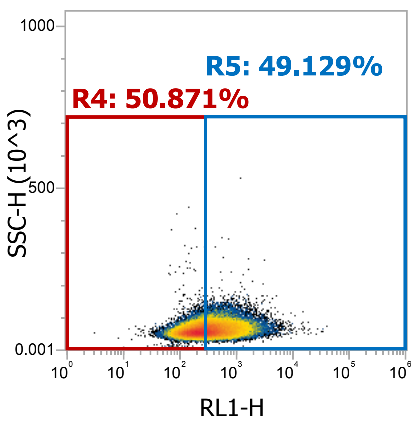

Experiment: **20210908**
Sample: **2. Trypsin Cells+SEQ2 Cy5**
Time Recorded: **21:34:00**

Name	Gate	X Parameter	Y Parameter	Count	%Total	%Gated
All Events	All Events	N/A	N/A	80,772	100.000	100.000
R1	R1	FSC - FSC-H	SSC - SSC-A	78,853	97.624	97.624
R2	R2	FSC - FSC-A	FSC - FSC-H	76,392	94.577	96.879
R3	R3	VL1 - VL1-H		74,689	92.469	97.771
R4	R4	RL1 - RL1-H	SSC - SSC-H	37,995	47.040	50.871
R5	R5	RL1 - RL1-H	SSC - SSC-H	36,694	45.429	49.129

All Events - 3. Trypsin Cells+SEQ2 Cy5

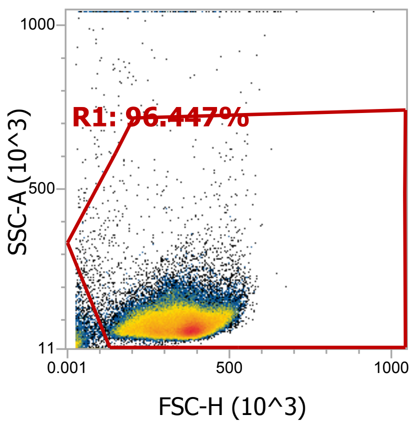

R1 - 3. Trypsin Cells+SEQ2 Cy5

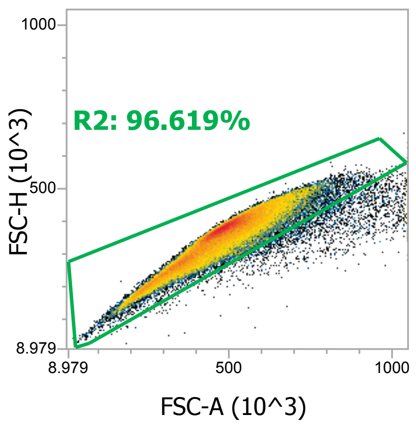

R2 - 3. Trypsin Cells+SEQ2 Cy5

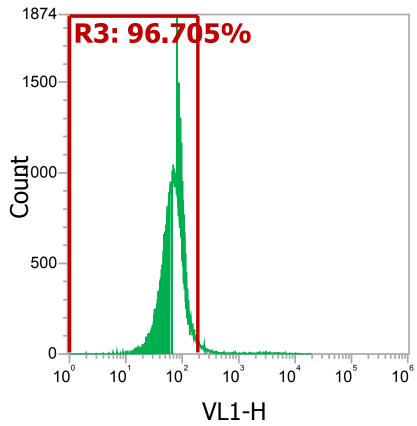

R3 - 3. Trypsin Cells+SEQ2 Cy5

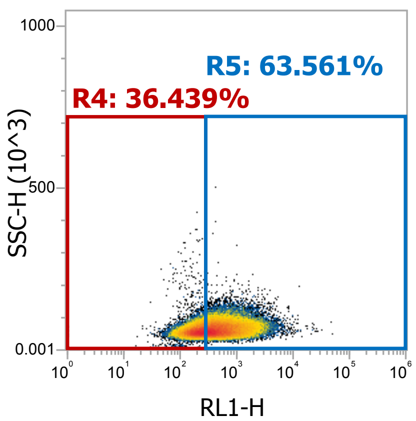

Experiment: **20210917**
Sample: **3. Trypsin Cells+SEQ2 Cy5**
Time Recorded: **19:18:24**

Name	Gate	X Parameter	Y Parameter	Count	%Total	%Gated
All Events	All Events	N/A	N/A	71,689	100.000	100.000
R1	R1	FSC - FSC-H	SSC - SSC-A	69,142	96.447	96.447
R2	R2	FSC - FSC-A	FSC - FSC-H	66,804	93.186	96.619
R3	R3	VL1 - VL1-H		64,603	90.116	96.705
R4	R4	RL1 - RL1-H	SSC - SSC-H	23,541	32.838	36.439
R5	R5	RL1 - RL1-H	SSC - SSC-H	41,062	57.278	63.561
