# Supplementary material for: A retrotransposon-derived DNA zip code internalizes myeloma cells through Clathrin-Rab5a-mediated endocytosis
Source: Front Oncol. 2024 Feb 23;14:1288724. doi: 10.3389/fonc.2024.1288724 (PMC10920344; doi:10.3389/fonc.2024.1288724)
Supplement: Supplementary file 12 [file DataSheet_1.zip › Raw data/Fig 1C, MM1S Timepoints/24hr NT Cells+MMZC Cy5.pdf]

All Events - 1. NT Cells+SEQ2 Cy5

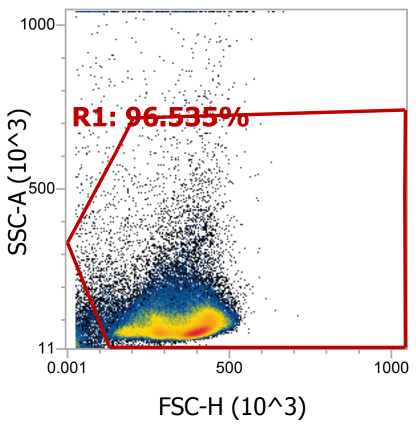

R1 - 1. NT Cells+SEQ2 Cy5

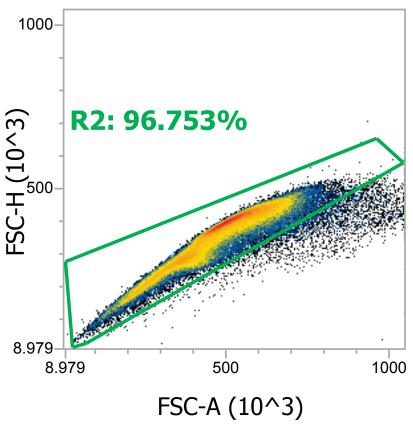

R2 - 1. NT Cells+SEQ2 Cy5

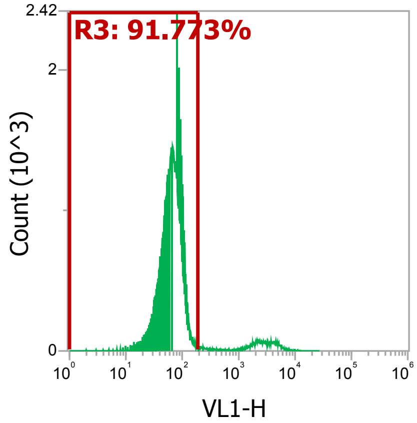

R3 - 1. NT Cells+SEQ2 Cy5

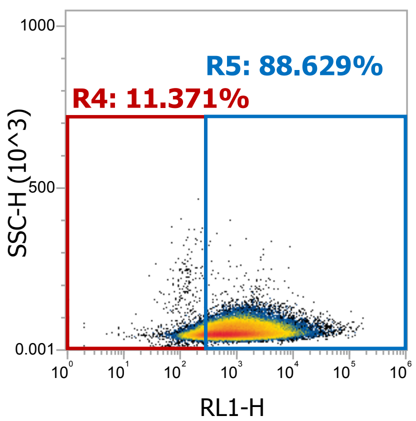

Experiment: **20210901**
Sample: **1. NT Cells+SEQ2 Cy5**
Time Recorded: **12:46:44**

Name	Gate	X Parameter	Y Parameter	Count	%Total	%Gated
All Events	All Events	N/A	N/A	101,024	100.000	100.000
R1	R1	FSC - FSC-H	SSC - SSC-A	97,524	96.535	96.535
R2	R2	FSC - FSC-A	FSC - FSC-H	94,357	93.401	96.753
R3	R3	VL1 - VL1-H		86,594	85.716	91.773
R4	R4	RL1 - RL1-H	SSC - SSC-H	9,847	9.747	11.371
R5	R5	RL1 - RL1-H	SSC - SSC-H	76,747	75.969	88.629

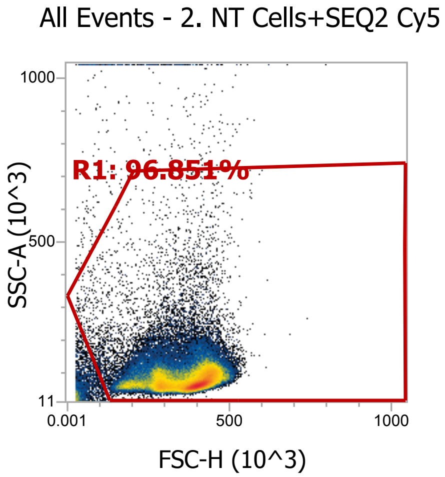

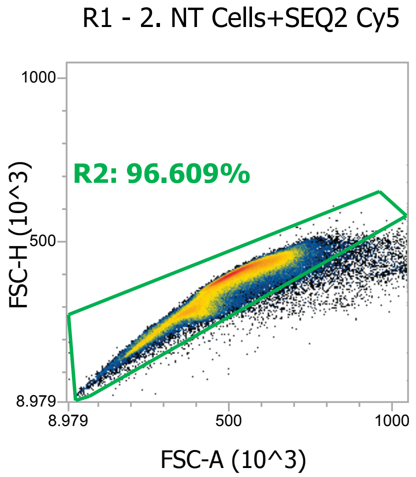

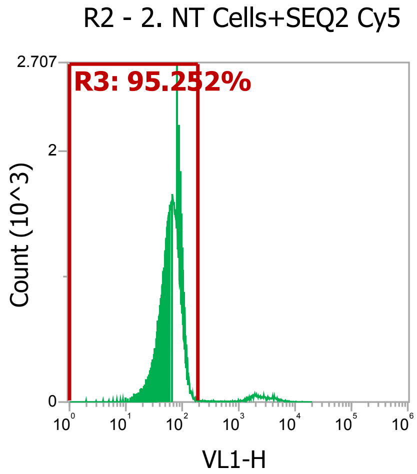

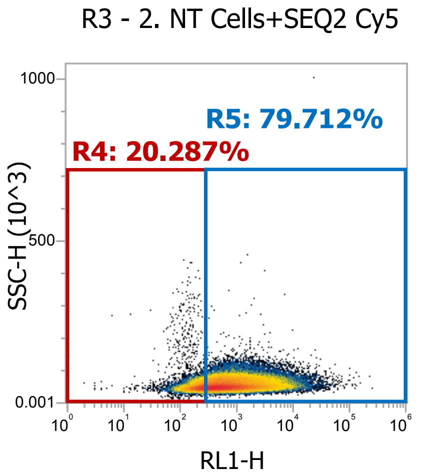

Experiment: **20210908**
Sample: **2. NT Cells+SEQ2 Cy5**
Time Recorded: **09:39:17**

Name	Gate	X Parameter	Y Parameter	Count	%Total	%Gated
All Events	All Events	N/A	N/A	108,282	100.000	100.000
R1	R1	FSC - FSC-H	SSC - SSC-A	104,872	96.851	96.851
R2	R2	FSC - FSC-A	FSC - FSC-H	101,316	93.567	96.609
R3	R3	VL1 - VL1-H		96,506	89.125	95.252
R4	R4	RL1 - RL1-H	SSC - SSC-H	19,578	18.081	20.287
R5	R5	RL1 - RL1-H	SSC - SSC-H	76,927	71.043	79.712

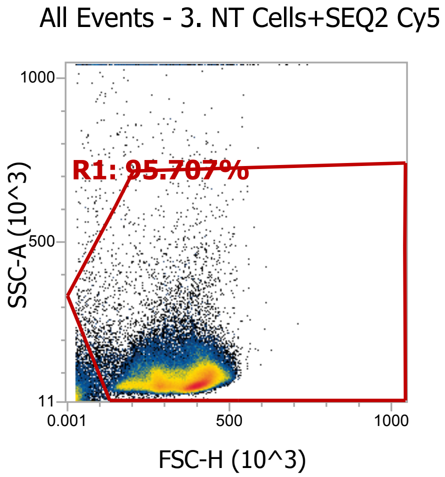

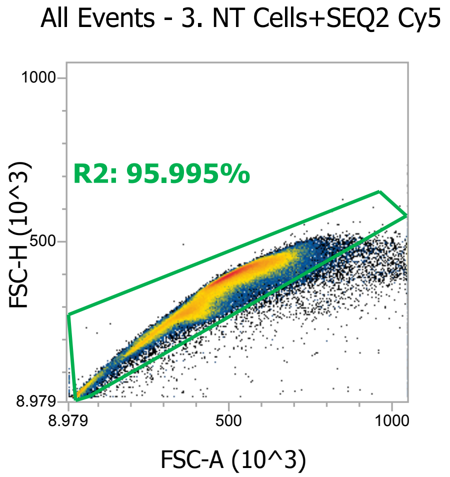

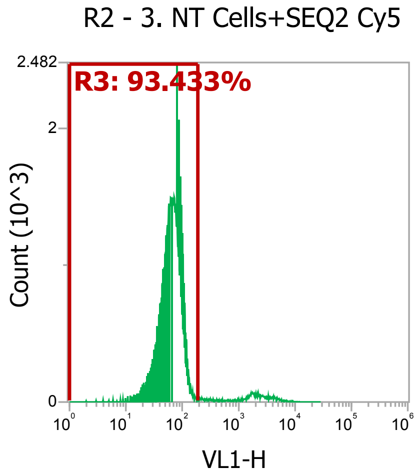

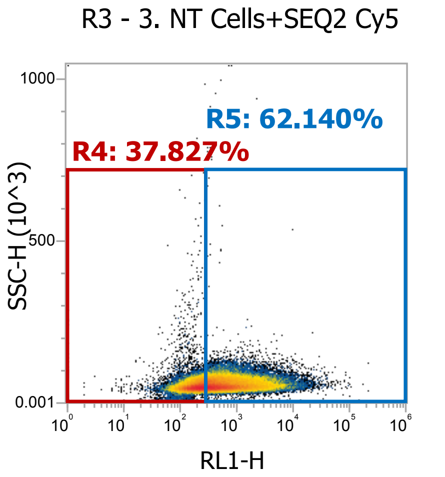

Experiment: **20210910**
Sample: **3. NT Cells+SEQ2 Cy5**
Time Recorded: **12:46:10**

Name	Gate	X Parameter	Y Parameter	Count	%Total	%Gated
All Events	All Events	N/A	N/A	100,774	100.000	100.000
R1	R1	FSC - FSC-H	SSC - SSC-A	96,448	95.707	95.707
R2	R2	FSC - FSC-A	FSC - FSC-H	96,738	95.995	95.995
R3	R3	VL1 - VL1-H		90,385	89.691	93.433
R4	R4	RL1 - RL1-H	SSC - SSC-H	34,190	33.927	37.827
R5	R5	RL1 - RL1-H	SSC - SSC-H	56,165	55.734	62.140

All Events - 4. NT Cells+SEQ2 Cy5

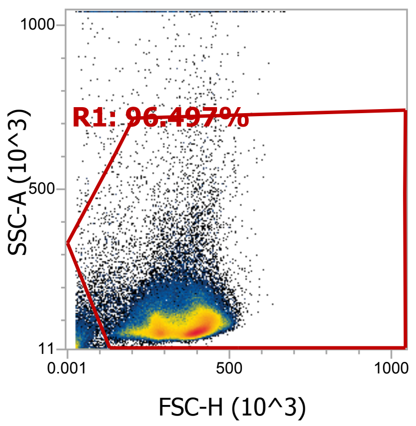

R1 - 4. NT Cells+SEQ2 Cy5

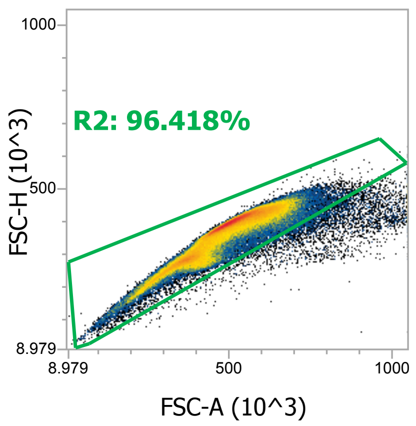

R2 - 4. NT Cells+SEQ2 Cy5

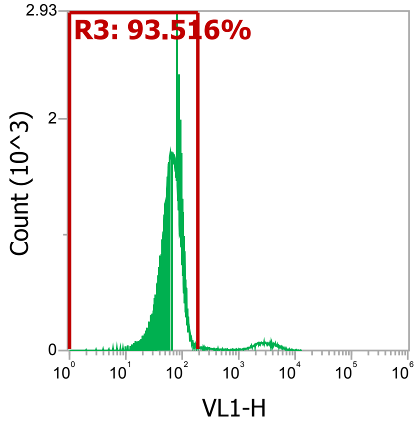

R3 - 4. NT Cells+SEQ2 Cy5

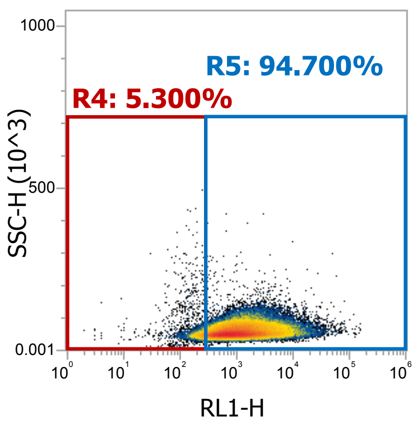

Experiment: **20210917**
Sample: **4. NT Cells+SEQ2 Cy5**
Time Recorded: **11:37:03**

Name	Gate	X Parameter	Y Parameter	Count	%Total	%Gated
All Events	All Events	N/A	N/A	118,198	100.000	100.000
R1	R1	FSC - FSC-H	SSC - SSC-A	114,058	96.497	96.497
R2	R2	FSC - FSC-A	FSC - FSC-H	109,972	93.040	96.418
R3	R3	VL1 - VL1-H		102,841	87.007	93.516
R4	R4	RL1 - RL1-H	SSC - SSC-H	5,451	4.612	5.300
R5	R5	RL1 - RL1-H	SSC - SSC-H	97,390	82.396	94.700
