# Supplementary material for: A retrotransposon-derived DNA zip code internalizes myeloma cells through Clathrin-Rab5a-mediated endocytosis
Source: Front Oncol. 2024 Feb 23;14:1288724. doi: 10.3389/fonc.2024.1288724 (PMC10920344; doi:10.3389/fonc.2024.1288724)
Supplement: Supplementary file 12 [file DataSheet_1.zip › Raw data/Fig 1C, MM1S Timepoints/24hr Trypsin Cells+MMZC Cy5.pdf]

All Events - 1. Trypsin Cells+SEQ2 Cy5

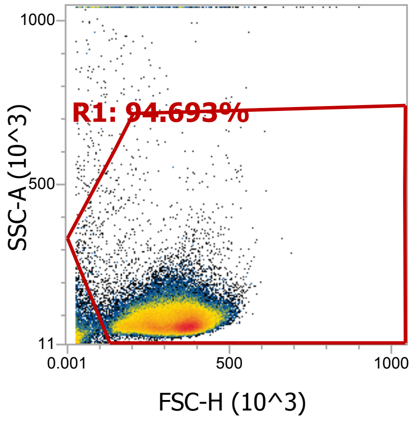

R1 - 1. Trypsin Cells+SEQ2 Cy5

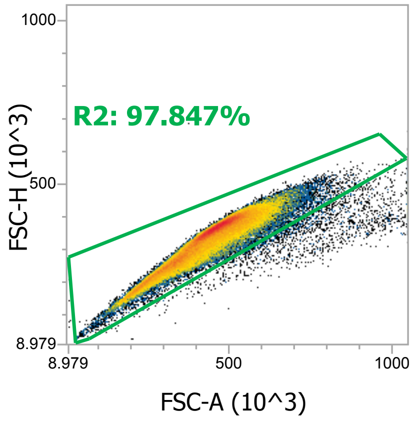

R2 - 1. Trypsin Cells+SEQ2 Cy5

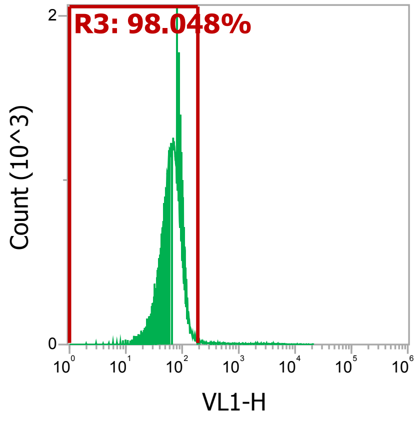

R3 - 1. Trypsin Cells+SEQ2 Cy5

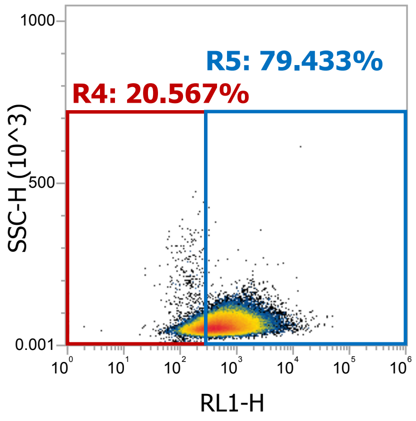

Experiment: **20210901**
Sample: **1. Trypsin Cells+SEQ2 Cy5**
Time Recorded: **12:45:10**

Name	Gate	X Parameter	Y Parameter	Count	%Total	%Gated
All Events	All Events	N/A	N/A	81,615	100.000	100.000
R1	R1	FSC - FSC-H	SSC - SSC-A	77,284	94.693	94.693
R2	R2	FSC - FSC-A	FSC - FSC-H	75,620	92.655	97.847
R3	R3	VL1 - VL1-H		74,144	90.846	98.048
R4	R4	RL1 - RL1-H	SSC - SSC-H	15,249	18.684	20.567
R5	R5	RL1 - RL1-H	SSC - SSC-H	58,895	72.162	79.433

All Events - 2. Trypsin Cells+SEQ2 Cy5

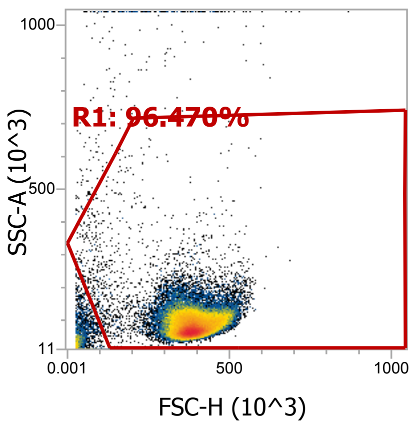

R1 - 2. Trypsin Cells+SEQ2 Cy5

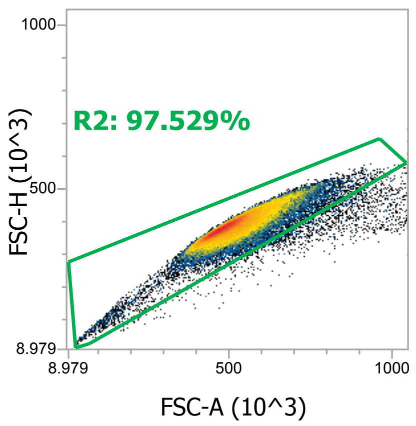

R2 - 2. Trypsin Cells+SEQ2 Cy5

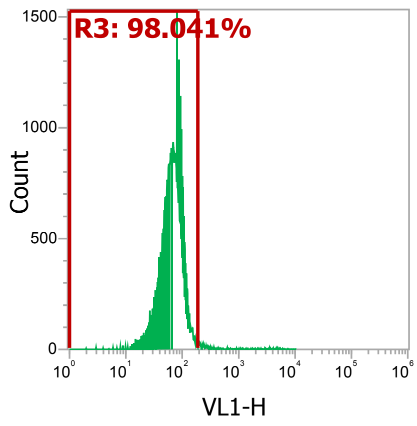

R3 - 2. Trypsin Cells+SEQ2 Cy5

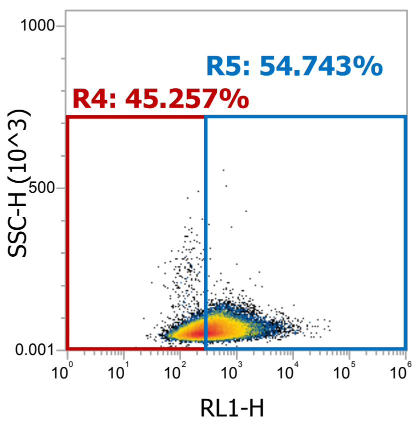

Experiment: **20210908**
Sample: **2. Trypsin Cells+SEQ2 Cy5**
Time Recorded: **09:37:18**

Name	Gate	X Parameter	Y Parameter	Count	%Total	%Gated
All Events	All Events	N/A	N/A	61,363	100.000	100.000
R1	R1	FSC - FSC-H	SSC - SSC-A	59,197	96.470	96.470
R2	R2	FSC - FSC-A	FSC - FSC-H	57,734	94.086	97.529
R3	R3	VL1 - VL1-H		56,603	92.243	98.041
R4	R4	RL1 - RL1-H	SSC - SSC-H	25,617	41.747	45.257
R5	R5	RL1 - RL1-H	SSC - SSC-H	30,986	50.496	54.743

All Events - 3. Trypsin Cells+SEQ2 Cy5

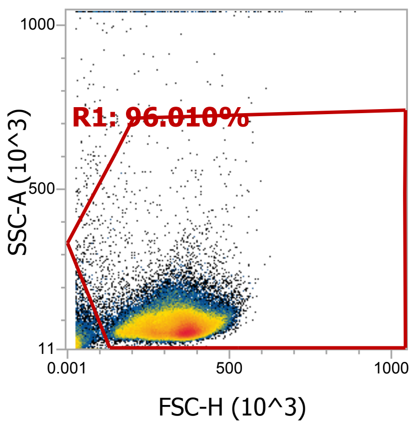

All Events - 3. Trypsin Cells+SEQ2 Cy5

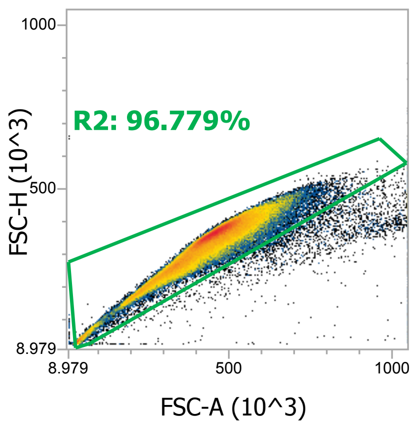

R2 - 3. Trypsin Cells+SEQ2 Cy5

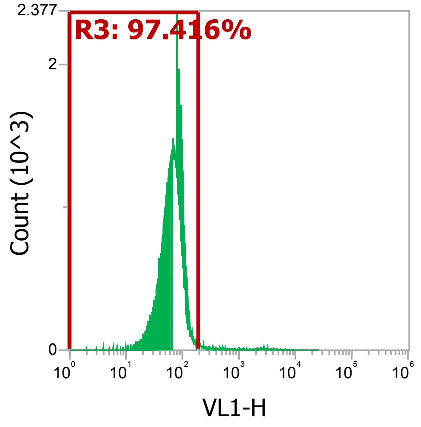

R3 - 3. Trypsin Cells+SEQ2 Cy5

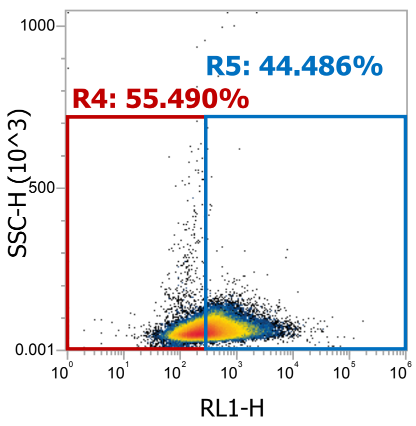

Experiment: **20210910**
Sample: **3. Trypsin Cells+SEQ2 Cy5**
Time Recorded: **12:44:21**

Name	Gate	X Parameter	Y Parameter	Count	%Total	%Gated
All Events	All Events	N/A	N/A	92,448	100.000	100.000
R1	R1	FSC - FSC-H	SSC - SSC-A	88,759	96.010	96.010
R2	R2	FSC - FSC-A	FSC - FSC-H	89,470	96.779	96.779
R3	R3	VL1 - VL1-H		87,158	94.278	97.416
R4	R4	RL1 - RL1-H	SSC - SSC-H	48,364	52.315	55.490
R5	R5	RL1 - RL1-H	SSC - SSC-H	38,773	41.940	44.486

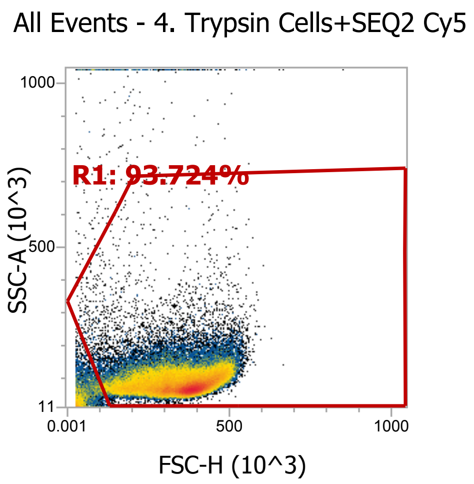

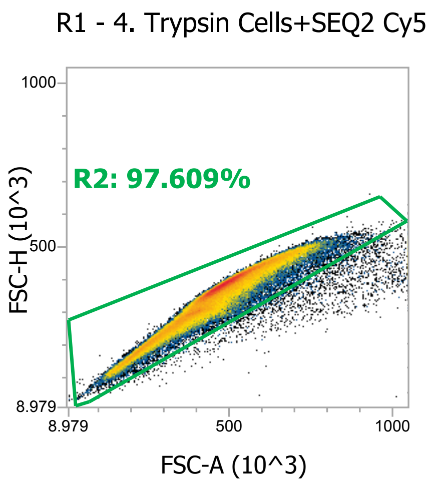

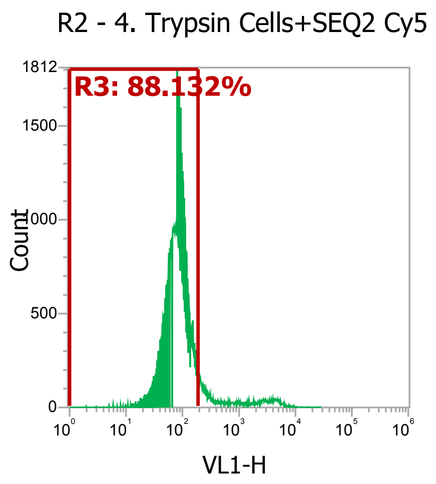

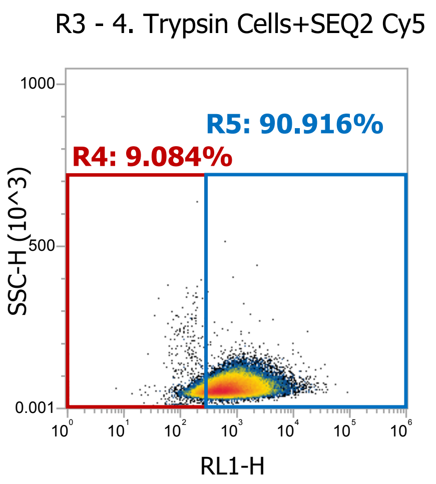

Experiment: **20210917**
Sample: **4. Trypsin Cells+SEQ2 Cy5**
Time Recorded: **11:35:33**

Name	Gate	X Parameter	Y Parameter	Count	%Total	%Gated
All Events	All Events	N/A	N/A	91,256	100.000	100.000
R1	R1	FSC - FSC-H	SSC - SSC-A	85,529	93.724	93.724
R2	R2	FSC - FSC-A	FSC - FSC-H	83,484	91.483	97.609
R3	R3	VL1 - VL1-H		73,576	80.626	88.132
R4	R4	RL1 - RL1-H	SSC - SSC-H	6,684	7.324	9.084
R5	R5	RL1 - RL1-H	SSC - SSC-H	66,892	73.301	90.916
