# Supplementary material for: A retrotransposon-derived DNA zip code internalizes myeloma cells through Clathrin-Rab5a-mediated endocytosis
Source: Front Oncol. 2024 Feb 23;14:1288724. doi: 10.3389/fonc.2024.1288724 (PMC10920344; doi:10.3389/fonc.2024.1288724)
Supplement: Supplementary file 12 [file DataSheet_1.zip › Raw data/Fig 1C, MM1S Timepoints/2hr NT Cells+MMZC Cy5.pdf]

All Events - 2. NT Cells+SEQ2 Cy5

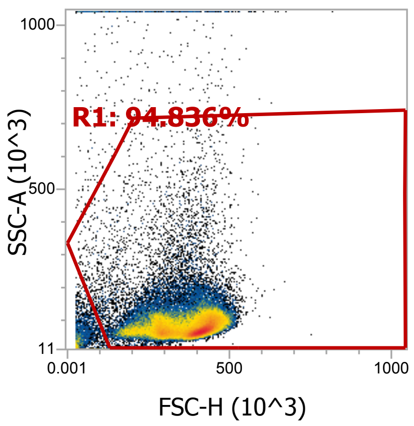

R1 - 2. NT Cells+SEQ2 Cy5

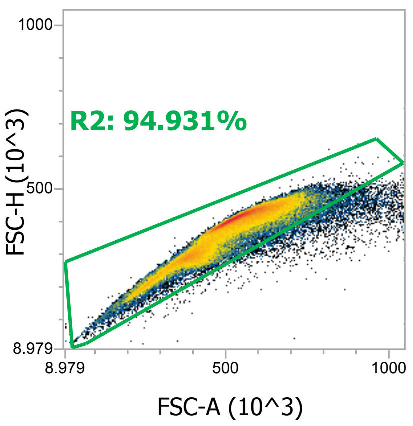

R2 - 2. NT Cells+SEQ2 Cy5

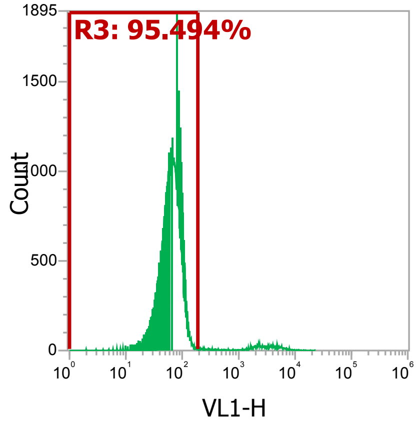

R3 - 2. NT Cells+SEQ2 Cy5

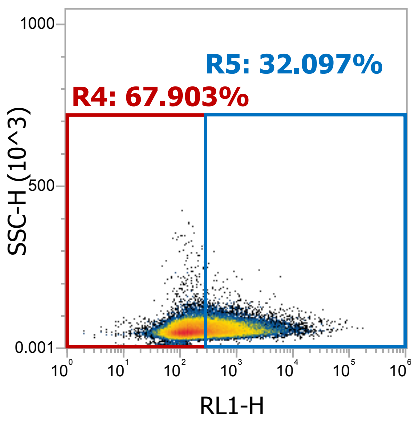

Experiment: **20210901**
Sample: **2. NT Cells+SEQ2 Cy5**
Time Recorded: **10:31:16**

Name	Gate	X Parameter	Y Parameter	Count	%Total	%Gated
All Events	All Events	N/A	N/A	75,214	100.000	100.000
R1	R1	FSC - FSC-H	SSC - SSC-A	71,330	94.836	94.836
R2	R2	FSC - FSC-A	FSC - FSC-H	67,714	90.028	94.931
R3	R3	VL1 - VL1-H		64,663	85.972	95.494
R4	R4	RL1 - RL1-H	SSC - SSC-H	43,908	58.377	67.903
R5	R5	RL1 - RL1-H	SSC - SSC-H	20,755	27.595	32.097

All Events - 3. NT Cells+SEQ2 Cy5

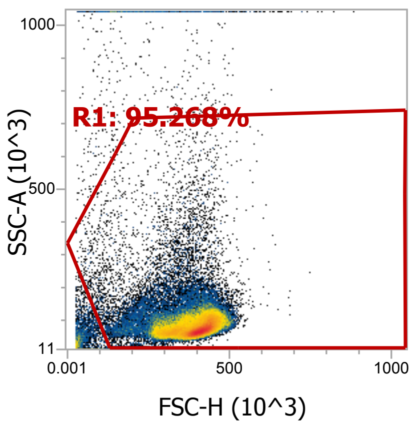

All Events - 3. NT Cells+SEQ2 Cy5

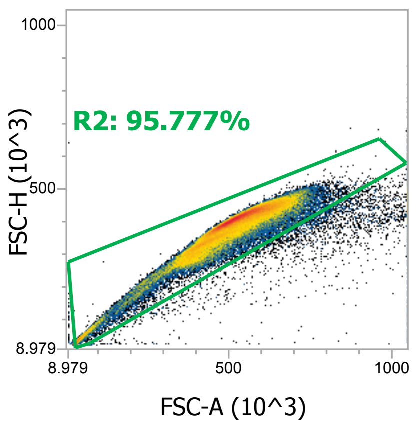

R2 - 3. NT Cells+SEQ2 Cy5

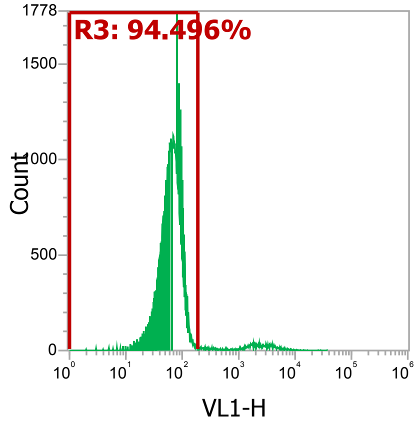

R3 - 3. NT Cells+SEQ2 Cy5

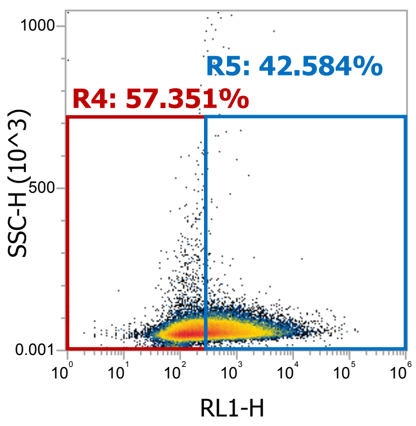

Experiment: **20210903**
Sample: **3. NT Cells+SEQ2 Cy5**
Time Recorded: **12:31:47**

Name	Gate	X Parameter	Y Parameter	Count	%Total	%Gated
All Events	All Events	N/A	N/A	73,027	100.000	100.000
R1	R1	FSC - FSC-H	SSC - SSC-A	69,571	95.268	95.268
R2	R2	FSC - FSC-A	FSC - FSC-H	69,943	95.777	95.777
R3	R3	VL1 - VL1-H		66,093	90.505	94.496
R4	R4	RL1 - RL1-H	SSC - SSC-H	37,905	51.905	57.351
R5	R5	RL1 - RL1-H	SSC - SSC-H	28,145	38.541	42.584

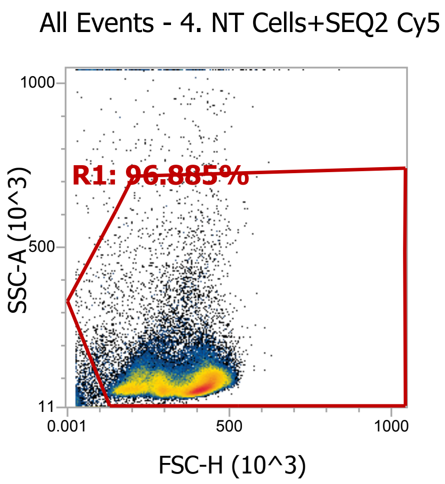

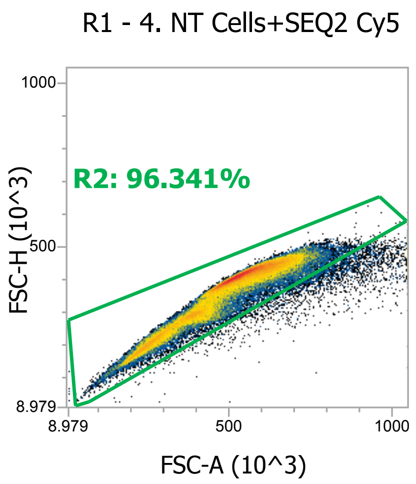

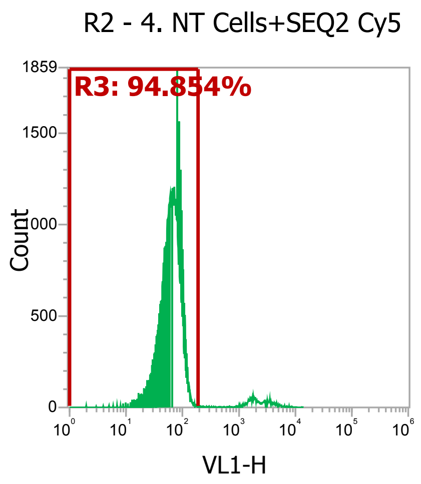

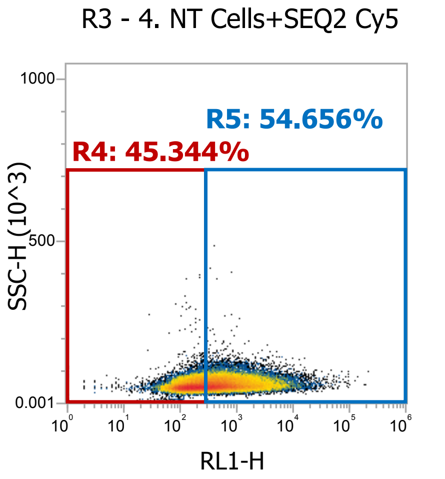

Experiment: **20210908**

Sample: **4. NT Cells+SEQ2 Cy5**

Time Recorded: **11:53:00**

Name	Gate	X Parameter	Y Parameter	Count	%Total	%Gated
All Events	All Events	N/A	N/A	78,172	100.000	100.000
R1	R1	FSC - FSC-H	SSC - SSC-A	75,737	96.885	96.885
R2	R2	FSC - FSC-A	FSC - FSC-H	72,966	93.340	96.341
R3	R3	VL1 - VL1-H		69,211	88.537	94.854
R4	R4	RL1 - RL1-H	SSC - SSC-H	31,383	40.146	45.344
R5	R5	RL1 - RL1-H	SSC - SSC-H	37,828	48.391	54.656

All Events - 5. NT Cells+SEQ2 Cy5

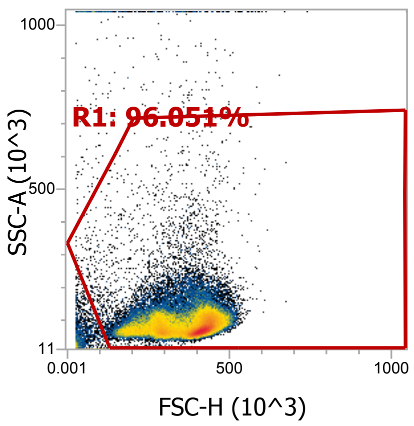

R1 - 5. NT Cells+SEQ2 Cy5

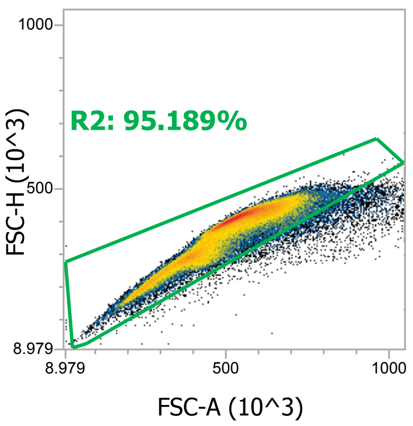

R2 - 5. NT Cells+SEQ2 Cy5

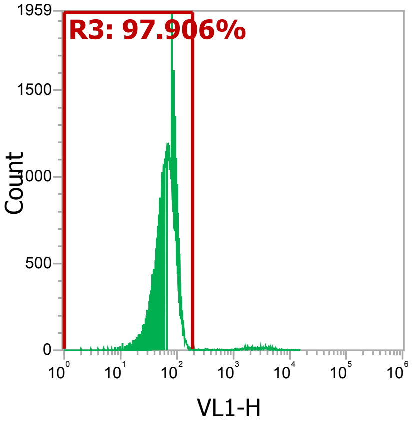

R3 - 5. NT Cells+SEQ2 Cy5

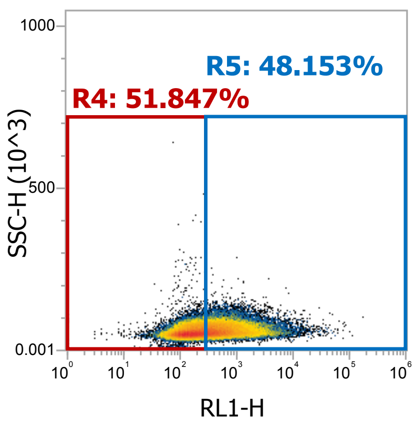

Experiment: **20210917**
Sample: **5. NT Cells+SEQ2 Cy5**
Time Recorded: **09:16:10**

Name	Gate	X Parameter	Y Parameter	Count	%Total	%Gated
All Events	All Events	N/A	N/A	76,199	100.000	100.000
R1	R1	FSC - FSC-H	SSC - SSC-A	73,190	96.051	96.051
R2	R2	FSC - FSC-A	FSC - FSC-H	69,669	91.430	95.189
R3	R3	VL1 - VL1-H		68,210	89.516	97.906
R4	R4	RL1 - RL1-H	SSC - SSC-H	35,365	46.411	51.847
R5	R5	RL1 - RL1-H	SSC - SSC-H	32,845	43.104	48.153

All Events - 6. NT Cells+SEQ2 Cy5

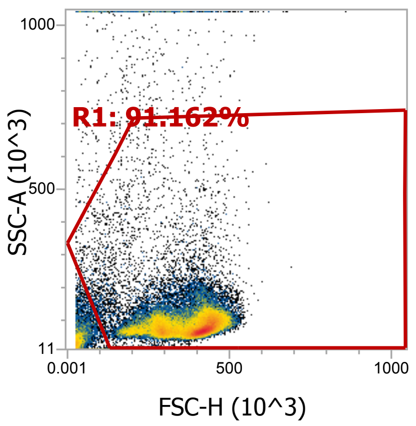

R1 - 6. NT Cells+SEQ2 Cy5

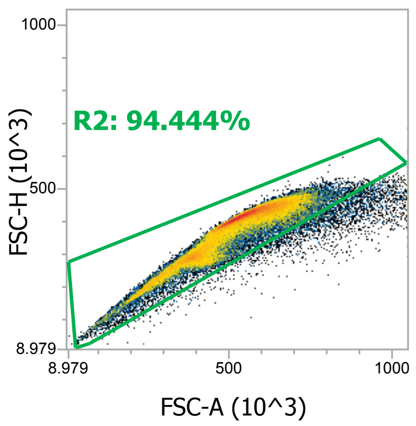

R2 - 6. NT Cells+SEQ2 Cy5

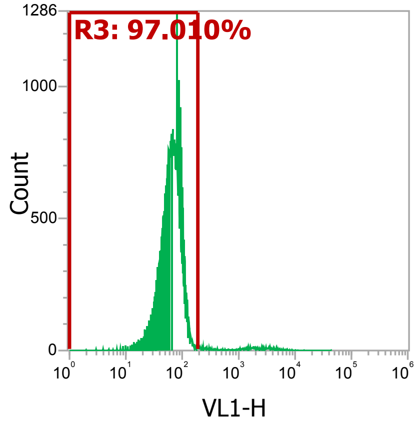

R3 - 6. NT Cells+SEQ2 Cy5

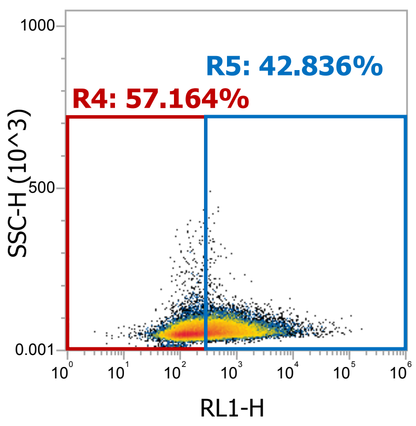

Experiment: **20210923**
Sample: **6. NT Cells+SEQ2 Cy5**
Time Recorded: **13:05:09**

Name	Gate	X Parameter	Y Parameter	Count	%Total	%Gated
All Events	All Events	N/A	N/A	56,800	100.000	100.000
R1	R1	FSC - FSC-H	SSC - SSC-A	51,780	91.162	91.162
R2	R2	FSC - FSC-A	FSC - FSC-H	48,903	86.097	94.444
R3	R3	VL1 - VL1-H		47,441	83.523	97.010
R4	R4	RL1 - RL1-H	SSC - SSC-H	27,119	47.745	57.164
R5	R5	RL1 - RL1-H	SSC - SSC-H	20,322	35.778	42.836
