# Supplementary material for: A retrotransposon-derived DNA zip code internalizes myeloma cells through Clathrin-Rab5a-mediated endocytosis
Source: Front Oncol. 2024 Feb 23;14:1288724. doi: 10.3389/fonc.2024.1288724 (PMC10920344; doi:10.3389/fonc.2024.1288724)
Supplement: Supplementary file 12 [file DataSheet_1.zip › Raw data/Fig 1C, MM1S Timepoints/2hr Trypsin Cells+MMZC Cy5.pdf]

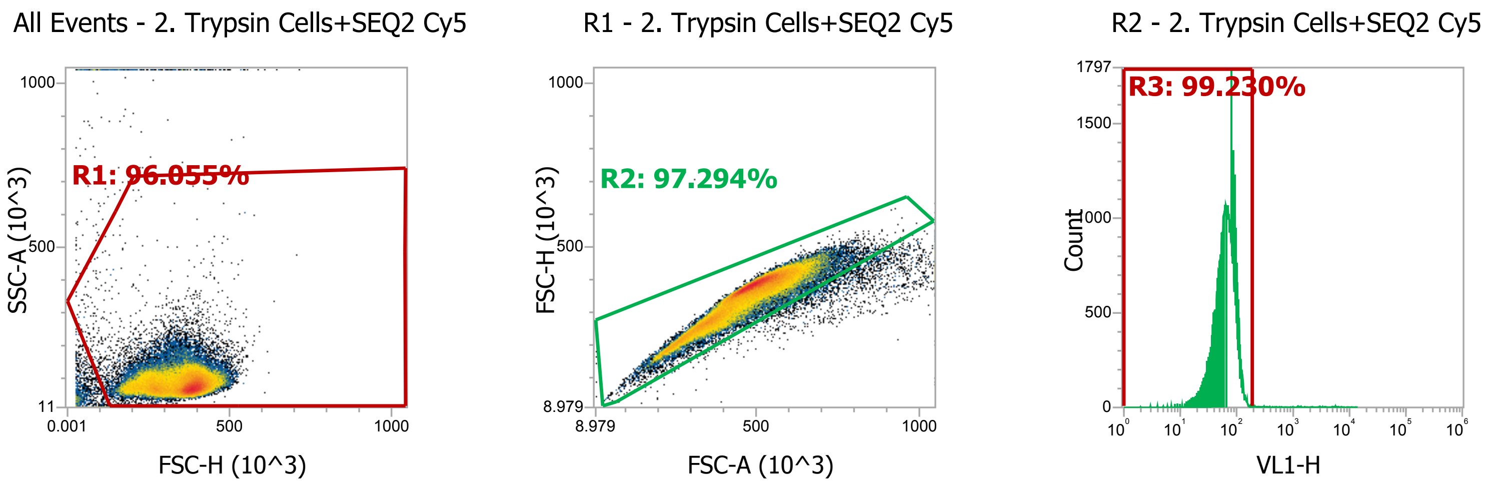

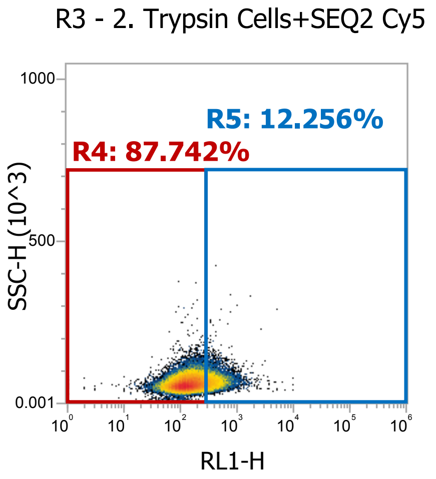

Experiment: **20210901**
Sample: **2. Trypsin Cells+SEQ2 Cy5**
Time Recorded: **10:29:31**

Name	Gate	X Parameter	Y Parameter	Count	%Total	%Gated
All Events	All Events	N/A	N/A	67,131	100.000	100.000
R1	R1	FSC - FSC-H	SSC - SSC-A	64,483	96.055	96.055
R2	R2	FSC - FSC-A	FSC - FSC-H	62,738	93.456	97.294
R3	R3	VL1 - VL1-H		62,255	92.737	99.230
R4	R4	RL1 - RL1-H	SSC - SSC-H	54,624	81.369	87.742
R5	R5	RL1 - RL1-H	SSC - SSC-H	7,630	11.366	12.256

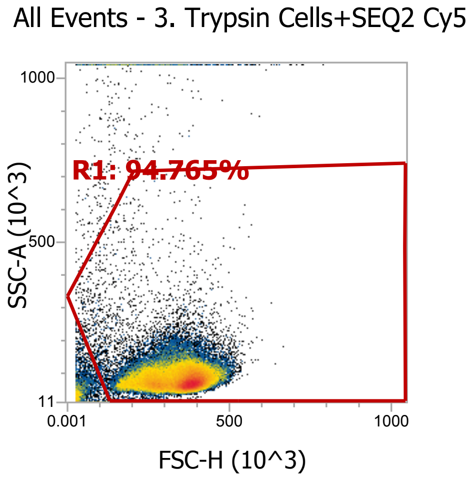

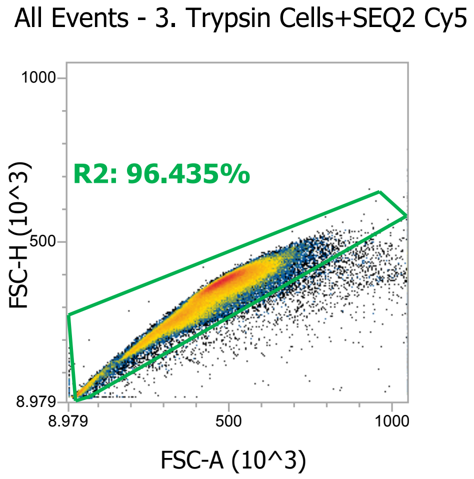

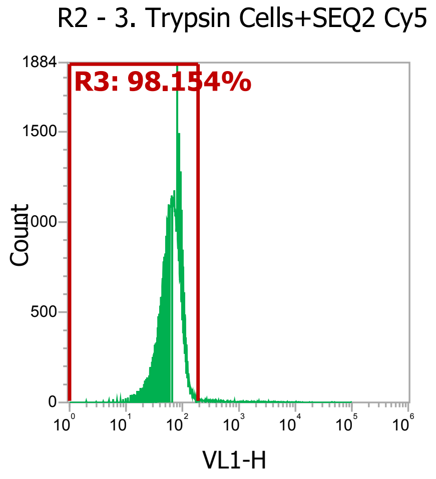

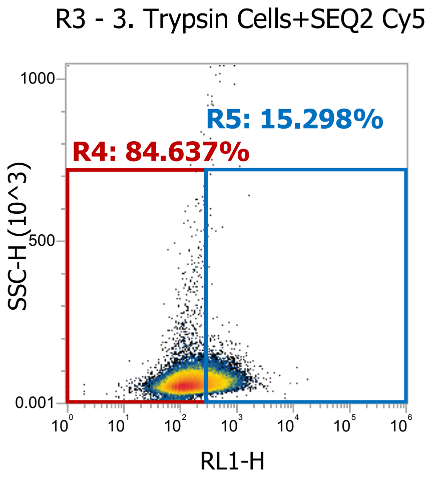

Experiment: **20210903**
Sample: **3. Trypsin Cells+SEQ2 Cy5**
Time Recorded: **12:30:15**

Name	Gate	X Parameter	Y Parameter	Count	%Total	%Gated
All Events	All Events	N/A	N/A	71,808	100.000	100.000
R1	R1	FSC - FSC-H	SSC - SSC-A	68,049	94.765	94.765
R2	R2	FSC - FSC-A	FSC - FSC-H	69,248	96.435	96.435
R3	R3	VL1 - VL1-H		67,970	94.655	98.154
R4	R4	RL1 - RL1-H	SSC - SSC-H	57,528	80.114	84.637
R5	R5	RL1 - RL1-H	SSC - SSC-H	10,398	14.480	15.298

All Events - 4. Trypsin Cells+SEQ2 Cy5

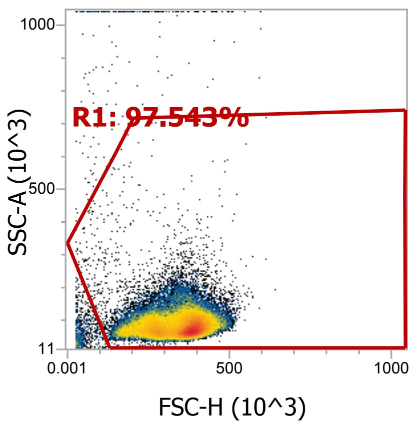

R1 - 4. Trypsin Cells+SEQ2 Cy5

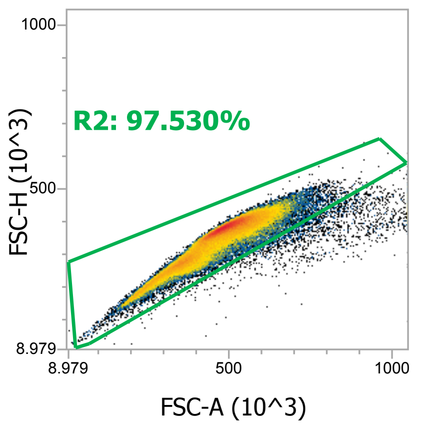

R2 - 4. Trypsin Cells+SEQ2 Cy5

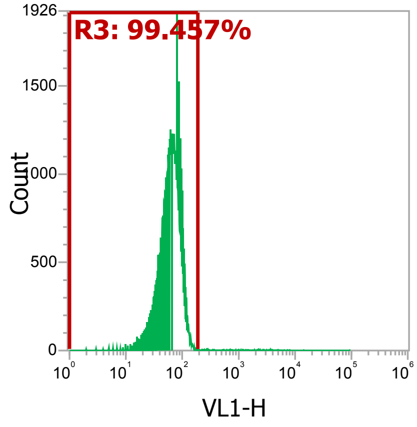

R3 - 4. Trypsin Cells+SEQ2 Cy5

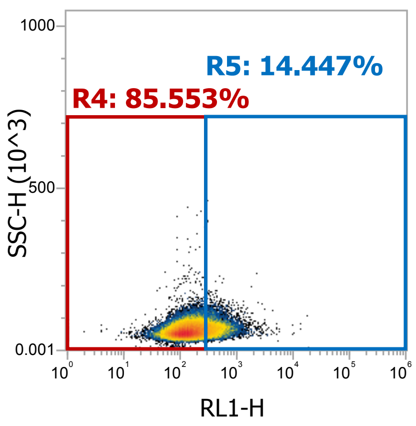

Experiment: **20210908**
Sample: **4. Trypsin Cells+SEQ2 Cy5**
Time Recorded: **11:47:44**

Name	Gate	X Parameter	Y Parameter	Count	%Total	%Gated
All Events	All Events	N/A	N/A	74,289	100.000	100.000
R1	R1	FSC - FSC-H	SSC - SSC-A	72,464	97.543	97.543
R2	R2	FSC - FSC-A	FSC - FSC-H	70,674	95.134	97.530
R3	R3	VL1 - VL1-H		70,290	94.617	99.457
R4	R4	RL1 - RL1-H	SSC - SSC-H	60,135	80.947	85.553
R5	R5	RL1 - RL1-H	SSC - SSC-H	10,155	13.670	14.447

All Events - 5. Trypsin Cells+SEQ2 Cy5

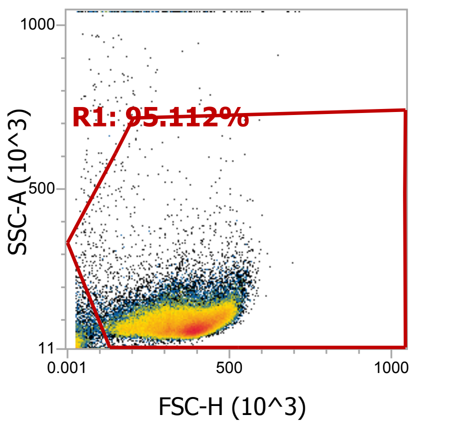

R1 - 5. Trypsin Cells+SEQ2 Cy5

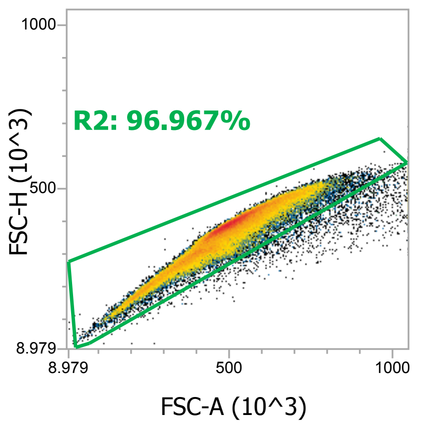

R2 - 5. Trypsin Cells+SEQ2 Cy5

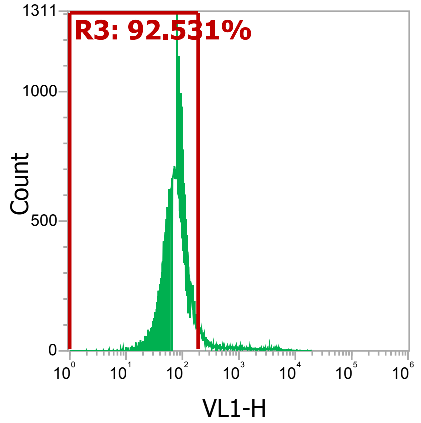

R3 - 5. Trypsin Cells+SEQ2 Cy5

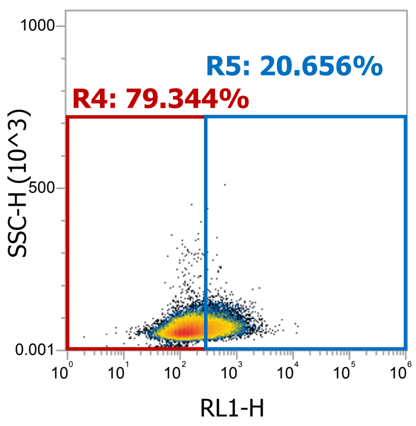

Experiment: **20210917**
Sample: **5. Trypsin Cells+SEQ2 Cy5**
Time Recorded: **09:14:45**

Name	Gate	X Parameter	Y Parameter	Count	%Total	%Gated
All Events	All Events	N/A	N/A	57,920	100.000	100.000
R1	R1	FSC - FSC-H	SSC - SSC-A	55,089	95.112	95.112
R2	R2	FSC - FSC-A	FSC - FSC-H	53,418	92.227	96.967
R3	R3	VL1 - VL1-H		49,428	85.338	92.531
R4	R4	RL1 - RL1-H	SSC - SSC-H	39,218	67.711	79.344
R5	R5	RL1 - RL1-H	SSC - SSC-H	10,210	17.628	20.656

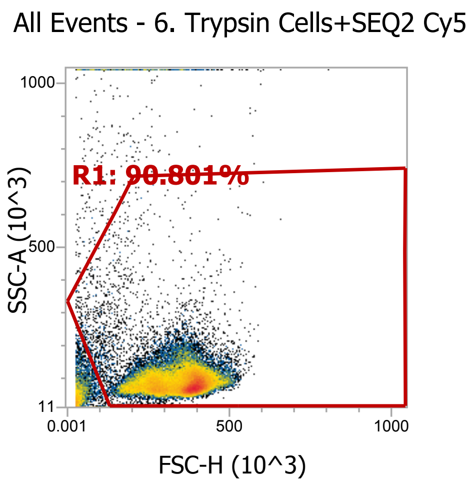

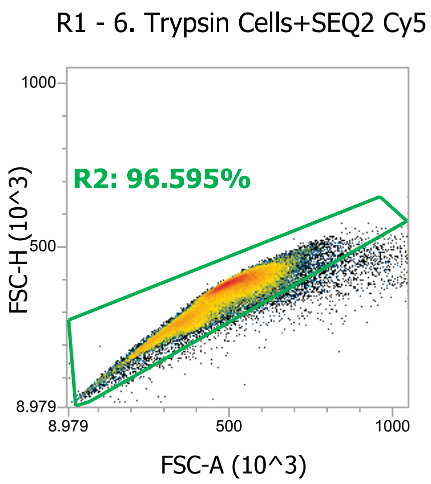

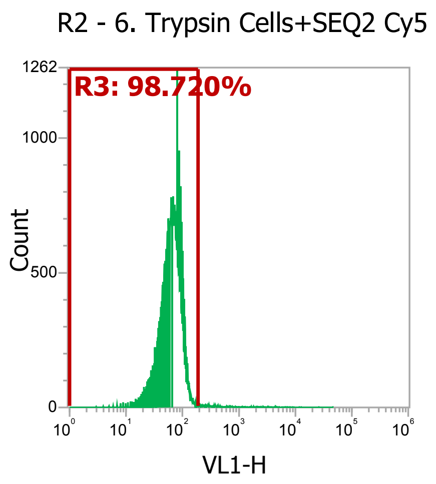

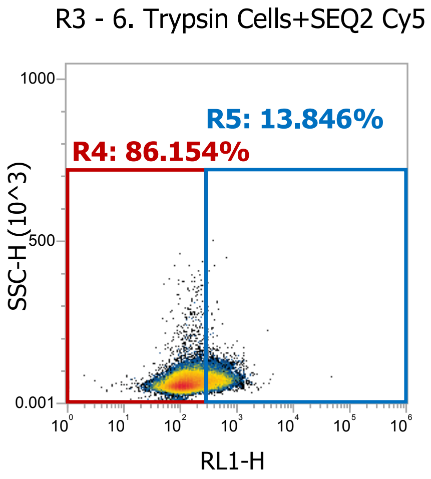

Experiment: **20210923**
Sample: **6. Trypsin Cells+SEQ2 Cy5**
Time Recorded: **13:03:28**

Name	Gate	X Parameter	Y Parameter	Count	%Total	%Gated
All Events	All Events	N/A	N/A	50,397	100.000	100.000
R1	R1	FSC - FSC-H	SSC - SSC-A	45,761	90.801	90.801
R2	R2	FSC - FSC-A	FSC - FSC-H	44,203	87.710	96.595
R3	R3	VL1 - VL1-H		43,637	86.587	98.720
R4	R4	RL1 - RL1-H	SSC - SSC-H	37,595	74.598	86.154
R5	R5	RL1 - RL1-H	SSC - SSC-H	6,042	11.989	13.846
