# Supplementary material for: A retrotransposon-derived DNA zip code internalizes myeloma cells through Clathrin-Rab5a-mediated endocytosis
Source: Front Oncol. 2024 Feb 23;14:1288724. doi: 10.3389/fonc.2024.1288724 (PMC10920344; doi:10.3389/fonc.2024.1288724)
Supplement: Supplementary file 12 [file DataSheet_1.zip › Raw data/Fig 1C, MM1S Timepoints/4hr NT Cells+MMZC Cy5.pdf]

All Events - 2. NT Cells+SEQ2 Cy5

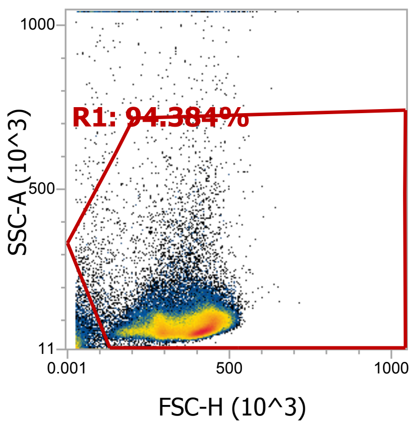

R1 - 2. NT Cells+SEQ2 Cy5

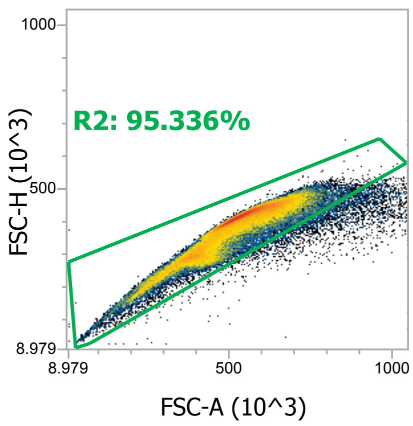

R2 - 2. NT Cells+SEQ2 Cy5

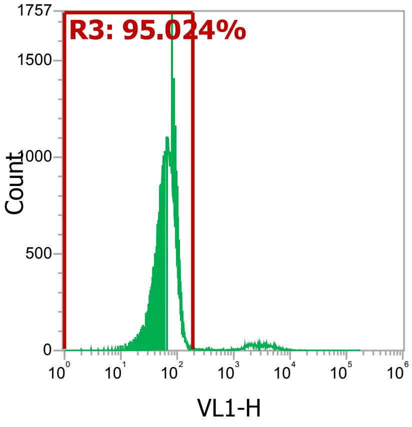

R3 - 2. NT Cells+SEQ2 Cy5

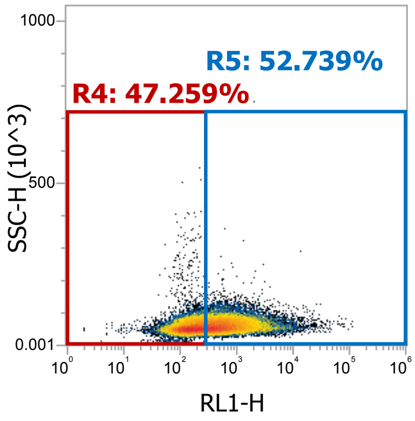

Experiment: **20210901**
Sample: **2. NT Cells+SEQ2 Cy5**
Time Recorded: **12:28:10**

Name	Gate	X Parameter	Y Parameter	Count	%Total	%Gated
All Events	All Events	N/A	N/A	73,739	100.000	100.000
R1	R1	FSC - FSC-H	SSC - SSC-A	69,598	94.384	94.384
R2	R2	FSC - FSC-A	FSC - FSC-H	66,352	89.982	95.336
R3	R3	VL1 - VL1-H		63,050	85.504	95.024
R4	R4	RL1 - RL1-H	SSC - SSC-H	29,797	40.409	47.259
R5	R5	RL1 - RL1-H	SSC - SSC-H	33,252	45.094	52.739

All Events - 3. NT Cells+SEQ2 Cy5

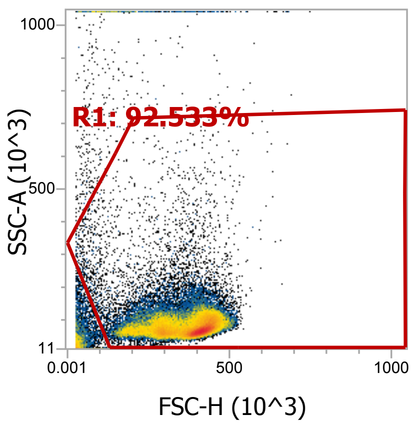

All Events - 3. NT Cells+SEQ2 Cy5

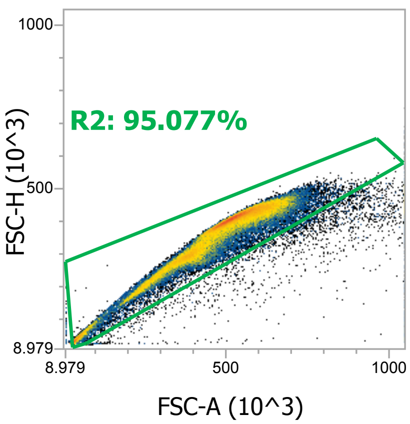

R2 - 3. NT Cells+SEQ2 Cy5

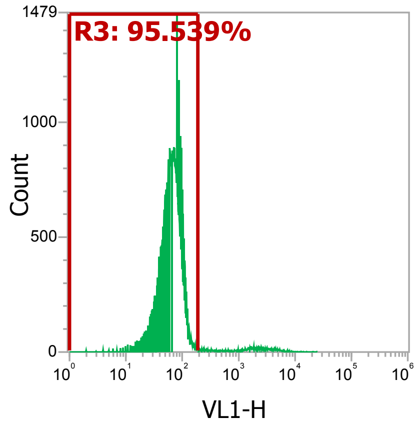

R3 - 3. NT Cells+SEQ2 Cy5

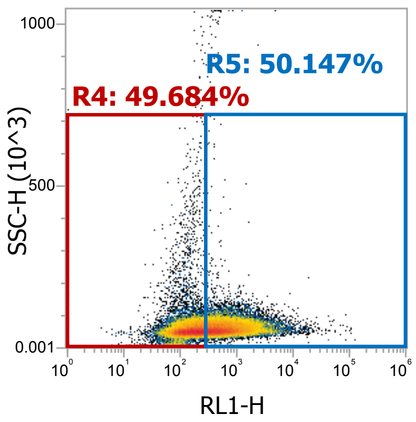

Experiment: **20210903**
Sample: **3. NT Cells+SEQ2 Cy5**
Time Recorded: **14:18:45**

Name	Gate	X Parameter	Y Parameter	Count	%Total	%Gated
All Events	All Events	N/A	N/A	57,952	100.000	100.000
R1	R1	FSC - FSC-H	SSC - SSC-A	53,625	92.533	92.533
R2	R2	FSC - FSC-A	FSC - FSC-H	55,099	95.077	95.077
R3	R3	VL1 - VL1-H		52,641	90.836	95.539
R4	R4	RL1 - RL1-H	SSC - SSC-H	26,154	45.130	49.684
R5	R5	RL1 - RL1-H	SSC - SSC-H	26,398	45.551	50.147

All Events - 4. NT Cells+SEQ2 Cy5

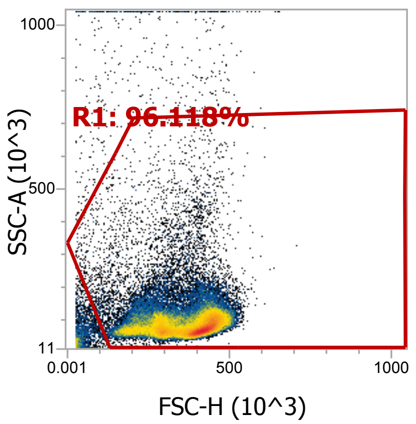

R1 - 4. NT Cells+SEQ2 Cy5

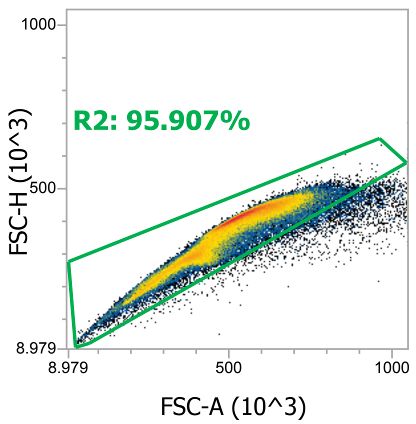

R2 - 4. NT Cells+SEQ2 Cy5

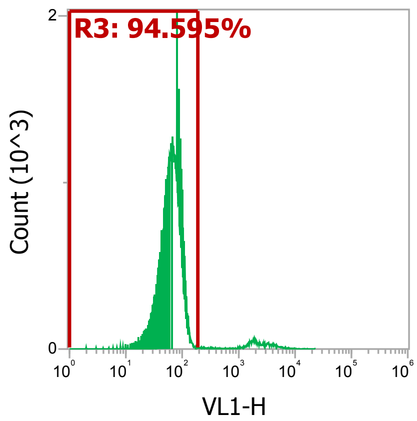

R3 - 4. NT Cells+SEQ2 Cy5

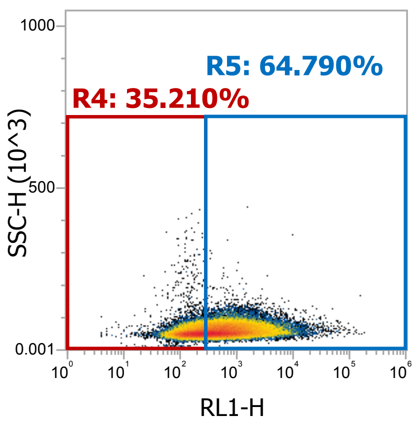

Experiment: **20210908**
Sample: **4. NT Cells+SEQ2 Cy5**
Time Recorded: **13:45:09**

Name	Gate	X Parameter	Y Parameter	Count	%Total	%Gated
All Events	All Events	N/A	N/A	81,945	100.000	100.000
R1	R1	FSC - FSC-H	SSC - SSC-A	78,764	96.118	96.118
R2	R2	FSC - FSC-A	FSC - FSC-H	75,540	92.184	95.907
R3	R3	VL1 - VL1-H		71,457	87.201	94.595
R4	R4	RL1 - RL1-H	SSC - SSC-H	25,160	30.704	35.210
R5	R5	RL1 - RL1-H	SSC - SSC-H	46,297	56.498	64.790

All Events - 5. NT Cells+SEQ2 Cy5

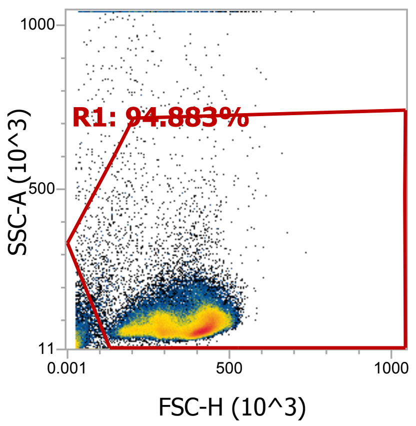

R1 - 5. NT Cells+SEQ2 Cy5

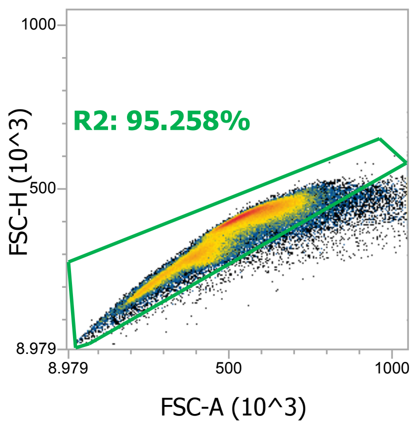

R2 - 5. NT Cells+SEQ2 Cy5

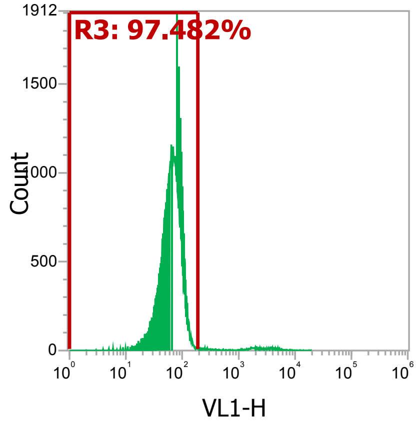

R3 - 5. NT Cells+SEQ2 Cy5

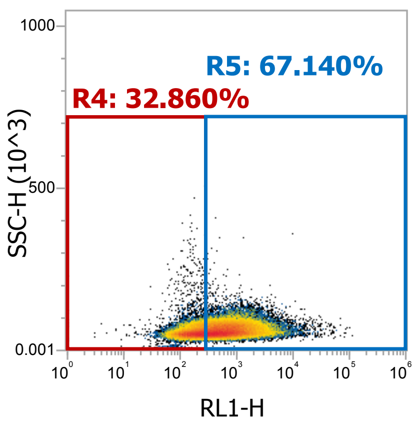

Experiment: **20210917**
Sample: **5. NT Cells+SEQ2 Cy5**
Time Recorded: **11:24:40**

Name	Gate	X Parameter	Y Parameter	Count	%Total	%Gated
All Events	All Events	N/A	N/A	74,791	100.000	100.000
R1	R1	FSC - FSC-H	SSC - SSC-A	70,964	94.883	94.883
R2	R2	FSC - FSC-A	FSC - FSC-H	67,599	90.384	95.258
R3	R3	VL1 - VL1-H		65,897	88.108	97.482
R4	R4	RL1 - RL1-H	SSC - SSC-H	21,654	28.953	32.860
R5	R5	RL1 - RL1-H	SSC - SSC-H	44,243	59.156	67.140

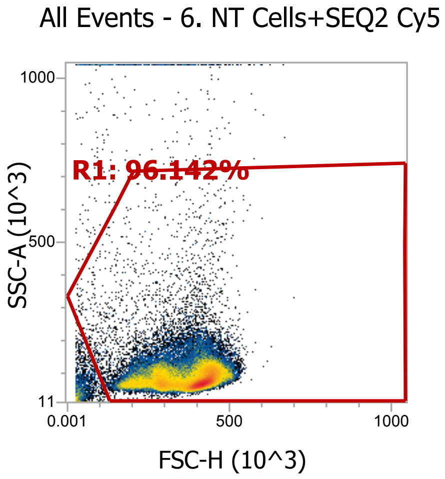

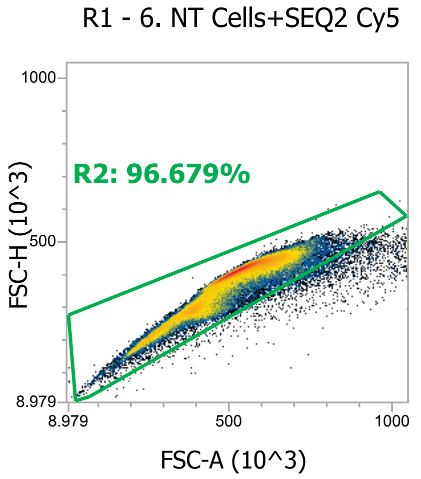

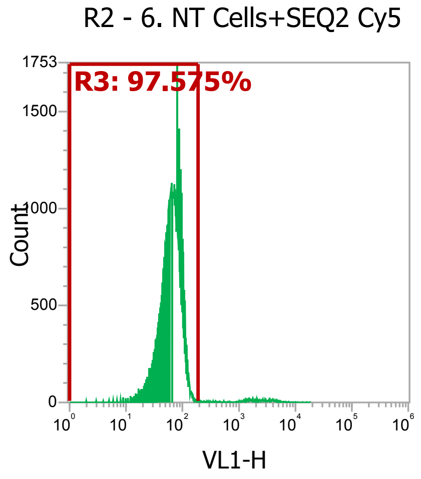

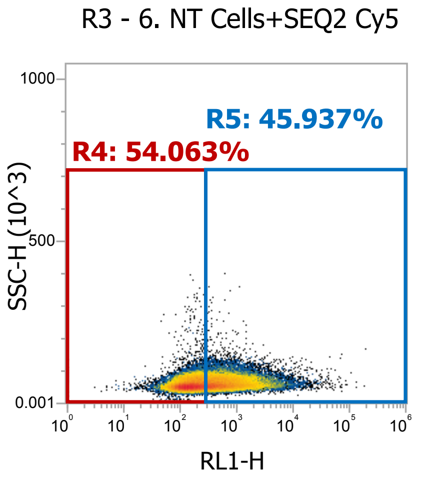

Experiment: **20210924**
Sample: **6. NT Cells+SEQ2 Cy5**
Time Recorded: **13:41:47**

Name	Gate	X Parameter	Y Parameter	Count	%Total	%Gated
All Events	All Events	N/A	N/A	71,039	100.000	100.000
R1	R1	FSC - FSC-H	SSC - SSC-A	68,298	96.142	96.142
R2	R2	FSC - FSC-A	FSC - FSC-H	66,030	92.949	96.679
R3	R3	VL1 - VL1-H		64,429	90.695	97.575
R4	R4	RL1 - RL1-H	SSC - SSC-H	34,832	49.032	54.063
R5	R5	RL1 - RL1-H	SSC - SSC-H	29,597	41.663	45.937
