# Supplementary material for: A retrotransposon-derived DNA zip code internalizes myeloma cells through Clathrin-Rab5a-mediated endocytosis
Source: Front Oncol. 2024 Feb 23;14:1288724. doi: 10.3389/fonc.2024.1288724 (PMC10920344; doi:10.3389/fonc.2024.1288724)
Supplement: Supplementary file 12 [file DataSheet_1.zip › Raw data/Fig 1C, MM1S Timepoints/4hr Trypsin Cells+MMZC Cy5.pdf]

All Events - 2. Trypsin Cells+SEQ2 Cy52

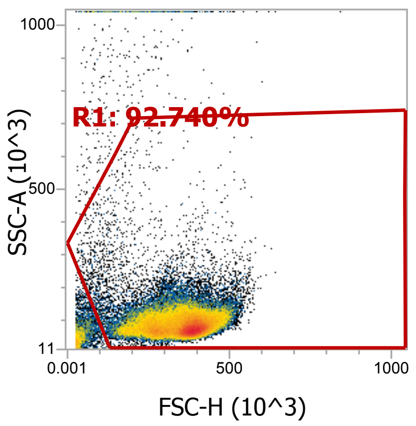

R1 - 2. Trypsin Cells+SEQ2 Cy52

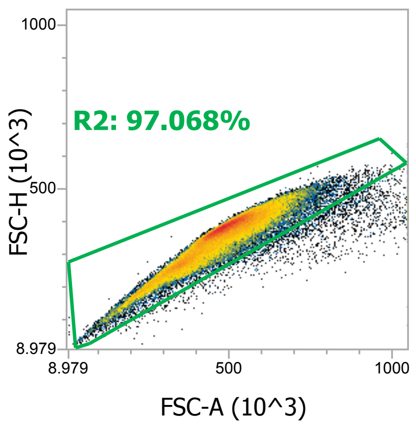

R2 - 2. Trypsin Cells+SEQ2 Cy52

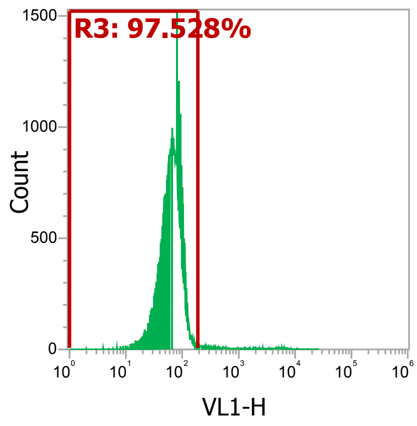

R3 - 2. Trypsin Cells+SEQ2 Cy52

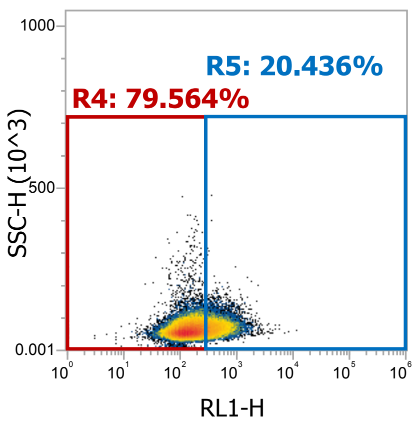

Experiment: **20210901**
Sample: **2. Trypsin Cells+SEQ2 Cy52**
Time Recorded: **12:26:22**

Name	Gate	X Parameter	Y Parameter	Count	%Total	%Gated
All Events	All Events	N/A	N/A	63,400	100.000	100.000
R1	R1	FSC - FSC-H	SSC - SSC-A	58,797	92.740	92.740
R2	R2	FSC - FSC-A	FSC - FSC-H	57,073	90.021	97.068
R3	R3	VL1 - VL1-H		55,662	87.795	97.528
R4	R4	RL1 - RL1-H	SSC - SSC-H	44,287	69.853	79.564
R5	R5	RL1 - RL1-H	SSC - SSC-H	11,375	17.942	20.436

All Events - 3. Trypsin Cells+SEQ2 Cy52

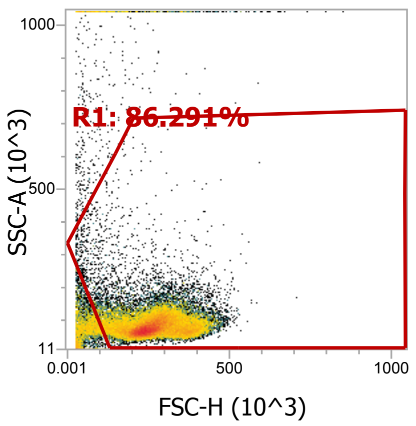

All Events - 3. Trypsin Cells+SEQ2 Cy52

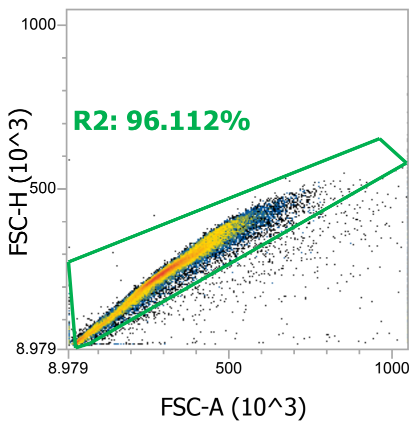

R2 - 3. Trypsin Cells+SEQ2 Cy52

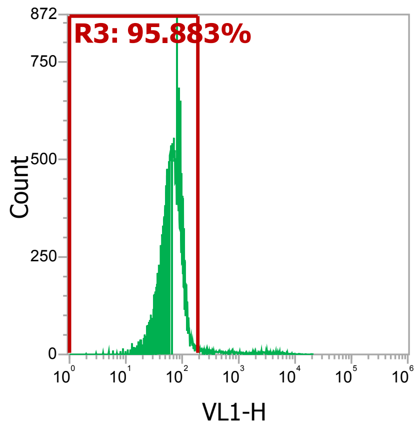

R3 - 3. Trypsin Cells+SEQ2 Cy52

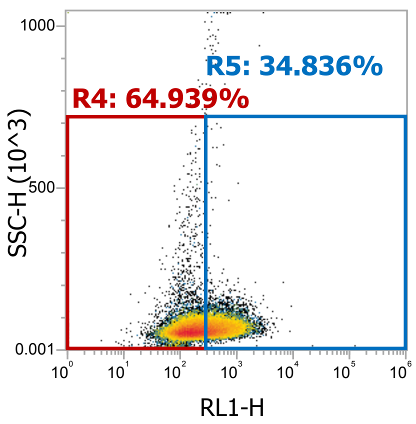

Experiment: **20210903**
Sample: **3. Trypsin Cells+SEQ2 Cy52**
Time Recorded: **14:16:36**

Name	Gate	X Parameter	Y Parameter	Count	%Total	%Gated
All Events	All Events	N/A	N/A	34,750	100.000	100.000
R1	R1	FSC - FSC-H	SSC - SSC-A	29,986	86.291	86.291
R2	R2	FSC - FSC-A	FSC - FSC-H	33,399	96.112	96.112
R3	R3	VL1 - VL1-H		32,024	92.155	95.883
R4	R4	RL1 - RL1-H	SSC - SSC-H	20,796	59.845	64.939
R5	R5	RL1 - RL1-H	SSC - SSC-H	11,156	32.104	34.836

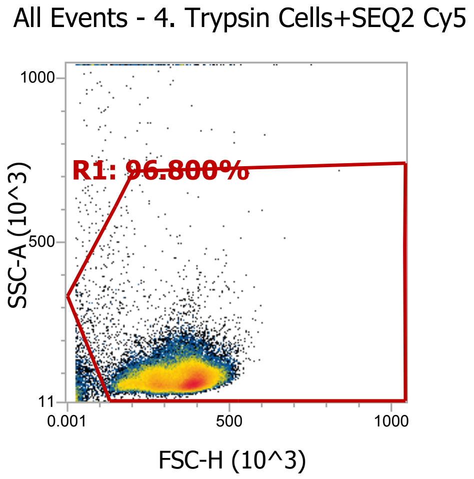

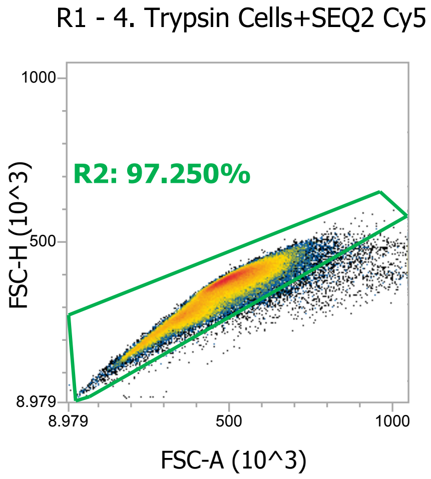

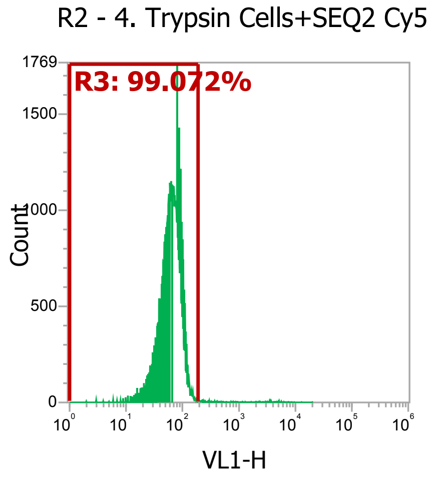

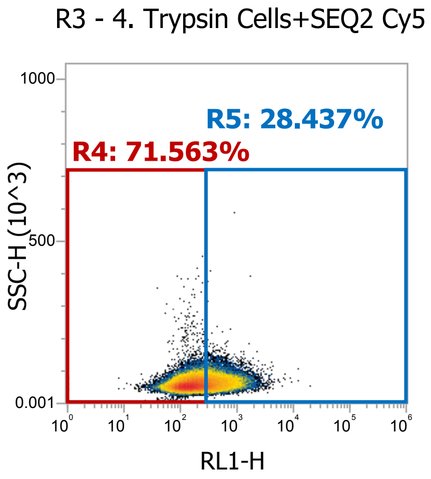

Experiment: **20210908**
Sample: **4. Trypsin Cells+SEQ2 Cy5**
Time Recorded: **13:42:33**

Name	Gate	X Parameter	Y Parameter	Count	%Total	%Gated
All Events	All Events	N/A	N/A	71,181	100.000	100.000
R1	R1	FSC - FSC-H	SSC - SSC-A	68,903	96.800	96.800
R2	R2	FSC - FSC-A	FSC - FSC-H	67,008	94.137	97.250
R3	R3	VL1 - VL1-H		66,386	93.264	99.072
R4	R4	RL1 - RL1-H	SSC - SSC-H	47,508	66.743	71.563
R5	R5	RL1 - RL1-H	SSC - SSC-H	18,878	26.521	28.437

All Events - 5. Trypsin Cells+SEQ2 Cy5

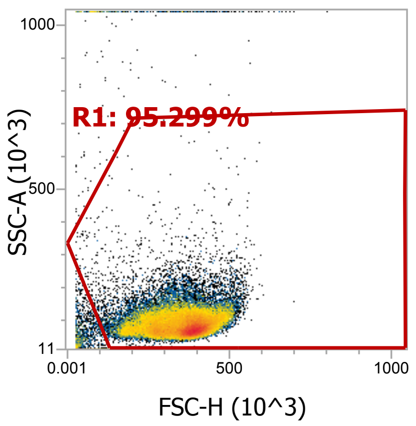

R1 - 5. Trypsin Cells+SEQ2 Cy5

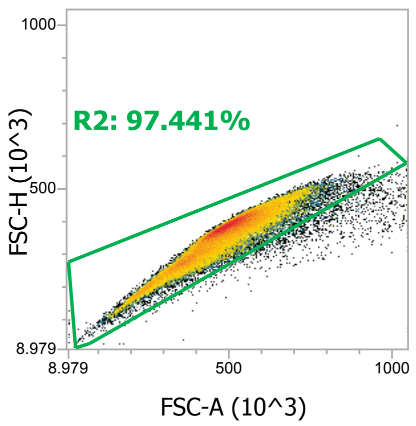

R2 - 5. Trypsin Cells+SEQ2 Cy5

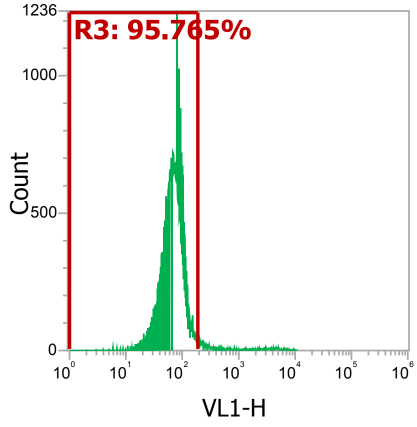

R3 - 5. Trypsin Cells+SEQ2 Cy5

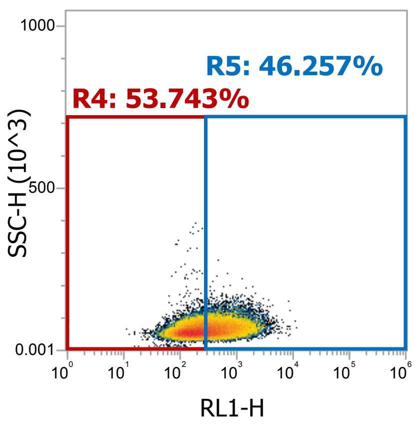

Experiment: **20210917**
Sample: **5. Trypsin Cells+SEQ2 Cy5**
Time Recorded: **11:23:08**

Name	Gate	X Parameter	Y Parameter	Count	%Total	%Gated
All Events	All Events	N/A	N/A	49,206	100.000	100.000
R1	R1	FSC - FSC-H	SSC - SSC-A	46,893	95.299	95.299
R2	R2	FSC - FSC-A	FSC - FSC-H	45,693	92.861	97.441
R3	R3	VL1 - VL1-H		43,758	88.928	95.765
R4	R4	RL1 - RL1-H	SSC - SSC-H	23,517	47.793	53.743
R5	R5	RL1 - RL1-H	SSC - SSC-H	20,241	41.135	46.257

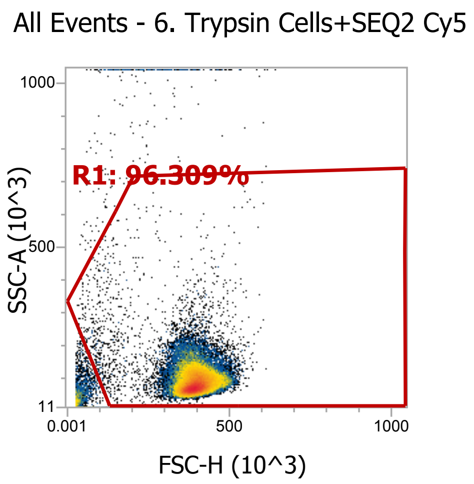

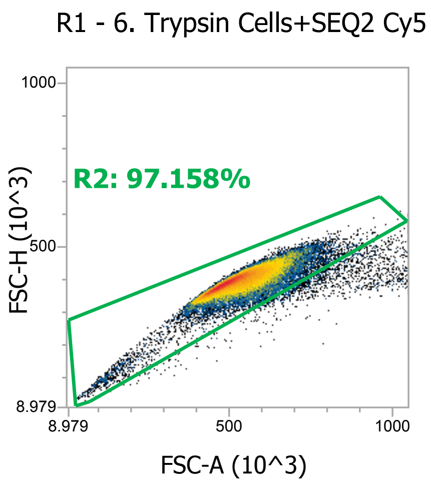

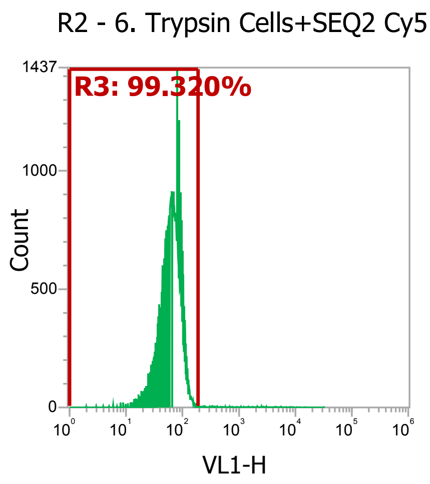

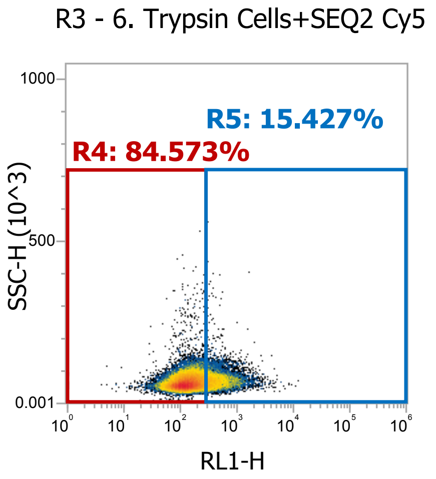

Experiment: **20210924**
Sample: **6. Trypsin Cells+SEQ2 Cy5**
Time Recorded: **13:40:16**

Name	Gate	X Parameter	Y Parameter	Count	%Total	%Gated
All Events	All Events	N/A	N/A	55,673	100.000	100.000
R1	R1	FSC - FSC-H	SSC - SSC-A	53,618	96.309	96.309
R2	R2	FSC - FSC-A	FSC - FSC-H	52,094	93.571	97.158
R3	R3	VL1 - VL1-H		51,740	92.936	99.320
R4	R4	RL1 - RL1-H	SSC - SSC-H	43,758	78.598	84.573
R5	R5	RL1 - RL1-H	SSC - SSC-H	7,982	14.337	15.427
