# Supplementary material for: A retrotransposon-derived DNA zip code internalizes myeloma cells through Clathrin-Rab5a-mediated endocytosis
Source: Front Oncol. 2024 Feb 23;14:1288724. doi: 10.3389/fonc.2024.1288724 (PMC10920344; doi:10.3389/fonc.2024.1288724)
Supplement: Supplementary file 12 [file DataSheet_1.zip › Raw data/Fig 1C, MM1S Timepoints/8hr NT Cells+MMZC Cy5.pdf]

All Events - 2. NT Cells+SEQ2 Cy5

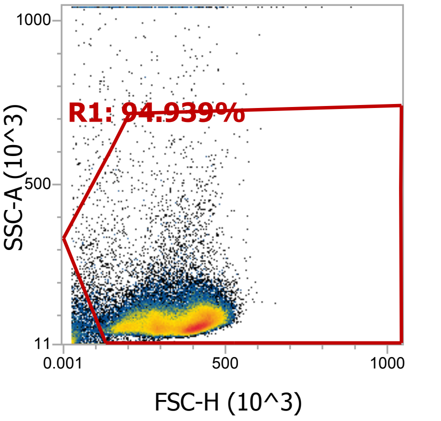

R1 - 2. NT Cells+SEQ2 Cy5

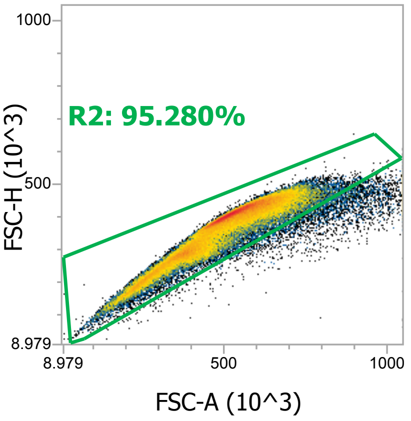

R2 - 2. NT Cells+SEQ2 Cy5

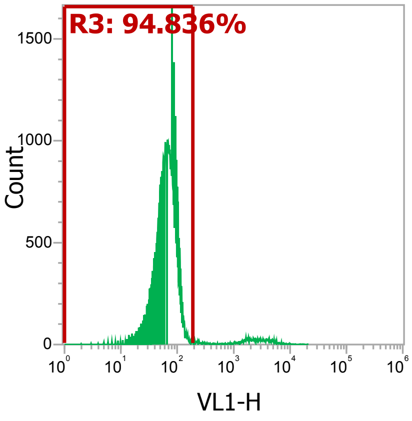

R3 - 2. NT Cells+SEQ2 Cy5

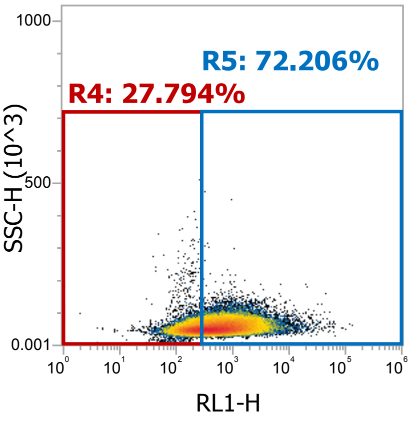

Experiment: **20210901**
Sample: **2. NT Cells+SEQ2 Cy5**
Time Recorded: **19:07:41**

Name	Gate	X Parameter	Y Parameter	Count	%Total	%Gated
All Events	All Events	N/A	N/A	68,845	100.000	100.000
R1	R1	FSC - FSC-H	SSC - SSC-A	65,361	94.939	94.939
R2	R2	FSC - FSC-A	FSC - FSC-H	62,276	90.458	95.280
R3	R3	VL1 - VL1-H		59,060	85.787	94.836
R4	R4	RL1 - RL1-H	SSC - SSC-H	16,415	23.843	27.794
R5	R5	RL1 - RL1-H	SSC - SSC-H	42,645	61.943	72.206

All Events - 3. NT Cells+SEQ2 Cy5

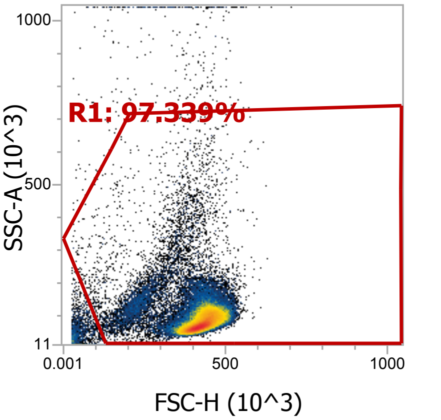

R1 - 3. NT Cells+SEQ2 Cy5

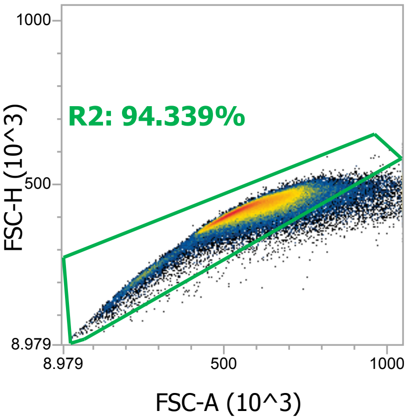

R2 - 3. NT Cells+SEQ2 Cy5

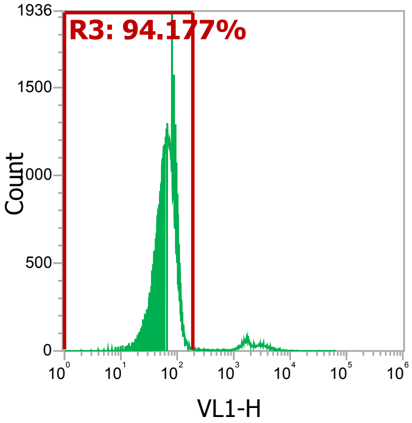

R3 - 3. NT Cells+SEQ2 Cy5

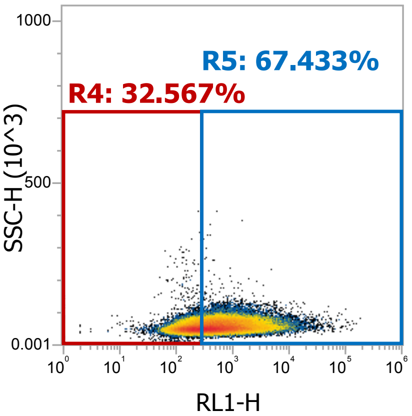

Experiment: **20210908**
Sample: **3. NT Cells+SEQ2 Cy5**
Time Recorded: **17:49:14**

Name	Gate	X Parameter	Y Parameter	Count	%Total	%Gated
All Events	All Events	N/A	N/A	83,009	100.000	100.000
R1	R1	FSC - FSC-H	SSC - SSC-A	80,800	97.339	97.339
R2	R2	FSC - FSC-A	FSC - FSC-H	76,226	91.829	94.339
R3	R3	VL1 - VL1-H		71,787	86.481	94.177
R4	R4	RL1 - RL1-H	SSC - SSC-H	23,379	28.164	32.567
R5	R5	RL1 - RL1-H	SSC - SSC-H	48,408	58.317	67.433

All Events - 4. NT Cells+SEQ2 Cy5

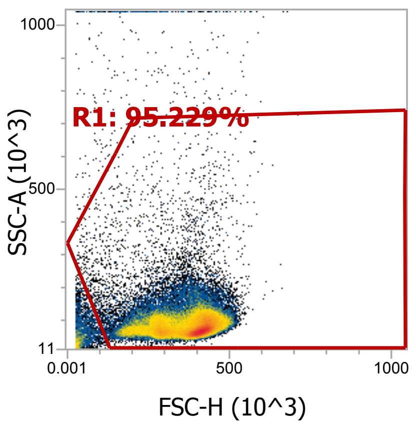

R1 - 4. NT Cells+SEQ2 Cy5

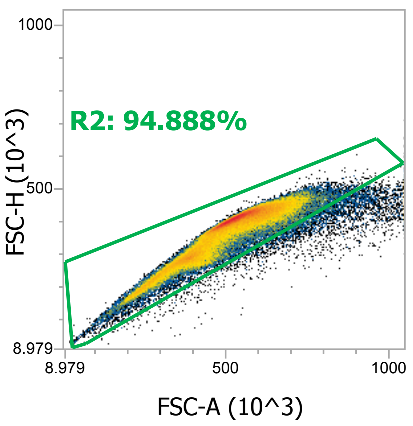

R2 - 4. NT Cells+SEQ2 Cy5

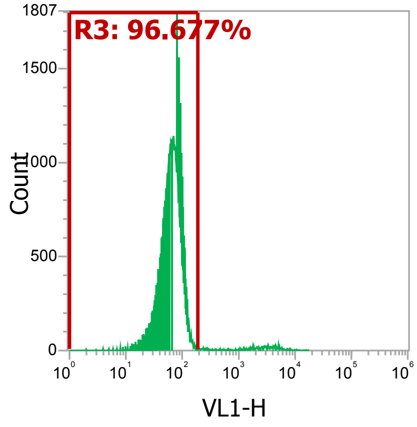

R3 - 4. NT Cells+SEQ2 Cy5

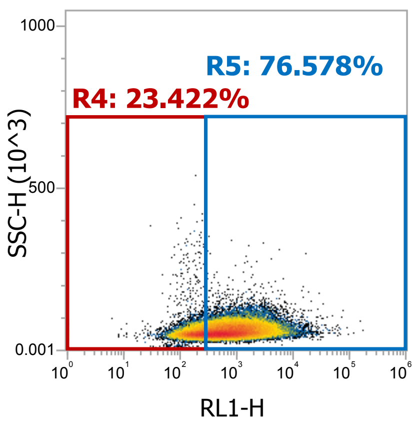

Experiment: **20210917**
Sample: **4. NT Cells+SEQ2 Cy5**
Time Recorded: **15:17:18**

Name	Gate	X Parameter	Y Parameter	Count	%Total	%Gated
All Events	All Events	N/A	N/A	76,188	100.000	100.000
R1	R1	FSC - FSC-H	SSC - SSC-A	72,553	95.229	95.229
R2	R2	FSC - FSC-A	FSC - FSC-H	68,844	90.361	94.888
R3	R3	VL1 - VL1-H		66,556	87.358	96.677
R4	R4	RL1 - RL1-H	SSC - SSC-H	15,589	20.461	23.422
R5	R5	RL1 - RL1-H	SSC - SSC-H	50,967	66.896	76.578
