# Supplementary material for: A retrotransposon-derived DNA zip code internalizes myeloma cells through Clathrin-Rab5a-mediated endocytosis
Source: Front Oncol. 2024 Feb 23;14:1288724. doi: 10.3389/fonc.2024.1288724 (PMC10920344; doi:10.3389/fonc.2024.1288724)
Supplement: Supplementary file 12 [file DataSheet_1.zip › Raw data/Fig 1C, MM1S Timepoints/8hr Trypsin Cells+MMZC Cy5.pdf]

All Events - 2. Trypsin Cells+SEQ2 Cy5

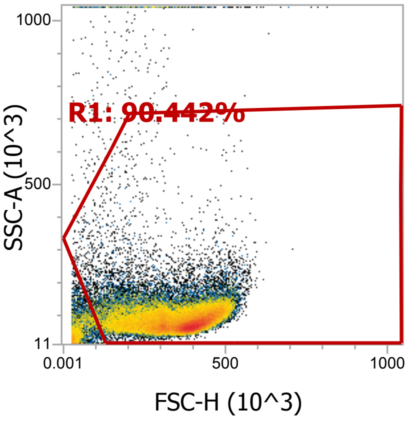


R1 - 2. Trypsin Cells+SEQ2 Cy5

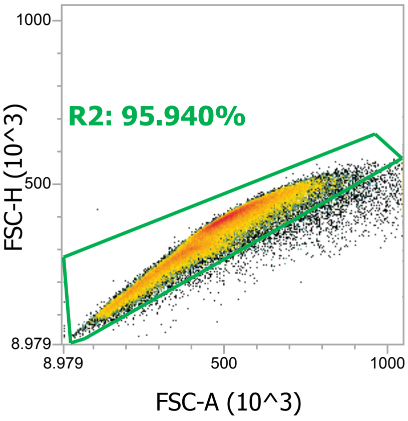


R2 - 2. Trypsin Cells+SEQ2 Cy5

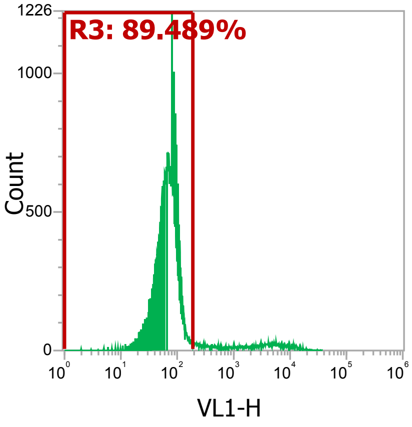


R3 - 2. Trypsin Cells+SEQ2 Cy5

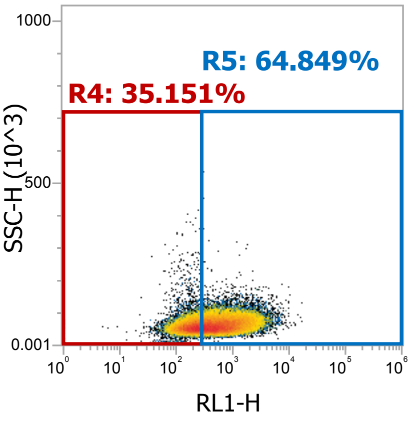


Experiment: **20210901**
Sample: **2. Trypsin Cells+SEQ2 Cy5**
Time Recorded: **19:06:02**

| Name | Gate | X Parameter | Y Parameter | Count | %Total | %Gated |
|---|---|---|---|---|---|---|
| All Events | All Events | N/A | N/A | 54,978 | 100.000 | 100.000 |
| R1 | R1 | FSC - FSC-H | SSC - SSC-A | 49,723 | 90.442 | 90.442 |
| R2 | R2 | FSC - FSC-A | FSC - FSC-H | 47,704 | 86.769 | 95.940 |
| R3 | R3 | VL1 - VL1-H | | 42,690 | 77.649 | 89.489 |
| R4 | R4 | RL1 - RL1-H | SSC - SSC-H | 15,006 | 27.295 | 35.151 |
| R5 | R5 | RL1 - RL1-H | SSC - SSC-H | 27,684 | 50.355 | 64.849 |

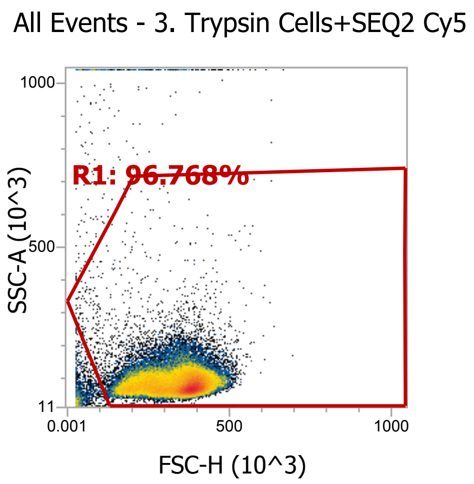


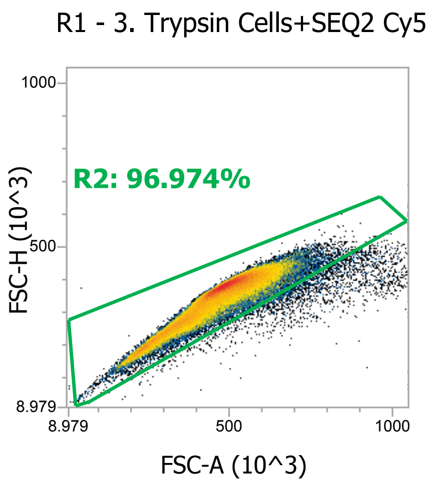


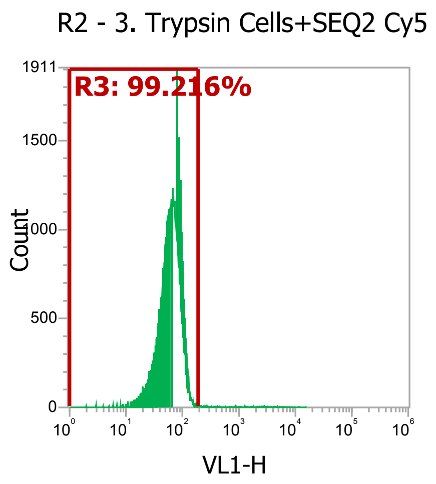


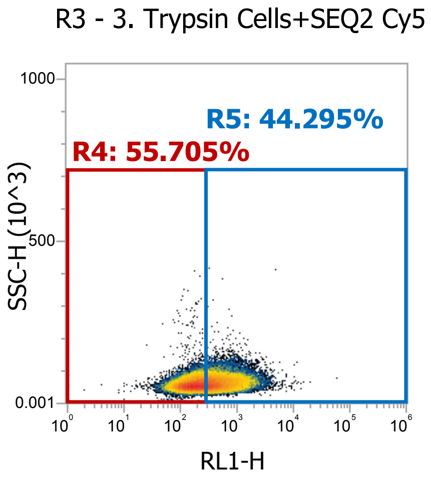


Experiment: **20210908**
Sample: **3. Trypsin Cells+SEQ2 Cy5**
Time Recorded: **17:47:33**

| Name | Gate | X Parameter | Y Parameter | Count | %Total | %Gated |
|---|---|---|---|---|---|---|
| All Events | All Events | N/A | N/A | 74,111 | 100.000 | 100.000 |
| R1 | R1 | FSC - FSC-H | SSC - SSC-A | 71,716 | 96.768 | 96.768 |
| R2 | R2 | FSC - FSC-A | FSC - FSC-H | 69,546 | 93.840 | 96.974 |
| R3 | R3 | VL1 - VL1-H | | 69,001 | 93.105 | 99.216 |
| R4 | R4 | RL1 - RL1-H | SSC - SSC-H | 38,437 | 51.864 | 55.705 |
| R5 | R5 | RL1 - RL1-H | SSC - SSC-H | 30,564 | 41.241 | 44.295 |

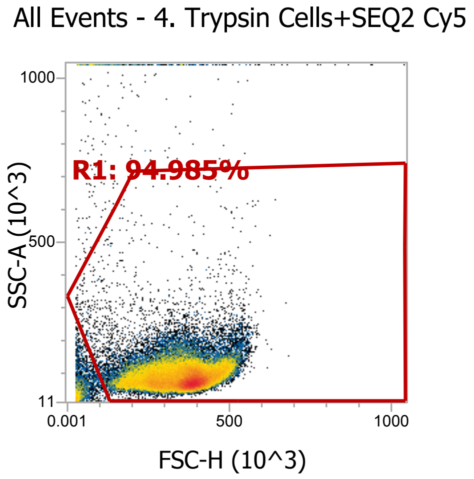


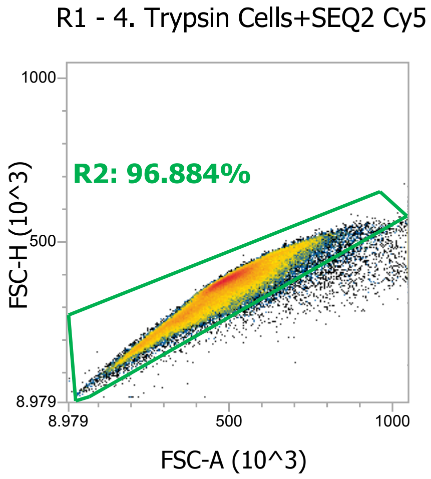


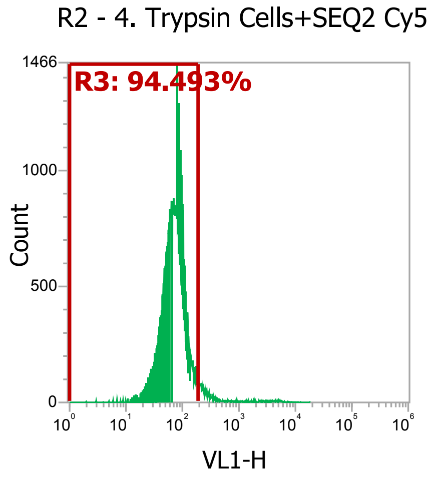


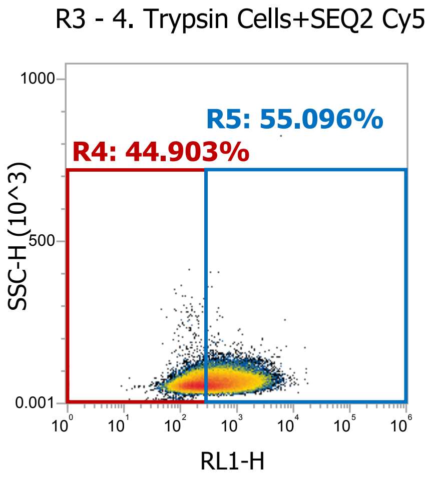


Experiment: **20210917**
Sample: **4. Trypsin Cells+SEQ2 Cy5**
Time Recorded: **15:15:44**

| Name | Gate | X Parameter | Y Parameter | Count | %Total | %Gated |
|---|---|---|---|---|---|---|
| All Events | All Events | N/A | N/A | 66,147 | 100.000 | 100.000 |
| R1 | R1 | FSC - FSC-H | SSC - SSC-A | 62,830 | 94.985 | 94.985 |
| R2 | R2 | FSC - FSC-A | FSC - FSC-H | 60,872 | 92.025 | 96.884 |
| R3 | R3 | VL1 - VL1-H | | 57,520 | 86.958 | 94.493 |
| R4 | R4 | RL1 - RL1-H | SSC - SSC-H | 25,828 | 39.046 | 44.903 |
| R5 | R5 | RL1 - RL1-H | SSC - SSC-H | 31,691 | 47.910 | 55.096 |
