# Supplementary material for: A retrotransposon-derived DNA zip code internalizes myeloma cells through Clathrin-Rab5a-mediated endocytosis
Source: Front Oncol. 2024 Feb 23;14:1288724. doi: 10.3389/fonc.2024.1288724 (PMC10920344; doi:10.3389/fonc.2024.1288724)
Supplement: Supplementary file 12 [file DataSheet_1.zip › Raw data/Fig 2A, MM1R with different ZCs/MM1R different ZC 1mg per mL.pdf]

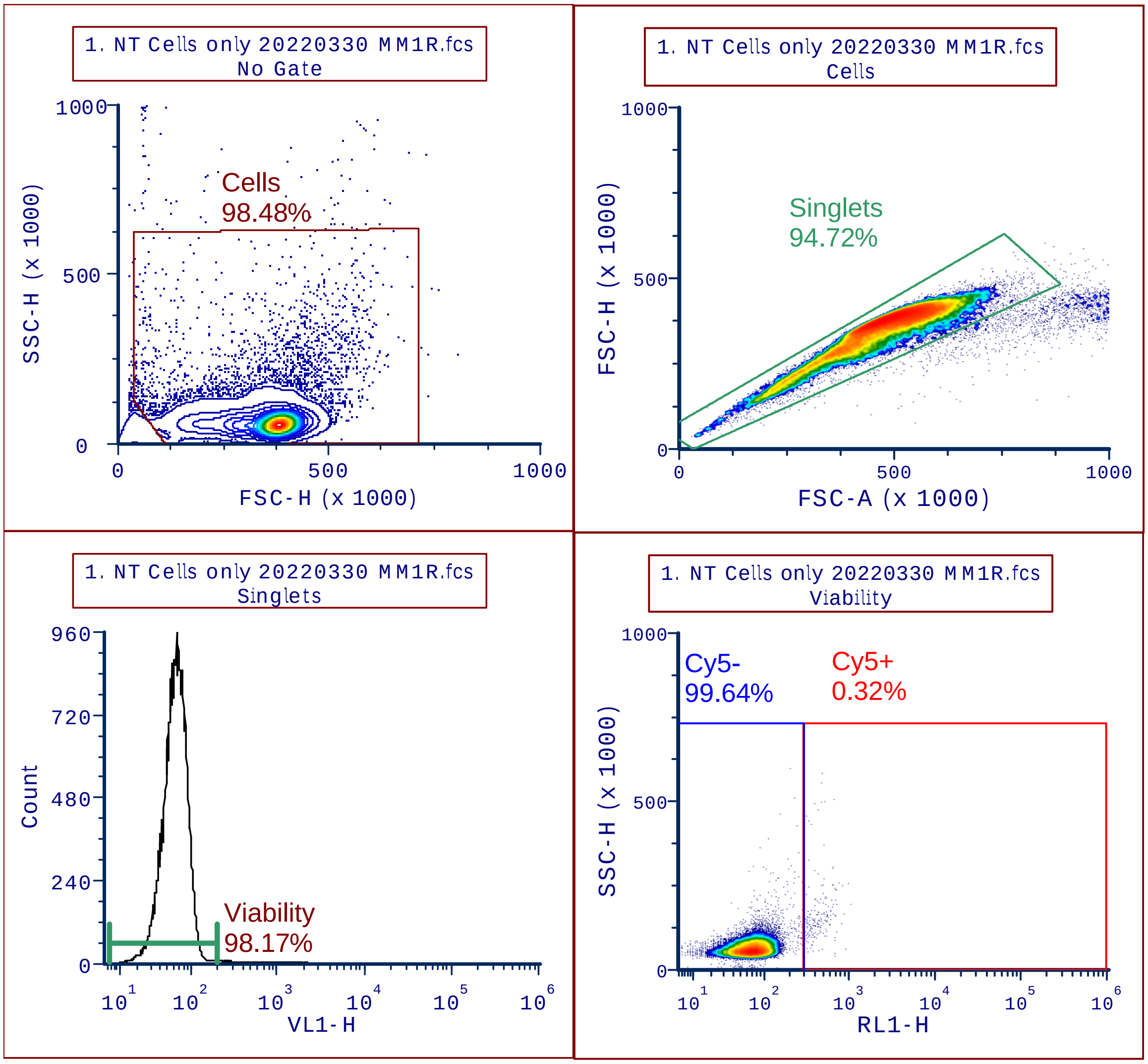

	A	B	C	D	E
1	Filename	Gate	# of Events	% of all cells	% of gated cells
2	1. NT Cells on	All Events	63906	100	100
3	1. NT Cells on	Cells	62935	98.48	98.48
4	1. NT Cells on	Singlets	59611	93.28	94.72
5	1. NT Cells on	Viability	58521	91.57	98.17
6	1. NT Cells on	Cy5+	190	0.3	0.32
7	1. NT Cells on	Cy5-	58312	91.25	99.64
8					
9					

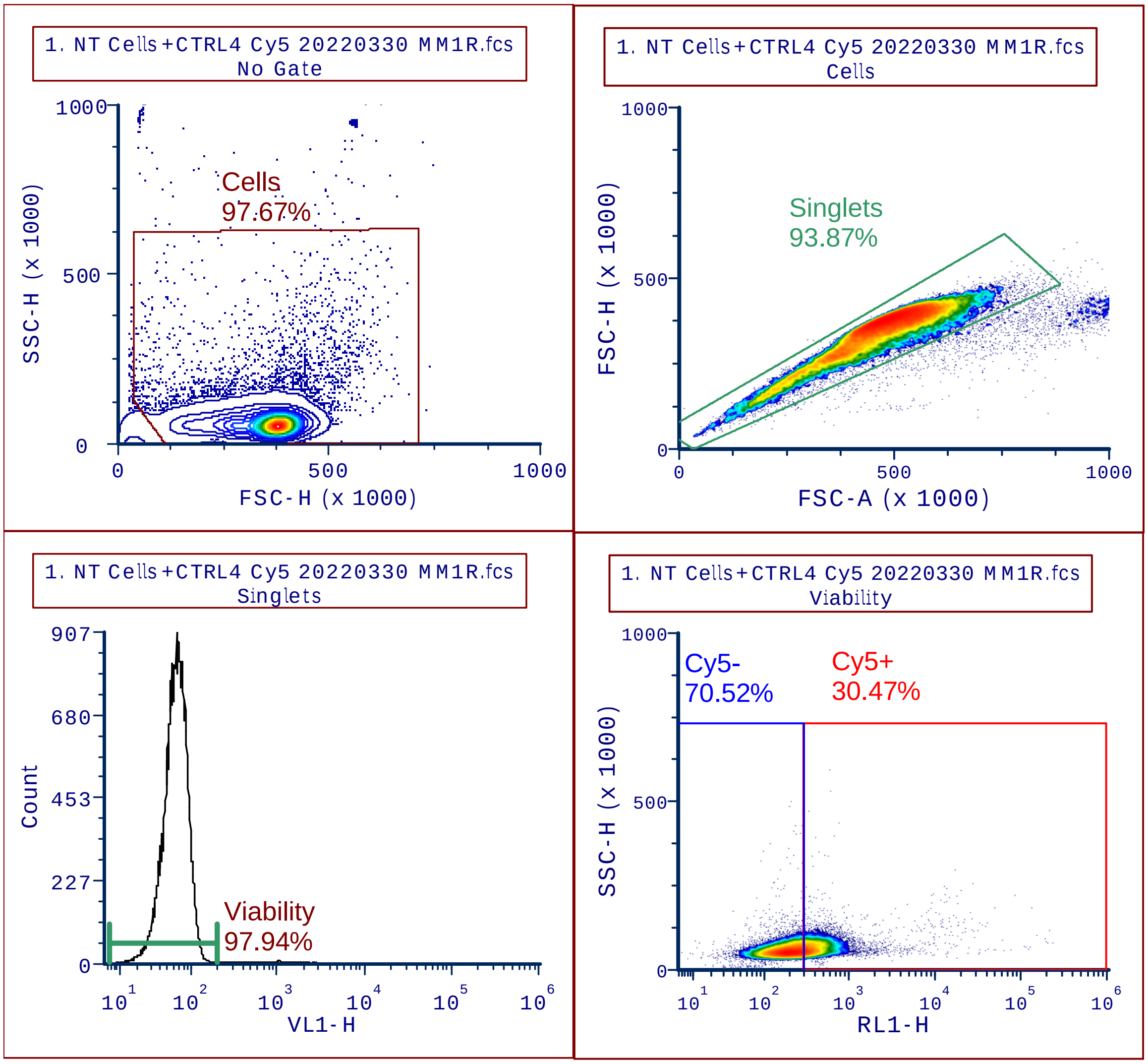

	A	B	C	D	E
1	Filename	Gate	# of Events	% of all cells	% of gated cells
2	1. NT Cells+C	All Events	61605	100	100
3	1. NT Cells+C	Cells	60168	97.67	97.67
4	1. NT Cells+C	Singlets	56477	91.68	93.87
5	1. NT Cells+C	Viability	55315	89.79	97.94
6	1. NT Cells+C	Cy5+	16853	27.36	30.47
7	1. NT Cells+C	Cy5-	39007	63.32	70.52
8					
9					

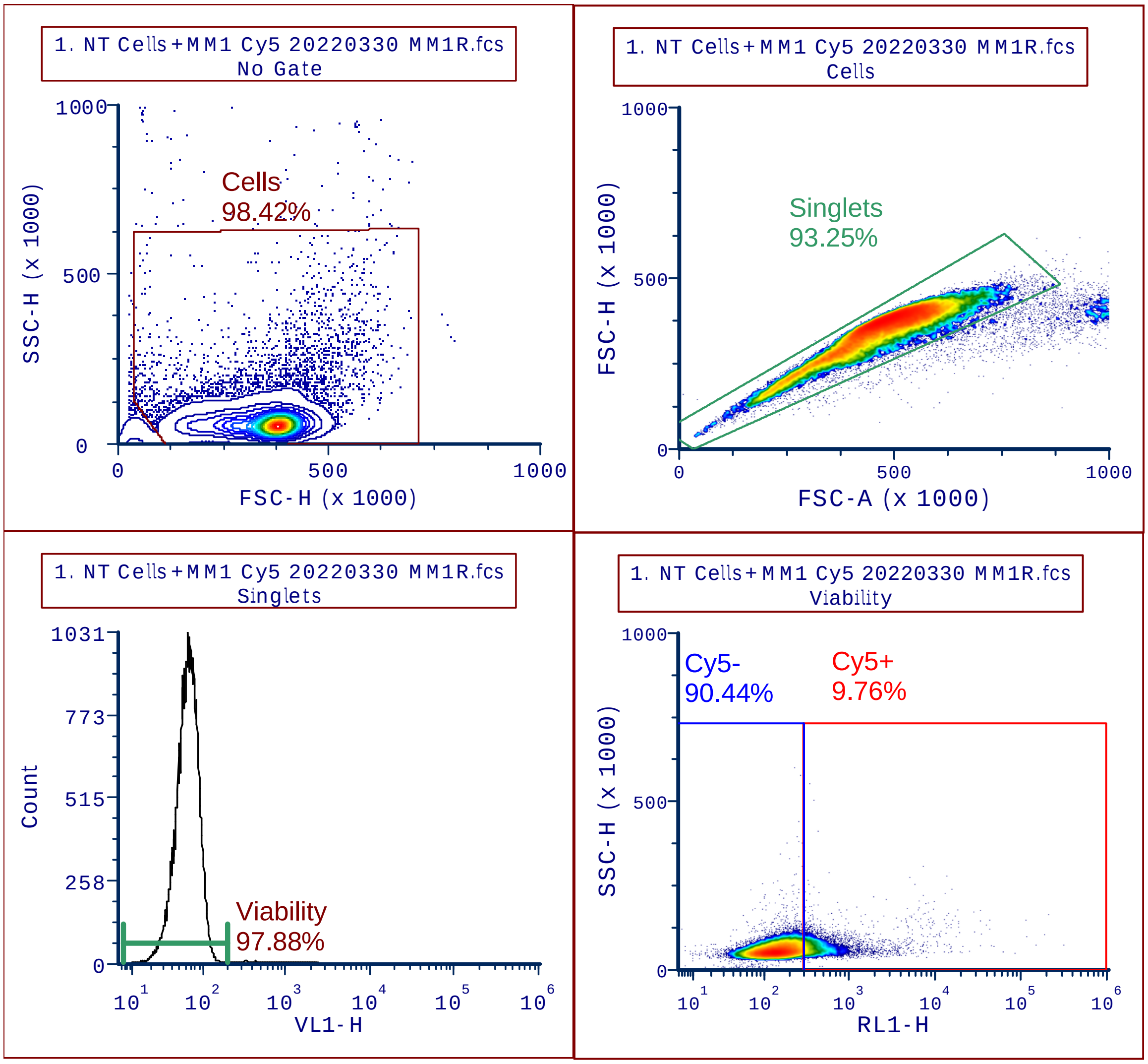

	A	B	C	D	E
1	Filename	Gate	# of Events	% of all cells	% of gated cells
2	1. NT Cells+M	All Events	69763	100	100
3	1. NT Cells+M	Cells	68664	98.42	98.42
4	1. NT Cells+M	Singlets	64032	91.79	93.25
5	1. NT Cells+M	Viability	62677	89.84	97.88
6	1. NT Cells+M	Cy5+	6117	8.77	9.76
7	1. NT Cells+M	Cy5-	56684	81.25	90.44
8					
9					

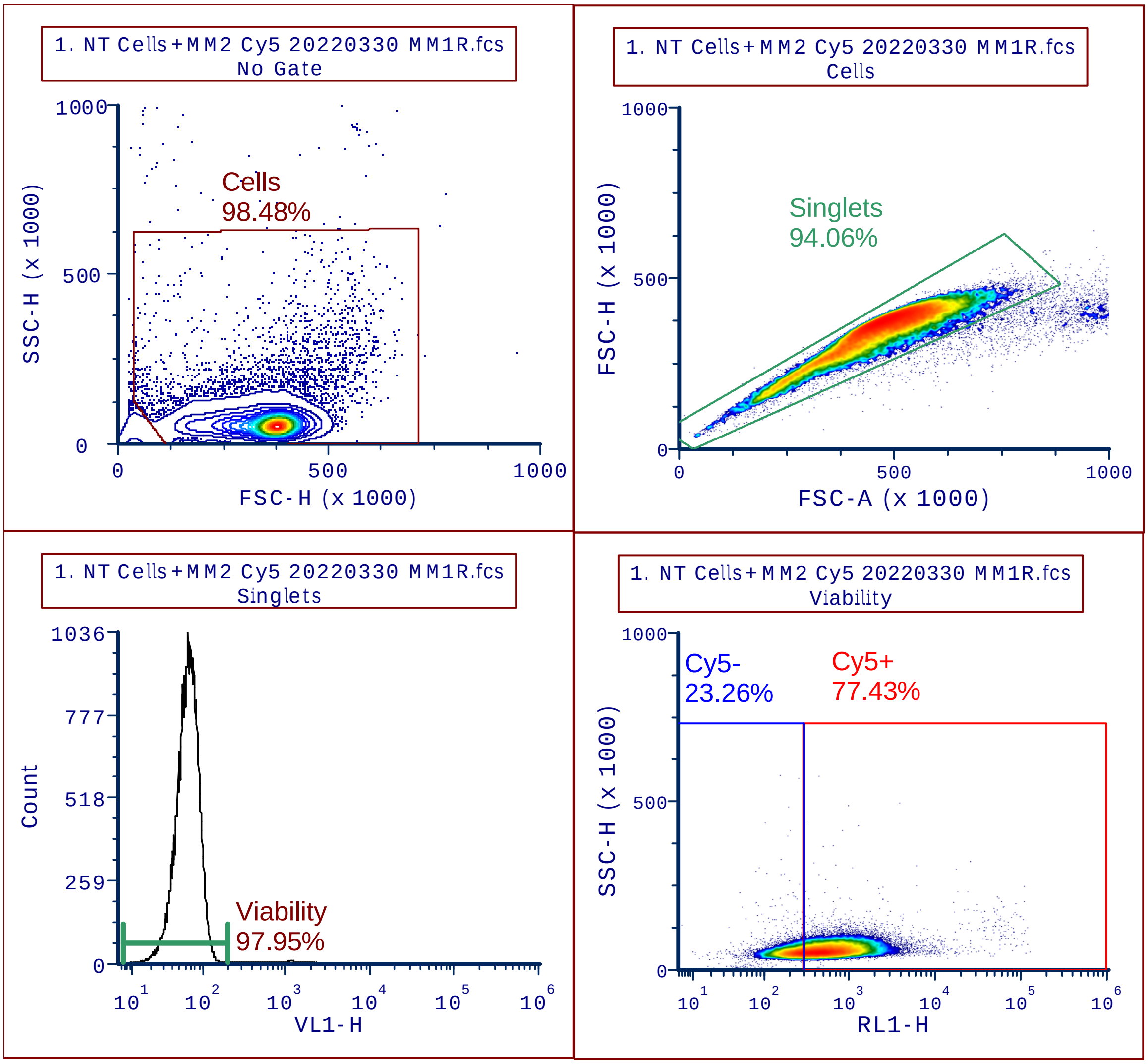

	A	B	C	D	E
1	Filename	Gate	# of Events	% of all cells	% of gated cells
2	1. NT Cells+M	All Events	68525	100	100
3	1. NT Cells+M	Cells	67484	98.48	98.48
4	1. NT Cells+M	Singlets	63476	92.63	94.06
5	1. NT Cells+M	Viability	62172	90.73	97.95
6	1. NT Cells+M	Cy5+	48142	70.25	77.43
7	1. NT Cells+M	Cy5-	14459	21.1	23.26
8					
9					

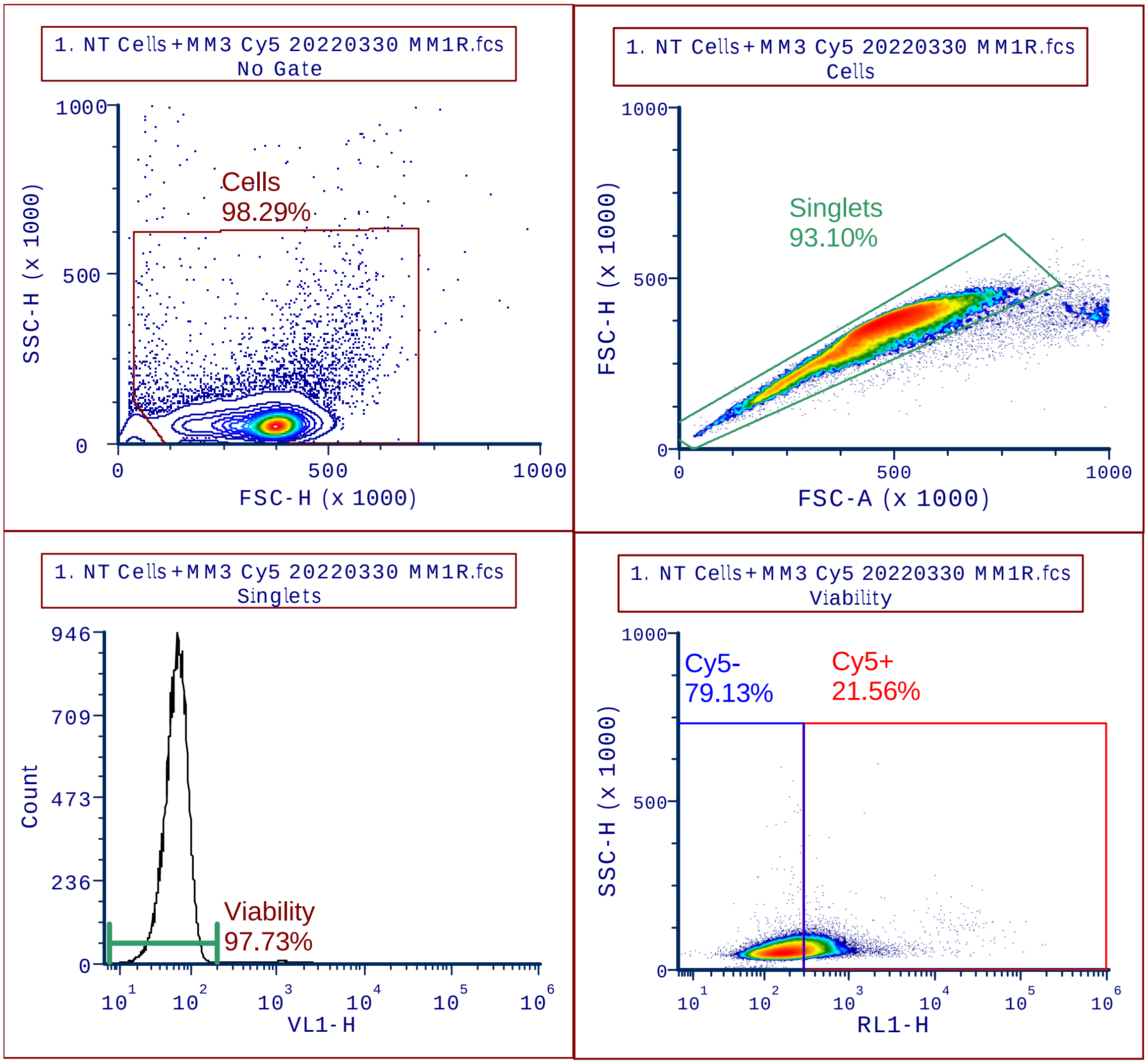

	A	B	C	D	E
1	**Filename**	**Gate**	**# of Events**	**% of all cells**	**% of gated cells**
2	1. NT Cells+M	All Events	64044	100	100
3	1. NT Cells+M	Cells	62952	98.29	98.29
4	1. NT Cells+M	Singlets	58609	91.51	93.1
5	1. NT Cells+M	Viability	57276	89.43	97.73
6	1. NT Cells+M	Cy5+	12349	19.28	21.56
7	1. NT Cells+M	Cy5-	45320	70.76	79.13
8					
9					

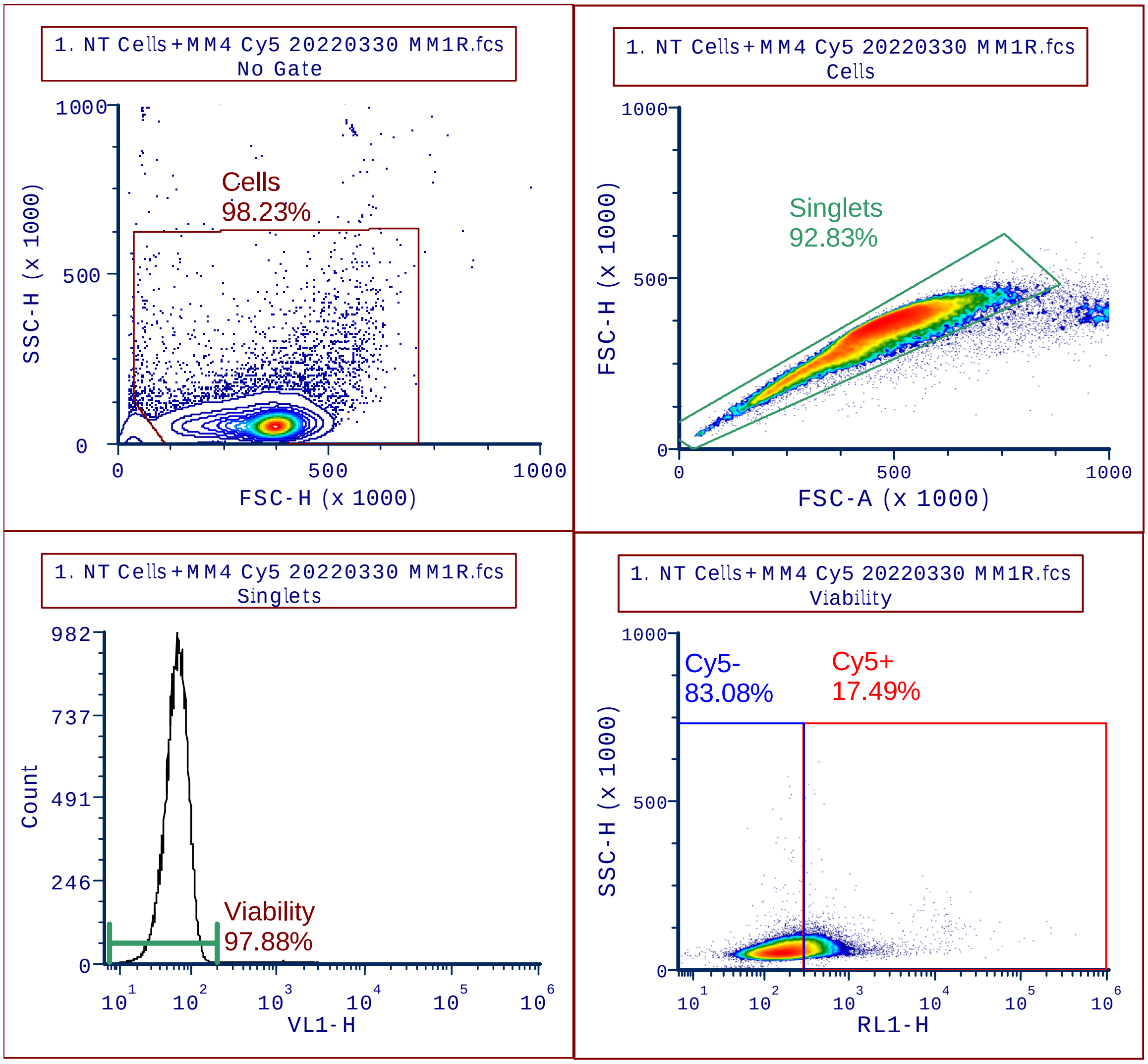

	A	B	C	D	E
1	Filename	Gate	# of Events	% of all cells	% of gated cells
2	1. NT Cells+M	All Events	67536	100	100
3	1. NT Cells+M	Cells	66342	98.23	98.23
4	1. NT Cells+M	Singlets	61584	91.19	92.83
5	1. NT Cells+M	Viability	60276	89.25	97.88
6	1. NT Cells+M	Cy5+	10541	15.61	17.49
7	1. NT Cells+M	Cy5-	50075	74.15	83.08
8					
9					

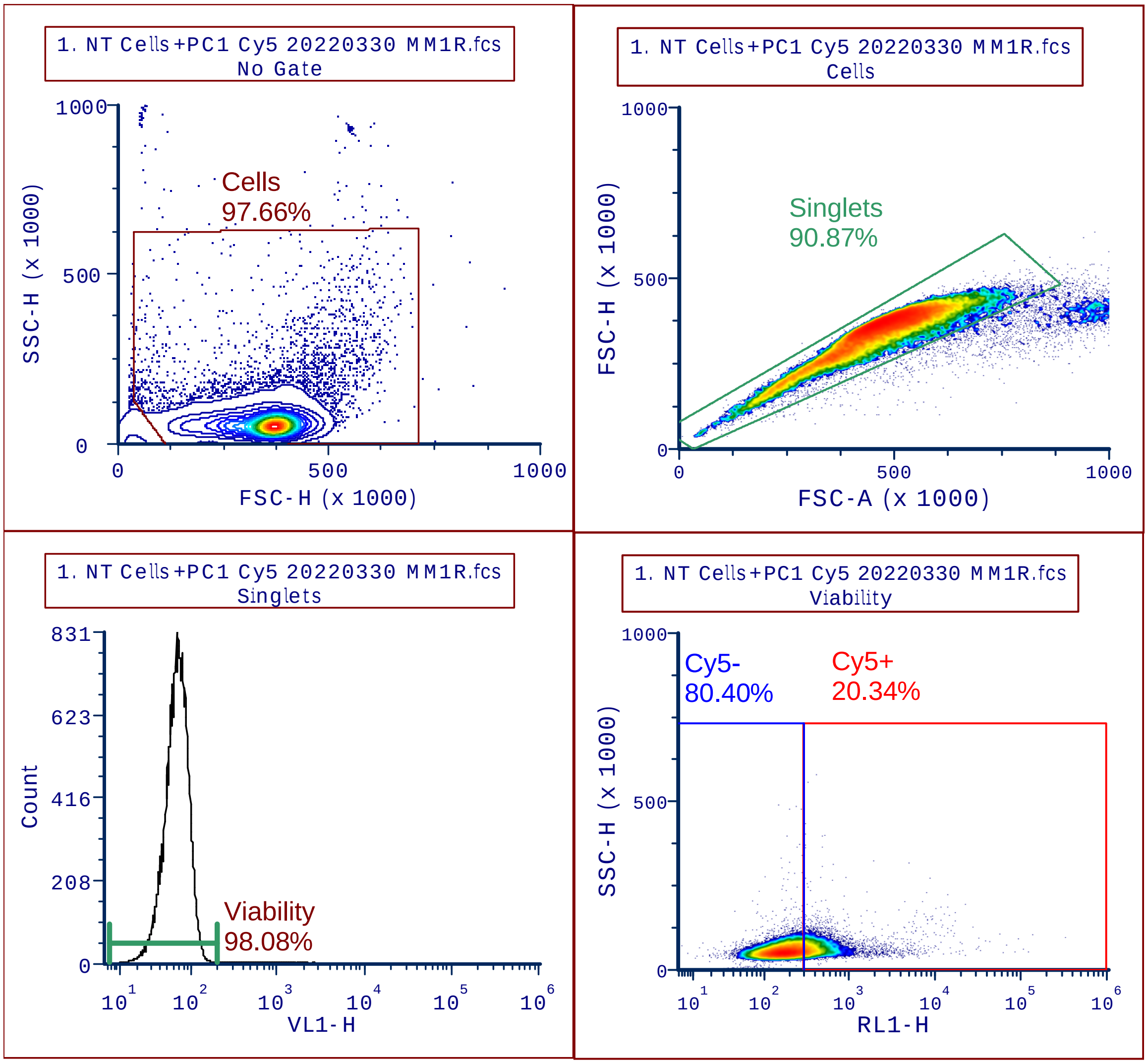

	A	B	C	D	E
1	Filename	Gate	# of Events	% of all cells	% of gated cells
2	1. NT Cells+P	All Events	58037	100	100
3	1. NT Cells+P	Cells	56680	97.66	97.66
4	1. NT Cells+P	Singlets	51504	88.74	90.87
5	1. NT Cells+P	Viability	50517	87.04	98.08
6	1. NT Cells+P	Cy5+	10277	17.71	20.34
7	1. NT Cells+P	Cy5-	40614	69.98	80.4
8					
9					

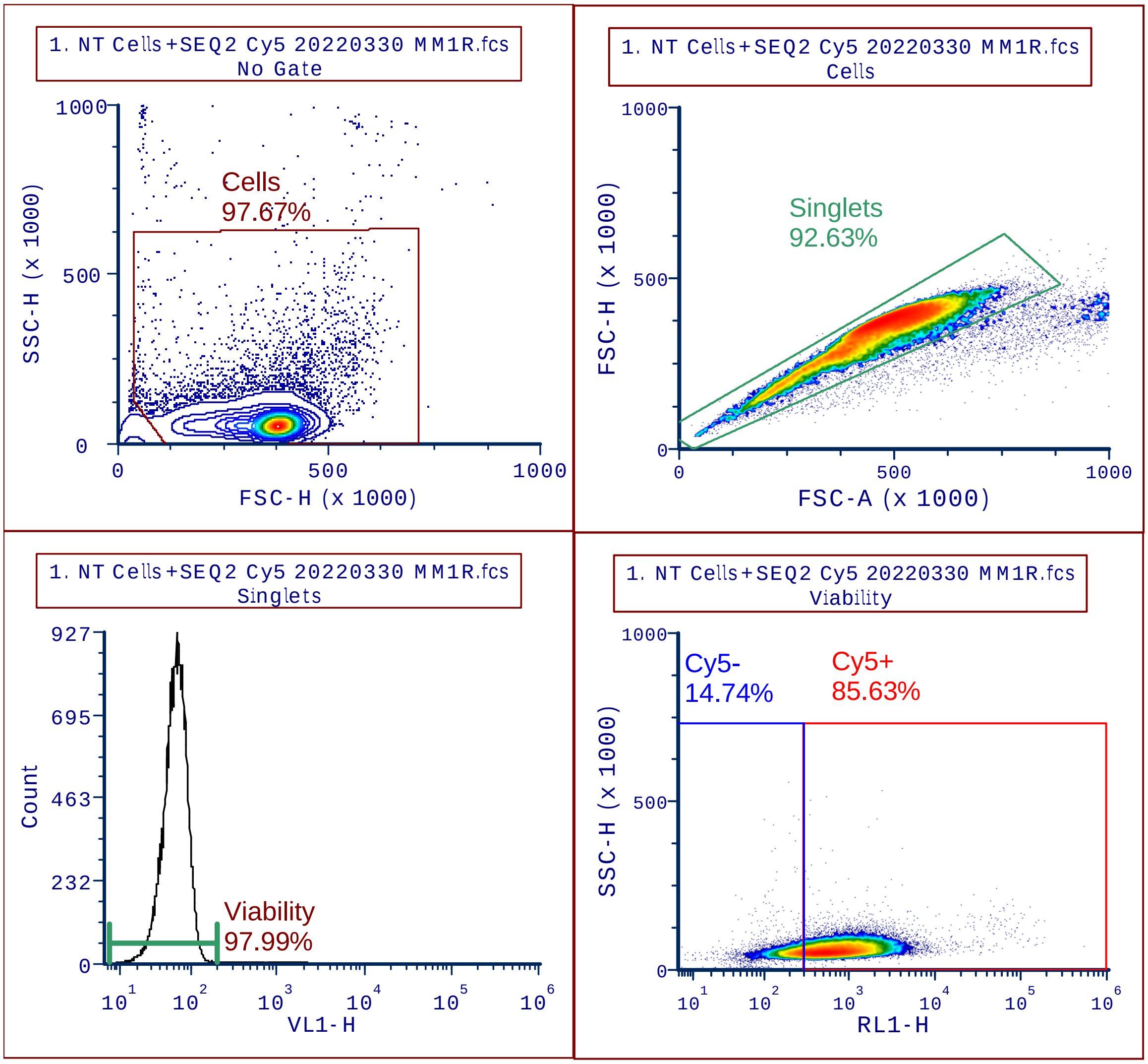

	A	B	C	D	E
1	**Filename**	**Gate**	**# of Events**	**% of all cells**	**% of gated cells**
2	1. NT Cells+S	All Events	62929	100	100
3	1. NT Cells+S	Cells	61464	97.67	97.67
4	1. NT Cells+S	Singlets	56937	90.48	92.63
5	1. NT Cells+S	Viability	55791	88.66	97.99
6	1. NT Cells+S	Cy5+	47773	75.92	85.63
7	1. NT Cells+S	Cy5-	8223	13.07	14.74
8					
9					

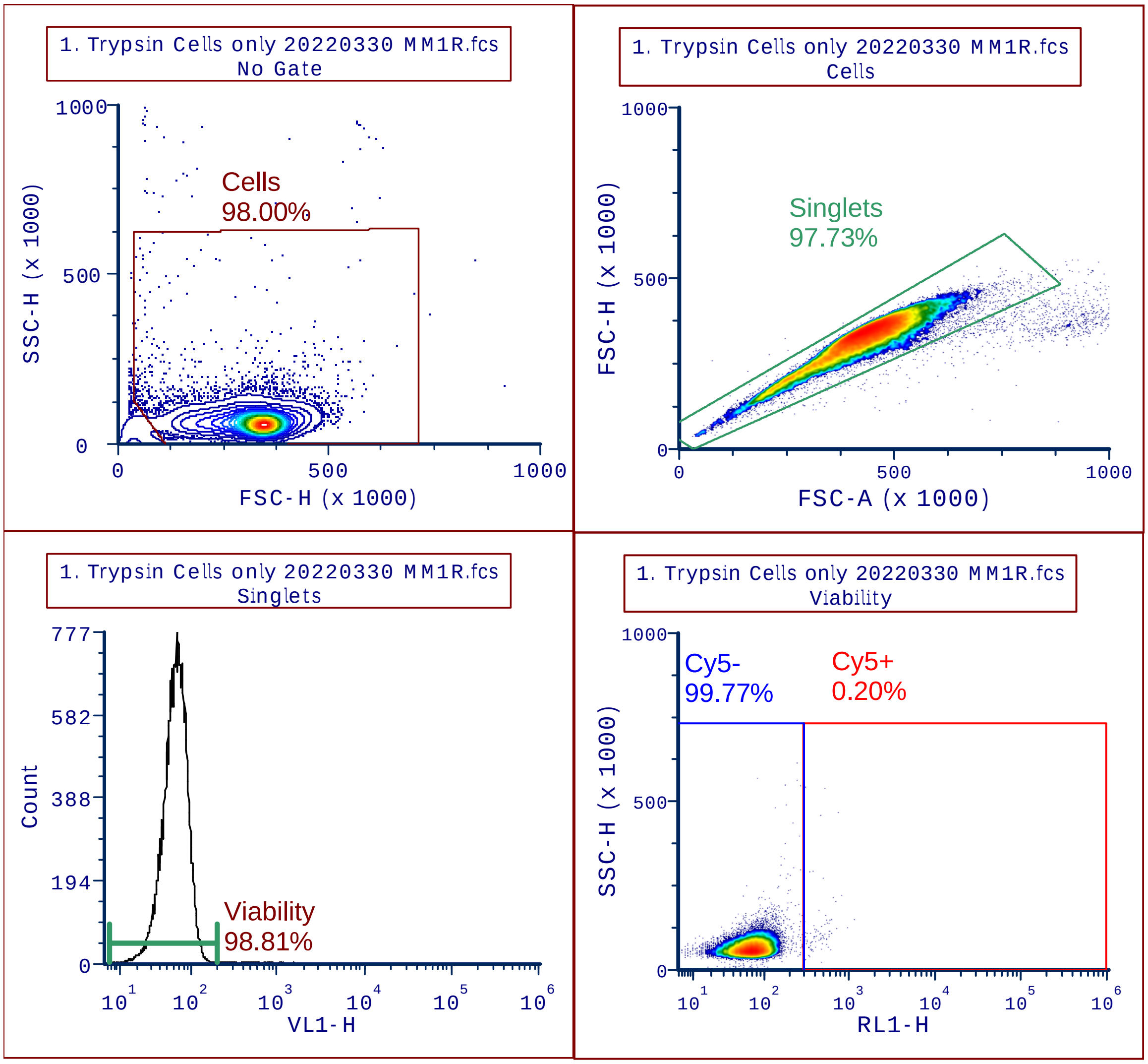

	A	B	C	D	E
1	Filename	Gate	# of Events	% of all cells	% of gated cells
2	1. Trypsin Ce	All Events	50750	100	100
3	1. Trypsin Ce	Cells	49733	98	98
4	1. Trypsin Ce	Singlets	48605	95.77	97.73
5	1. Trypsin Ce	Viability	48025	94.63	98.81
6	1. Trypsin Ce	Cy5+	94	0.19	0.2
7	1. Trypsin Ce	Cy5-	47915	94.41	99.77
8					
9					

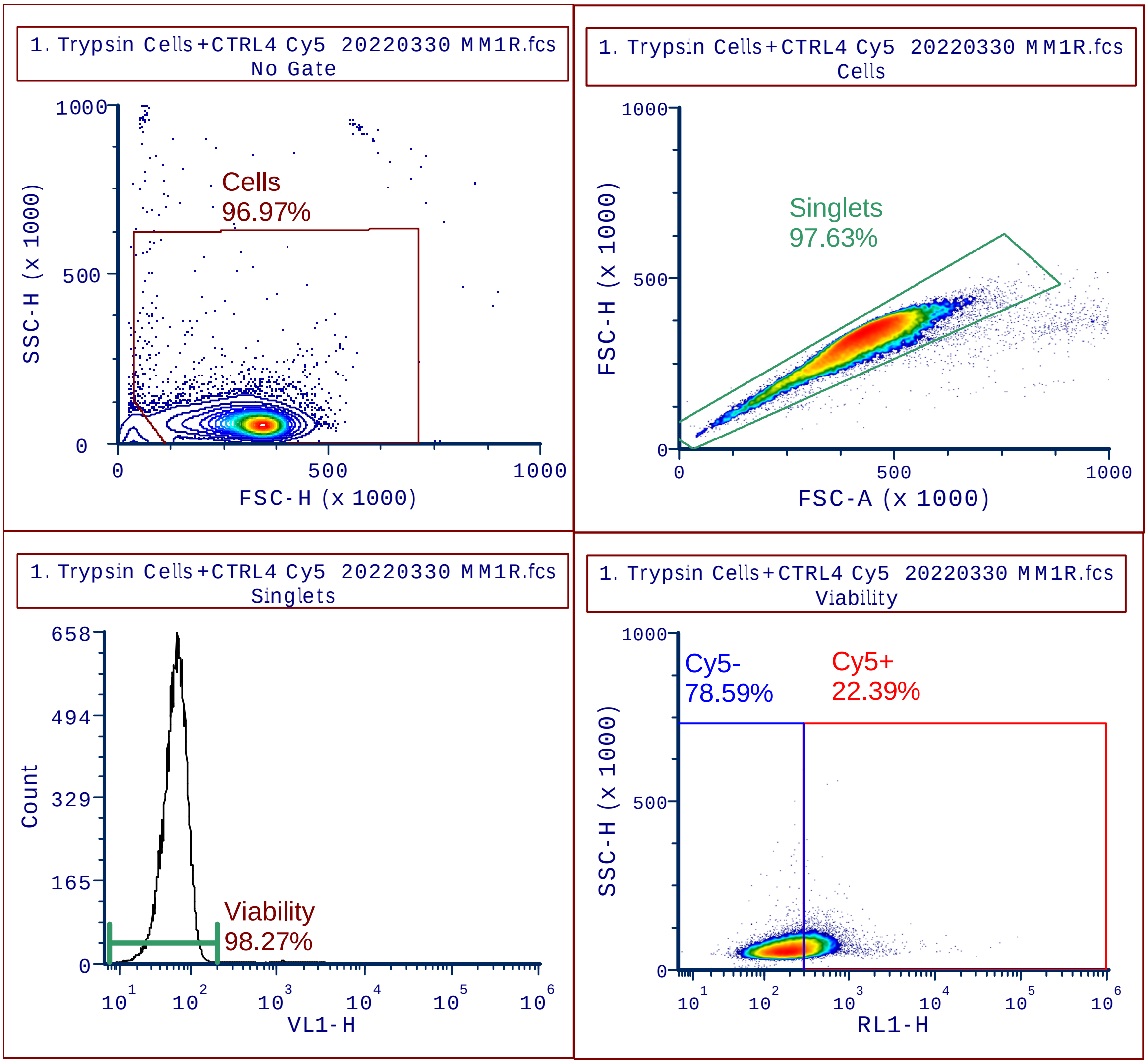

	A	B	C	D	E
1	Filename	Gate	# of Events	% of all cells	% of gated cells
2	1. Trypsin Ce	All Events	43375	100	100
3	1. Trypsin Ce	Cells	42062	96.97	96.97
4	1. Trypsin Ce	Singlets	41064	94.67	97.63
5	1. Trypsin Ce	Viability	40353	93.03	98.27
6	1. Trypsin Ce	Cy5+	9035	20.83	22.39
7	1. Trypsin Ce	Cy5-	31715	73.12	78.59
8					
9					

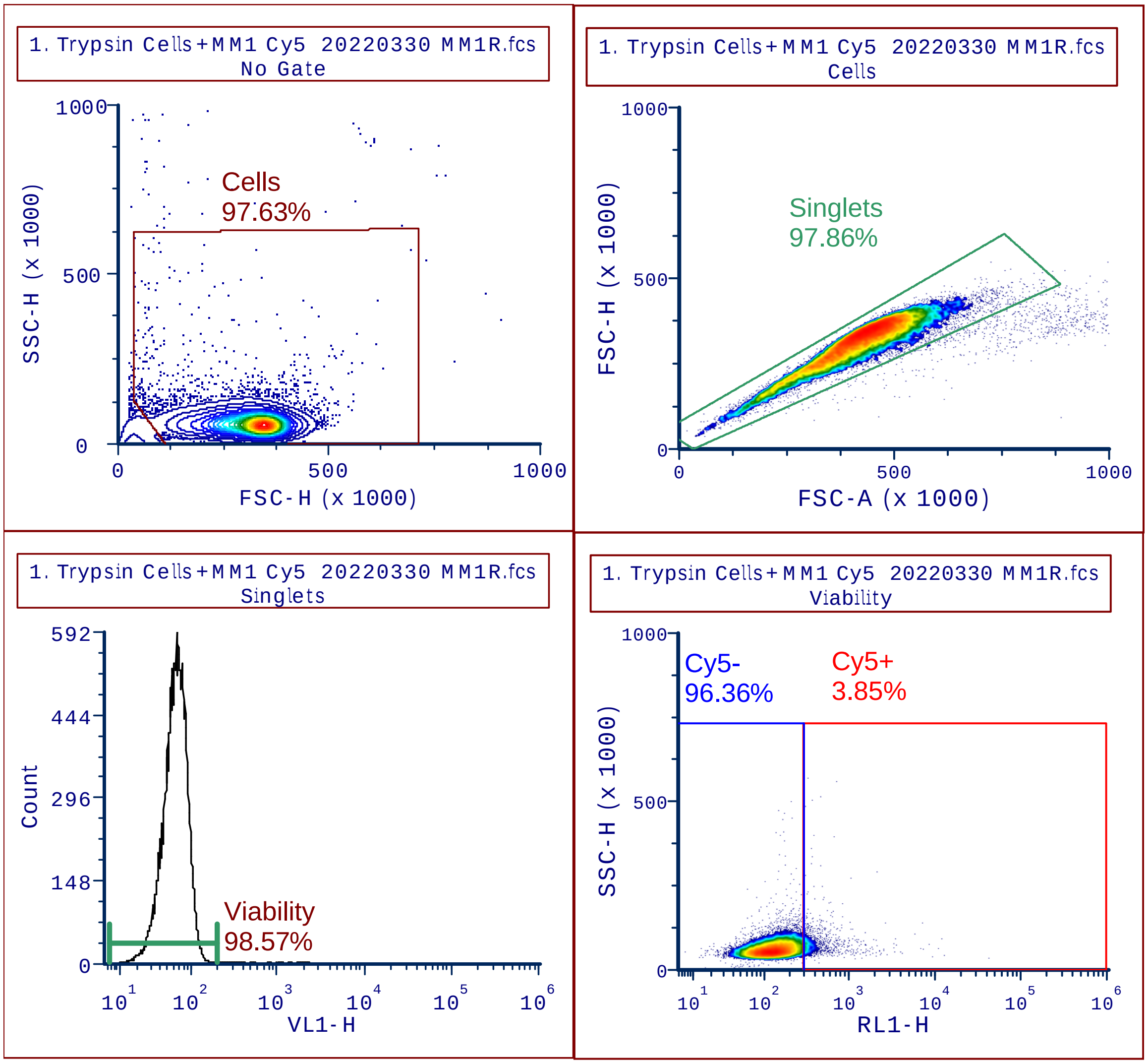

	A	B	C	D	E
1	Filename	Gate	# of Events	% of all cells	% of gated cells
2	1. Trypsin Ce	All Events	38411	100	100
3	1. Trypsin Ce	Cells	37501	97.63	97.63
4	1. Trypsin Ce	Singlets	36697	95.54	97.86
5	1. Trypsin Ce	Viability	36173	94.17	98.57
6	1. Trypsin Ce	Cy5+	1392	3.62	3.85
7	1. Trypsin Ce	Cy5-	34858	90.75	96.36
8					
9					

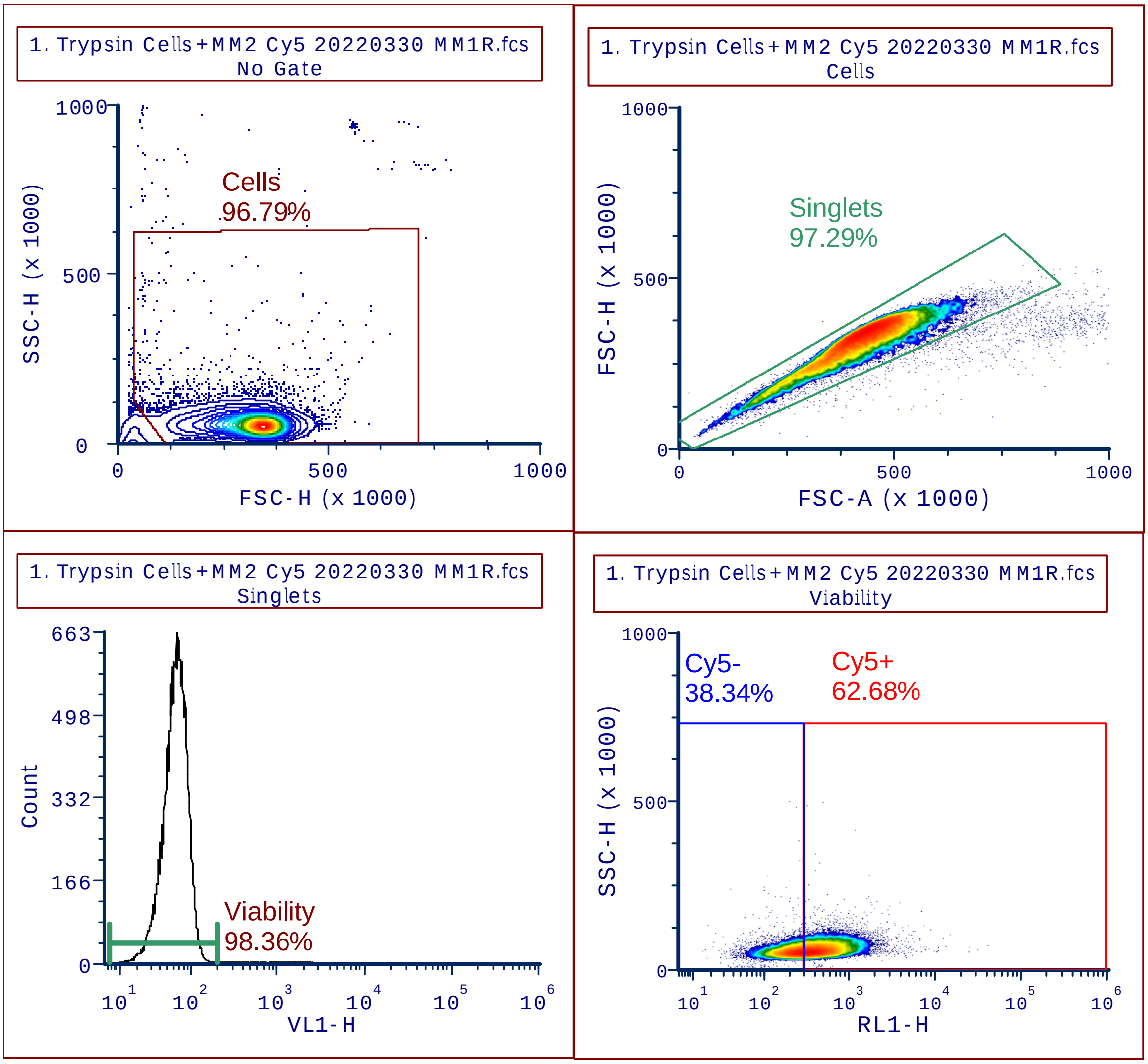

	A	B	C	D	E
1	Filename	Gate	# of Events	% of all cells	% of gated cells
2	1. Trypsin Ce	All Events	44243	100	100
3	1. Trypsin Ce	Cells	42825	96.79	96.79
4	1. Trypsin Ce	Singlets	41666	94.18	97.29
5	1. Trypsin Ce	Viability	40982	92.63	98.36
6	1. Trypsin Ce	Cy5+	25687	58.06	62.68
7	1. Trypsin Ce	Cy5-	15713	35.52	38.34
8					
9					

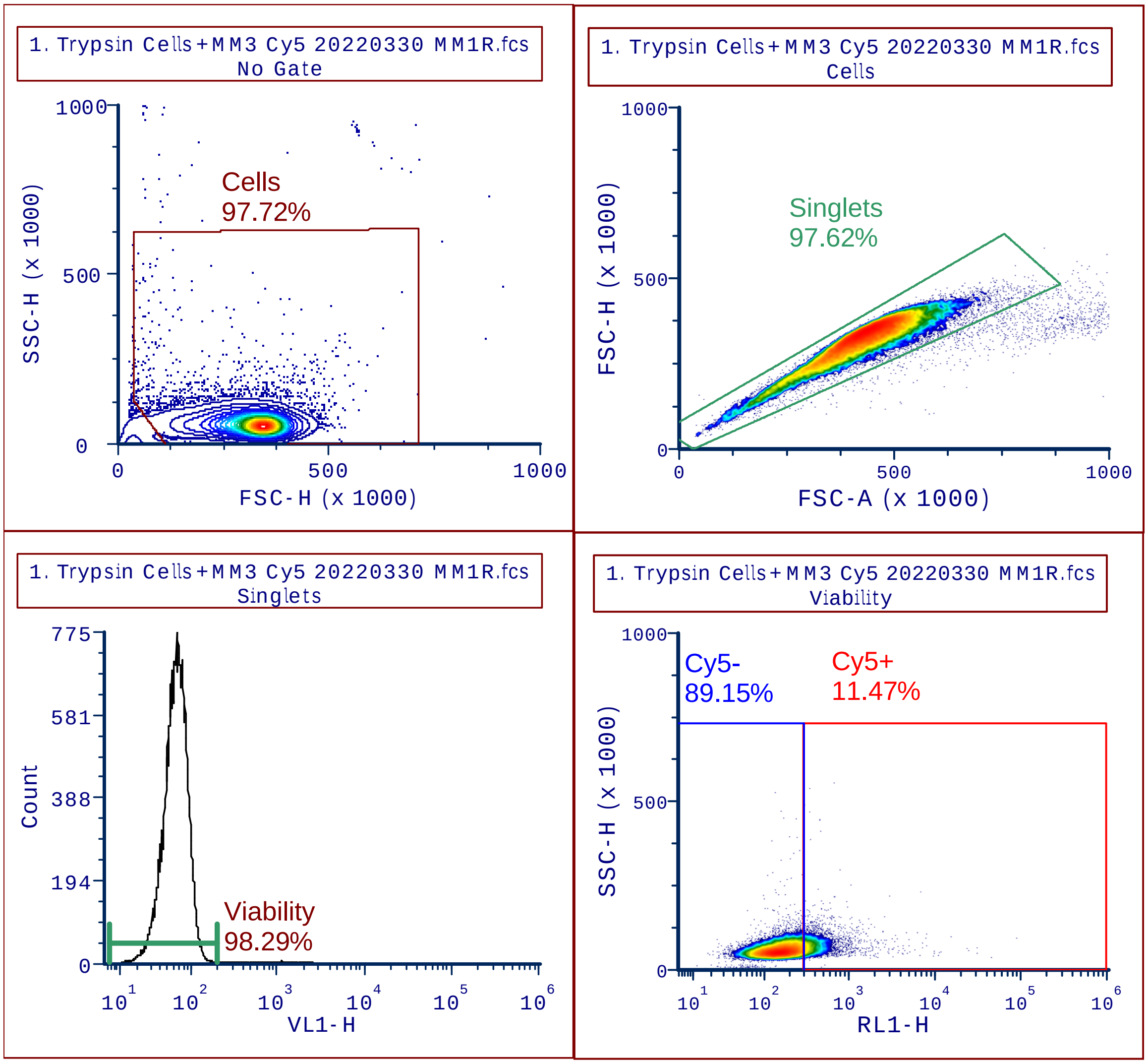

	A	B	C	D	E
1	Filename	Gate	# of Events	% of all cells	% of gated cells
2	1. Trypsin Ce	All Events	50569	100	100
3	1. Trypsin Ce	Cells	49414	97.72	97.72
4	1. Trypsin Ce	Singlets	48240	95.39	97.62
5	1. Trypsin Ce	Viability	47415	93.76	98.29
6	1. Trypsin Ce	Cy5+	5438	10.75	11.47
7	1. Trypsin Ce	Cy5-	42272	83.59	89.15
8					
9					

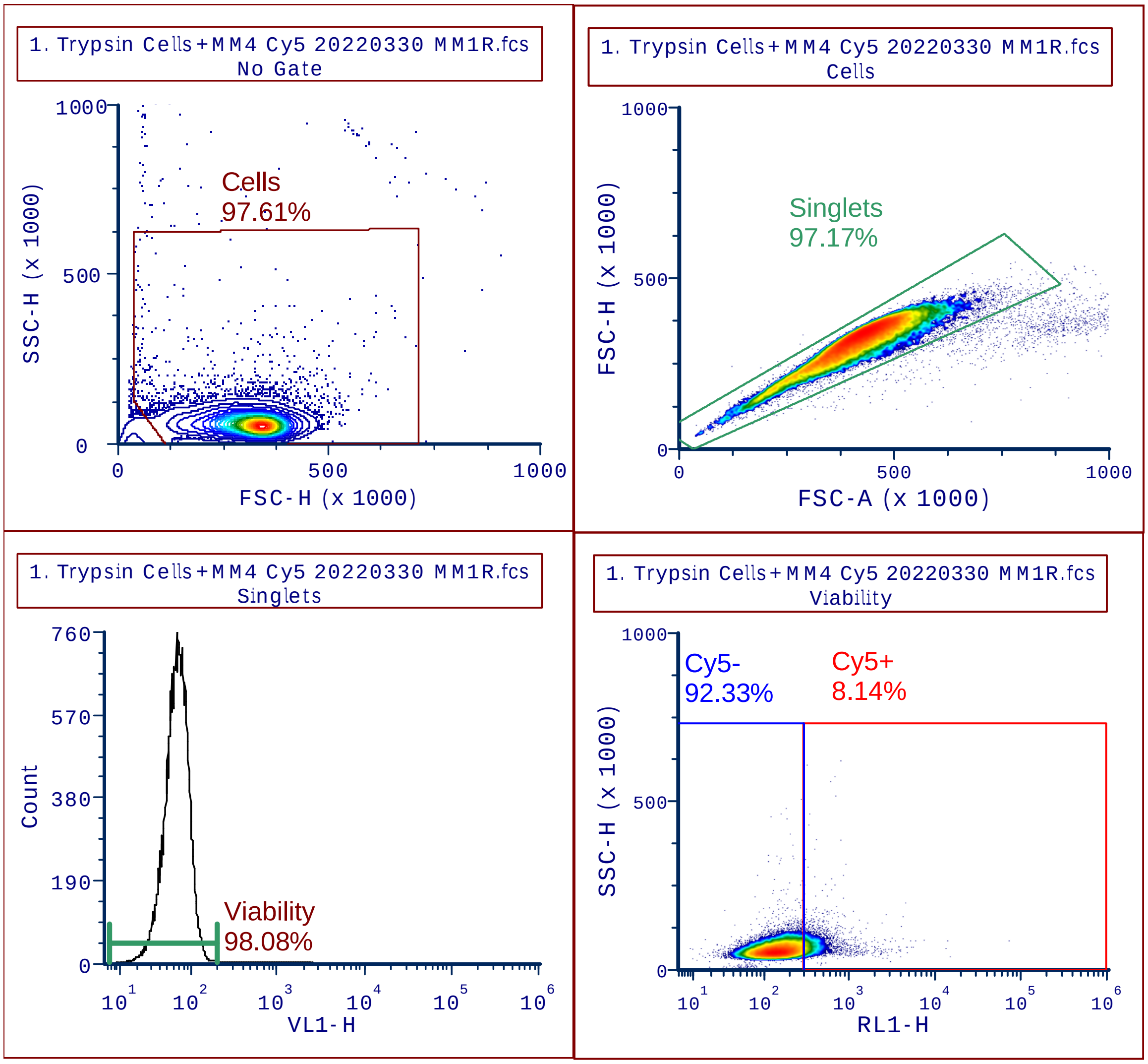

	A	B	C	D	E
1	**Filename**	**Gate**	**# of Events**	**% of all cells**	**% of gated cells**
2	1. Trypsin Ce	All Events	51278	100	100
3	1. Trypsin Ce	Cells	50050	97.61	97.61
4	1. Trypsin Ce	Singlets	48632	94.84	97.17
5	1. Trypsin Ce	Viability	47696	93.01	98.08
6	1. Trypsin Ce	Cy5+	3884	7.57	8.14
7	1. Trypsin Ce	Cy5-	44040	85.88	92.33
8					
9					

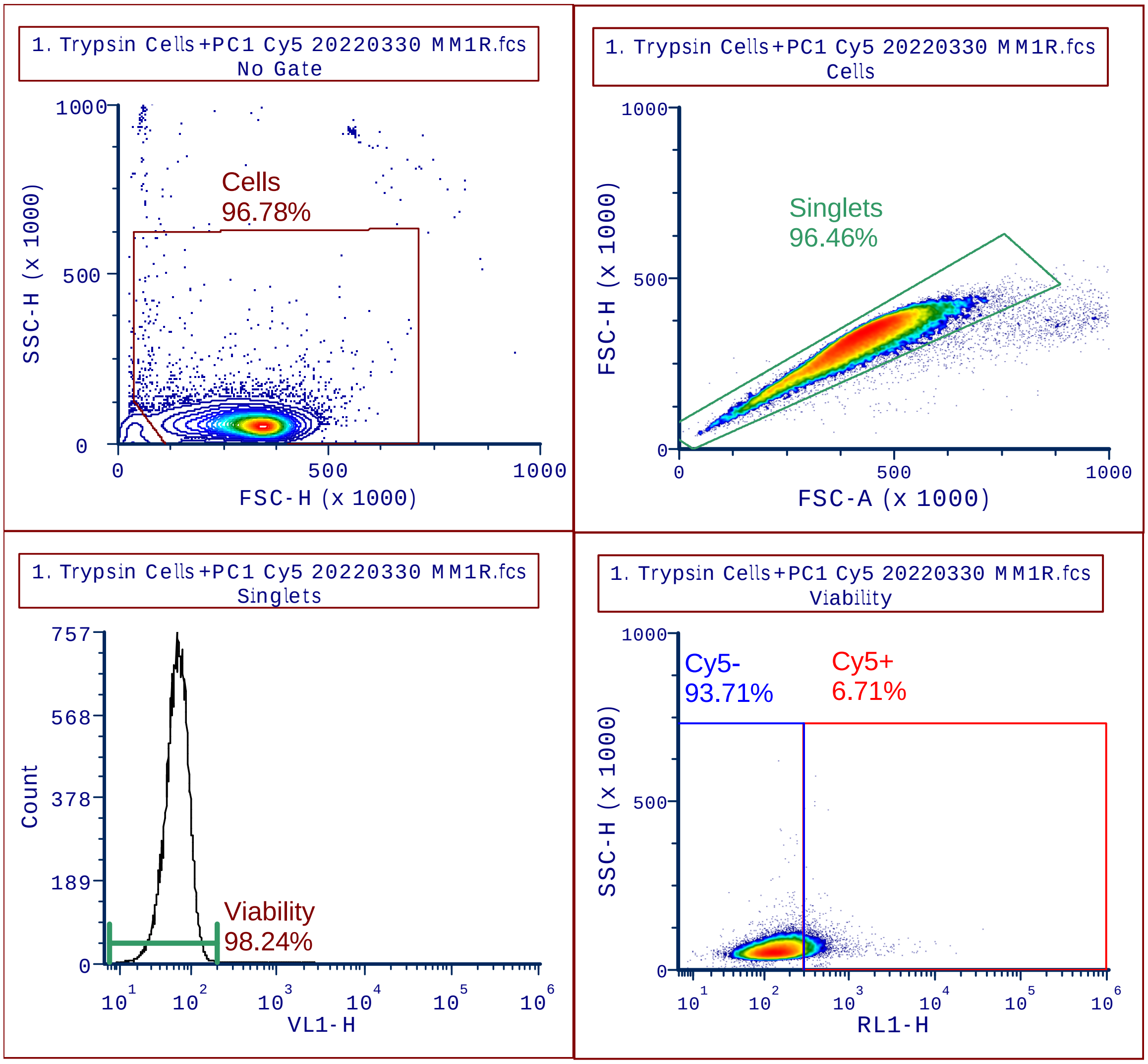

	A	B	C	D	E
1	Filename	**Gate**	**# of Events**	**% of all cells**	**% of gated cells**
2	1. Trypsin Ce	All Events	51524	100	100
3	1. Trypsin Ce	Cells	49867	96.78	96.78
4	1. Trypsin Ce	Singlets	48101	93.36	96.46
5	1. Trypsin Ce	Viability	47253	91.71	98.24
6	1. Trypsin Ce	Cy5+	3169	6.15	6.71
7	1. Trypsin Ce	Cy5-	44279	85.94	93.71
8					
9					

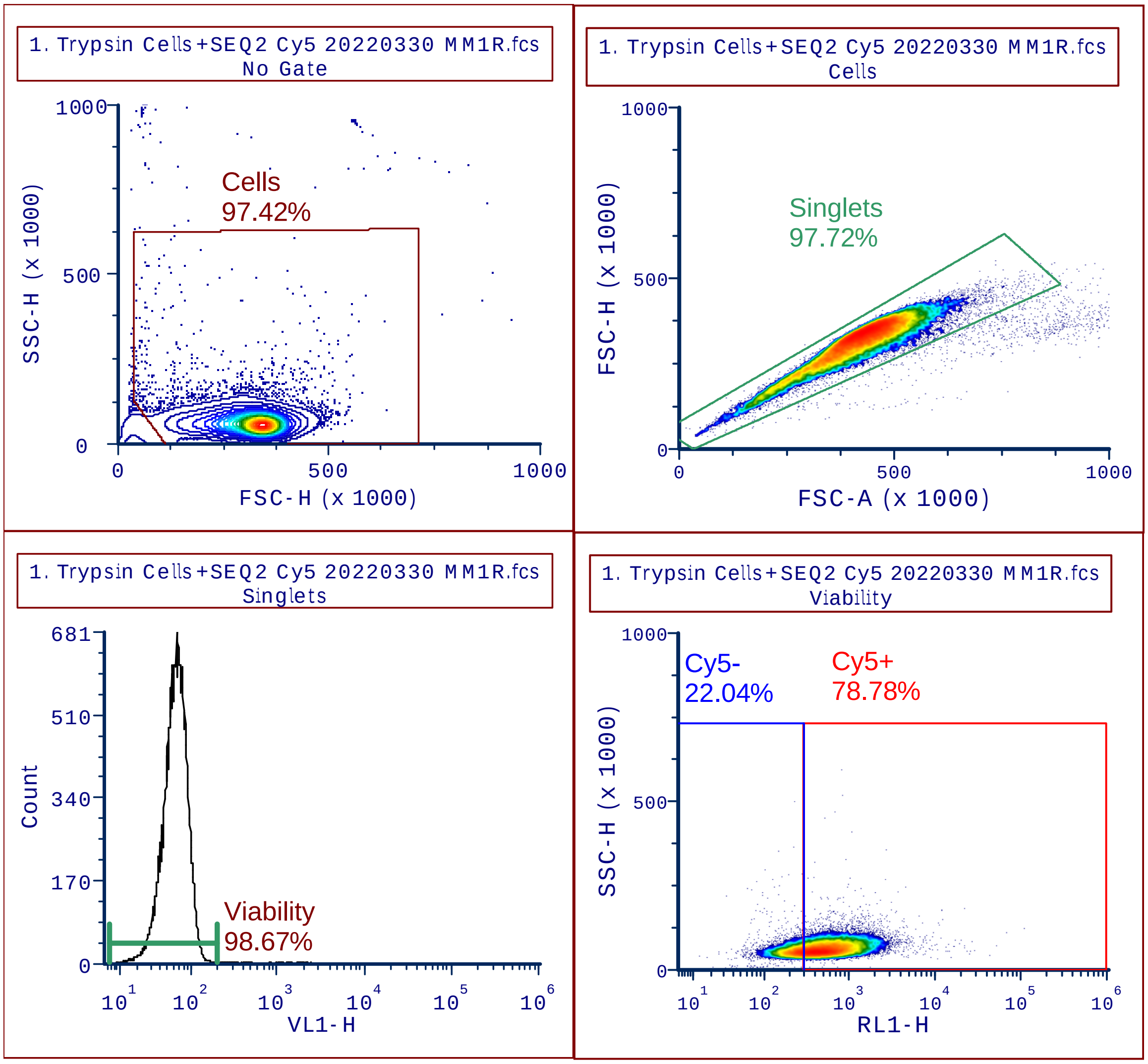

	A	B	C	D	E
1	Filename	Gate	# of Events	% of all cells	% of gated cells
2	1. Trypsin Ce	All Events	44077	100	100
3	1. Trypsin Ce	Cells	42940	97.42	97.42
4	1. Trypsin Ce	Singlets	41960	95.2	97.72
5	1. Trypsin Ce	Viability	41400	93.93	98.67
6	1. Trypsin Ce	Cy5+	32614	73.99	78.78
7	1. Trypsin Ce	Cy5-	9124	20.7	22.04
8					
9					

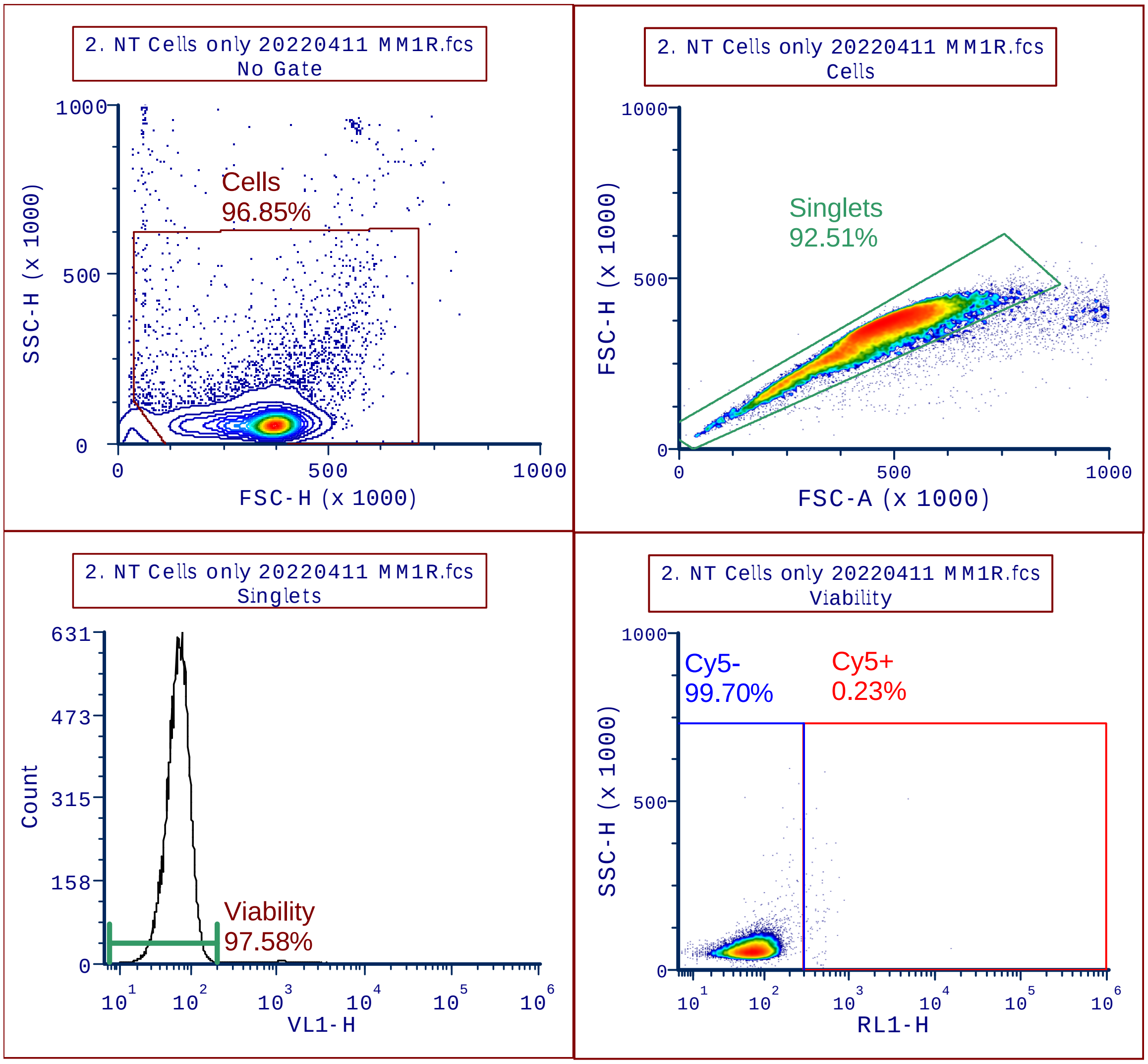

	A	B	C	D	E
1	**Filename**	**Gate**	**# of Events**	**% of all cells**	**% of gated cells**
2	2. NT Cells on	All Events	44534	100	100
3	2. NT Cells on	Cells	43131	96.85	96.85
4	2. NT Cells on	Singlets	39900	89.59	92.51
5	2. NT Cells on	Viability	38934	87.43	97.58
6	2. NT Cells on	Cy5+	89	0.2	0.23
7	2. NT Cells on	Cy5-	38819	87.17	99.7
8					
9					

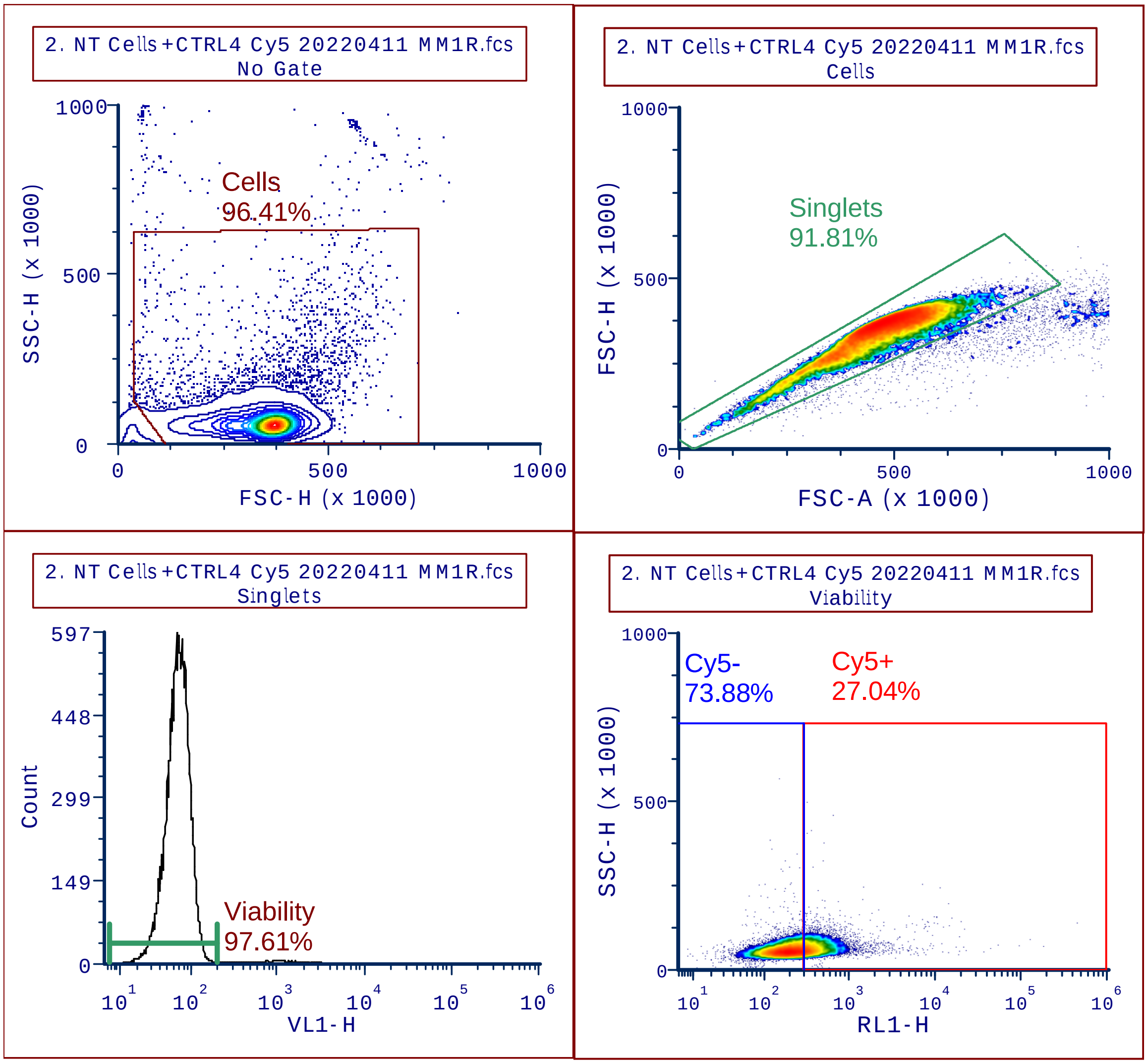

Filename	Gate	# of Events	% of all cells	% of gated cells
2. NT Cells+C	All Events	42976	100	100
2. NT Cells+C	Cells	41434	96.41	96.41
2. NT Cells+C	Singlets	38041	88.52	91.81
2. NT Cells+C	Viability	37130	86.4	97.61
2. NT Cells+C	Cy5+	10040	23.36	27.04
2. NT Cells+C	Cy5-	27433	63.83	73.88

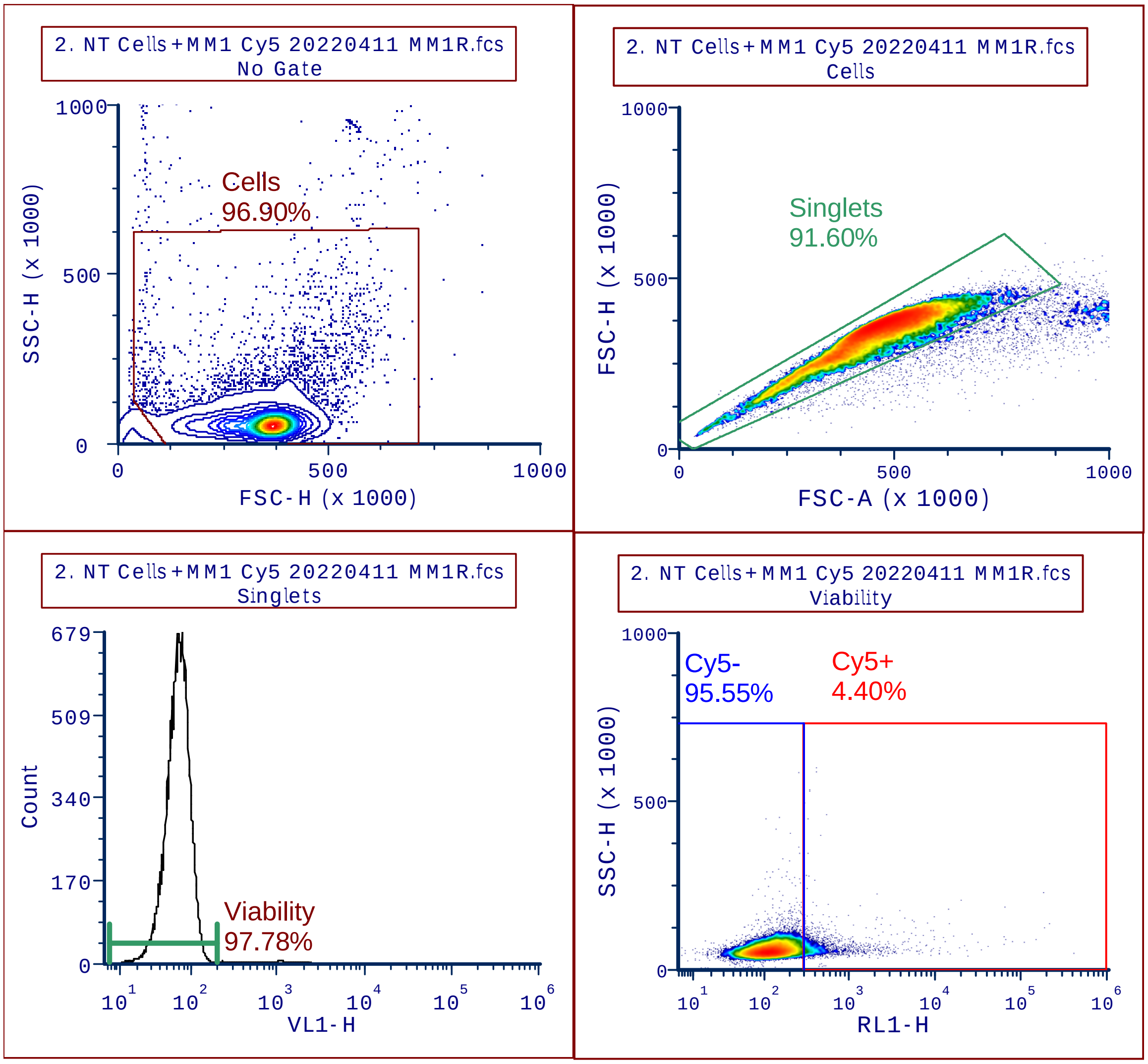

	A	B	C	D	E
1	**Filename**	**Gate**	**# of Events**	**% of all cells**	**% of gated cells**
2	2. NT Cells+M	All Events	48474	100	100
3	2. NT Cells+M	Cells	46972	96.9	96.9
4	2. NT Cells+M	Singlets	43028	88.77	91.6
5	2. NT Cells+M	Viability	42071	86.79	97.78
6	2. NT Cells+M	Cy5+	1851	3.82	4.4
7	2. NT Cells+M	Cy5-	40200	82.93	95.55
8					
9					

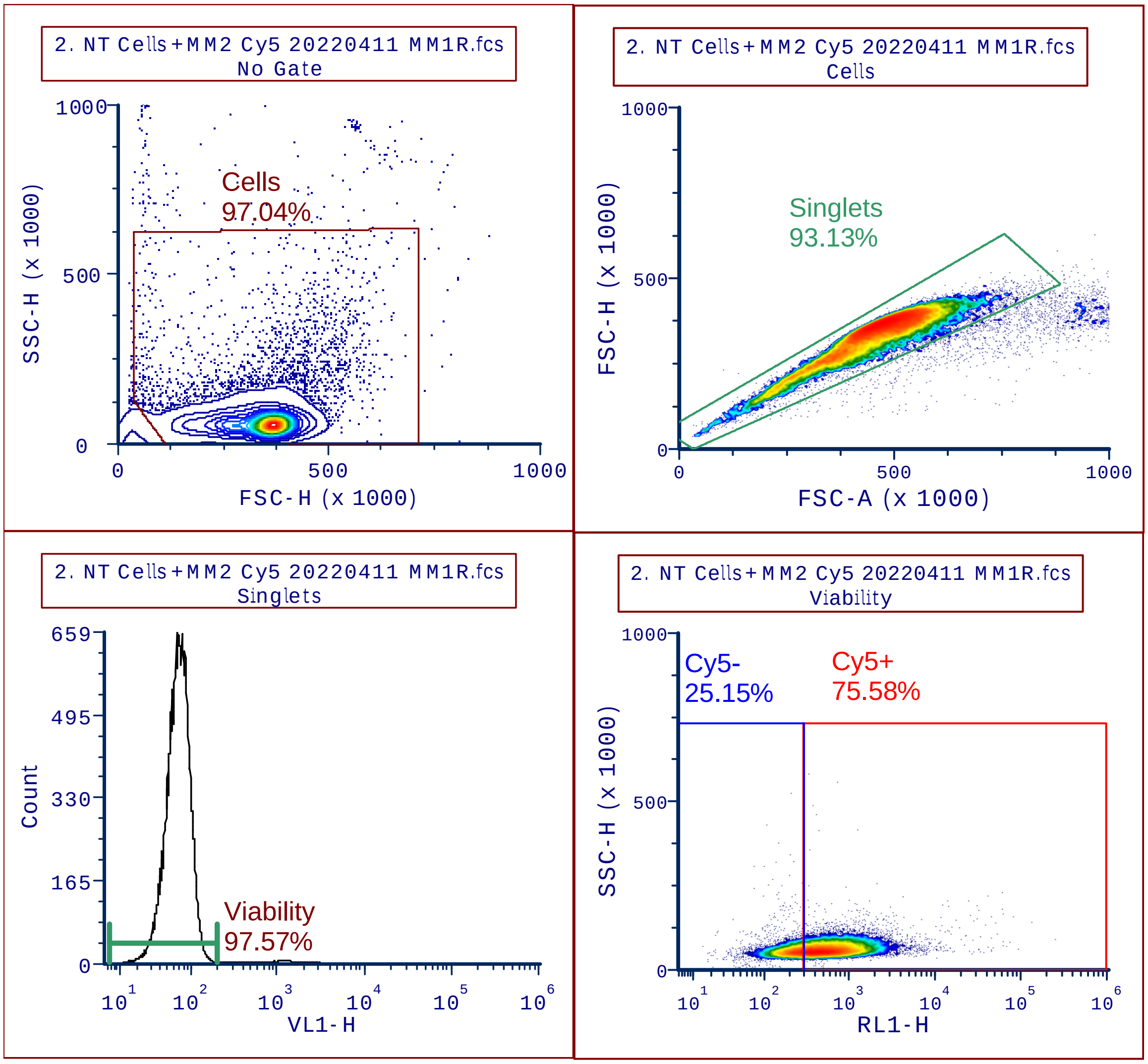

	A	B	C	D	E
1	Filename	Gate	# of Events	% of all cells	% of gated cells
2	2. NT Cells+M	All Events	47170	100	100
3	2. NT Cells+M	Cells	45775	97.04	97.04
4	2. NT Cells+M	Singlets	42632	90.38	93.13
5	2. NT Cells+M	Viability	41596	88.18	97.57
6	2. NT Cells+M	Cy5+	31438	66.65	75.58
7	2. NT Cells+M	Cy5-	10461	22.18	25.15
8					
9					

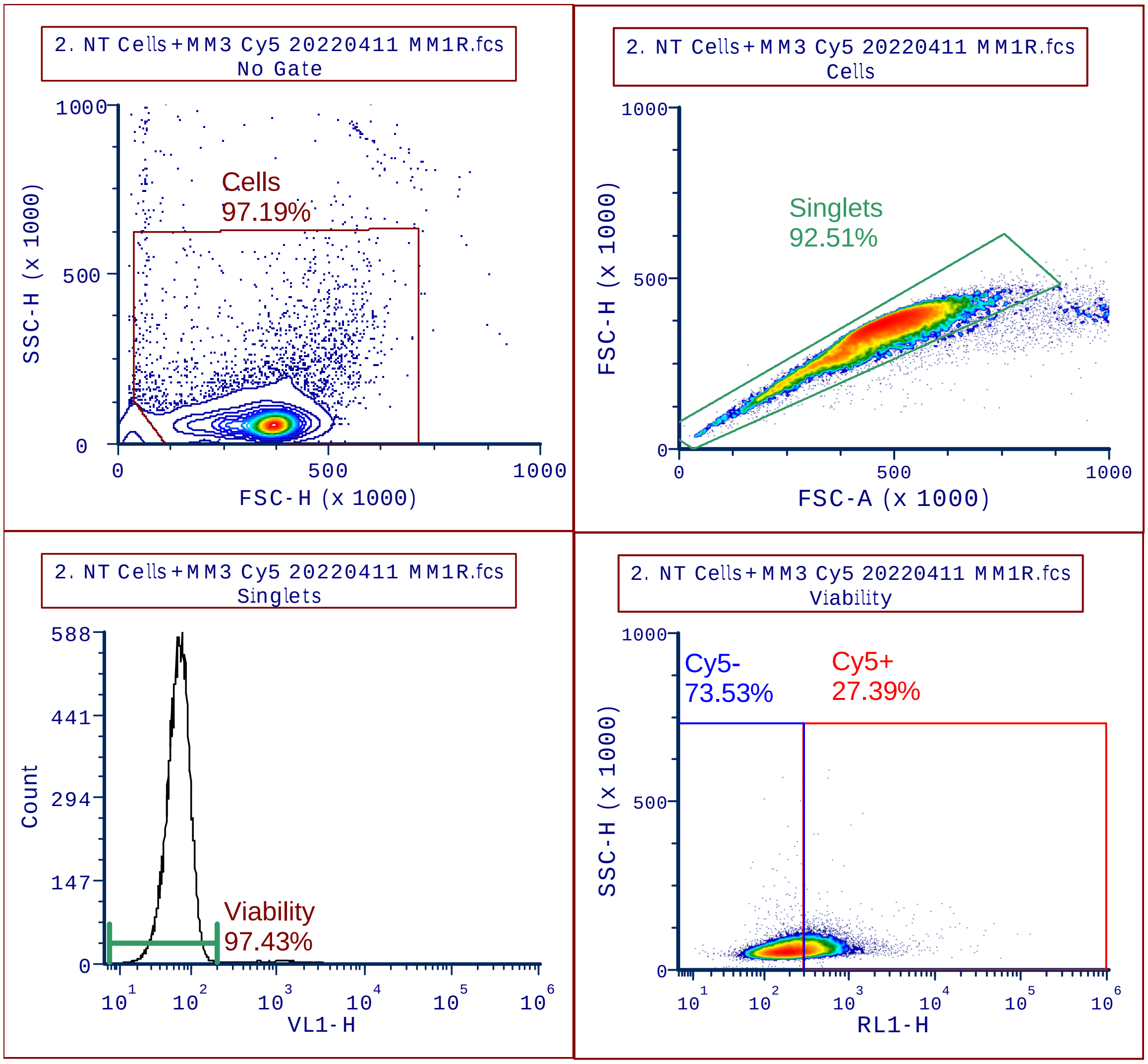

	A	B	C	D	E
1	**Filename**	**Gate**	**# of Events**	**% of all cells**	**% of gated cells**
2	2. NT Cells+M	All Events	41598	100	100
3	2. NT Cells+M	Cells	40431	97.19	97.19
4	2. NT Cells+M	Singlets	37404	89.92	92.51
5	2. NT Cells+M	Viability	36442	87.61	97.43
6	2. NT Cells+M	Cy5+	9980	23.99	27.39
7	2. NT Cells+M	Cy5-	26795	64.41	73.53
8					
9					

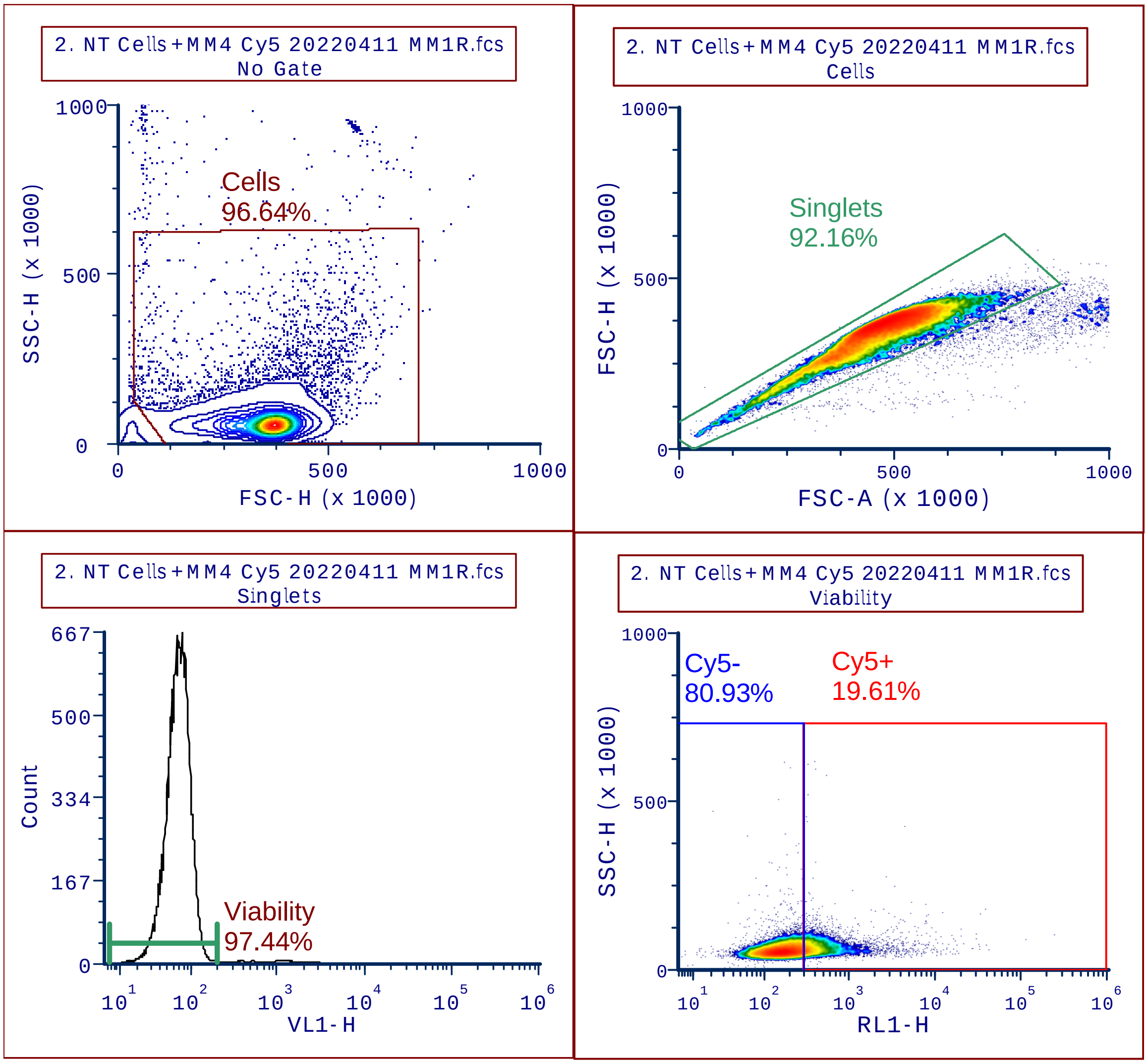

	A	B	C	D	E
1	Filename	**Gate**	**# of Events**	**% of all cells**	**% of gated cells**
2	2. NT Cells+M	All Events	48502	100	100
3	2. NT Cells+M	Cells	46874	96.64	96.64
4	2. NT Cells+M	Singlets	43201	89.07	92.16
5	2. NT Cells+M	Viability	42097	86.79	97.44
6	2. NT Cells+M	Cy5+	8257	17.02	19.61
7	2. NT Cells+M	Cy5-	34067	70.24	80.93
8					
9					

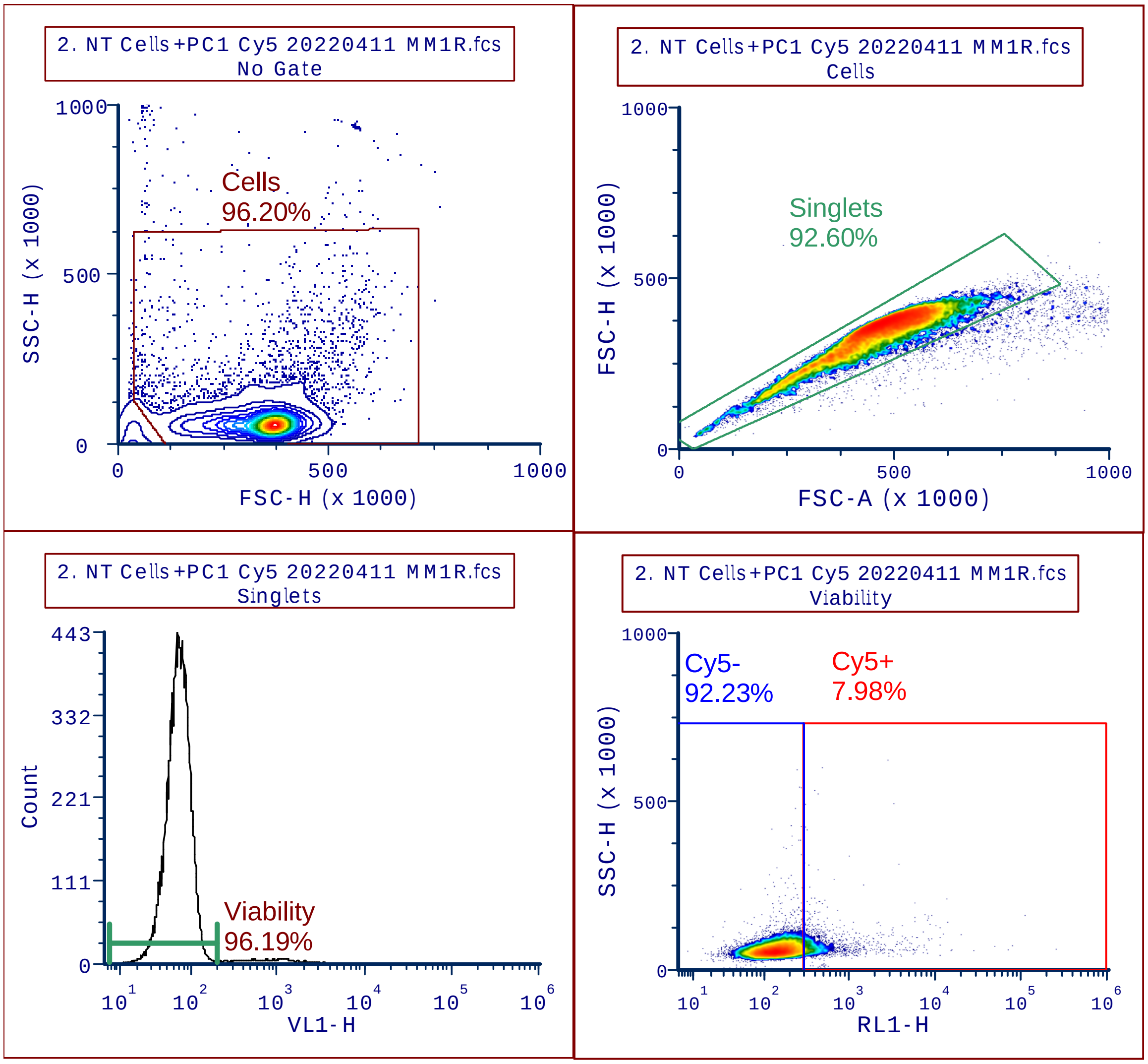

	A	B	C	D	E
1	Filename	Gate	# of Events	% of all cells	% of gated cells
2	2. NT Cells+P	All Events	32330	100	100
3	2. NT Cells+P	Cells	31103	96.2	96.2
4	2. NT Cells+P	Singlets	28801	89.08	92.6
5	2. NT Cells+P	Viability	27705	85.69	96.19
6	2. NT Cells+P	Cy5+	2212	6.84	7.98
7	2. NT Cells+P	Cy5-	25553	79.04	92.23
8					
9					

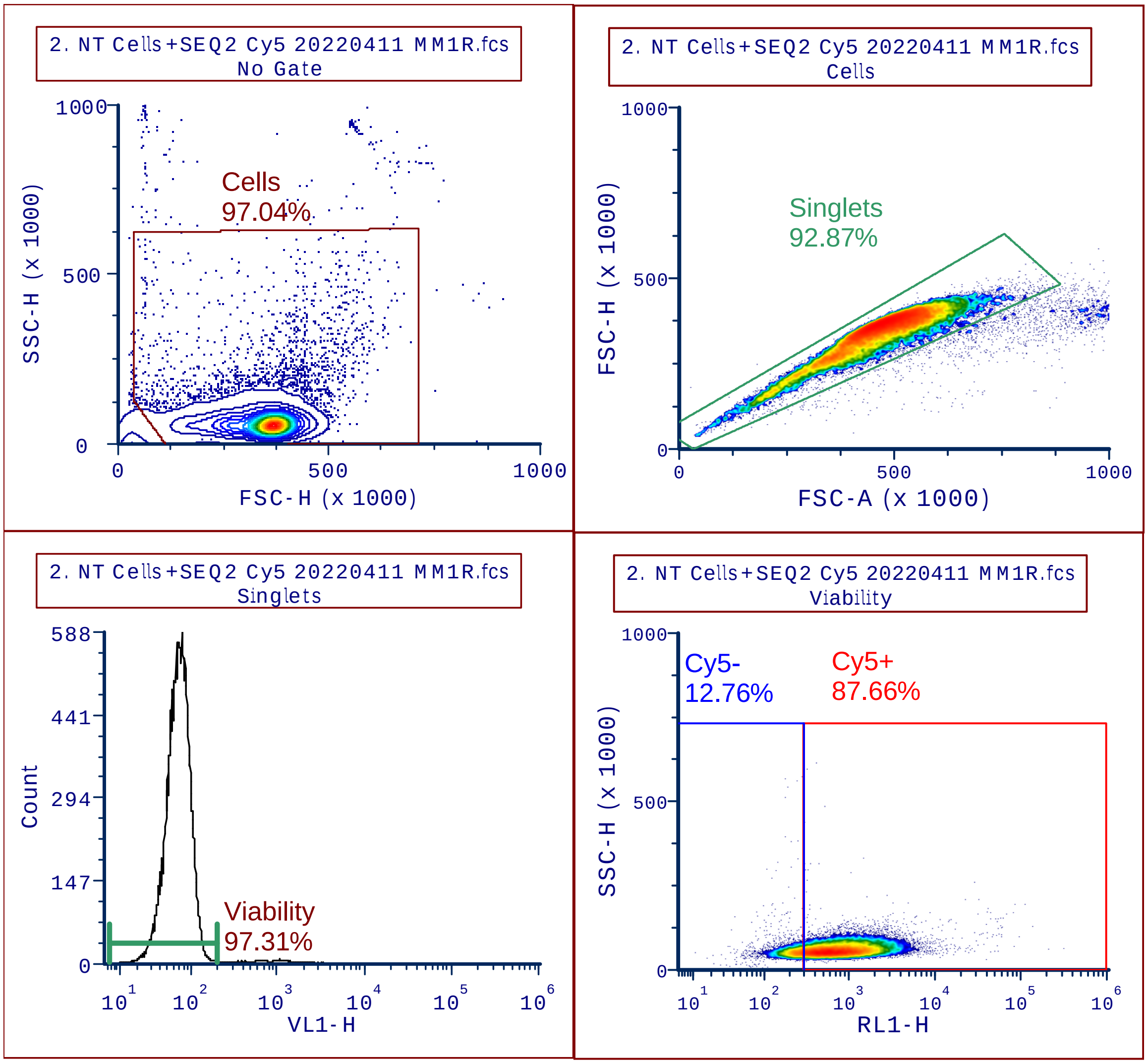

	A	B	C	D	E
1	**Filename**	**Gate**	**# of Events**	**% of all cells**	**% of gated cells**
2	2. NT Cells+S	All Events	42061	100	100
3	2. NT Cells+S	Cells	40818	97.04	97.04
4	2. NT Cells+S	Singlets	37908	90.13	92.87
5	2. NT Cells+S	Viability	36888	87.7	97.31
6	2. NT Cells+S	Cy5+	32335	76.88	87.66
7	2. NT Cells+S	Cy5-	4708	11.19	12.76
8					
9					

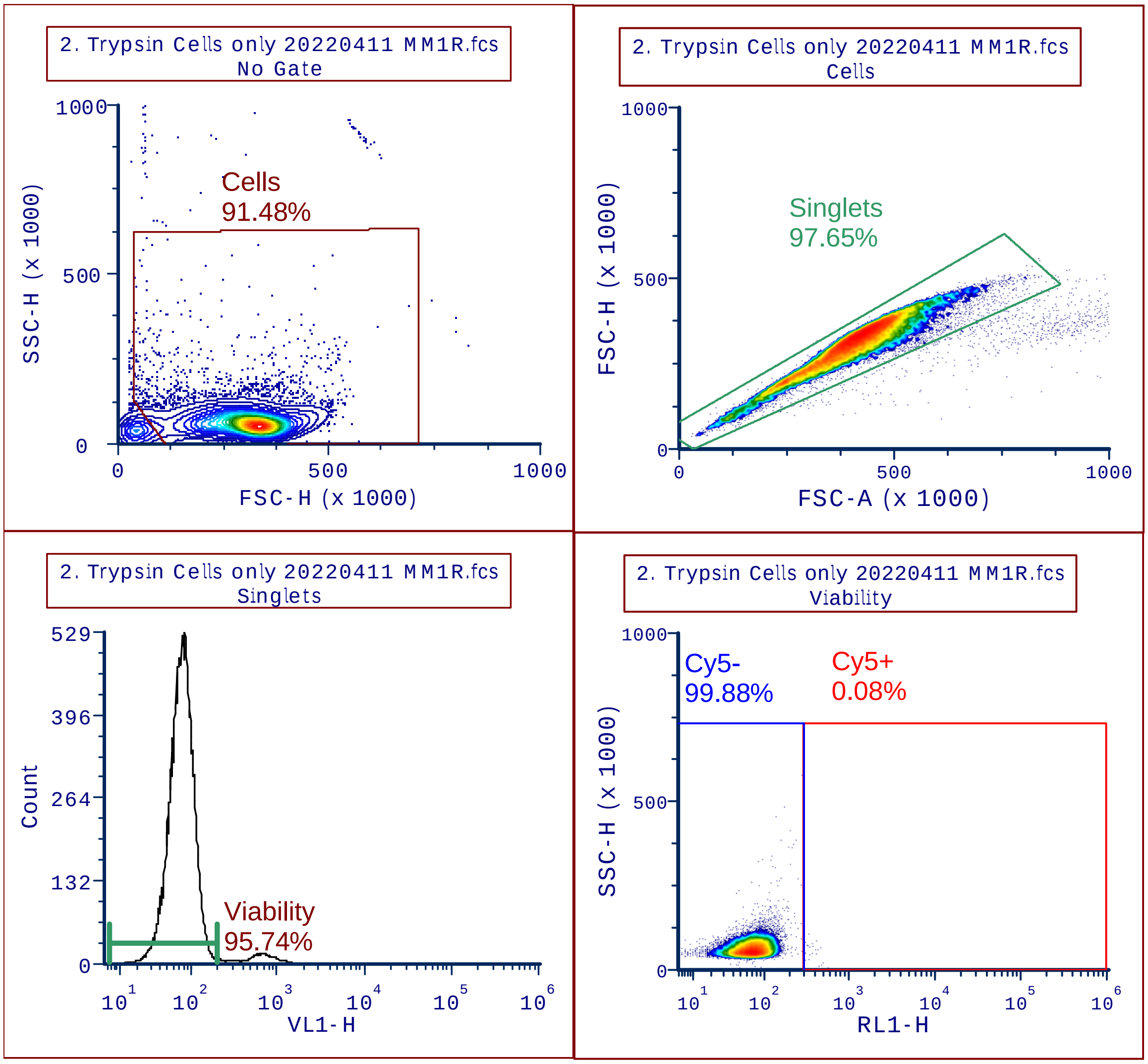

	A	B	C	D	E
1	Filename	Gate	# of Events	% of all cells	% of gated cells
2	2. Trypsin Ce	All Events	39465	100	100
3	2. Trypsin Ce	Cells	36104	91.48	91.48
4	2. Trypsin Ce	Singlets	35257	89.34	97.65
5	2. Trypsin Ce	Viability	33756	85.53	95.74
6	2. Trypsin Ce	Cy5+	27	0.07	0.08
7	2. Trypsin Ce	Cy5-	33716	85.43	99.88
8					
9					

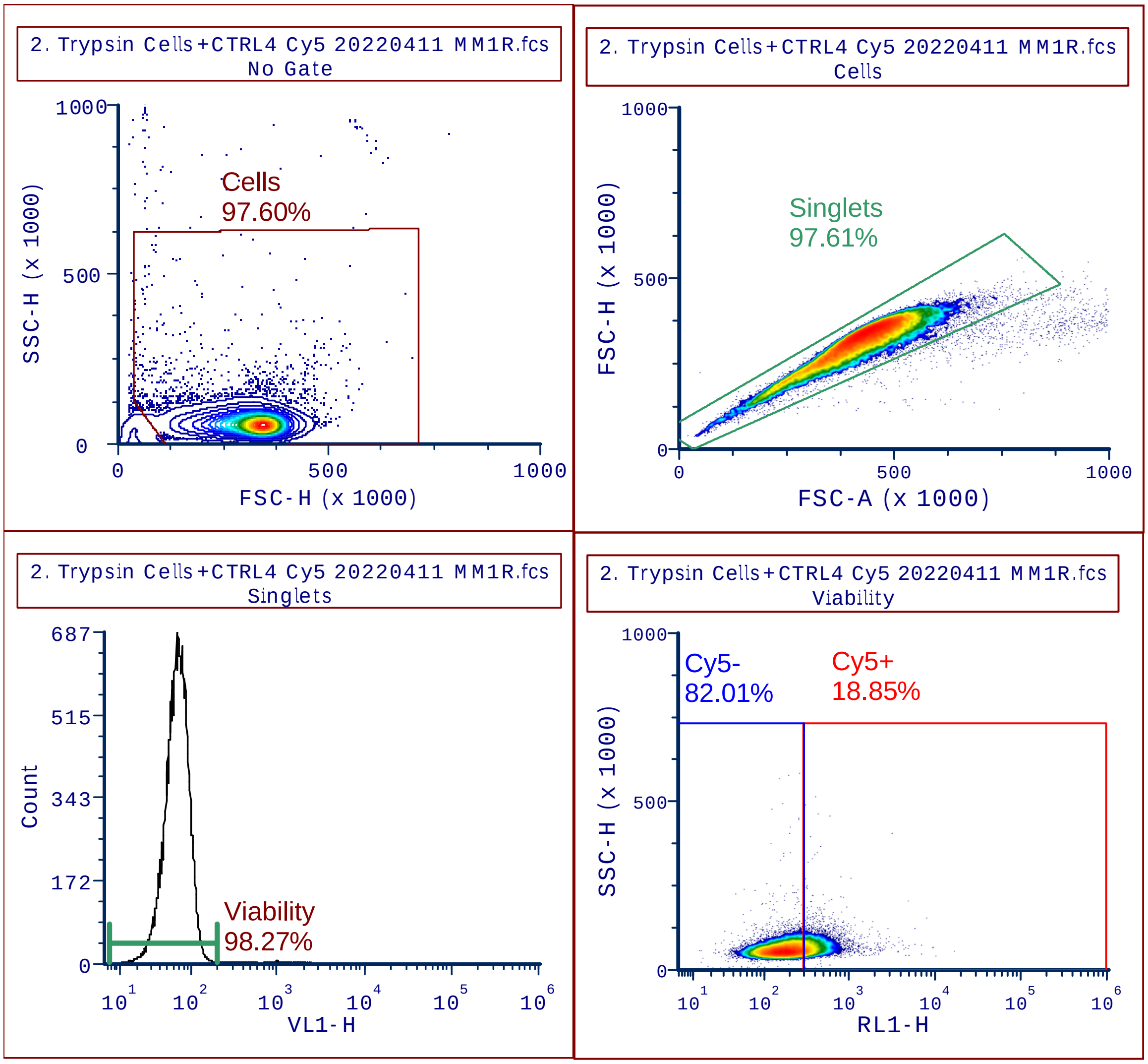

	A	B	C	D	E
1	**Filename**	**Gate**	**# of Events**	**% of all cells**	**% of gated cells**
2	2. Trypsin Ce	All Events	45392	100	100
3	2. Trypsin Ce	Cells	44303	97.6	97.6
4	2. Trypsin Ce	Singlets	43246	95.27	97.61
5	2. Trypsin Ce	Viability	42496	93.62	98.27
6	2. Trypsin Ce	Cy5+	8011	17.65	18.85
7	2. Trypsin Ce	Cy5-	34850	76.78	82.01
8					
9					

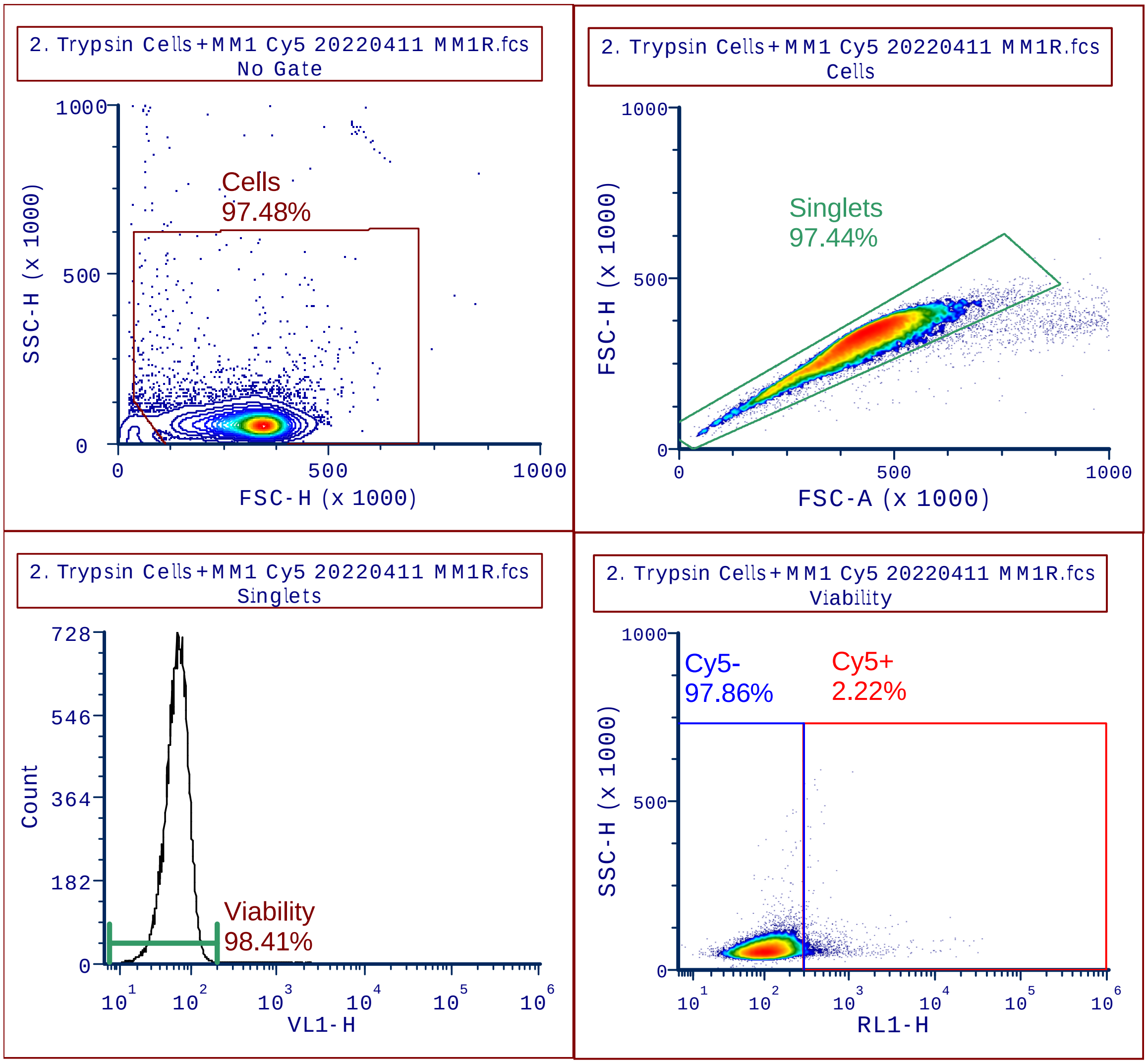

	A	B	C	D	E
1	Filename	Gate	# of Events	% of all cells	% of gated cells
2	2. Trypsin Ce	All Events	48270	100	100
3	2. Trypsin Ce	Cells	47054	97.48	97.48
4	2. Trypsin Ce	Singlets	45851	94.99	97.44
5	2. Trypsin Ce	Viability	45120	93.47	98.41
6	2. Trypsin Ce	Cy5+	1000	2.07	2.22
7	2. Trypsin Ce	Cy5-	44156	91.48	97.86
8					
9					

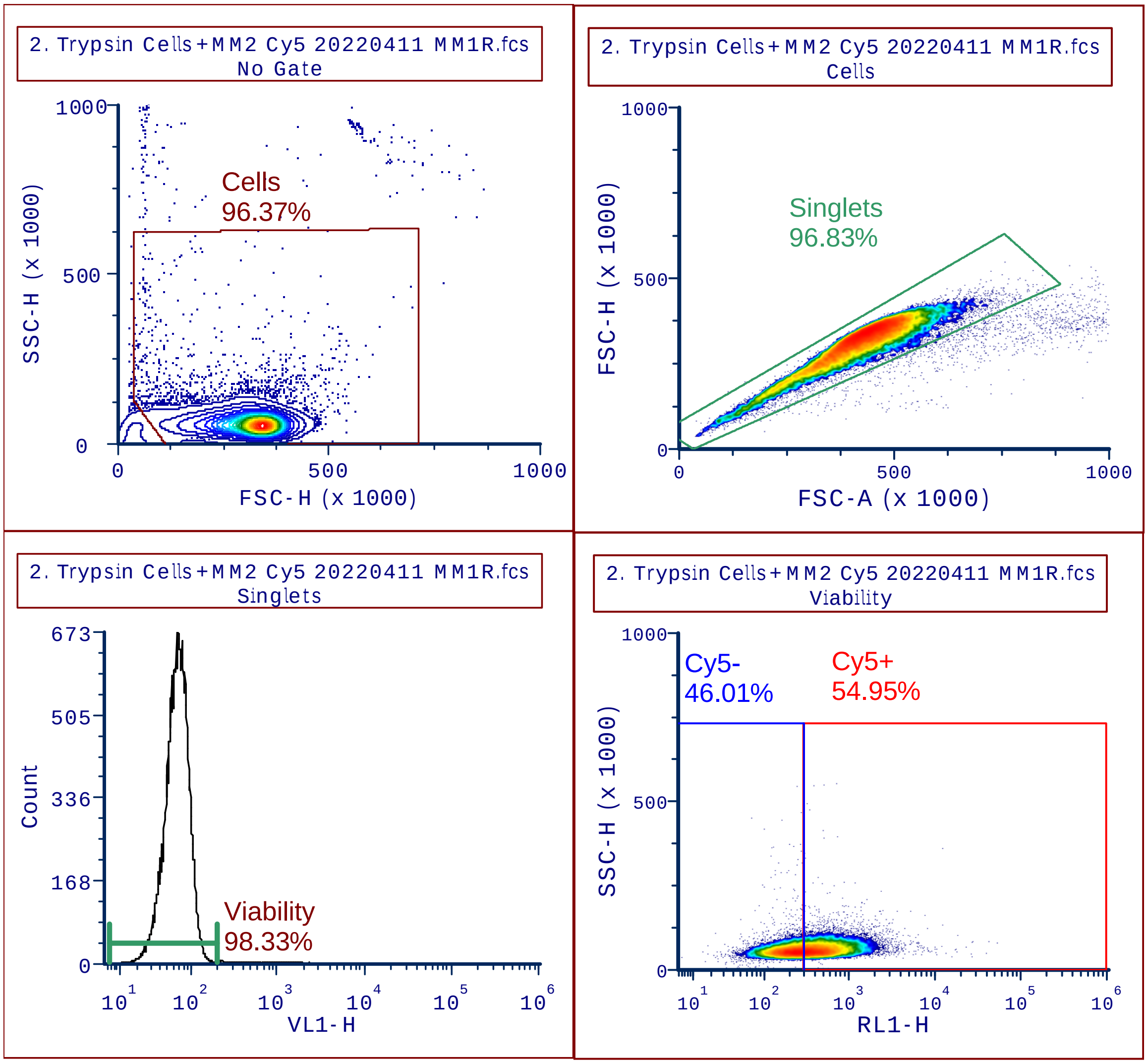

	A	B	C	D	E
1	**Filename**	**Gate**	**# of Events**	**% of all cells**	**% of gated cells**
2	2. Trypsin Ce	All Events	45906	100	100
3	2. Trypsin Ce	Cells	44240	96.37	96.37
4	2. Trypsin Ce	Singlets	42836	93.31	96.83
5	2. Trypsin Ce	Viability	42121	91.75	98.33
6	2. Trypsin Ce	Cy5+	23146	50.42	54.95
7	2. Trypsin Ce	Cy5-	19381	42.22	46.01
8					
9					

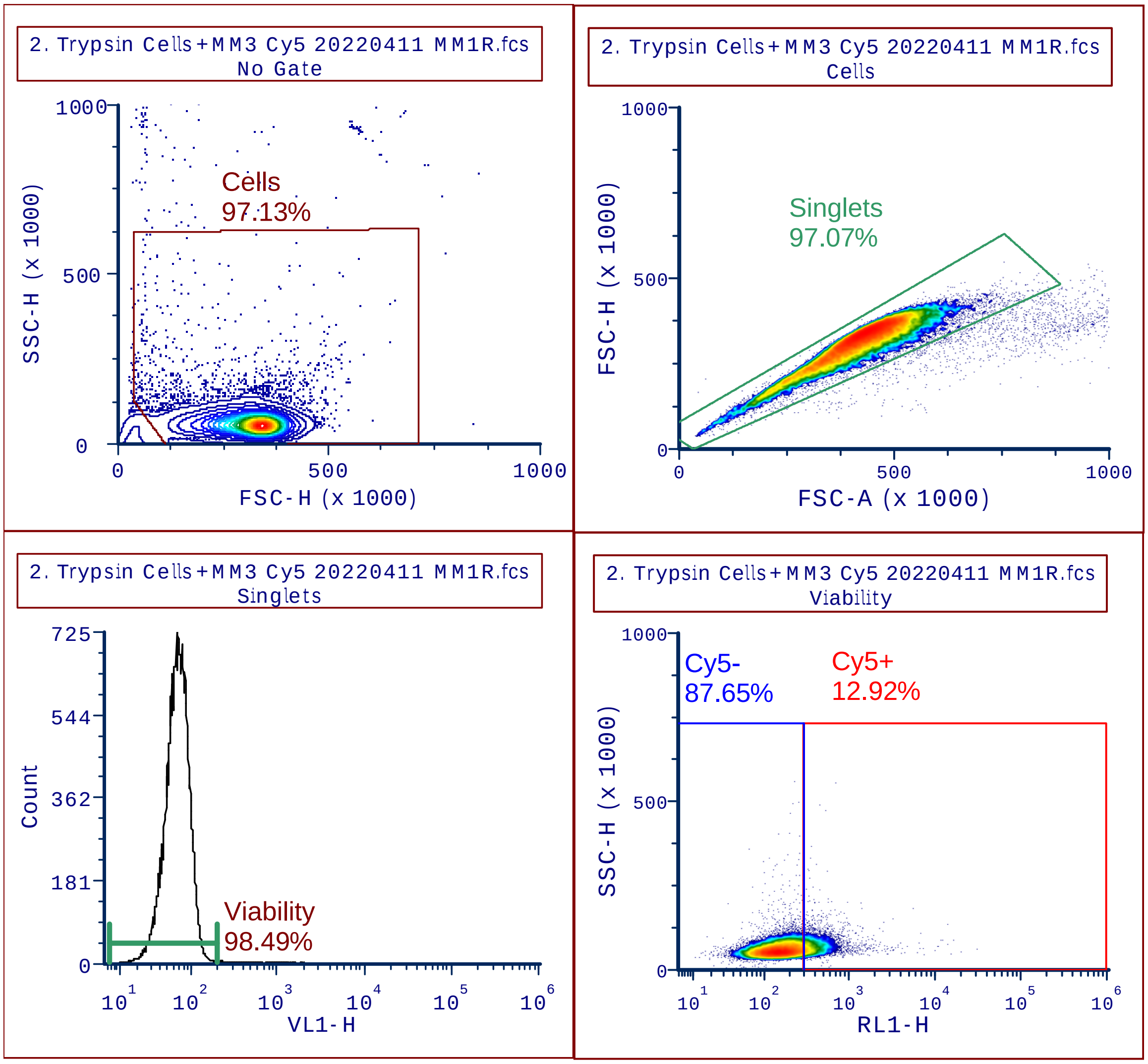

	A	B	C	D	E
1	**Filename**	**Gate**	**# of Events**	**% of all cells**	**% of gated cells**
2	2. Trypsin Ce	All Events	49044	100	100
3	2. Trypsin Ce	Cells	47638	97.13	97.13
4	2. Trypsin Ce	Singlets	46240	94.28	97.07
5	2. Trypsin Ce	Viability	45540	92.86	98.49
6	2. Trypsin Ce	Cy5+	5885	12	12.92
7	2. Trypsin Ce	Cy5-	39917	81.39	87.65
8					
9					

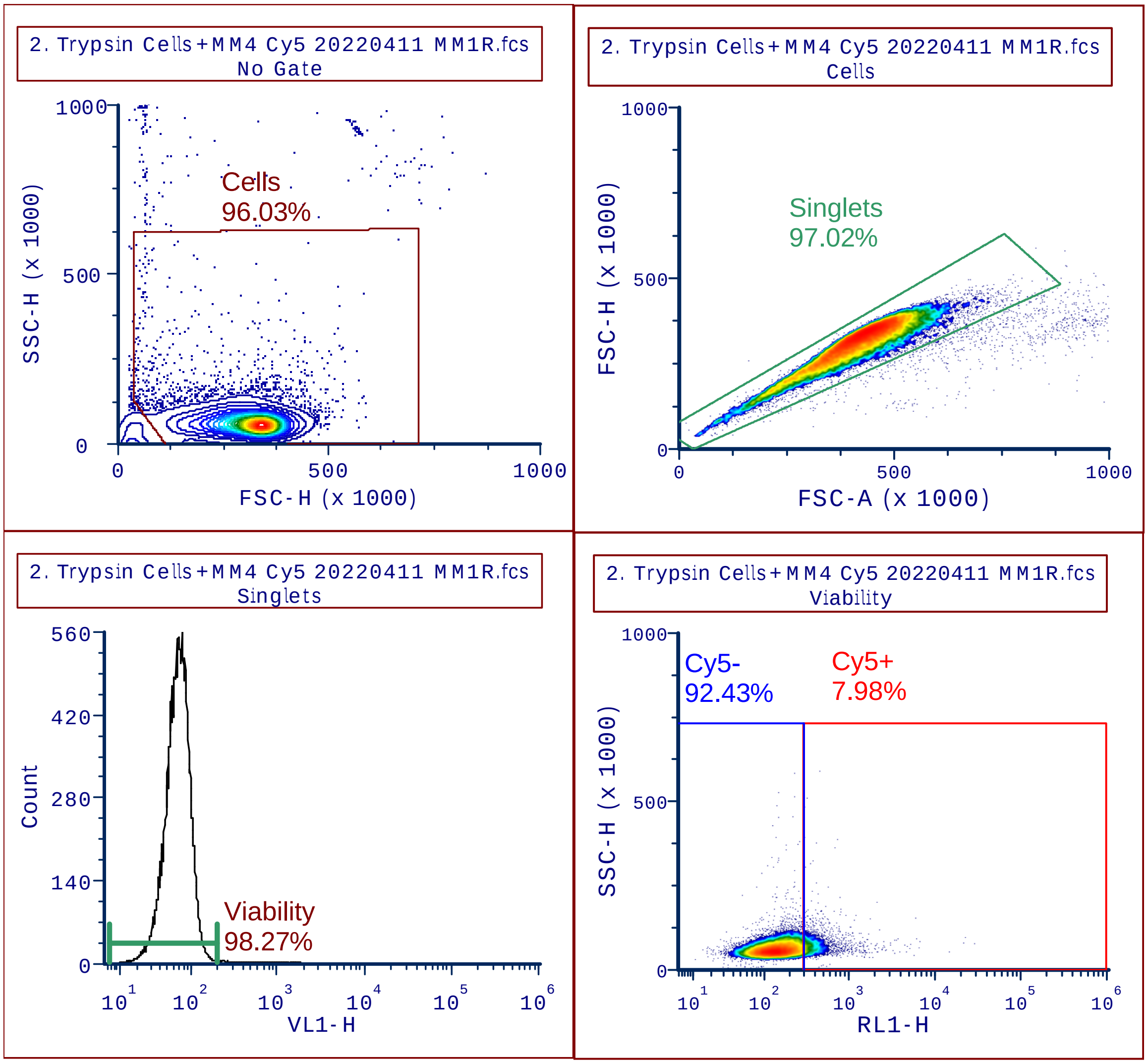

	A	B	C	D	E
1	Filename	Gate	# of Events	% of all cells	% of gated cells
2	2. Trypsin Ce	All Events	39071	100	100
3	2. Trypsin Ce	Cells	37520	96.03	96.03
4	2. Trypsin Ce	Singlets	36402	93.17	97.02
5	2. Trypsin Ce	Viability	35773	91.56	98.27
6	2. Trypsin Ce	Cy5+	2856	7.31	7.98
7	2. Trypsin Ce	Cy5-	33066	84.63	92.43
8					
9					

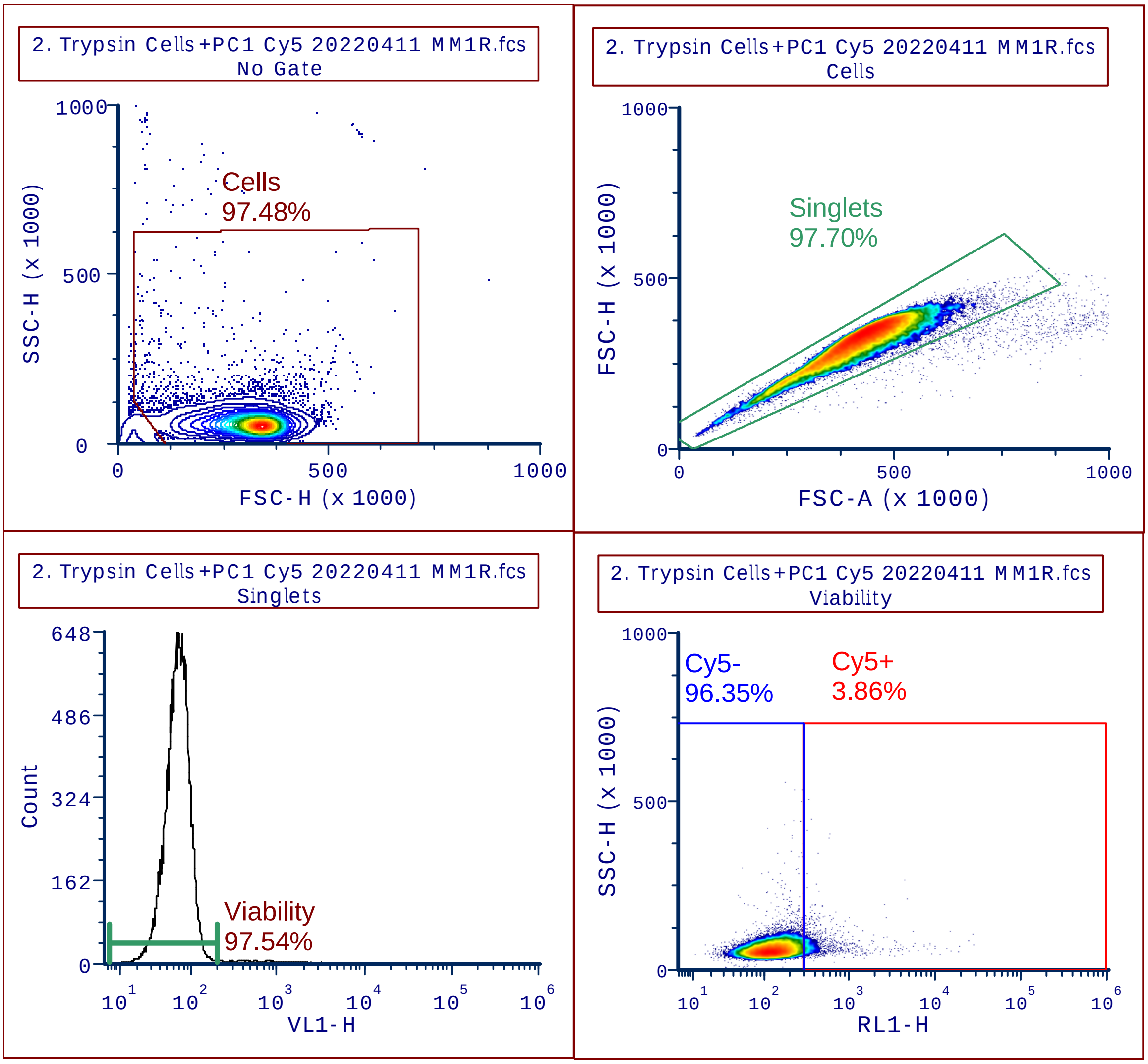

	A	B	C	D	E
1	Filename	**Gate**	**# of Events**	**% of all cells**	**% of gated cells**
2	2. Trypsin Ce	All Events	44377	100	100
3	2. Trypsin Ce	Cells	43258	97.48	97.48
4	2. Trypsin Ce	Singlets	42265	95.24	97.7
5	2. Trypsin Ce	Viability	41224	92.89	97.54
6	2. Trypsin Ce	Cy5+	1593	3.59	3.86
7	2. Trypsin Ce	Cy5-	39718	89.5	96.35
8					
9					

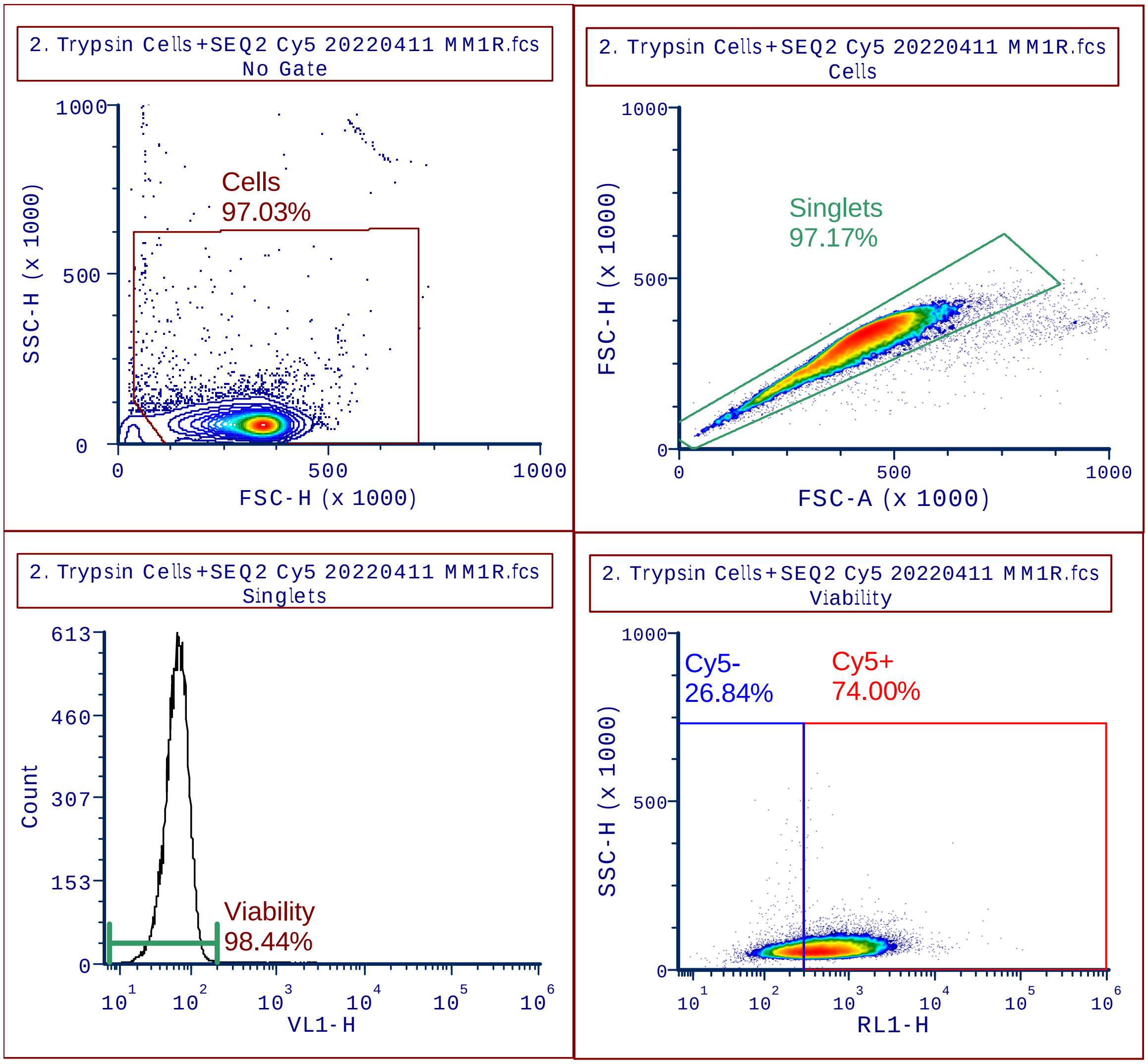

	A	B	C	D	E
1	**Filename**	**Gate**	**# of Events**	**% of all cells**	**% of gated cells**
2	2. Trypsin Ce	All Events	41095	100	100
3	2. Trypsin Ce	Cells	39875	97.03	97.03
4	2. Trypsin Ce	Singlets	38747	94.29	97.17
5	2. Trypsin Ce	Viability	38142	92.81	98.44
6	2. Trypsin Ce	Cy5+	28224	68.68	74
7	2. Trypsin Ce	Cy5-	10237	24.91	26.84
8					
9					

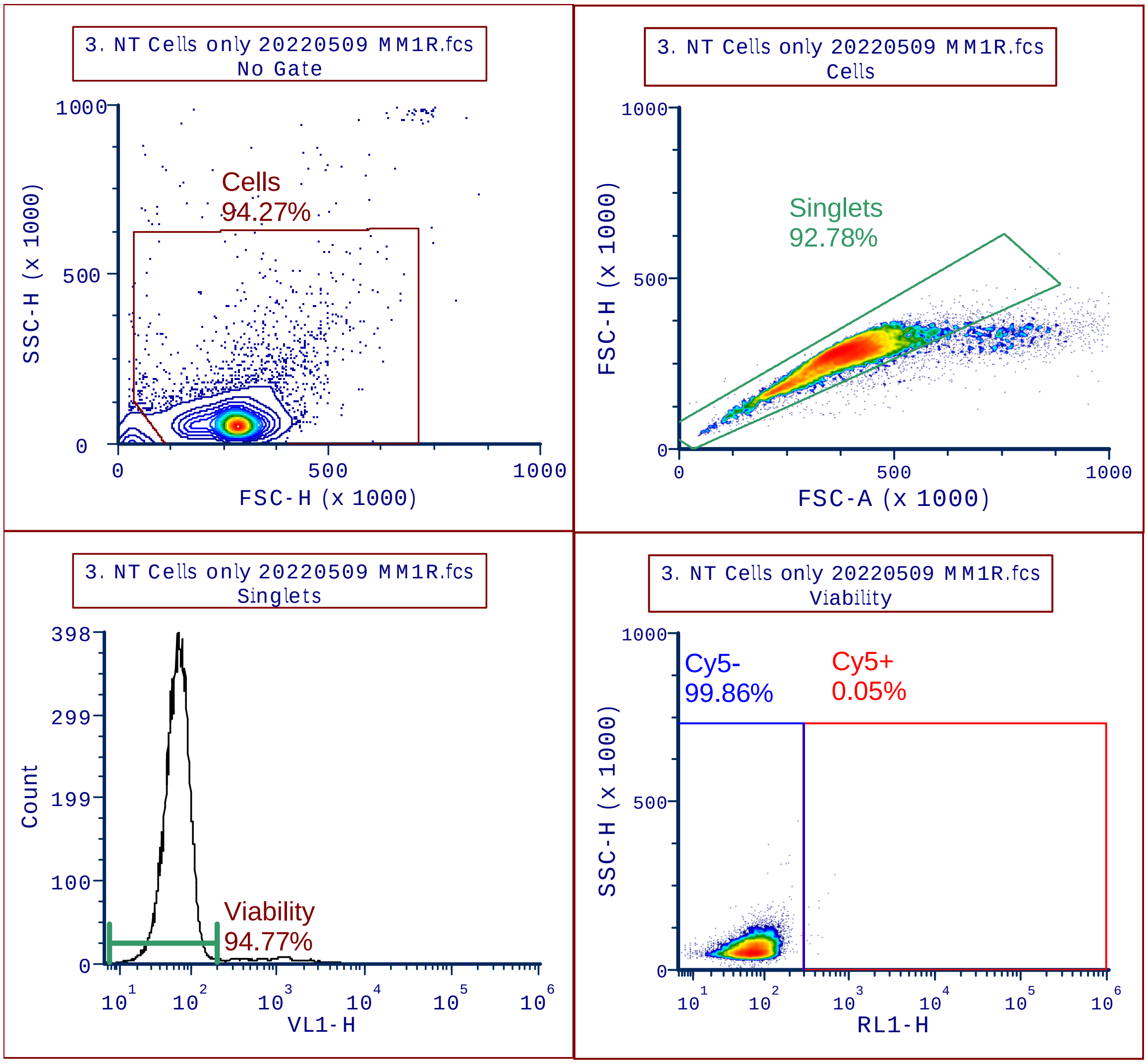

	A	B	C	D	E
1	Filename	Gate	# of Events	% of all cells	% of gated cells
2	3. NT Cells on	All Events	31376	100	100
3	3. NT Cells on	Cells	29577	94.27	94.27
4	3. NT Cells on	Singlets	27443	87.46	92.78
5	3. NT Cells on	Viability	26007	82.89	94.77
6	3. NT Cells on	Cy5+	12	0.04	0.05
7	3. NT Cells on	Cy5-	25971	82.77	99.86
8					
9					

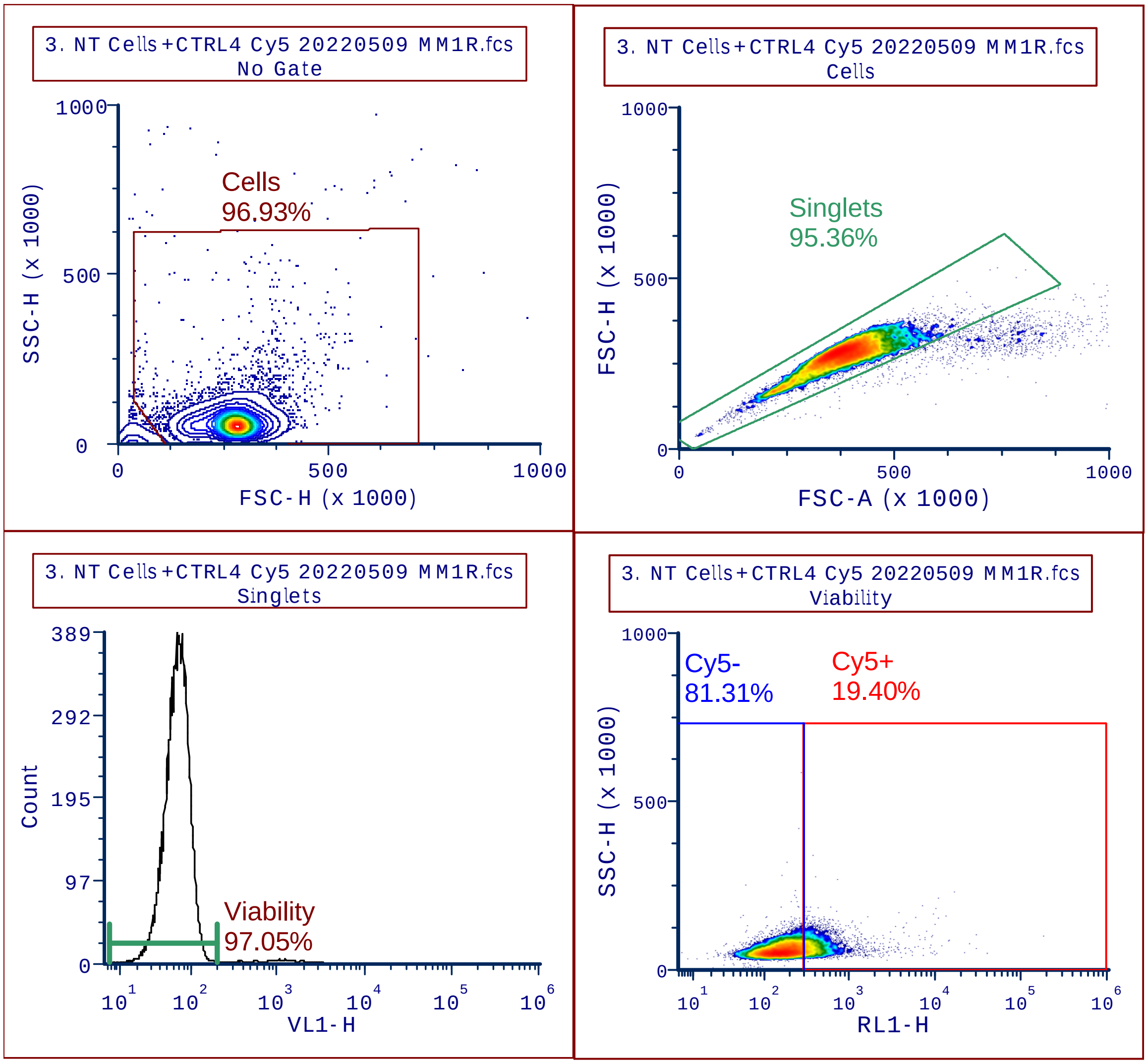

	A	B	C	D	E
1	**Filename**	**Gate**	**# of Events**	**% of all cells**	**% of gated cells**
2	3. NT Cells+C	All Events	28202	100	100
3	3. NT Cells+C	Cells	27336	96.93	96.93
4	3. NT Cells+C	Singlets	26068	92.43	95.36
5	3. NT Cells+C	Viability	25298	89.7	97.05
6	3. NT Cells+C	Cy5+	4908	17.4	19.4
7	3. NT Cells+C	Cy5-	20571	72.94	81.31
8					
9					

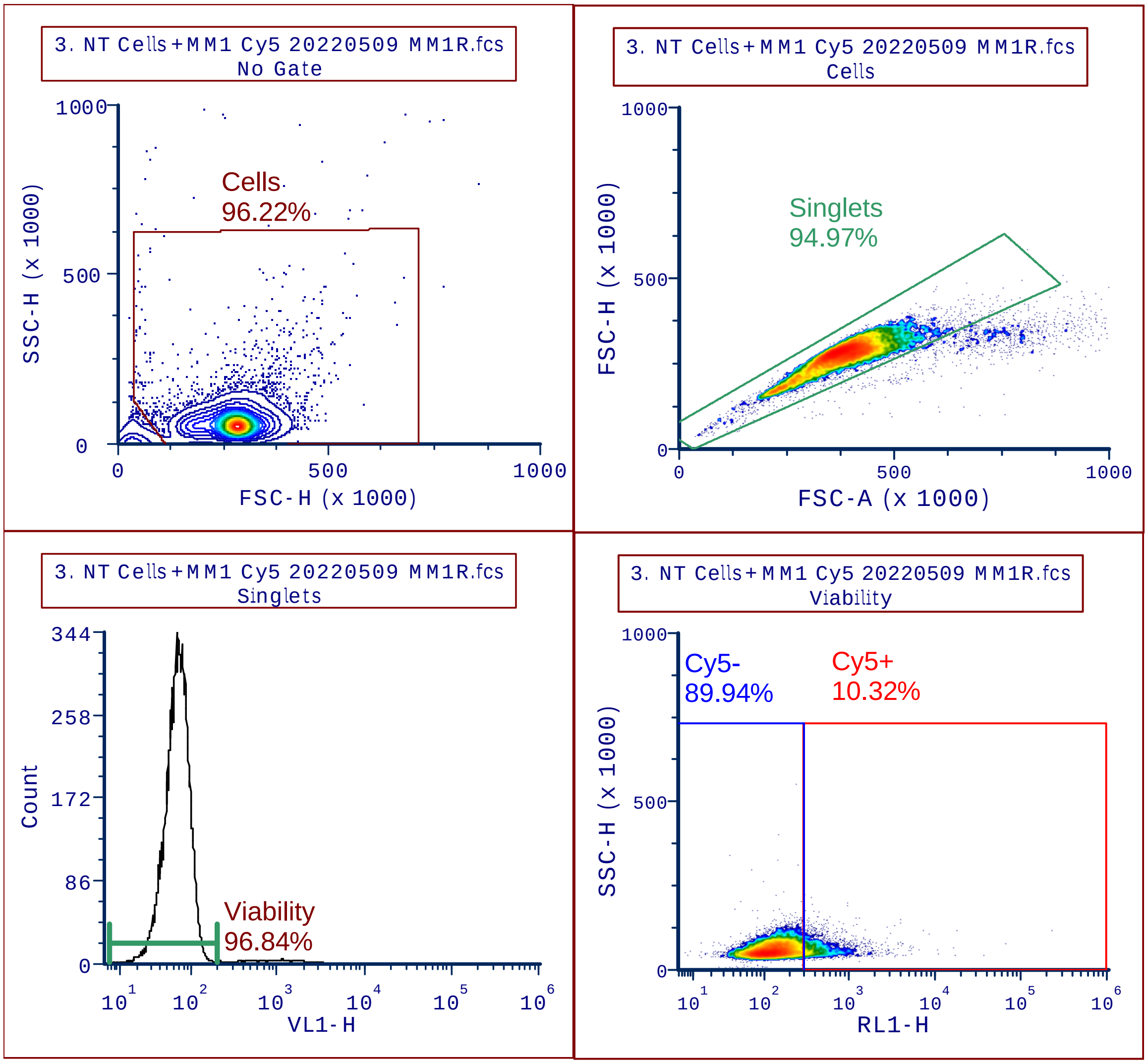

	A	B	C	D	E
1	**Filename**	**Gate**	**# of Events**	**% of all cells**	**% of gated cells**
2	3. NT Cells+M	All Events	24346	100	100
3	3. NT Cells+M	Cells	23426	96.22	96.22
4	3. NT Cells+M	Singlets	22247	91.38	94.97
5	3. NT Cells+M	Viability	21544	88.49	96.84
6	3. NT Cells+M	Cy5+	2224	9.13	10.32
7	3. NT Cells+M	Cy5-	19377	79.59	89.94
8					
9					

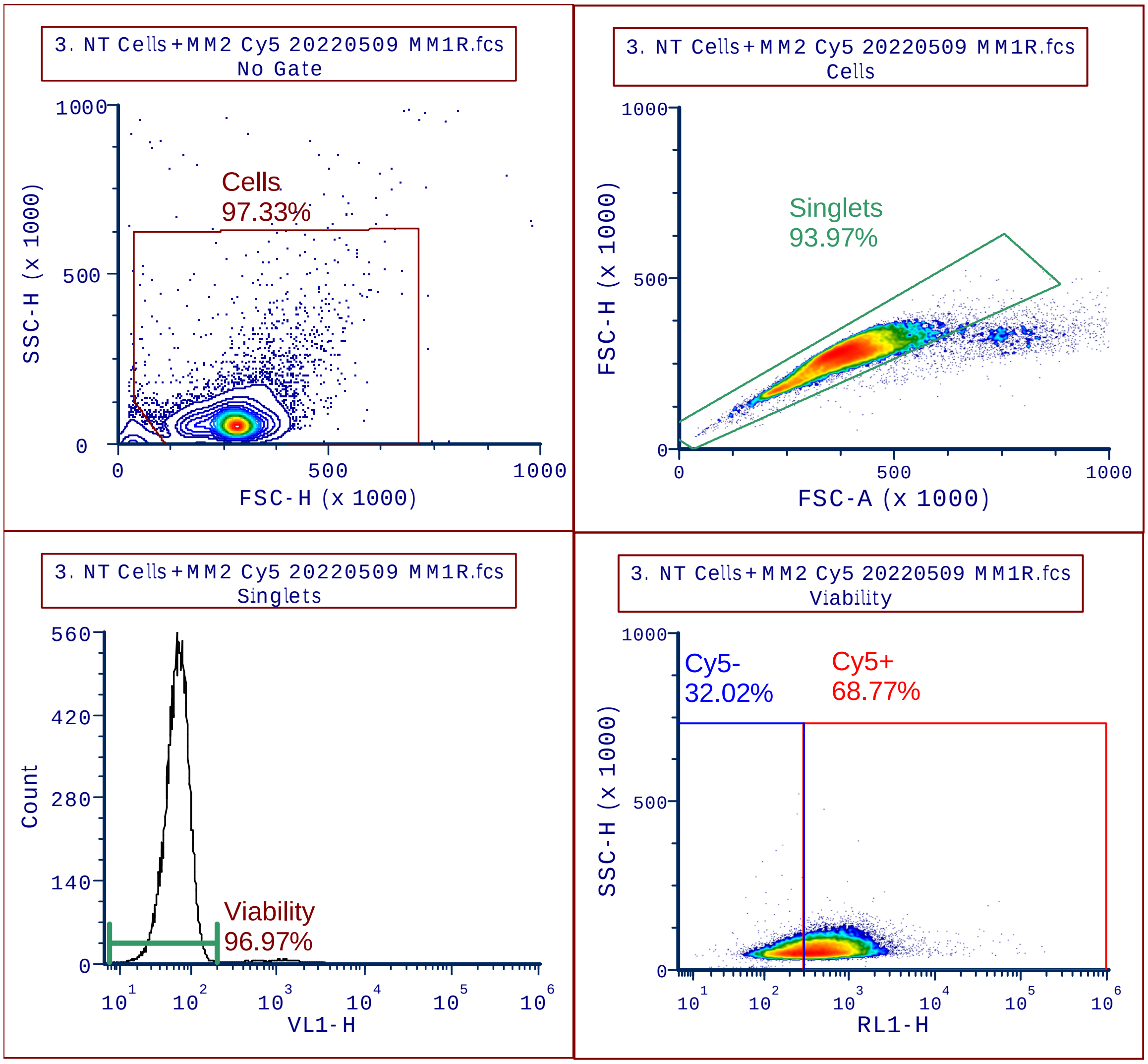

	A	B	C	D	E
1	**Filename**	**Gate**	**# of Events**	**% of all cells**	**% of gated cells**
2	3. NT Cells+M	All Events	39756	100	100
3	3. NT Cells+M	Cells	38694	97.33	97.33
4	3. NT Cells+M	Singlets	36361	91.46	93.97
5	3. NT Cells+M	Viability	35258	88.69	96.97
6	3. NT Cells+M	Cy5+	24246	60.99	68.77
7	3. NT Cells+M	Cy5-	11290	28.4	32.02
8					
9					

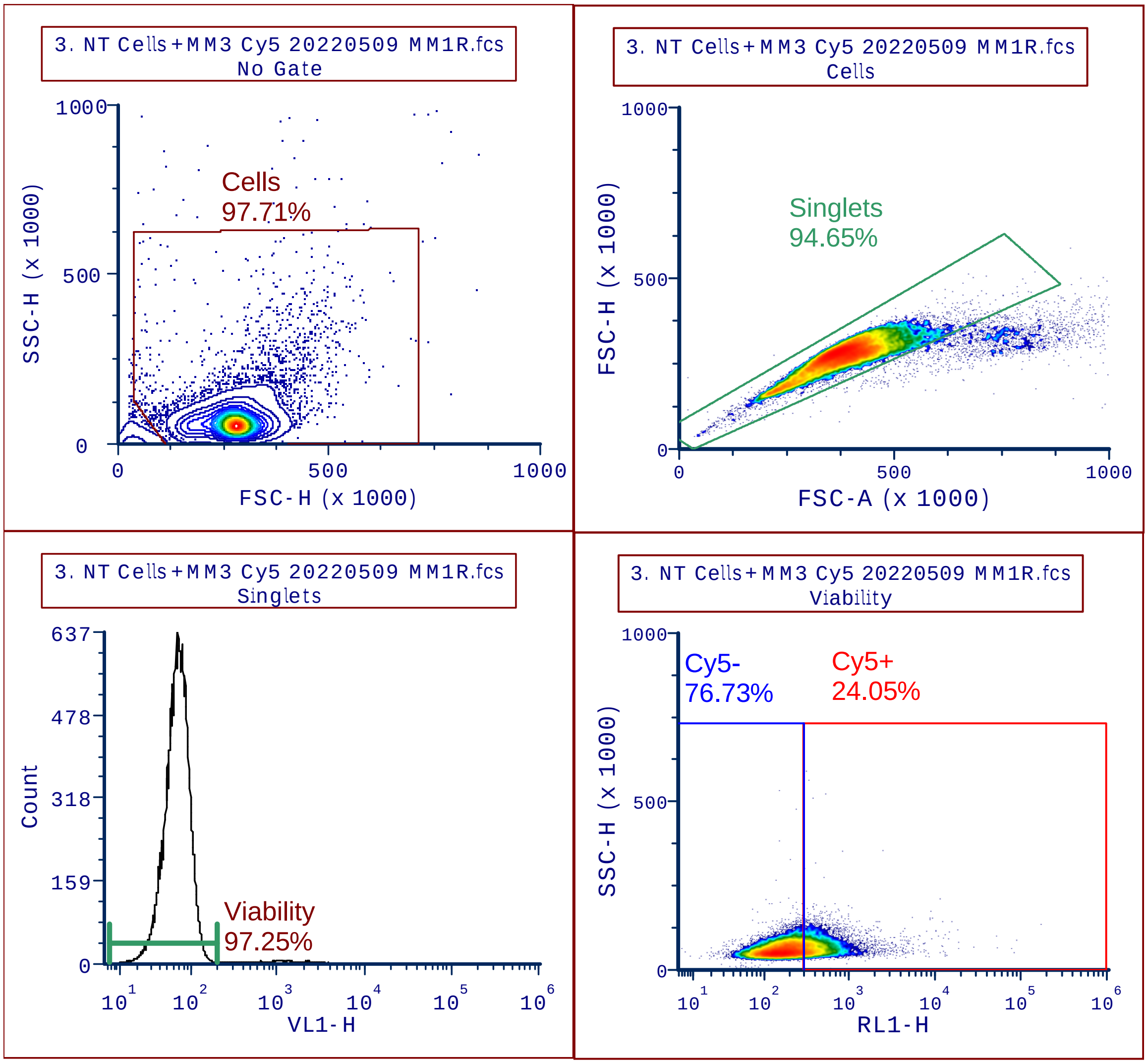

	A	B	C	D	E
1	Filename	Gate	# of Events	% of all cells	% of gated cells
2	3. NT Cells+M	All Events	44523	100	100
3	3. NT Cells+M	Cells	43505	97.71	97.71
4	3. NT Cells+M	Singlets	41178	92.49	94.65
5	3. NT Cells+M	Viability	40047	89.95	97.25
6	3. NT Cells+M	Cy5+	9631	21.63	24.05
7	3. NT Cells+M	Cy5-	30729	69.02	76.73
8					
9					

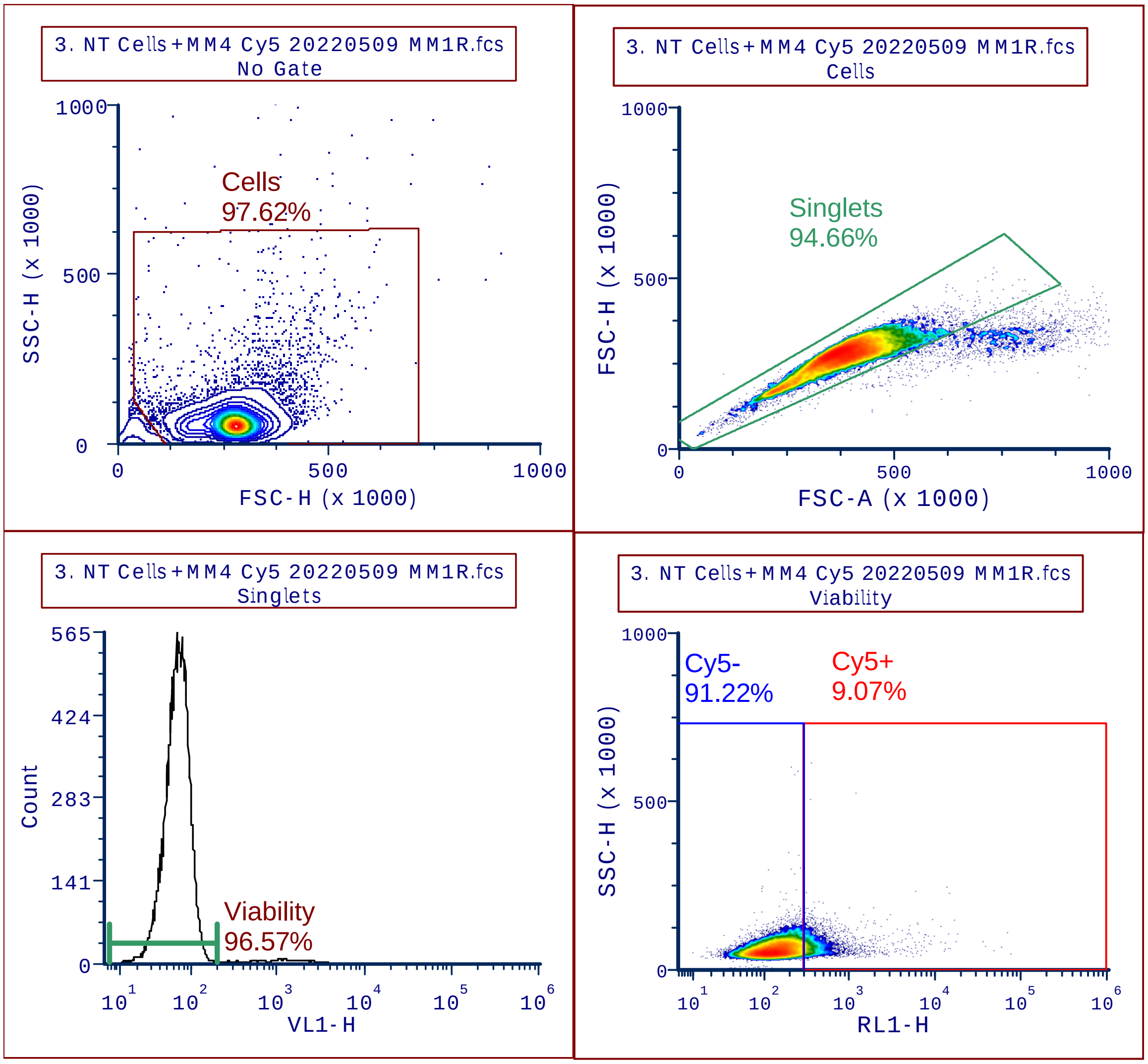

	A	B	C	D	E
1	**Filename**	**Gate**	**# of Events**	**% of all cells**	**% of gated cells**
2	3. NT Cells+M	All Events	40874	100	100
3	3. NT Cells+M	Cells	39900	97.62	97.62
4	3. NT Cells+M	Singlets	37768	92.4	94.66
5	3. NT Cells+M	Viability	36474	89.24	96.57
6	3. NT Cells+M	Cy5+	3309	8.1	9.07
7	3. NT Cells+M	Cy5-	33271	81.4	91.22
8					
9					

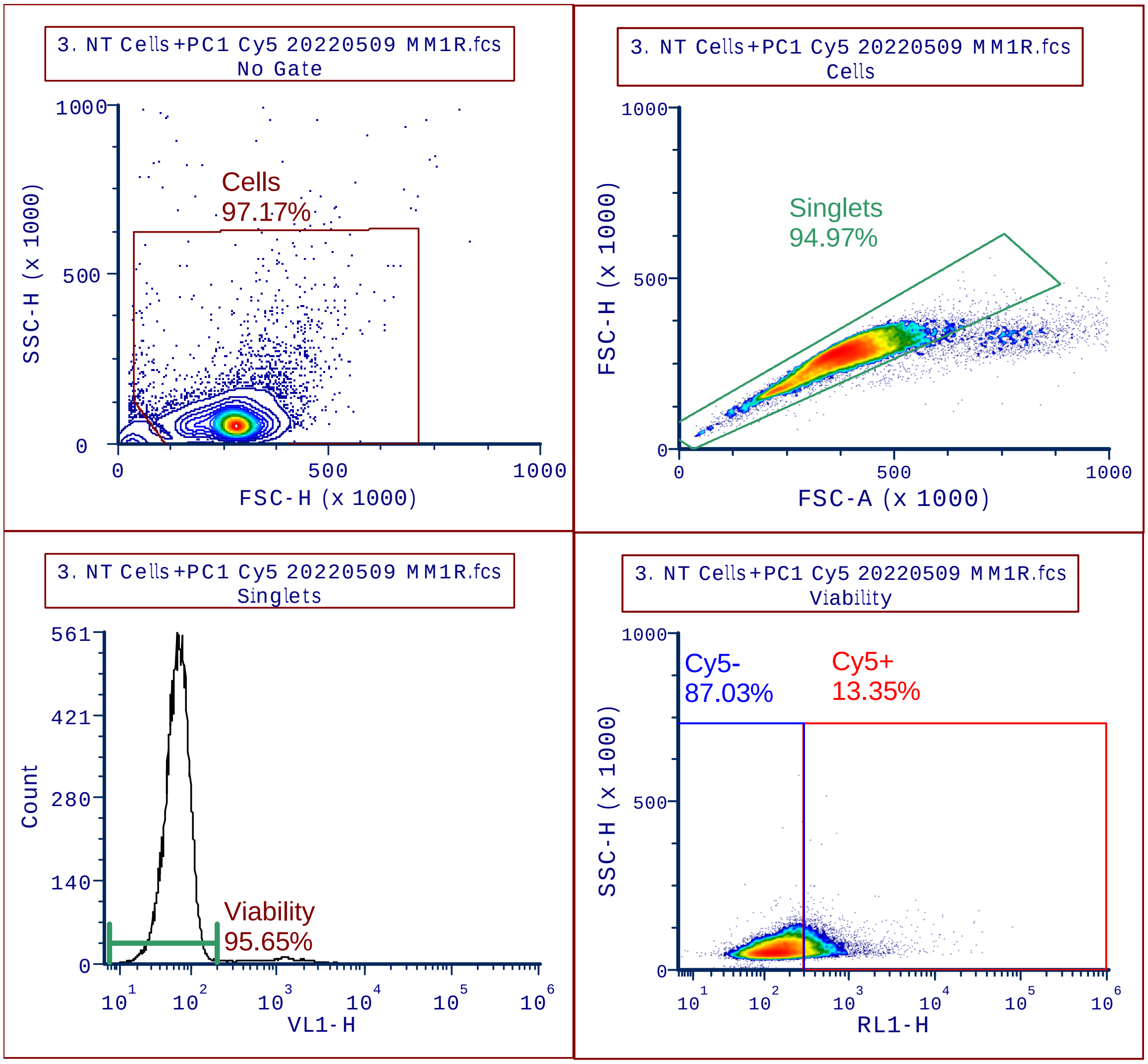

	A	B	C	D	E
1	**Filename**	**Gate**	**# of Events**	**% of all cells**	**% of gated cells**
2	3. NT Cells+P	All Events	41454	100	100
3	3. NT Cells+P	Cells	40282	97.17	97.17
4	3. NT Cells+P	Singlets	38256	92.29	94.97
5	3. NT Cells+P	Viability	36592	88.27	95.65
6	3. NT Cells+P	Cy5+	4885	11.78	13.35
7	3. NT Cells+P	Cy5-	31845	76.82	87.03
8					
9					

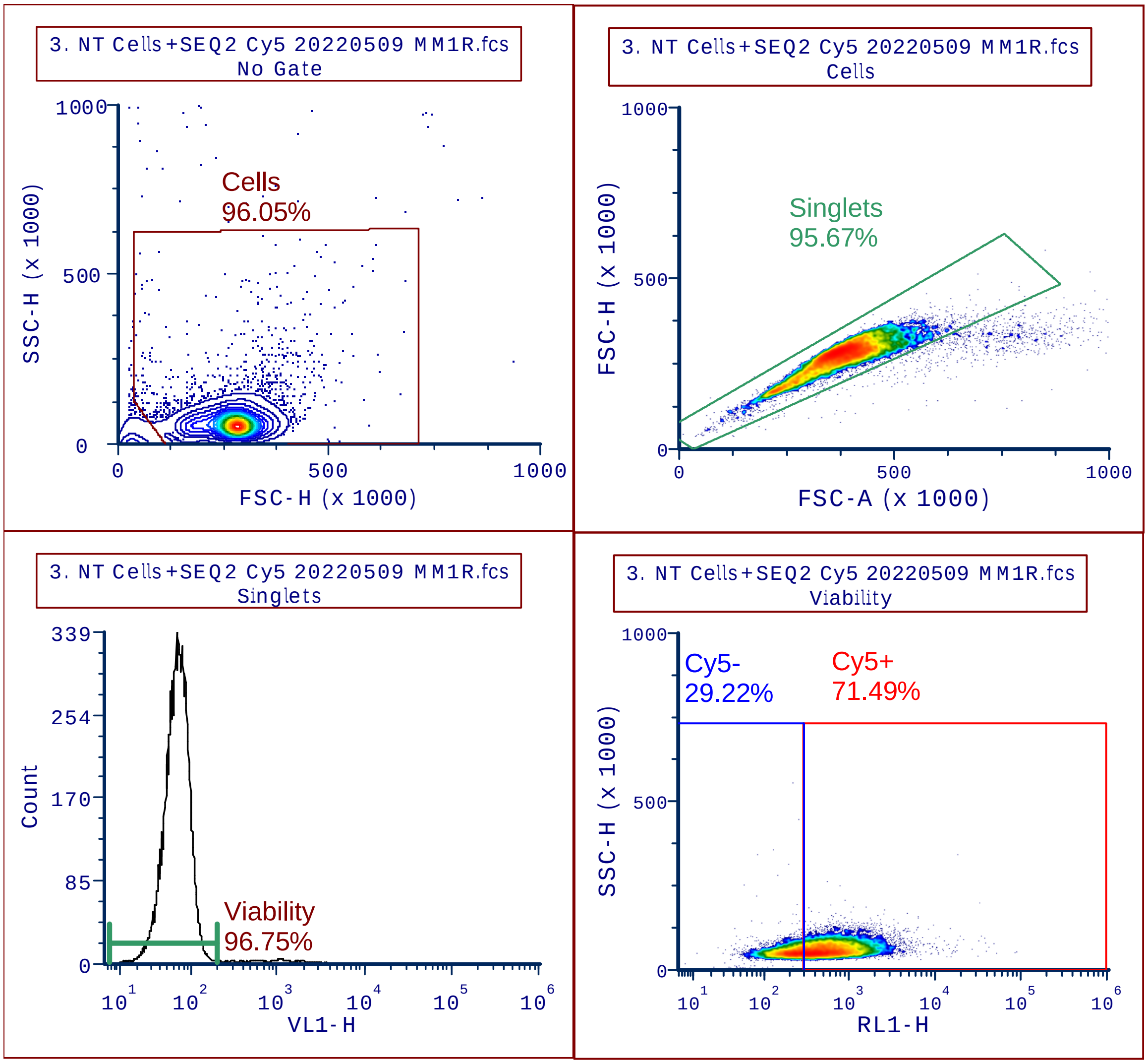

	A	B	C	D	E
1	Filename	Gate	# of Events	% of all cells	% of gated cells
2	3. NT Cells+S	All Events	24548	100	100
3	3. NT Cells+S	Cells	23579	96.05	96.05
4	3. NT Cells+S	Singlets	22557	91.89	95.67
5	3. NT Cells+S	Viability	21824	88.9	96.75
6	3. NT Cells+S	Cy5+	15601	63.55	71.49
7	3. NT Cells+S	Cy5-	6377	25.98	29.22
8					
9					

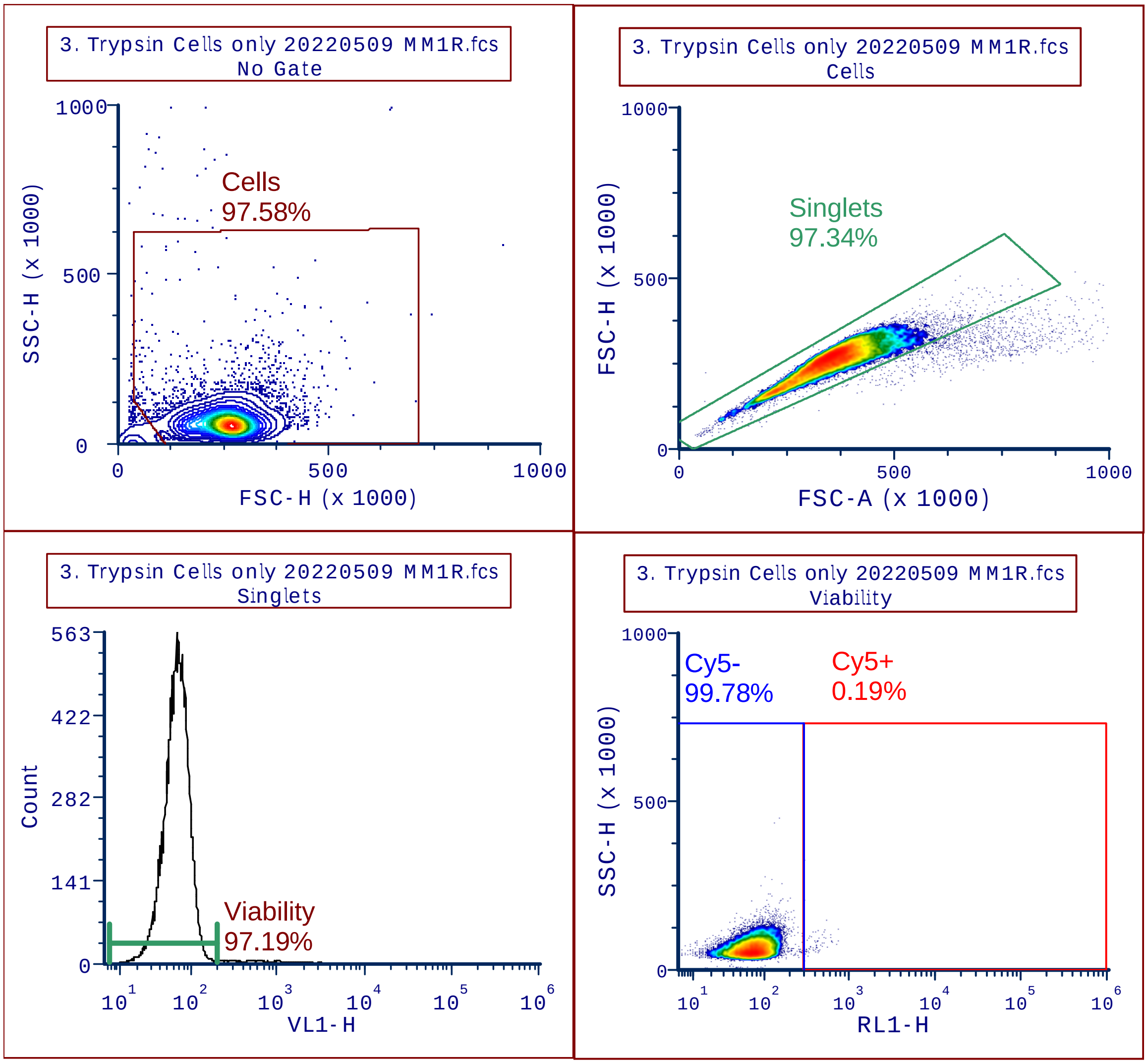

Filename	Gate	# of Events	% of all cells	% of gated cells
3. Trypsin Ce	All Events	38384	100	100
3. Trypsin Ce	Cells	37455	97.58	97.58
3. Trypsin Ce	Singlets	36458	94.98	97.34
3. Trypsin Ce	Viability	35432	92.31	97.19
3. Trypsin Ce	Cy5+	66	0.17	0.19
3. Trypsin Ce	Cy5-	35353	92.1	99.78

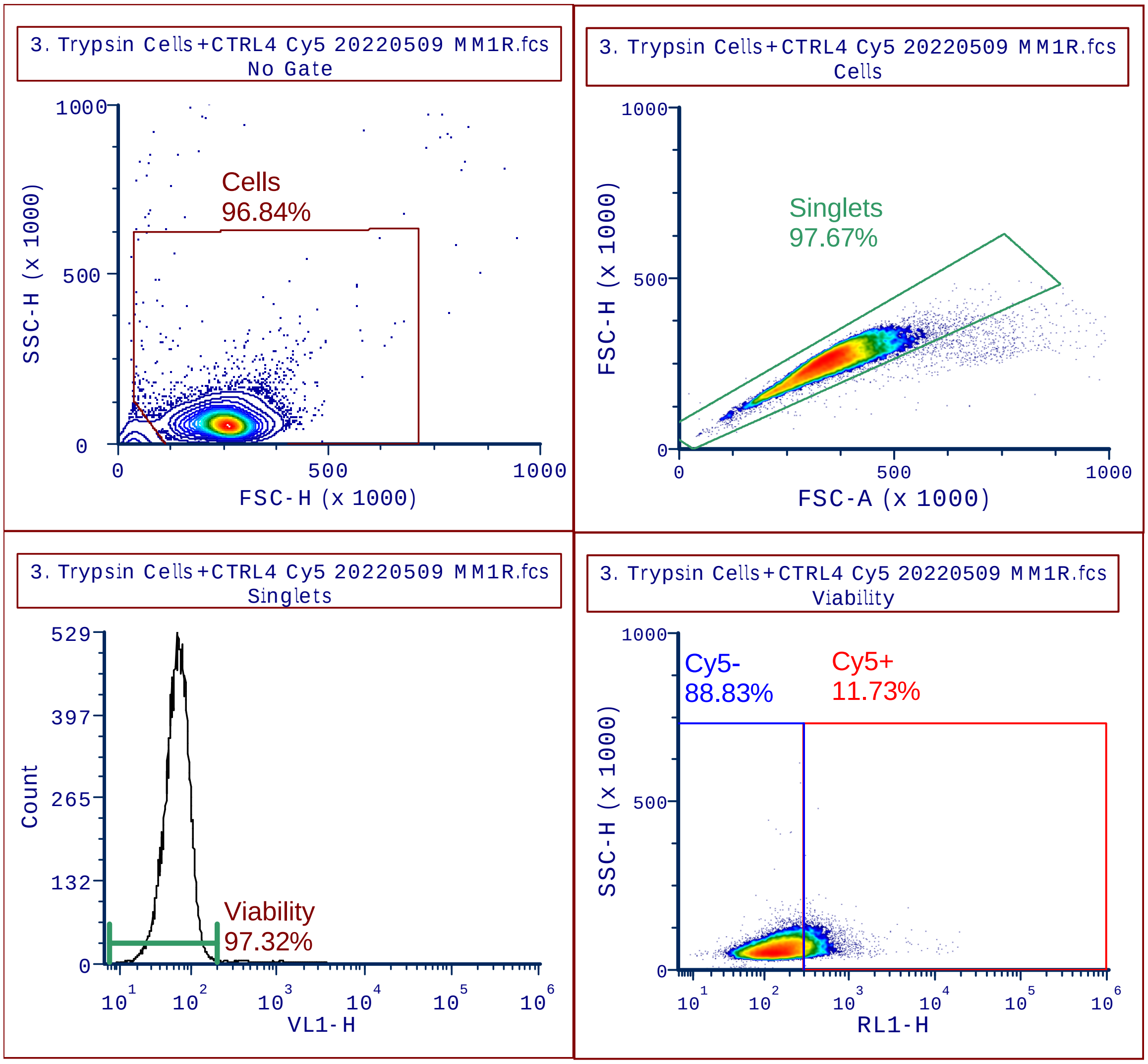

	A	B	C	D	E
1	Filename	Gate	# of Events	% of all cells	% of gated cells
2	3. Trypsin Ce	All Events	37514	100	100
3	3. Trypsin Ce	Cells	36329	96.84	96.84
4	3. Trypsin Ce	Singlets	35482	94.58	97.67
5	3. Trypsin Ce	Viability	34531	92.05	97.32
6	3. Trypsin Ce	Cy5+	4052	10.8	11.73
7	3. Trypsin Ce	Cy5-	30674	81.77	88.83
8					
9					

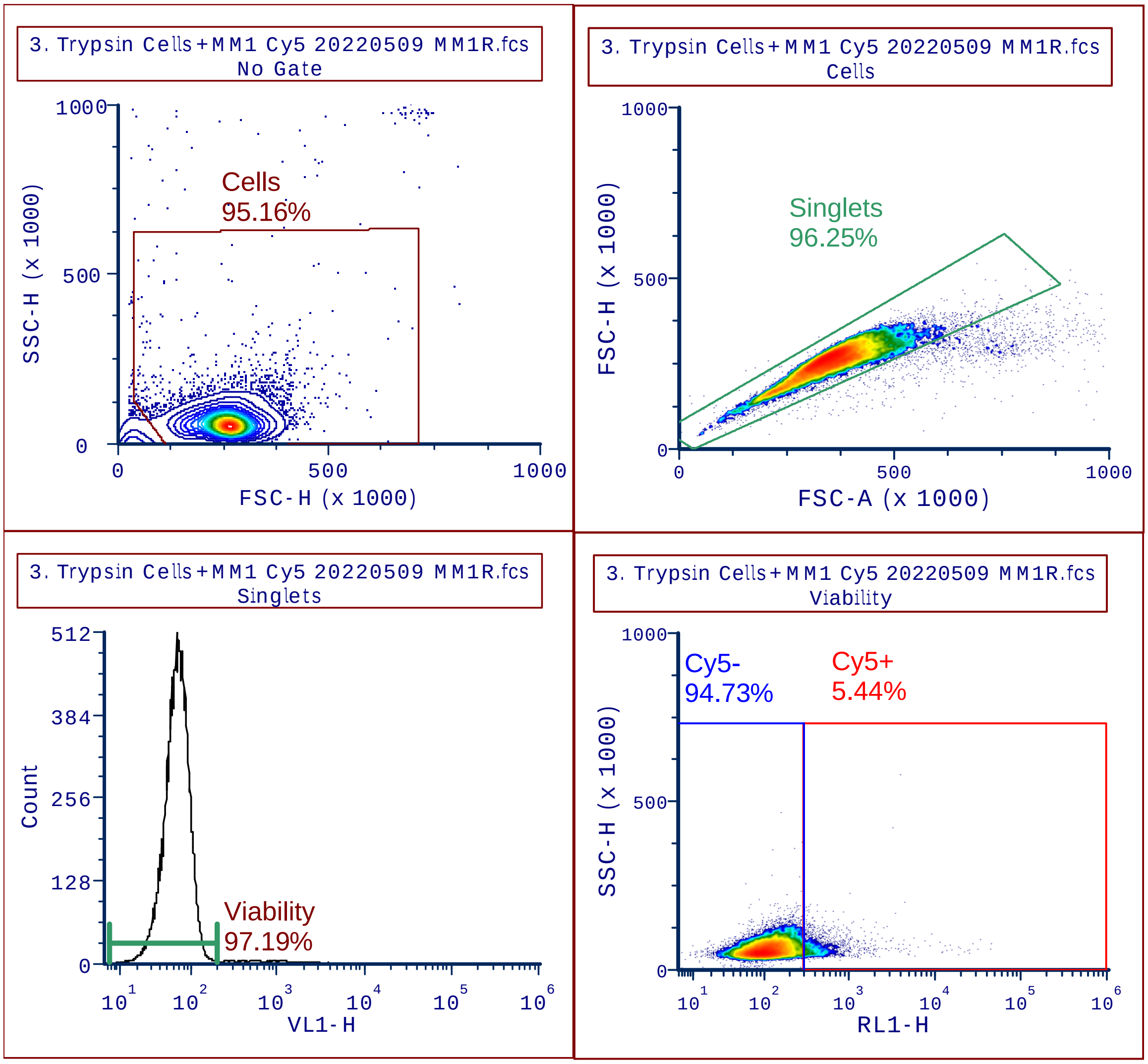

	A	B	C	D	E
1	**Filename**	**Gate**	**# of Events**	**% of all cells**	**% of gated cells**
2	3. Trypsin Ce	All Events	36470	100	100
3	3. Trypsin Ce	Cells	34706	95.16	95.16
4	3. Trypsin Ce	Singlets	33403	91.59	96.25
5	3. Trypsin Ce	Viability	32463	89.01	97.19
6	3. Trypsin Ce	Cy5+	1766	4.84	5.44
7	3. Trypsin Ce	Cy5-	30753	84.32	94.73
8					
9					

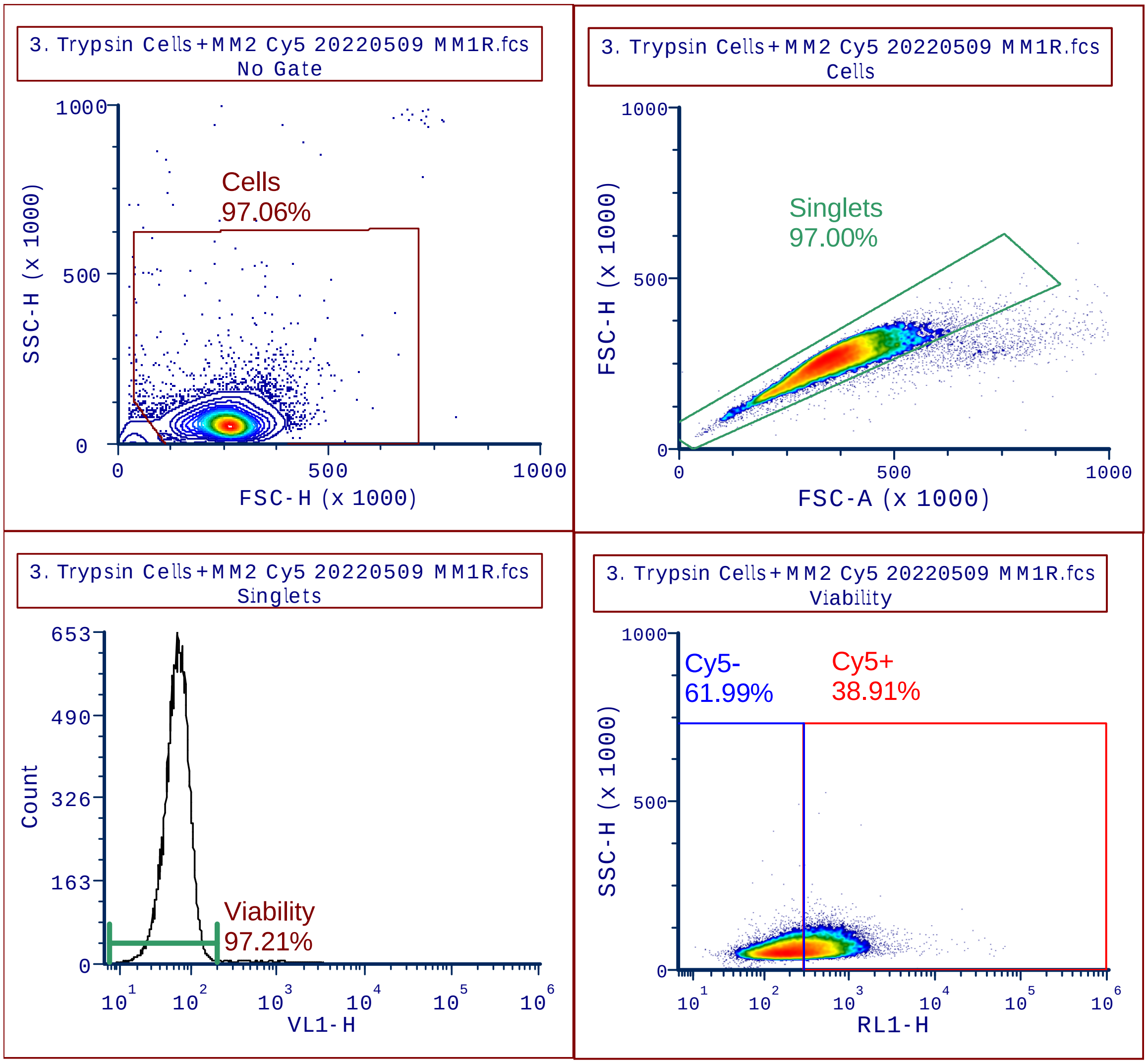

	A	B	C	D	E
1	Filename	**Gate**	**# of Events**	**% of all cells**	**% of gated cells**
2	3. Trypsin Ce	All Events	46150	100	100
3	3. Trypsin Ce	Cells	44792	97.06	97.06
4	3. Trypsin Ce	Singlets	43447	94.14	97
5	3. Trypsin Ce	Viability	42233	91.51	97.21
6	3. Trypsin Ce	Cy5+	16433	35.61	38.91
7	3. Trypsin Ce	Cy5-	26179	56.73	61.99
8					
9					

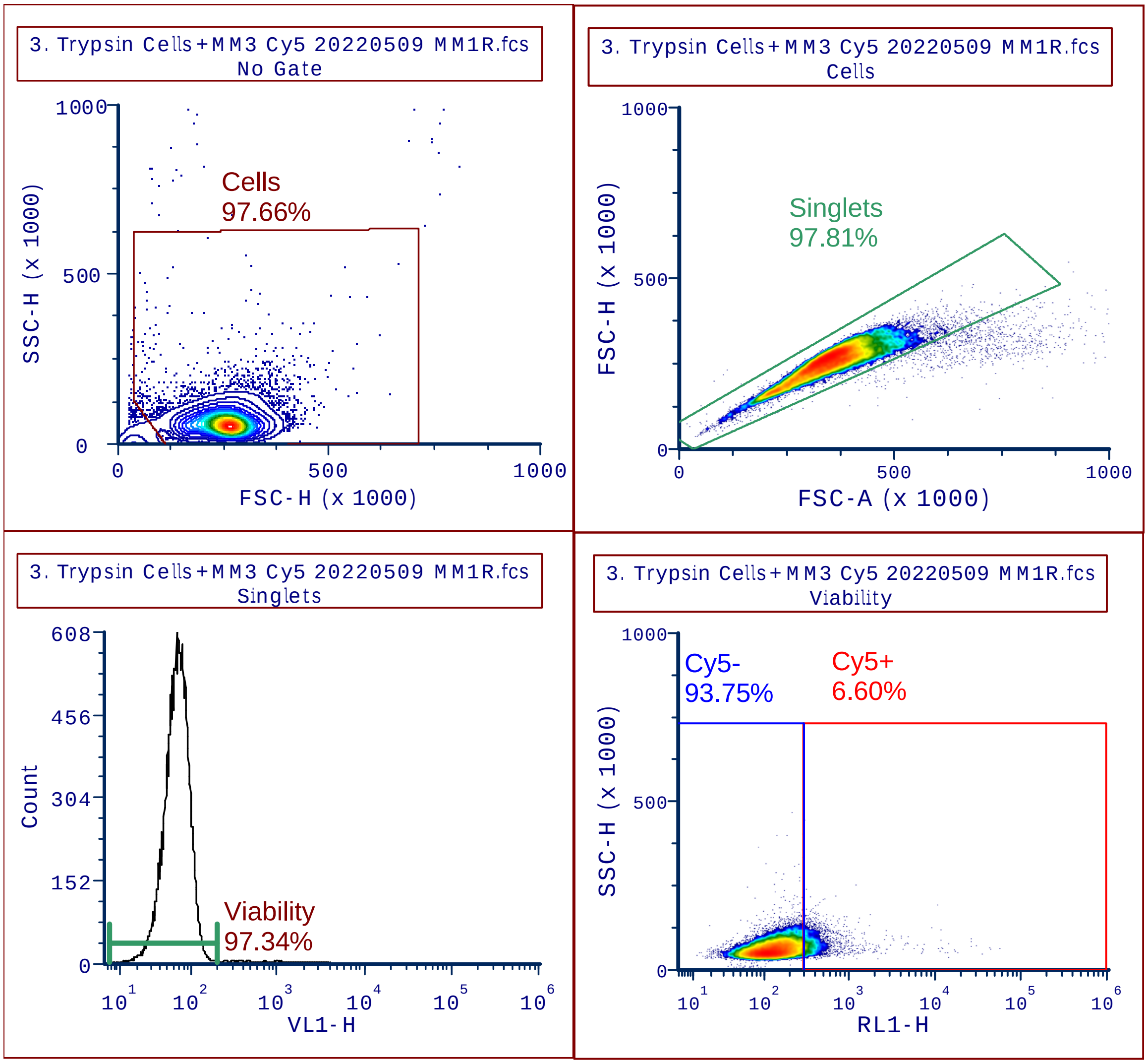

	A	B	C	D	E
1	**Filename**	**Gate**	**# of Events**	**% of all cells**	**% of gated cells**
2	3. Trypsin Ce	All Events	42165	100	100
3	3. Trypsin Ce	Cells	41177	97.66	97.66
4	3. Trypsin Ce	Singlets	40277	95.52	97.81
5	3. Trypsin Ce	Viability	39204	92.98	97.34
6	3. Trypsin Ce	Cy5+	2589	6.14	6.6
7	3. Trypsin Ce	Cy5-	36754	87.17	93.75
8					
9					

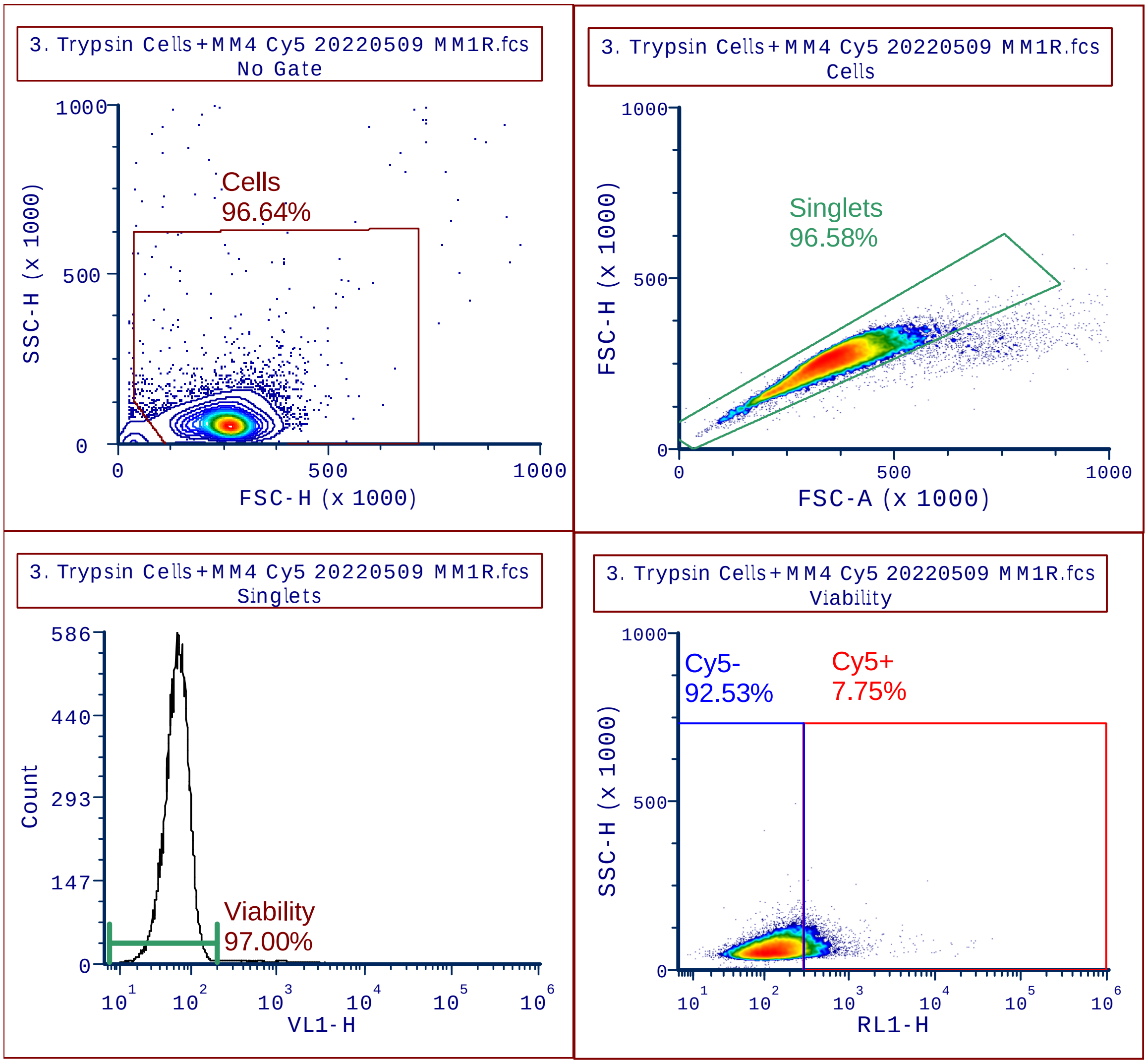

	A	B	C	D	E
1	Filename	**Gate**	**# of Events**	**% of all cells**	**% of gated cells**
2	3. Trypsin Ce	All Events	41398	100	100
3	3. Trypsin Ce	Cells	40008	96.64	96.64
4	3. Trypsin Ce	Singlets	38641	93.34	96.58
5	3. Trypsin Ce	Viability	37483	90.54	97
6	3. Trypsin Ce	Cy5+	2906	7.02	7.75
7	3. Trypsin Ce	Cy5-	34682	83.78	92.53
8					
9					

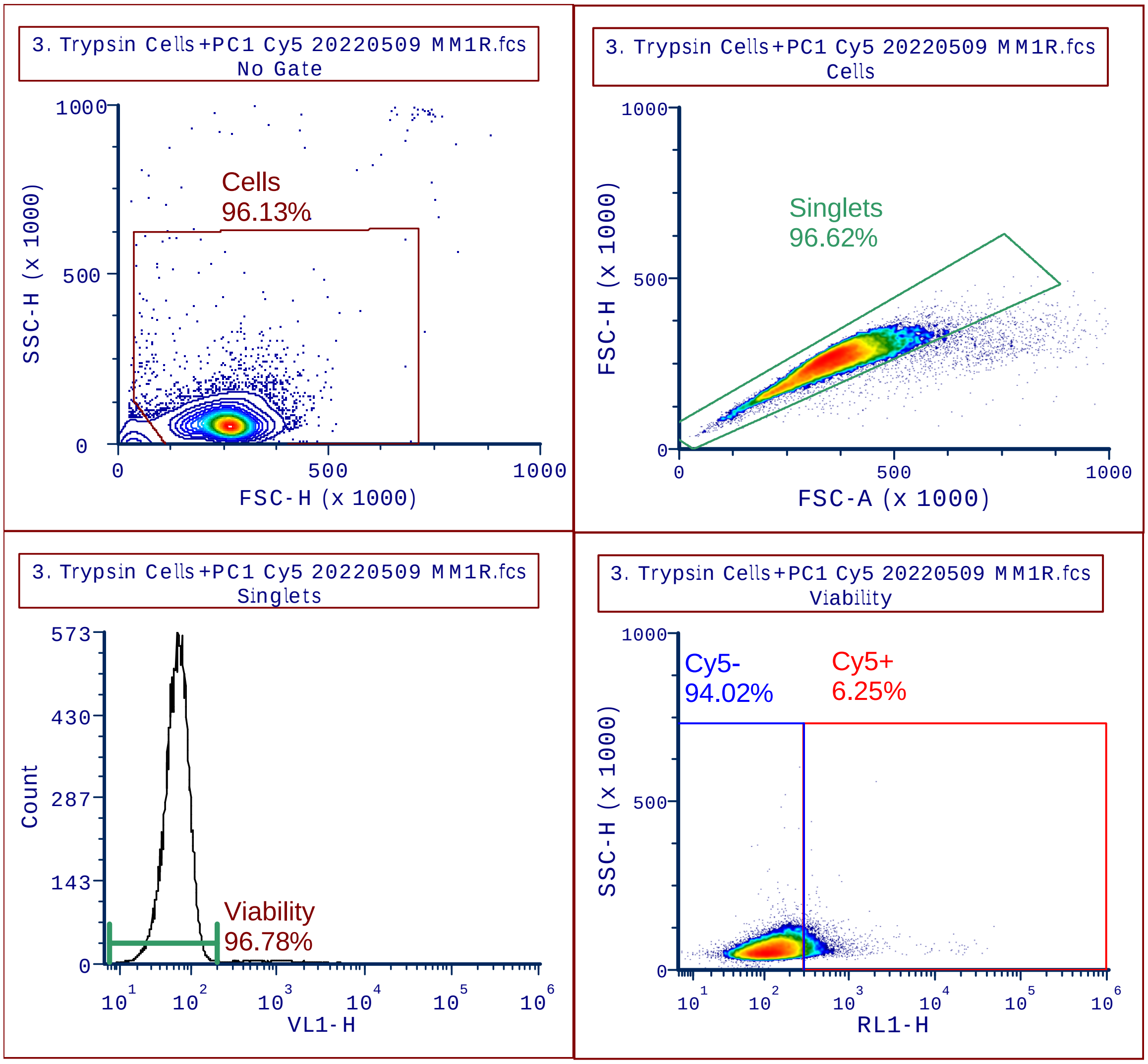

	A	B	C	D	E
1	**Filename**	**Gate**	**# of Events**	**% of all cells**	**% of gated cells**
2	3. Trypsin Ce	All Events	41437	100	100
3	3. Trypsin Ce	Cells	39833	96.13	96.13
4	3. Trypsin Ce	Singlets	38487	92.88	96.62
5	3. Trypsin Ce	Viability	37249	89.89	96.78
6	3. Trypsin Ce	Cy5+	2328	5.62	6.25
7	3. Trypsin Ce	Cy5-	35020	84.51	94.02
8					
9					

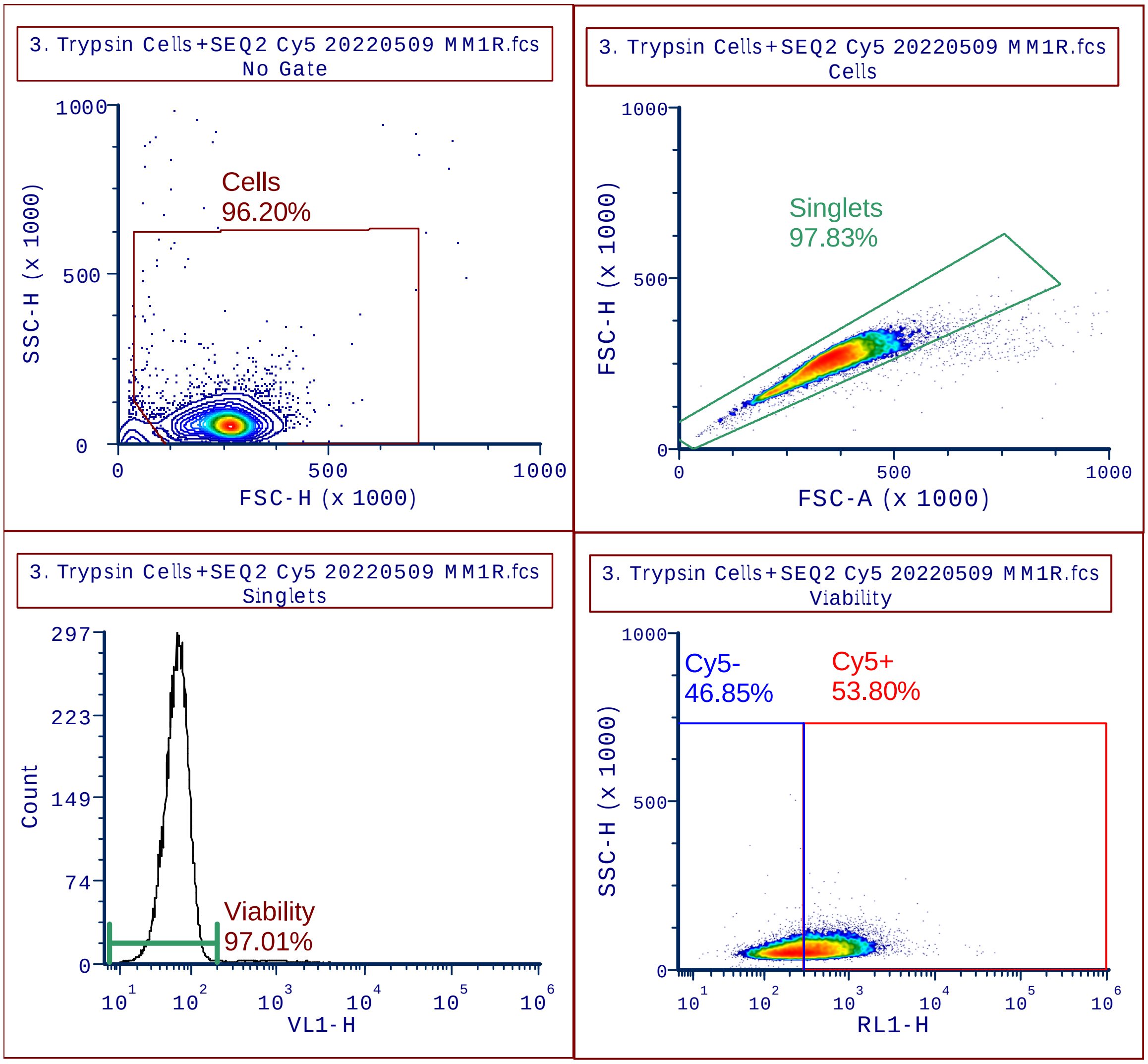

	A	B	C	D	E
1	**Filename**	**Gate**	**# of Events**	**% of all cells**	**% of gated cells**
2	3. Trypsin Ce	All Events	20834	100	100
3	3. Trypsin Ce	Cells	20043	96.2	96.2
4	3. Trypsin Ce	Singlets	19609	94.12	97.83
5	3. Trypsin Ce	Viability	19023	91.31	97.01
6	3. Trypsin Ce	Cy5+	10234	49.12	53.8
7	3. Trypsin Ce	Cy5-	8913	42.78	46.85
8					
9					
