# Supplementary material for: A retrotransposon-derived DNA zip code internalizes myeloma cells through Clathrin-Rab5a-mediated endocytosis
Source: Front Oncol. 2024 Feb 23;14:1288724. doi: 10.3389/fonc.2024.1288724 (PMC10920344; doi:10.3389/fonc.2024.1288724)
Supplement: Supplementary file 12 [file DataSheet_1.zip › Raw data/Fig 2B, SKO-007 with different ZCs/SKO-007 different ZC 1mg per mL.pdf]

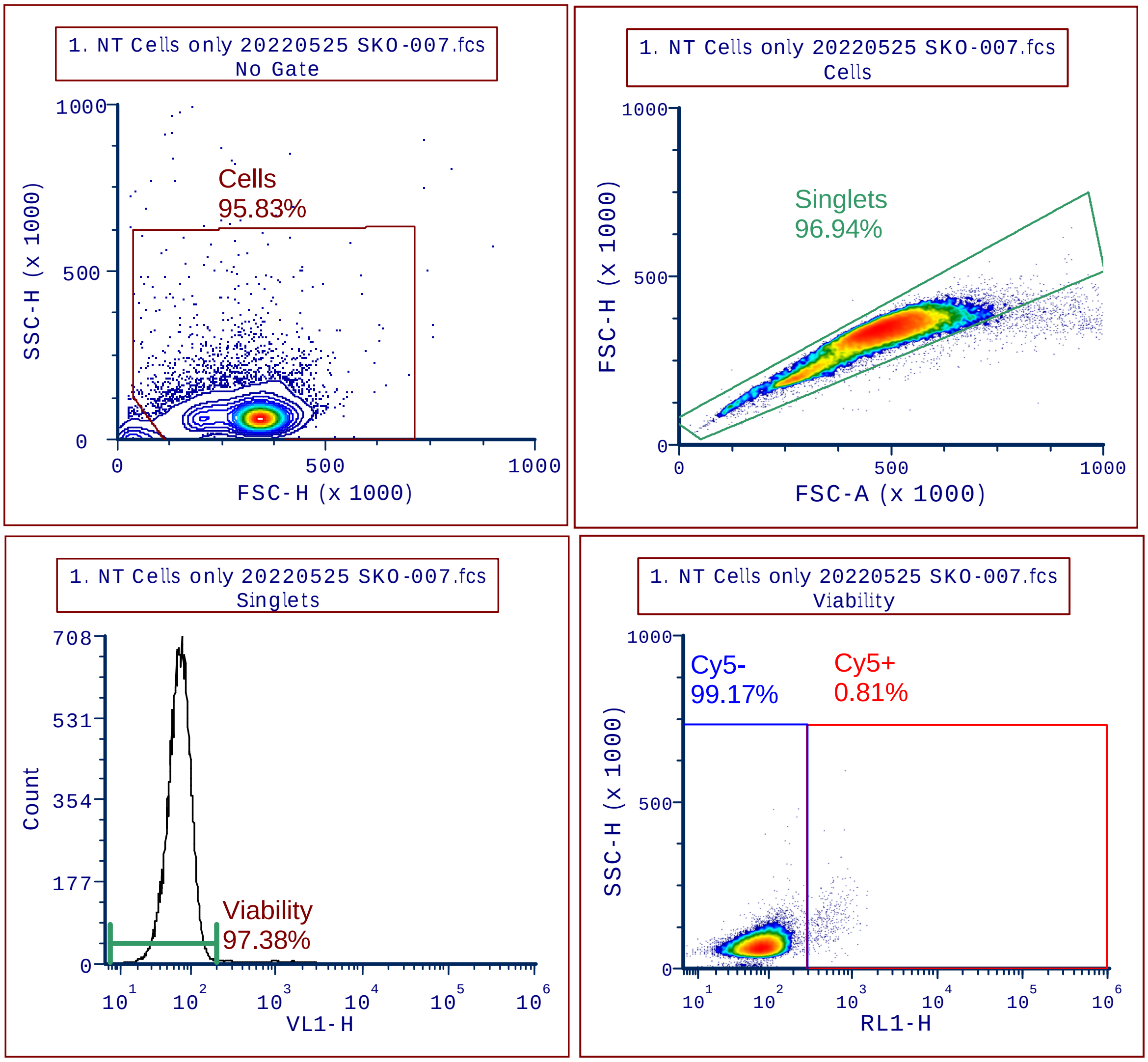

	A	B	C	D	E	F
1	**Filename**	**Gate**	**# of Events**	**% of all cells**	**% of gated cells**	
2	1. NT Cells or	All Events	48791	100	100	
3	1. NT Cells or	Cells	46755	95.83	95.83	
4	1. NT Cells or	Singlets	45323	92.89	96.94	
5	1. NT Cells or	Viability	44134	90.46	97.38	
6	1. NT Cells or	Cy5+	357	0.73	0.81	
7	1. NT Cells or	Cy5-	43769	89.71	99.17	
8						
9						
10						

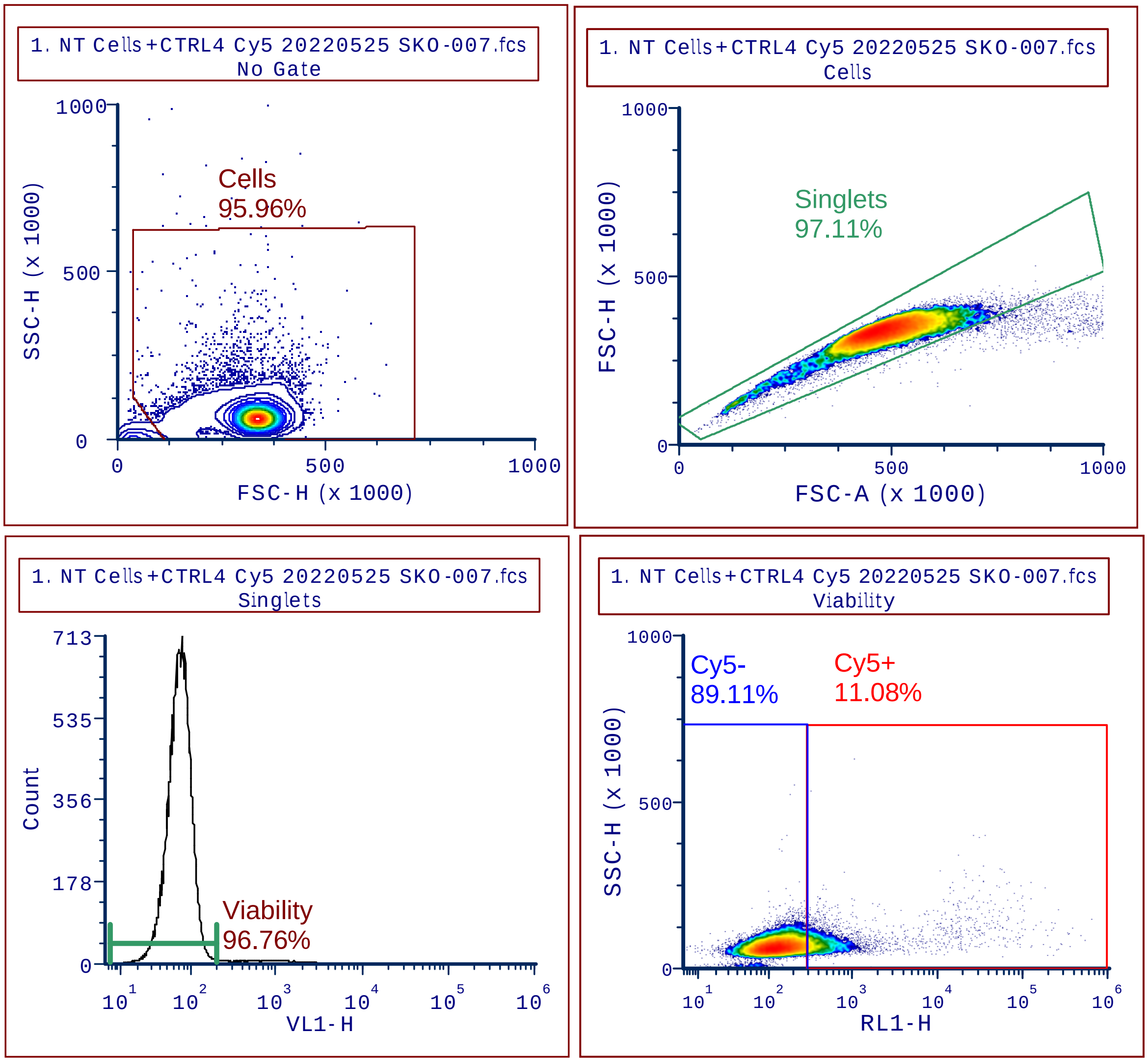

	A	B	C	D	E	F
1	**Filename**	**Gate**	**# of Events**	**% of all cells**	**% of gated cells**	
2	1. NT Cells+C	All Events	48917	100	100	
3	1. NT Cells+C	Cells	46941	95.96	95.96	
4	1. NT Cells+C	Singlets	45583	93.18	97.11	
5	1. NT Cells+C	Viability	44107	90.17	96.76	
6	1. NT Cells+C	Cy5+	4885	9.99	11.08	
7	1. NT Cells+C	Cy5-	39302	80.34	89.11	
8						
9						
10						

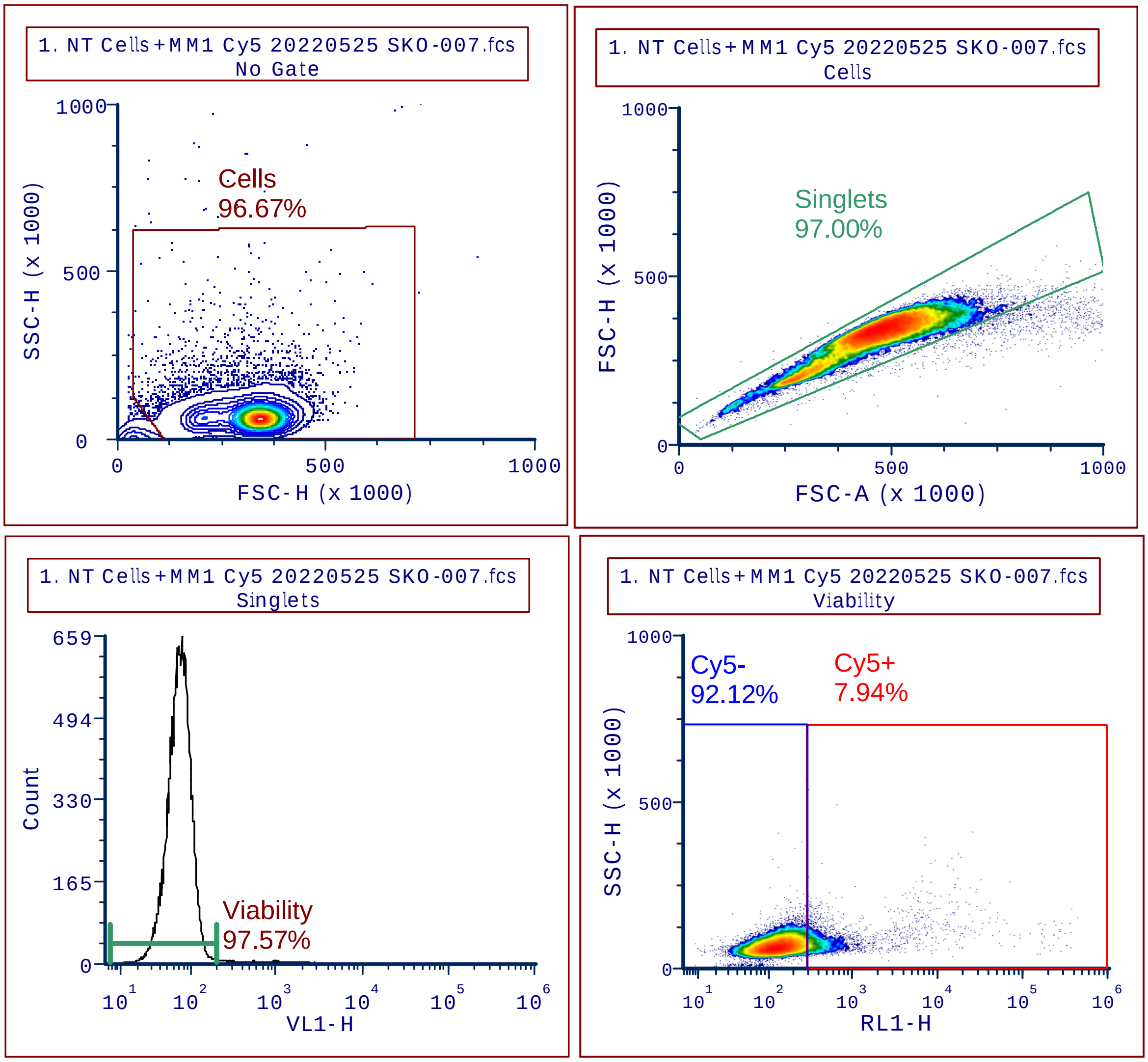

	A	B	C	D	E	F
1	**Filename**	**Gate**	**# of Events**	**% of all cells**	**% of gated cells**	
2	1. NT Cells+M	All Events	44867	100	100	
3	1. NT Cells+M	Cells	43374	96.67	96.67	
4	1. NT Cells+M	Singlets	42073	93.77	97	
5	1. NT Cells+M	Viability	41049	91.49	97.57	
6	1. NT Cells+M	Cy5+	3260	7.27	7.94	
7	1. NT Cells+M	Cy5-	37814	84.28	92.12	
8						
9						
10						

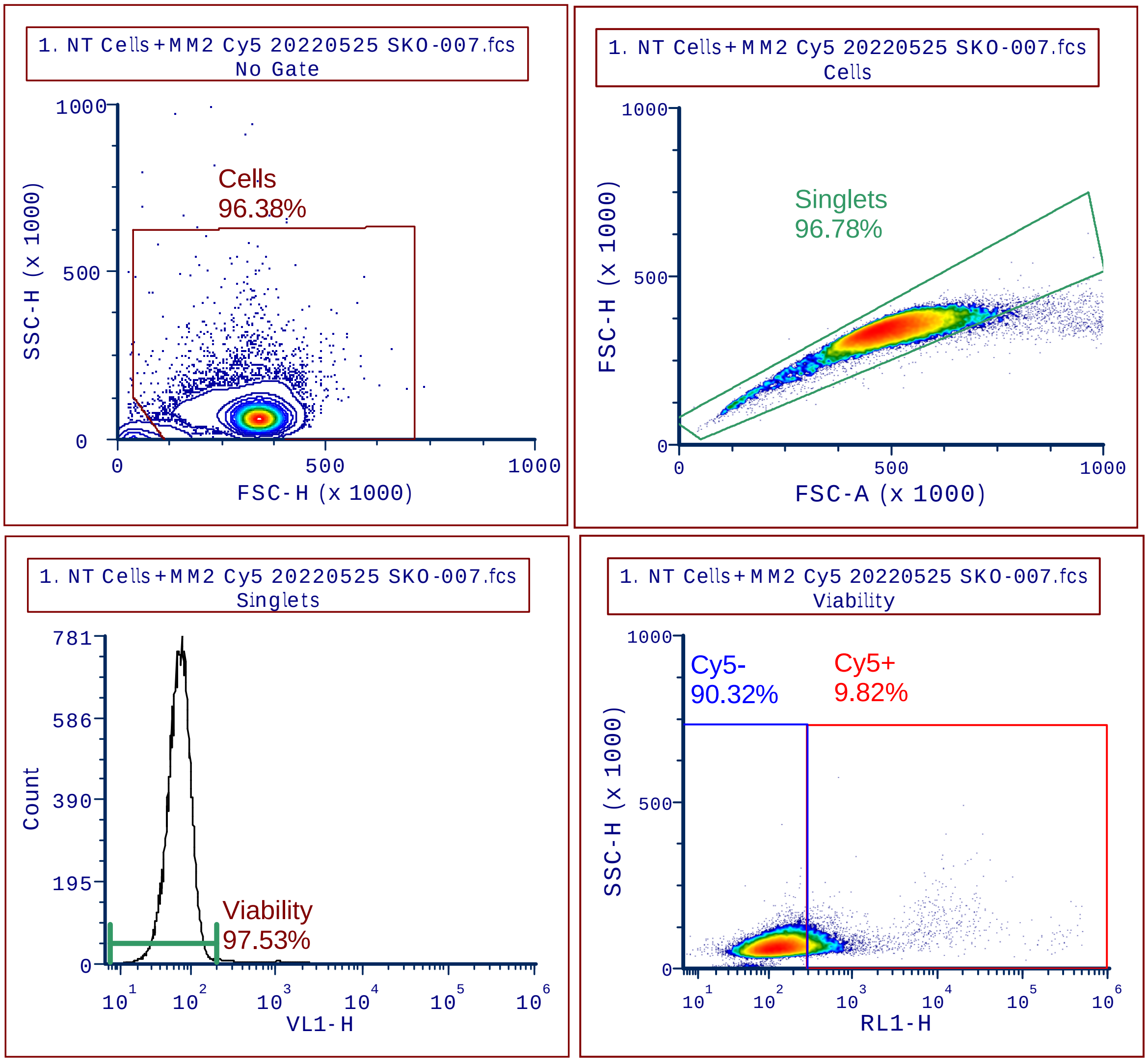

	A	B	C	D	E	F
1	**Filename**	**Gate**	**# of Events**	**% of all cells**	**% of gated cells**	
2	1. NT Cells+M	All Events	53472	100	100	
3	1. NT Cells+M	Cells	51536	96.38	96.38	
4	1. NT Cells+M	Singlets	49878	93.28	96.78	
5	1. NT Cells+M	Viability	48645	90.97	97.53	
6	1. NT Cells+M	Cy5+	4777	8.93	9.82	
7	1. NT Cells+M	Cy5-	43938	82.17	90.32	
8						
9						
10						

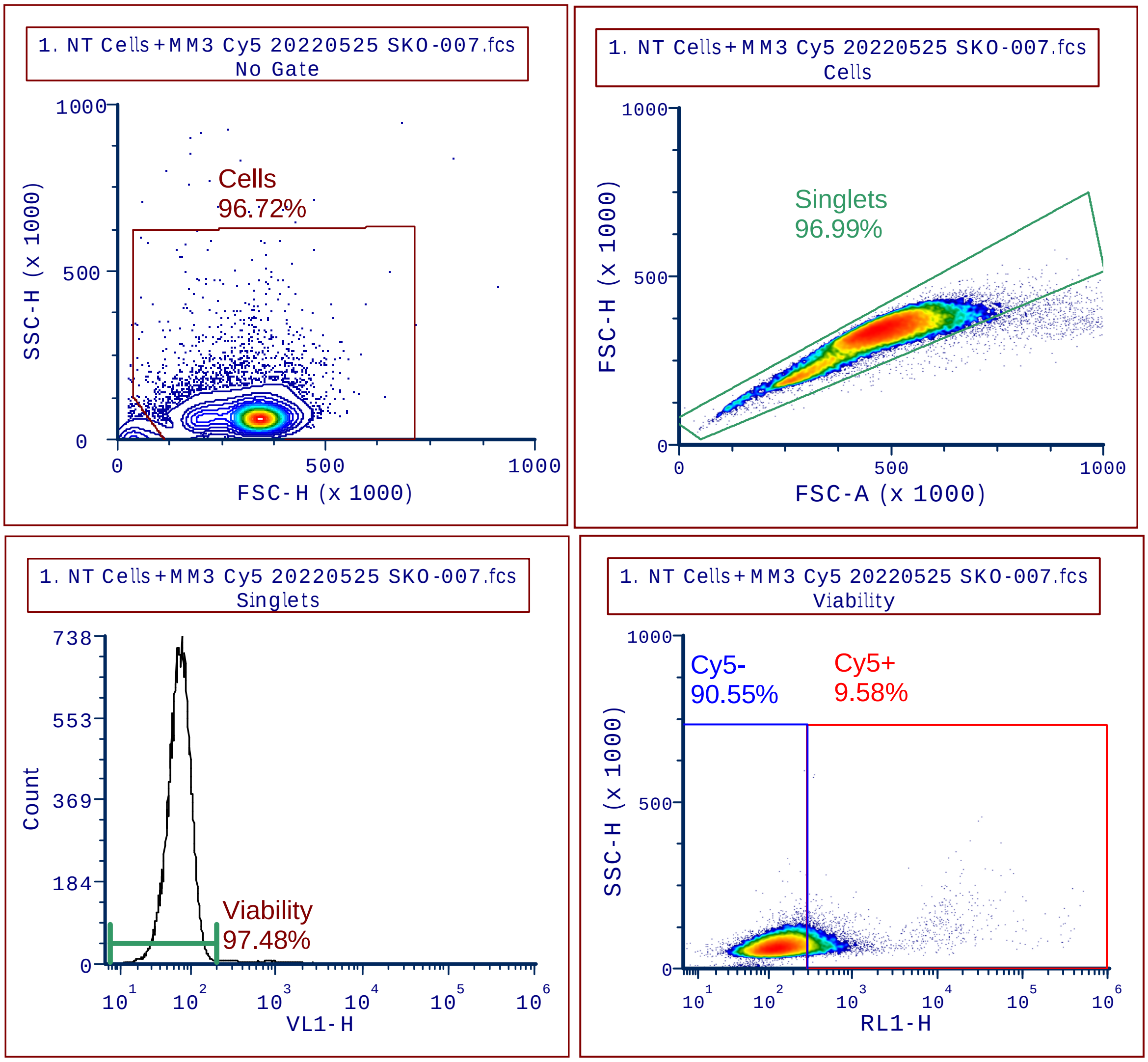

	A	B	C	D	E	F
1	**Filename**	**Gate**	**# of Events**	**% of all cells**	**% of gated cells**	
2	1. NT Cells+M	All Events	50259	100	100	
3	1. NT Cells+M	Cells	48612	96.72	96.72	
4	1. NT Cells+M	Singlets	47147	93.81	96.99	
5	1. NT Cells+M	Viability	45959	91.44	97.48	
6	1. NT Cells+M	Cy5+	4404	8.76	9.58	
7	1. NT Cells+M	Cy5-	41615	82.8	90.55	
8						
9						
10						

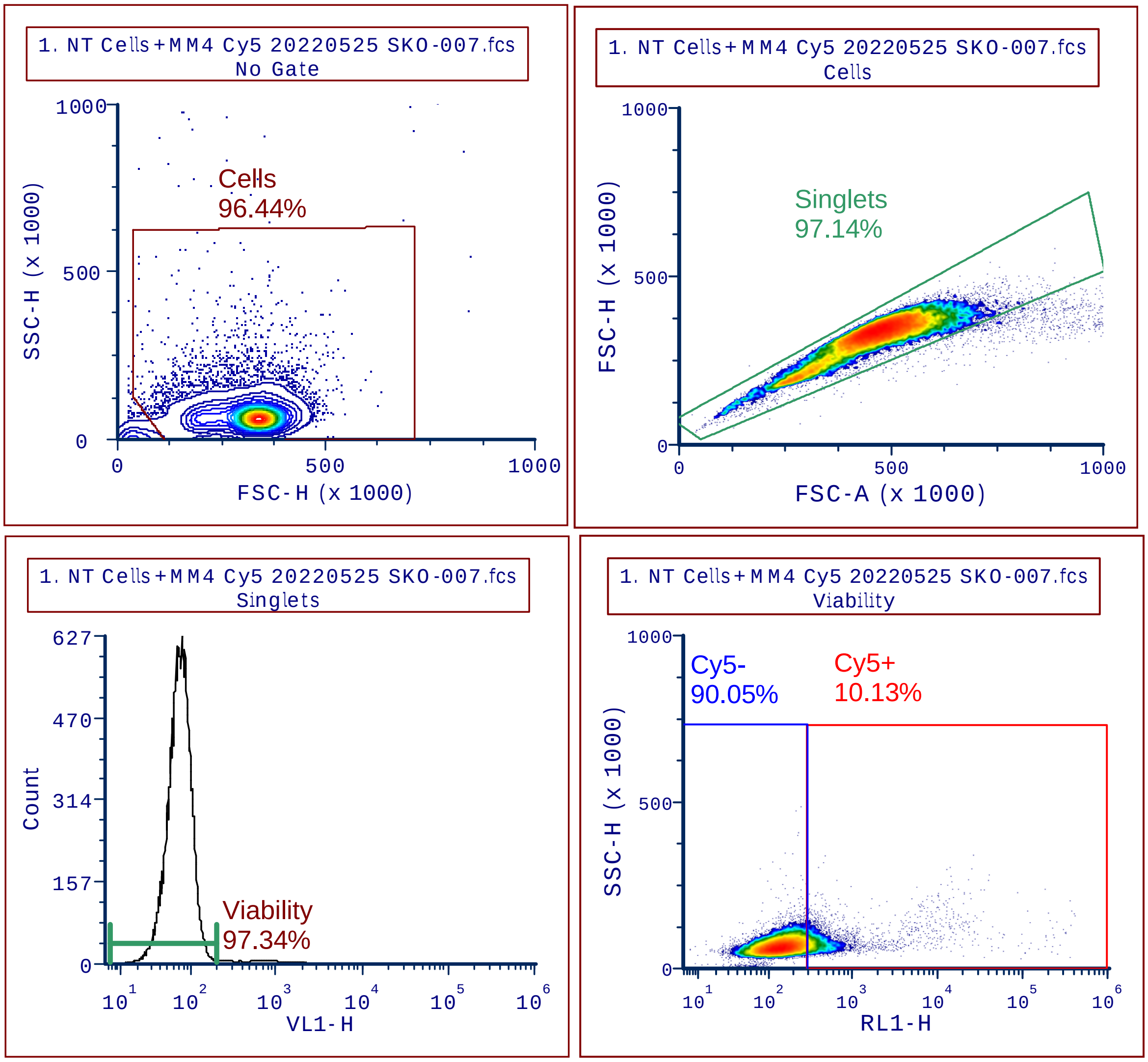

	A	B	C	D	E	F
1	**Filename**	**Gate**	**# of Events**	**% of all cells**	**% of gated cells**	
2	1. NT Cells+M	All Events	42943	100	100	
3	1. NT Cells+M	Cells	41413	96.44	96.44	
4	1. NT Cells+M	Singlets	40230	93.68	97.14	
5	1. NT Cells+M	Viability	39159	91.19	97.34	
6	1. NT Cells+M	Cy5+	3967	9.24	10.13	
7	1. NT Cells+M	Cy5-	35264	82.12	90.05	
8						
9						
10						

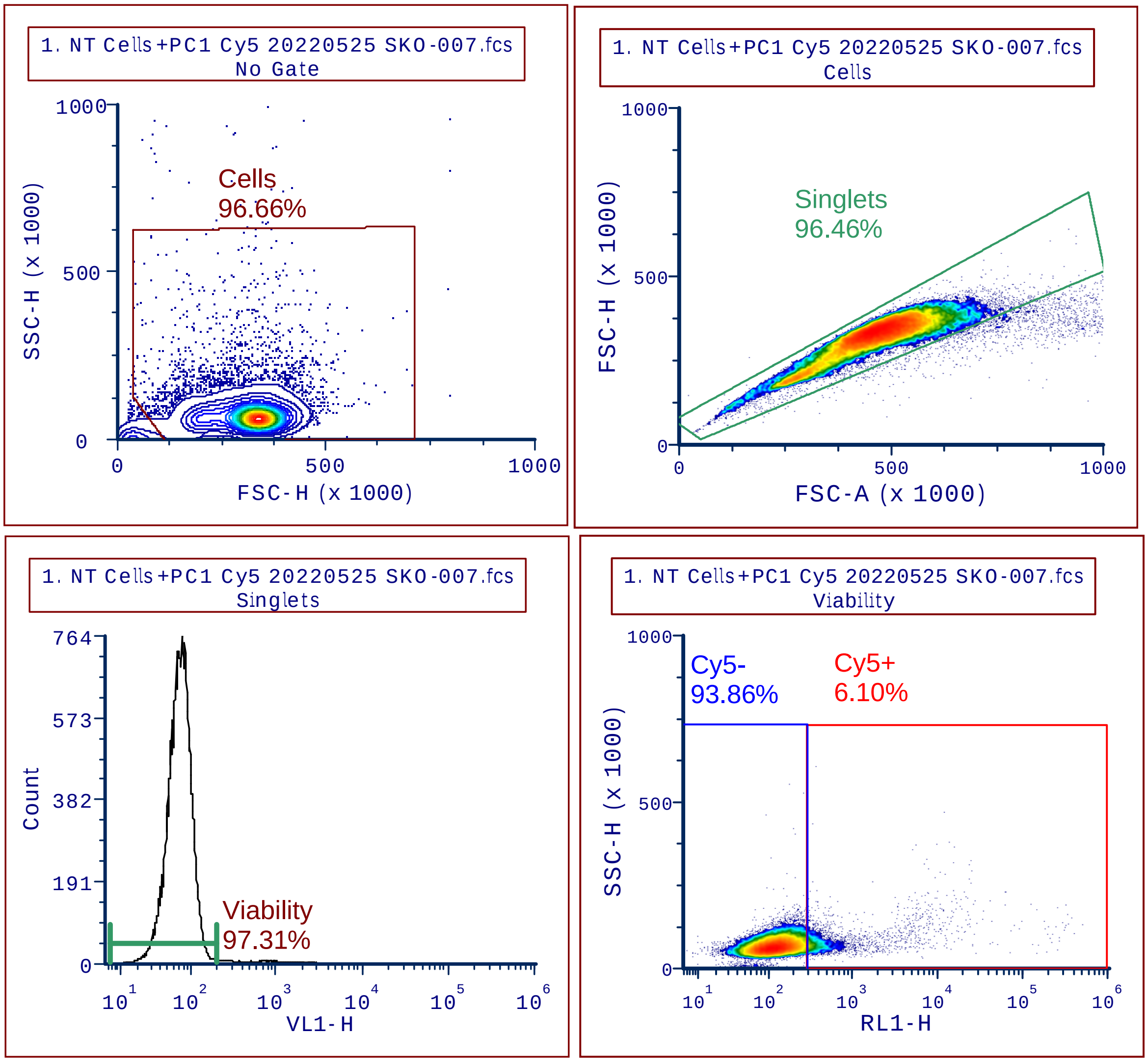

	A	B	C	D	E	F
1	**Filename**	**Gate**	**# of Events**	**% of all cells**	**% of gated cells**	
2	1. NT Cells+P	All Events	52548	100	100	
3	1. NT Cells+P	Cells	50792	96.66	96.66	
4	1. NT Cells+P	Singlets	48994	93.24	96.46	
5	1. NT Cells+P	Viability	47675	90.73	97.31	
6	1. NT Cells+P	Cy5+	2906	5.53	6.1	
7	1. NT Cells+P	Cy5-	44746	85.15	93.86	
8						
9						
10						

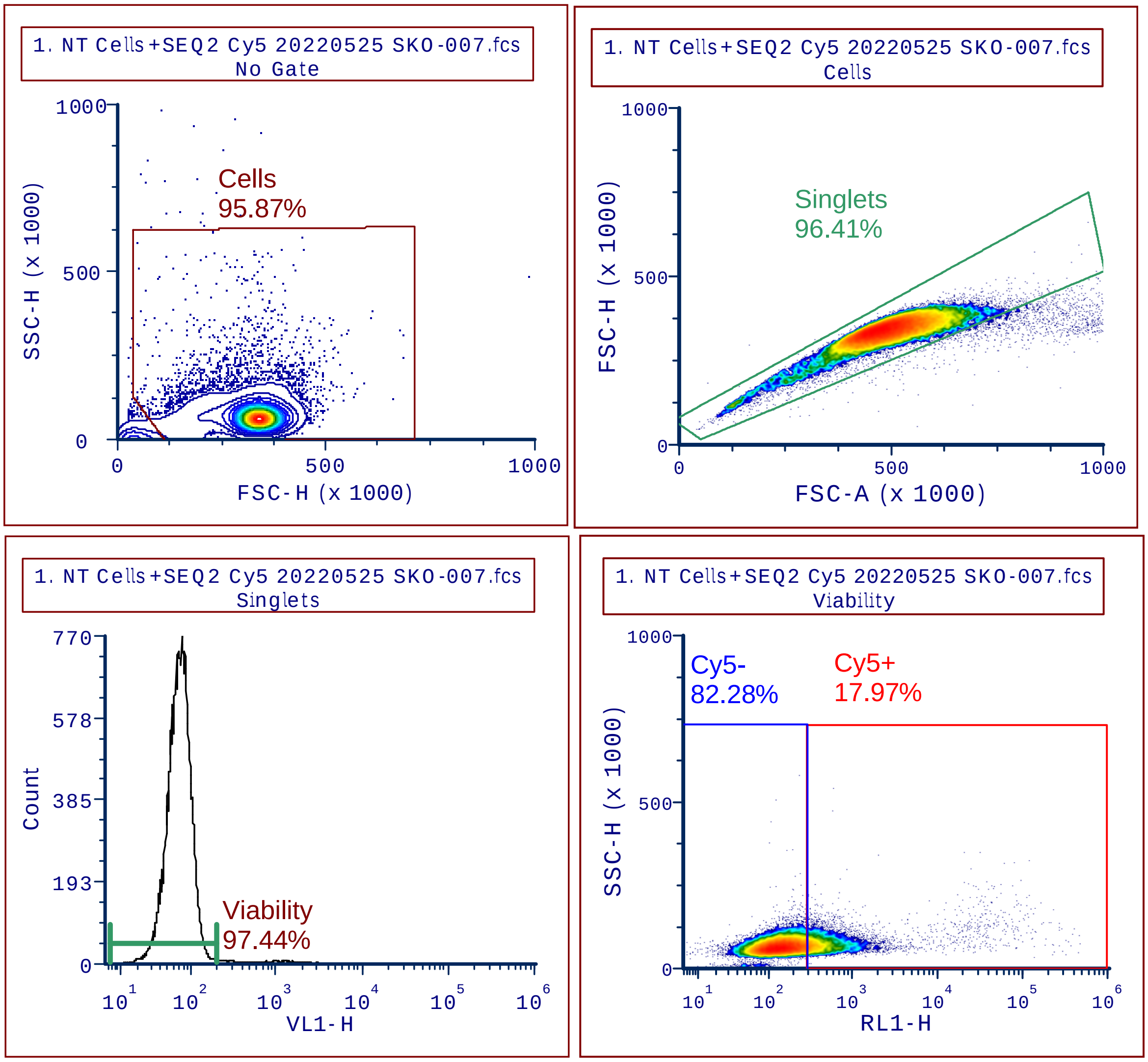

	A	B	C	D	E	F
1	**Filename**	**Gate**	**# of Events**	**% of all cells**	**% of gated cells**	
2	1. NT Cells+SI	All Events	53272	100	100	
3	1. NT Cells+SI	Cells	51073	95.87	95.87	
4	1. NT Cells+SI	Singlets	49241	92.43	96.41	
5	1. NT Cells+SI	Viability	47981	90.07	97.44	
6	1. NT Cells+SI	Cy5+	8623	16.19	17.97	
7	1. NT Cells+SI	Cy5-	39477	74.1	82.28	
8						
9						
10						

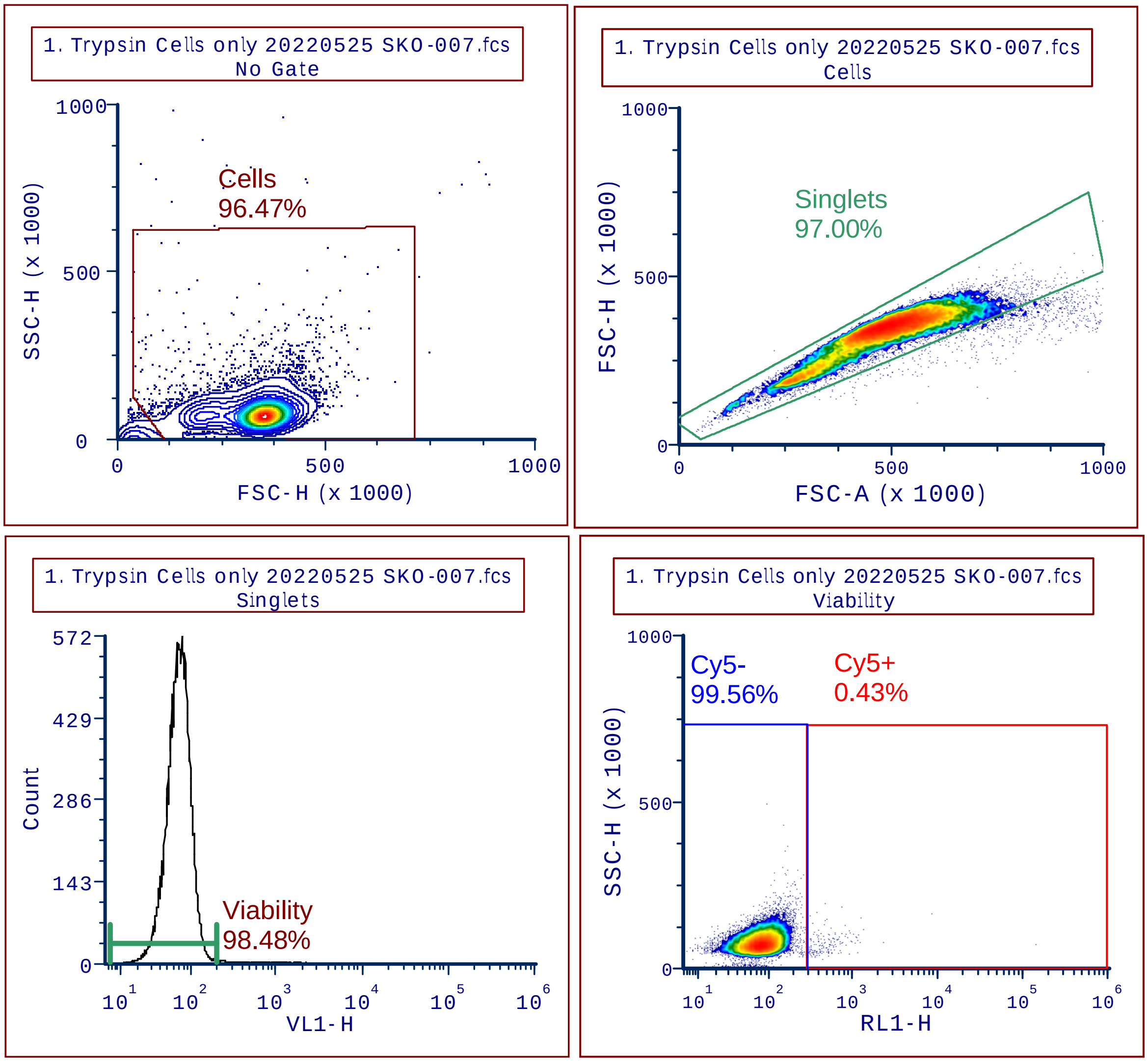

	A	B	C	D	E	F
1	**Filename**	**Gate**	**# of Events**	**% of all cells**	**% of gated cells**	
2	1. Trypsin Cel	All Events	39344	100	100	
3	1. Trypsin Cel	Cells	37957	96.47	96.47	
4	1. Trypsin Cel	Singlets	36818	93.58	97	
5	1. Trypsin Cel	Viability	36260	92.16	98.48	
6	1. Trypsin Cel	Cy5+	155	0.39	0.43	
7	1. Trypsin Cel	Cy5-	36100	91.75	99.56	
8						
9						
10						

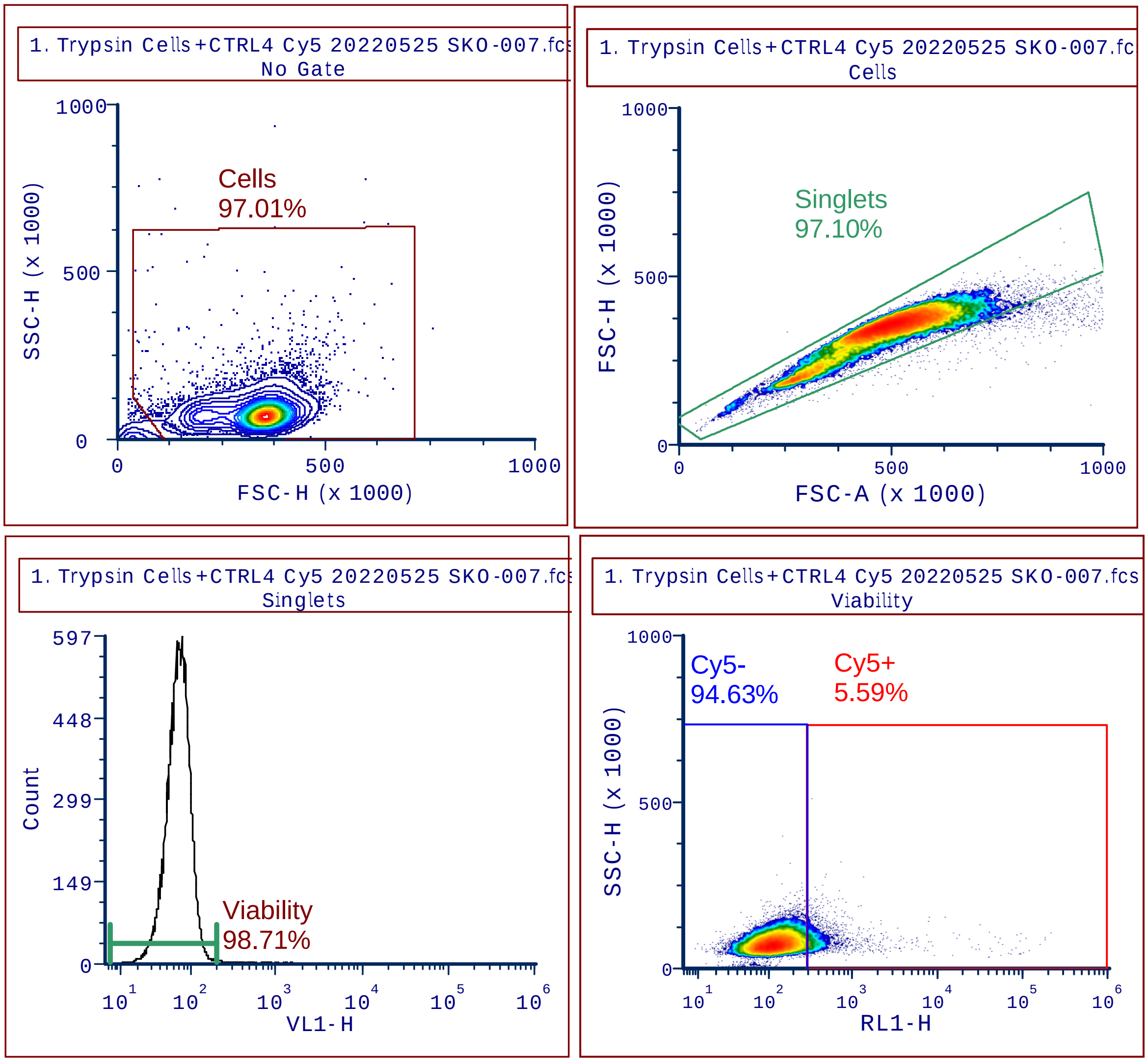

	A	B	C	D	E	F
1	**Filename**	**Gate**	**# of Events**	**% of all cells**	**% of gated cells**	
2	1. Trypsin Cel	All Events	40201	100	100	
3	1. Trypsin Cel	Cells	39001	97.01	97.01	
4	1. Trypsin Cel	Singlets	37870	94.2	97.1	
5	1. Trypsin Cel	Viability	37380	92.98	98.71	
6	1. Trypsin Cel	Cy5+	2091	5.2	5.59	
7	1. Trypsin Cel	Cy5-	35371	87.99	94.63	
8						
9						
10						

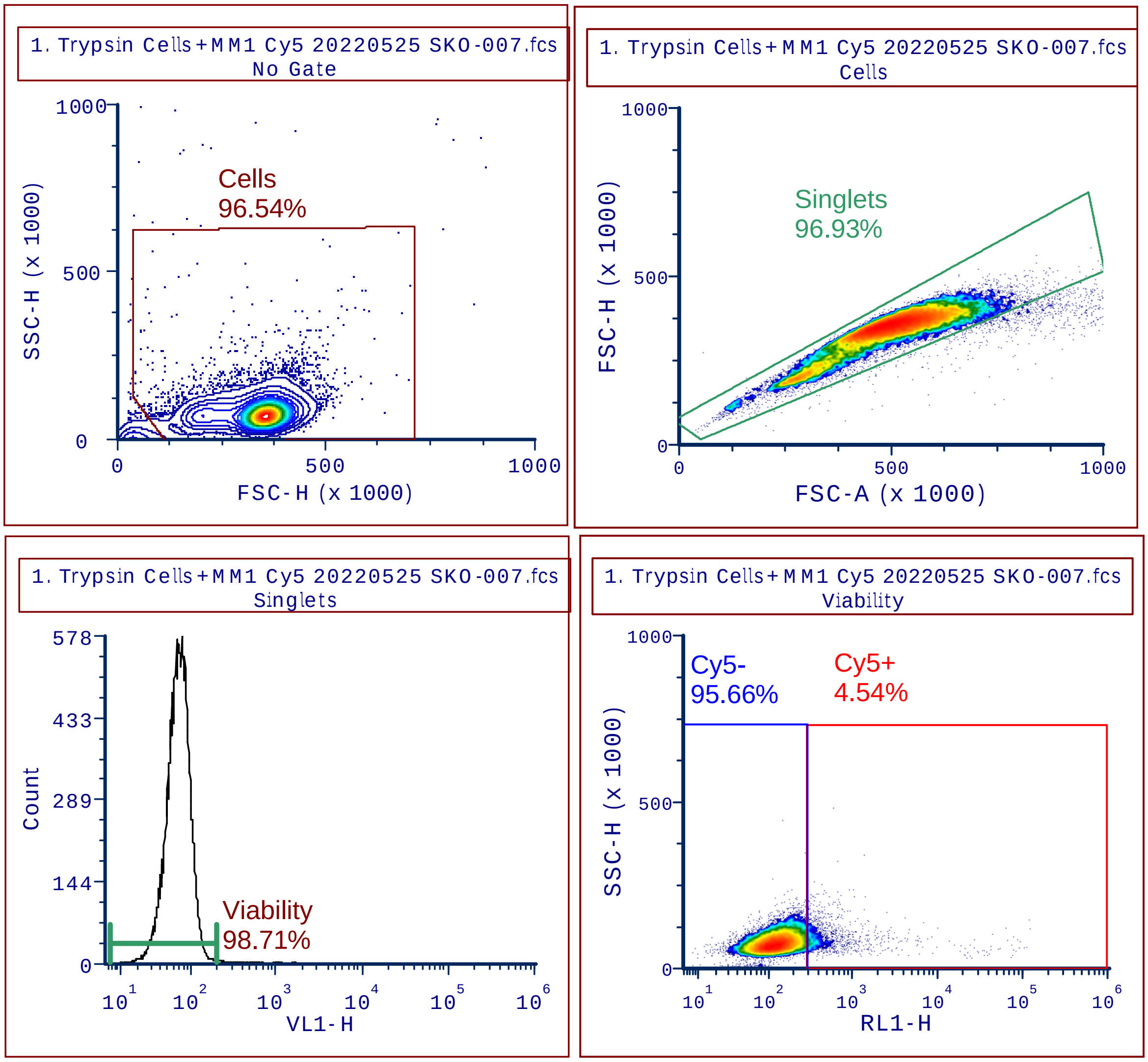

	A	B	C	D	E	F
1	**Filename**	**Gate**	**# of Events**	**% of all cells**	**% of gated cells**	
2	1. Trypsin Cel	All Events	39057	100	100	
3	1. Trypsin Cel	Cells	37704	96.54	96.54	
4	1. Trypsin Cel	Singlets	36548	93.58	96.93	
5	1. Trypsin Cel	Viability	36078	92.37	98.71	
6	1. Trypsin Cel	Cy5+	1639	4.2	4.54	
7	1. Trypsin Cel	Cy5-	34514	88.37	95.66	
8						
9						
10						

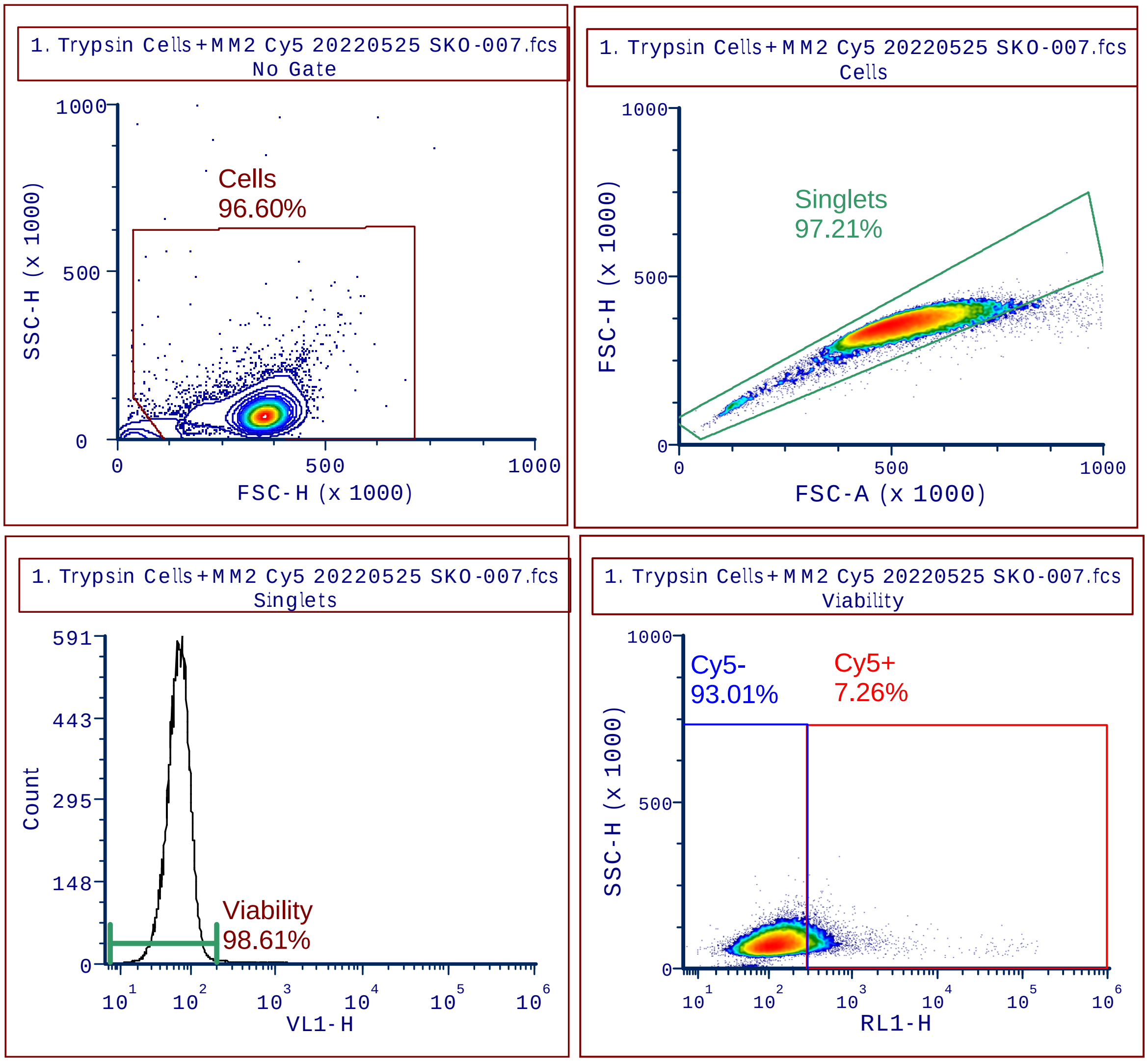

	A	B	C	D	E	F
1	**Filename**	**Gate**	**# of Events**	**% of all cells**	**% of gated cells**	
2	1. Trypsin Cel	All Events	39902	100	100	
3	1. Trypsin Cel	Cells	38545	96.6	96.6	
4	1. Trypsin Cel	Singlets	37471	93.91	97.21	
5	1. Trypsin Cel	Viability	36951	92.6	98.61	
6	1. Trypsin Cel	Cy5+	2683	6.72	7.26	
7	1. Trypsin Cel	Cy5-	34367	86.13	93.01	
8						
9						
10						

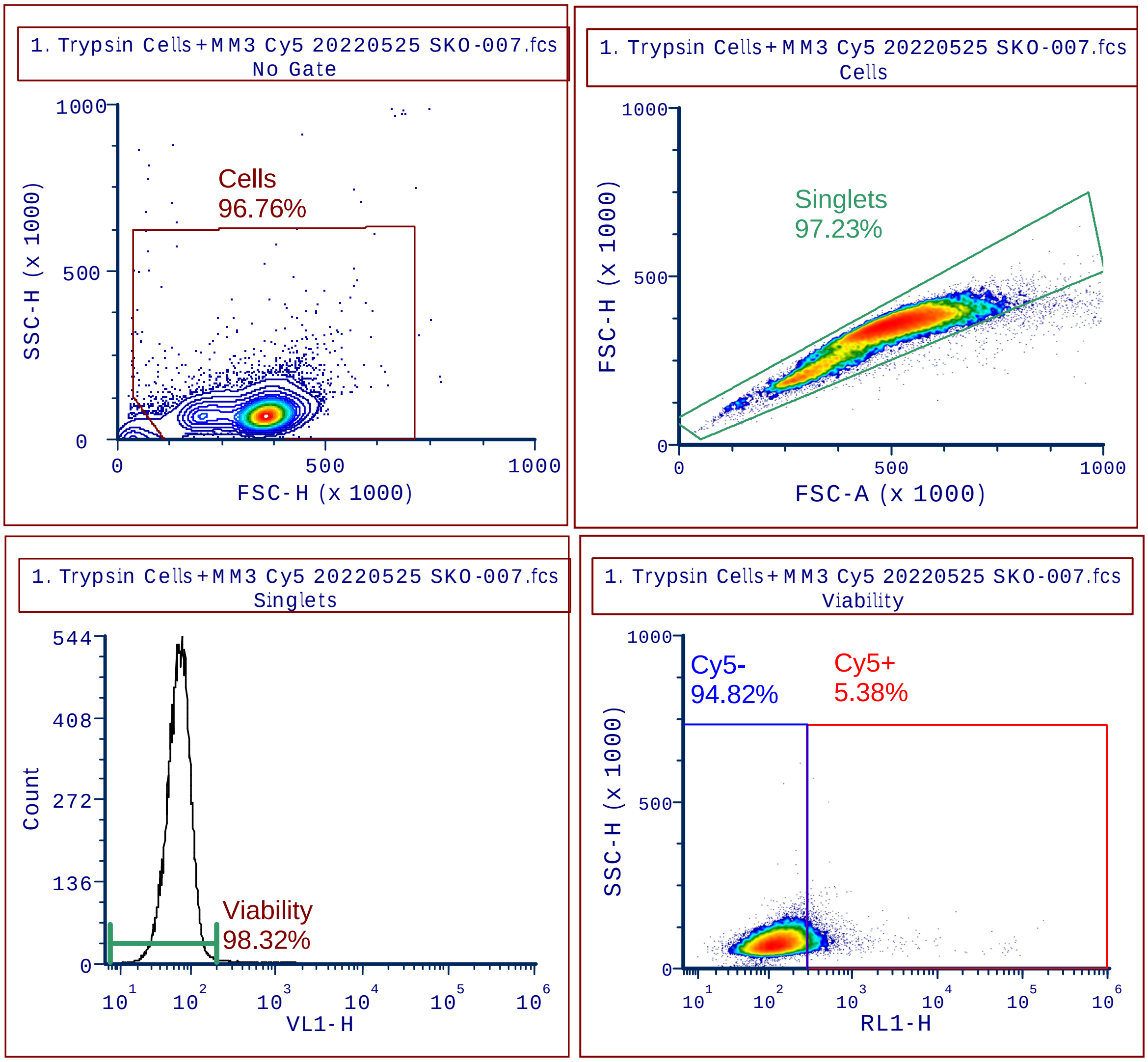

	A	B	C	D	E	F
1	**Filename**	**Gate**	**# of Events**	**% of all cells**	**% of gated cells**	
2	1. Trypsin Cel	All Events	37545	100	100	
3	1. Trypsin Cel	Cells	36329	96.76	96.76	
4	1. Trypsin Cel	Singlets	35323	94.08	97.23	
5	1. Trypsin Cel	Viability	34730	92.5	98.32	
6	1. Trypsin Cel	Cy5+	1869	4.98	5.38	
7	1. Trypsin Cel	Cy5-	32932	87.71	94.82	
8						
9						
10						

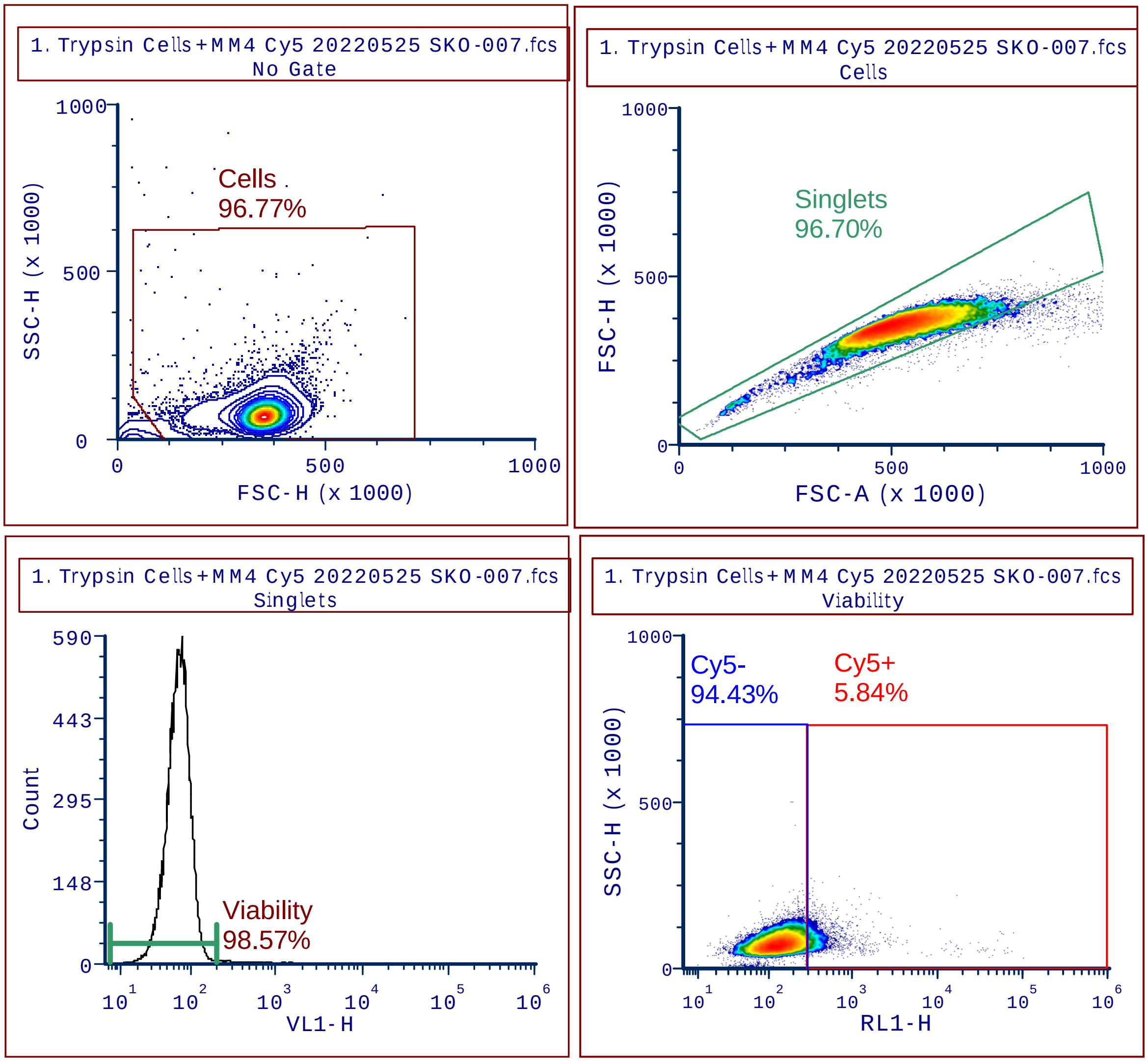

	A	B	C	D	E	F
1	**Filename**	**Gate**	**# of Events**	**% of all cells**	**% of gated cells**	
2	1. Trypsin Cel	All Events	39127	100	100	
3	1. Trypsin Cel	Cells	37865	96.77	96.77	
4	1. Trypsin Cel	Singlets	36616	93.58	96.7	
5	1. Trypsin Cel	Viability	36091	92.24	98.57	
6	1. Trypsin Cel	Cy5+	2107	5.39	5.84	
7	1. Trypsin Cel	Cy5-	34080	87.1	94.43	
8						
9						
10						

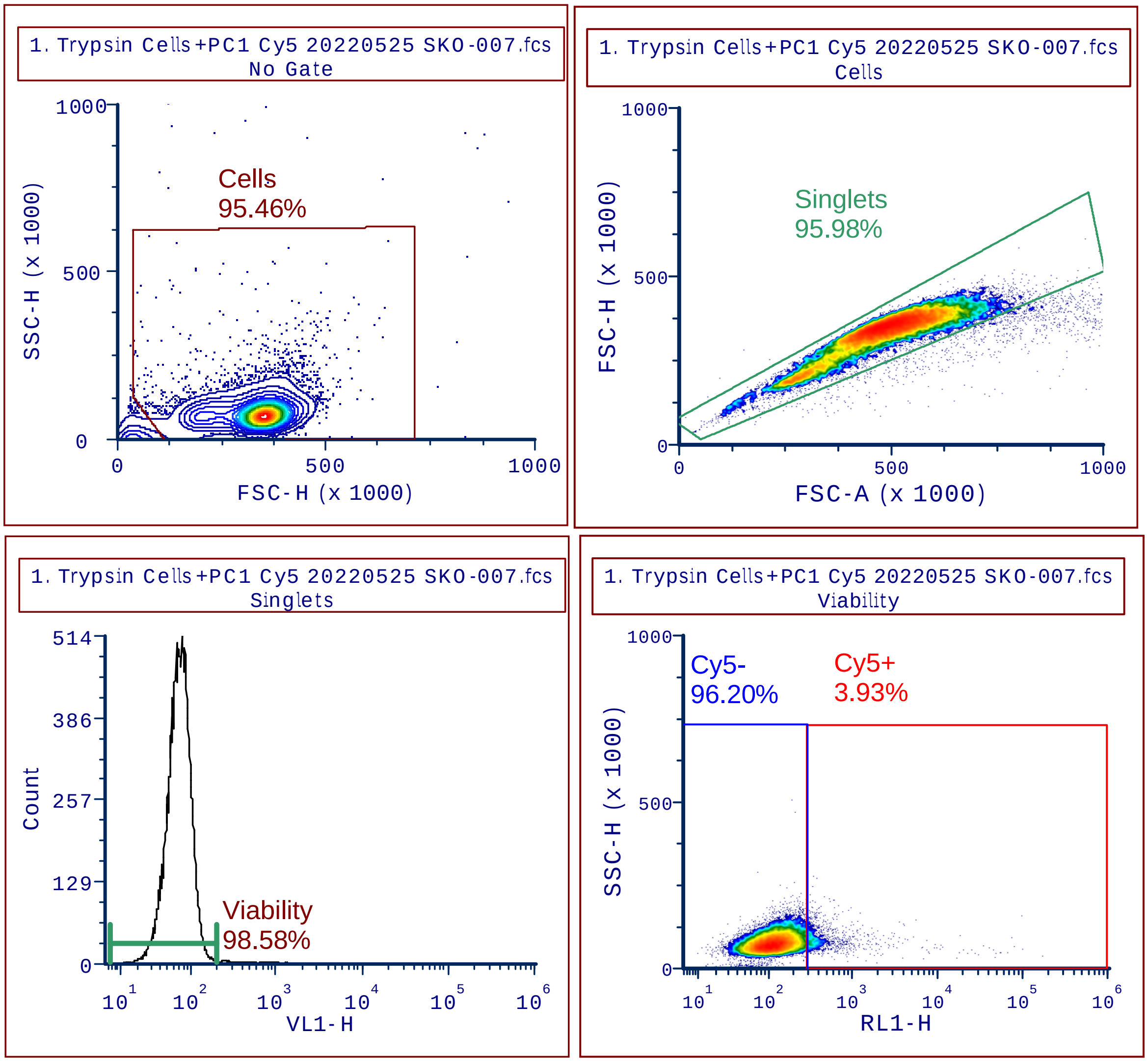

	A	B	C	D	E	F
1	**Filename**	**Gate**	**# of Events**	**% of all cells**	**% of gated cells**	
2	1. Trypsin Cel	All Events	35939	100	100	
3	1. Trypsin Cel	Cells	34309	95.46	95.46	
4	1. Trypsin Cel	Singlets	32931	91.63	95.98	
5	1. Trypsin Cel	Viability	32462	90.33	98.58	
6	1. Trypsin Cel	Cy5+	1276	3.55	3.93	
7	1. Trypsin Cel	Cy5-	31228	86.89	96.2	
8						
9						
10						

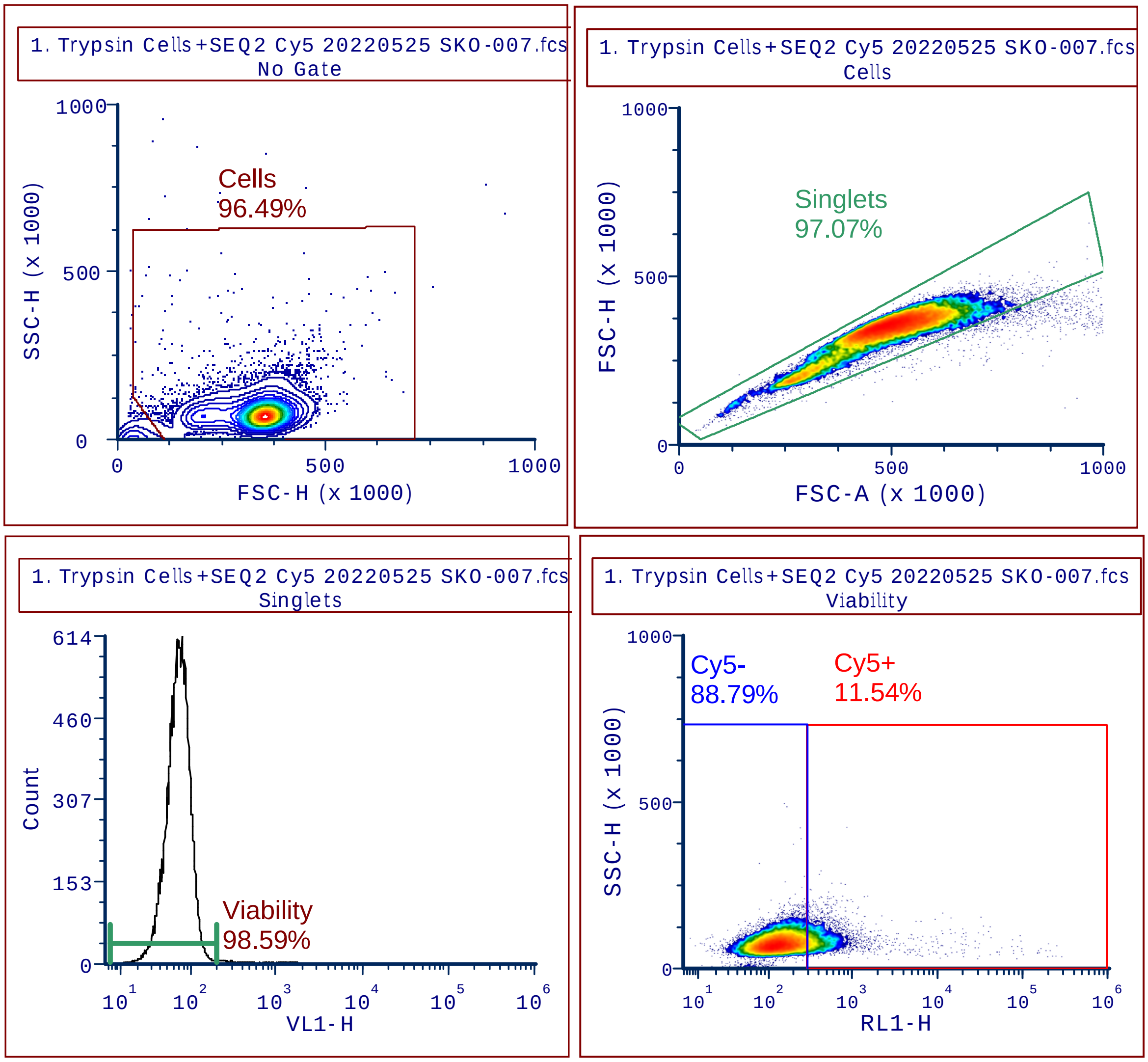

	A	B	C	D	E	F
1	**Filename**	**Gate**	**# of Events**	**% of all cells**	**% of gated cells**	
2	1. Trypsin Cel	All Events	41557	100	100	
3	1. Trypsin Cel	Cells	40097	96.49	96.49	
4	1. Trypsin Cel	Singlets	38922	93.66	97.07	
5	1. Trypsin Cel	Viability	38372	92.34	98.59	
6	1. Trypsin Cel	Cy5+	4427	10.65	11.54	
7	1. Trypsin Cel	Cy5-	34069	81.98	88.79	
8						
9						
10						

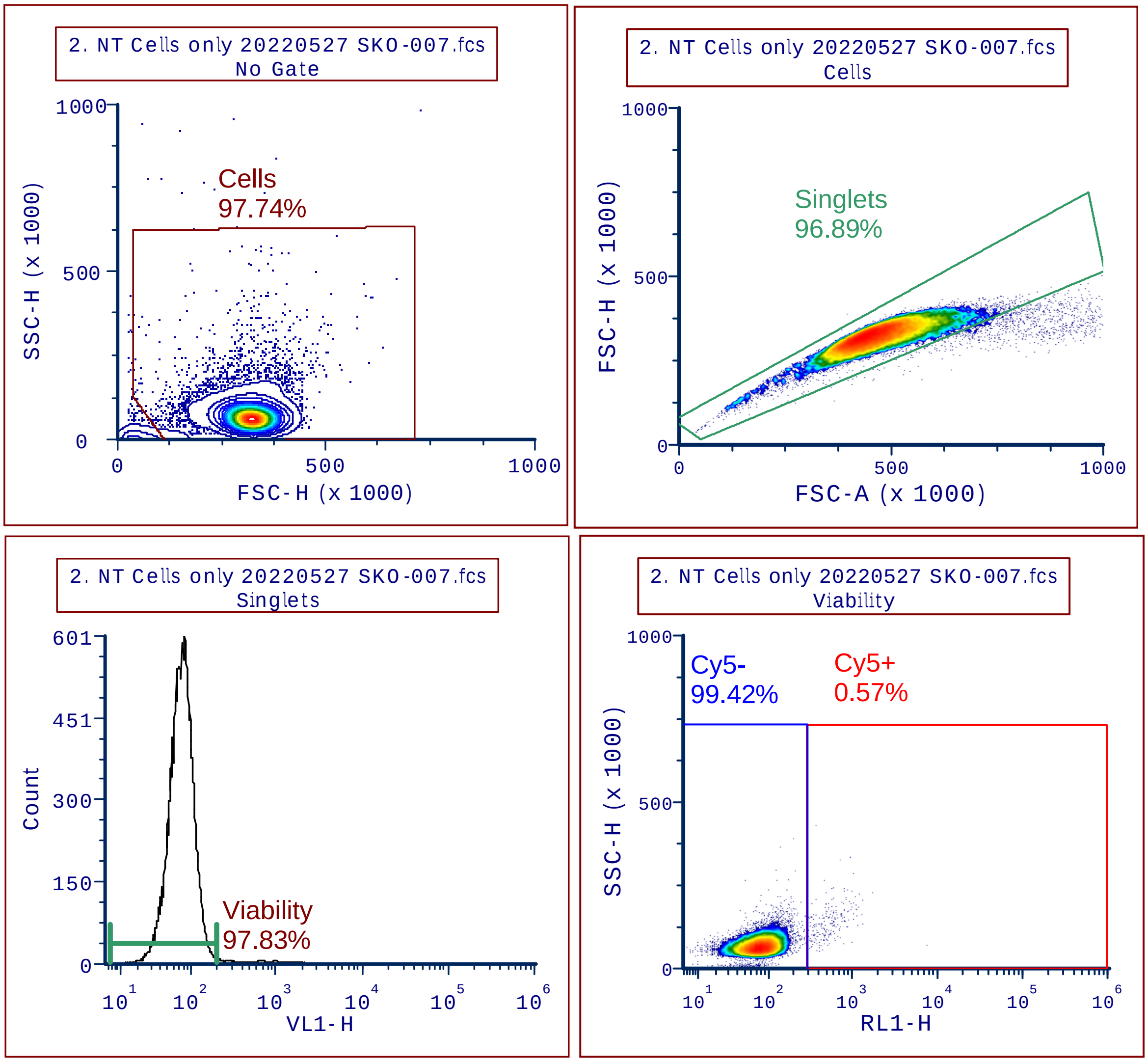

Filename	Gate	# of Events	% of all cells	% of gated cells
2. NT Cells or	All Events	41568	100	100
2. NT Cells or	Cells	40630	97.74	97.74
2. NT Cells or	Singlets	39368	94.71	96.89
2. NT Cells or	Viability	38512	92.65	97.83
2. NT Cells or	Cy5+	220	0.53	0.57
2. NT Cells or	Cy5-	38288	92.11	99.42

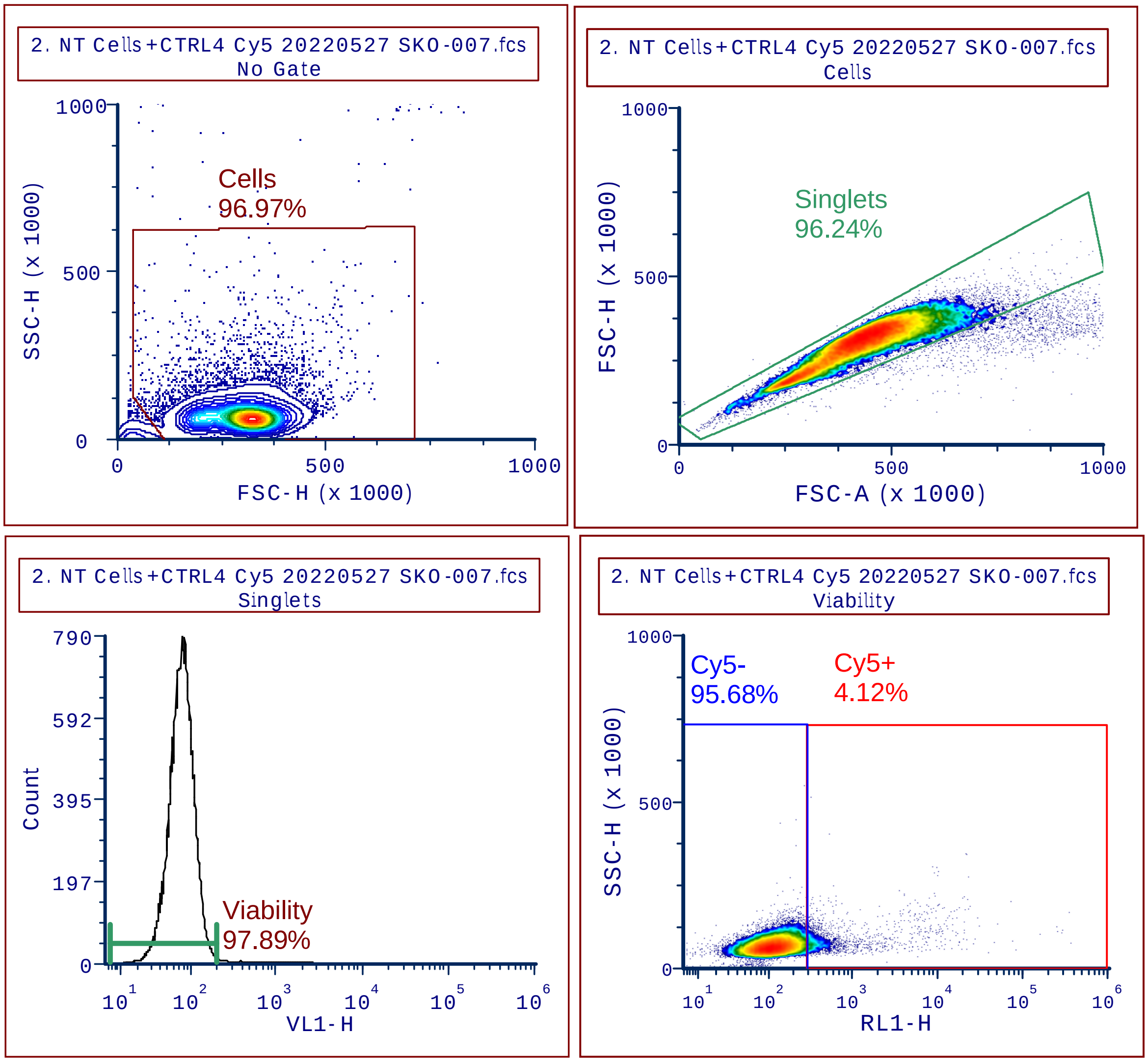

	A	B	C	D	E	F
1	**Filename**	**Gate**	**# of Events**	**% of all cells**	**% of gated cells**	
2	2. NT Cells+C	All Events	56411	100	100	
3	2. NT Cells+C	Cells	54701	96.97	96.97	
4	2. NT Cells+C	Singlets	52646	93.33	96.24	
5	2. NT Cells+C	Viability	51534	91.35	97.89	
6	2. NT Cells+C	Cy5+	2123	3.76	4.12	
7	2. NT Cells+C	Cy5-	49310	87.41	95.68	
8						
9						
10						

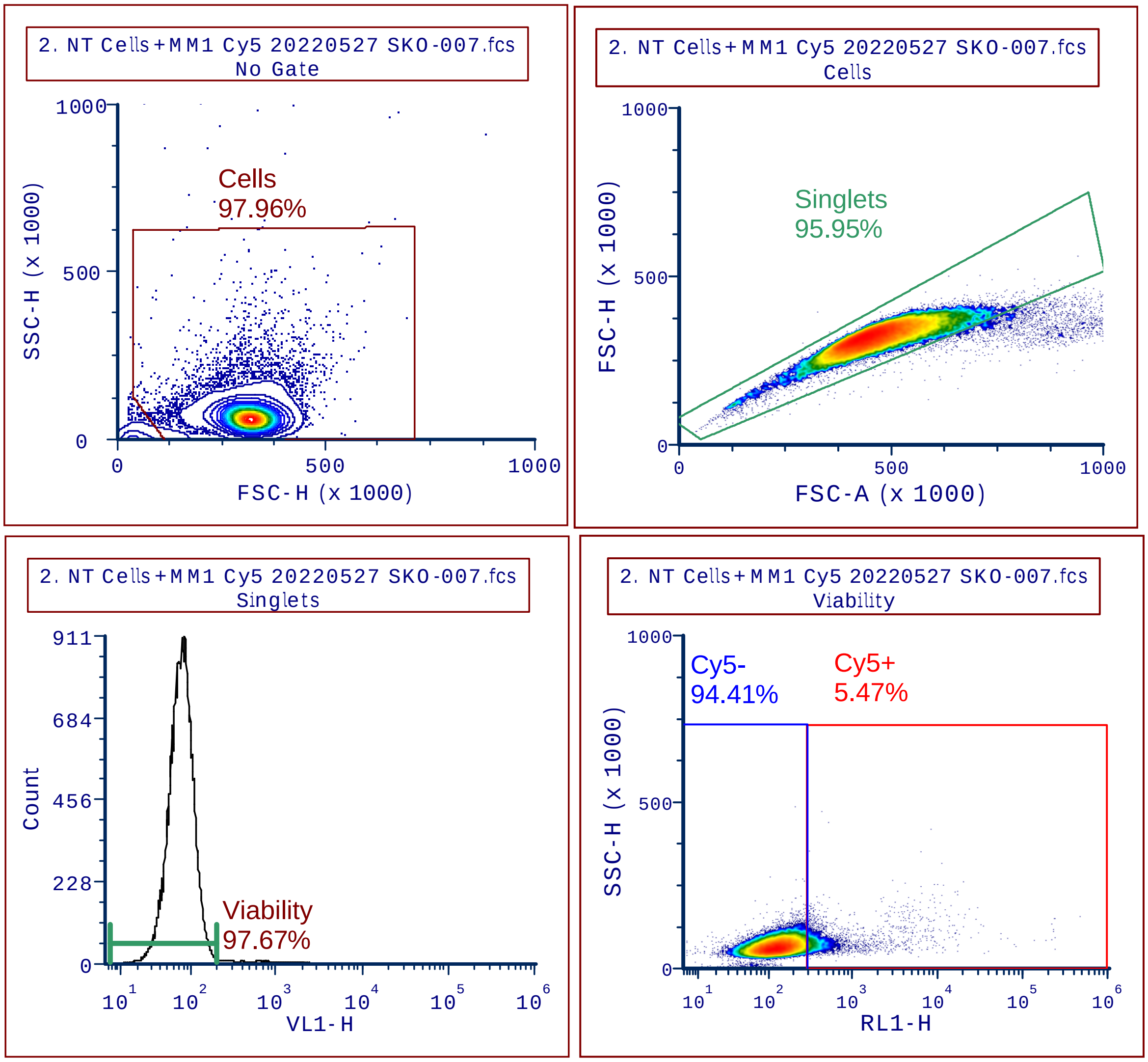

	A	B	C	D	E	F
1	**Filename**	**Gate**	**# of Events**	**% of all cells**	**% of gated cells**	
2	2. NT Cells+N	All Events	65579	100	100	
3	2. NT Cells+N	Cells	64240	97.96	97.96	
4	2. NT Cells+N	Singlets	61636	93.99	95.95	
5	2. NT Cells+N	Viability	60198	91.79	97.67	
6	2. NT Cells+N	Cy5+	3290	5.02	5.47	
7	2. NT Cells+N	Cy5-	56833	86.66	94.41	
8						
9						
10						

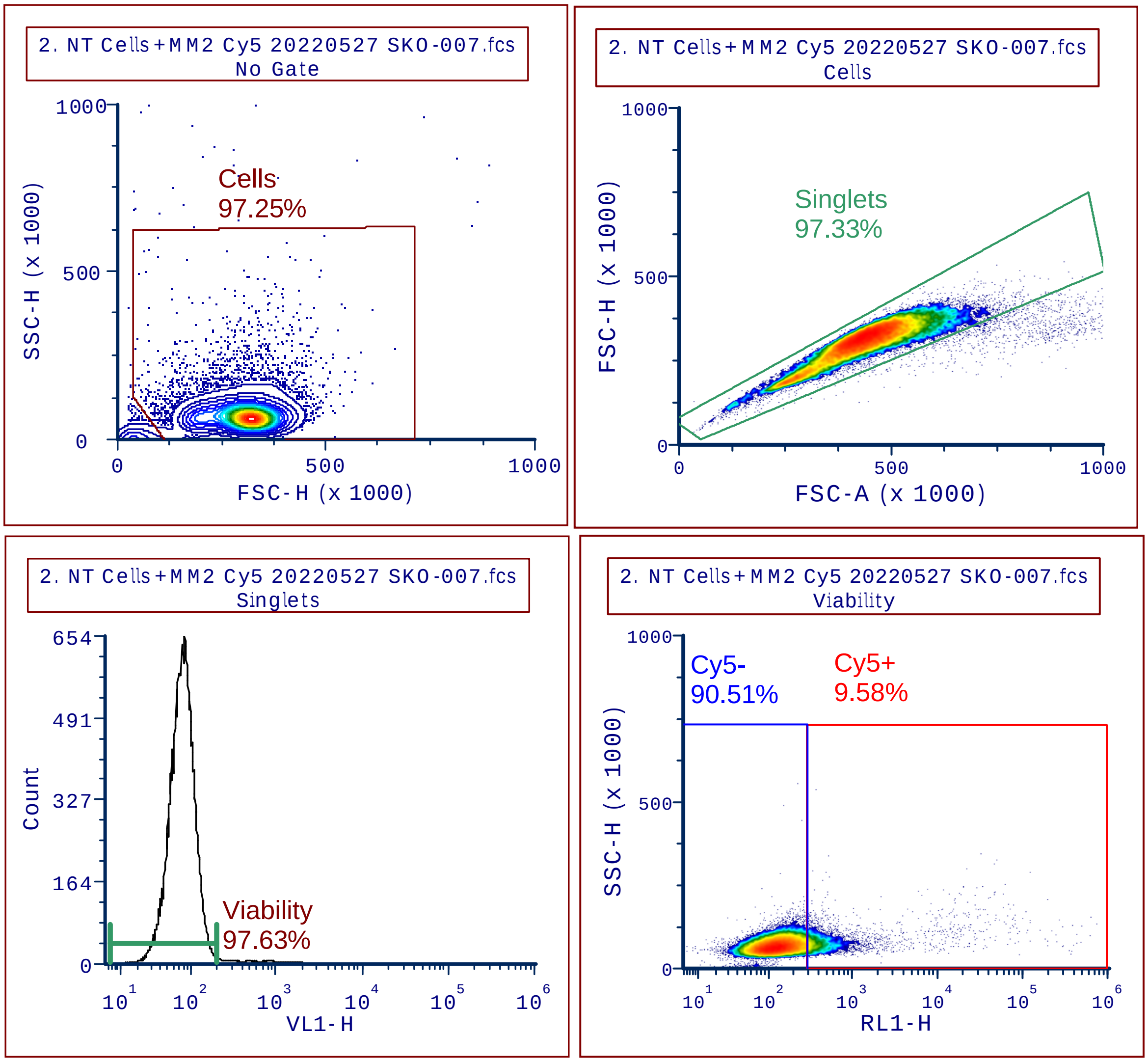

	A	B	C	D	E	F
1	**Filename**	**Gate**	**# of Events**	**% of all cells**	**% of gated cells**	
2	2. NT Cells+M	All Events	46083	100	100	
3	2. NT Cells+M	Cells	44815	97.25	97.25	
4	2. NT Cells+M	Singlets	43619	94.65	97.33	
5	2. NT Cells+M	Viability	42586	92.41	97.63	
6	2. NT Cells+M	Cy5+	4081	8.86	9.58	
7	2. NT Cells+M	Cy5-	38545	83.64	90.51	
8						
9						
10						

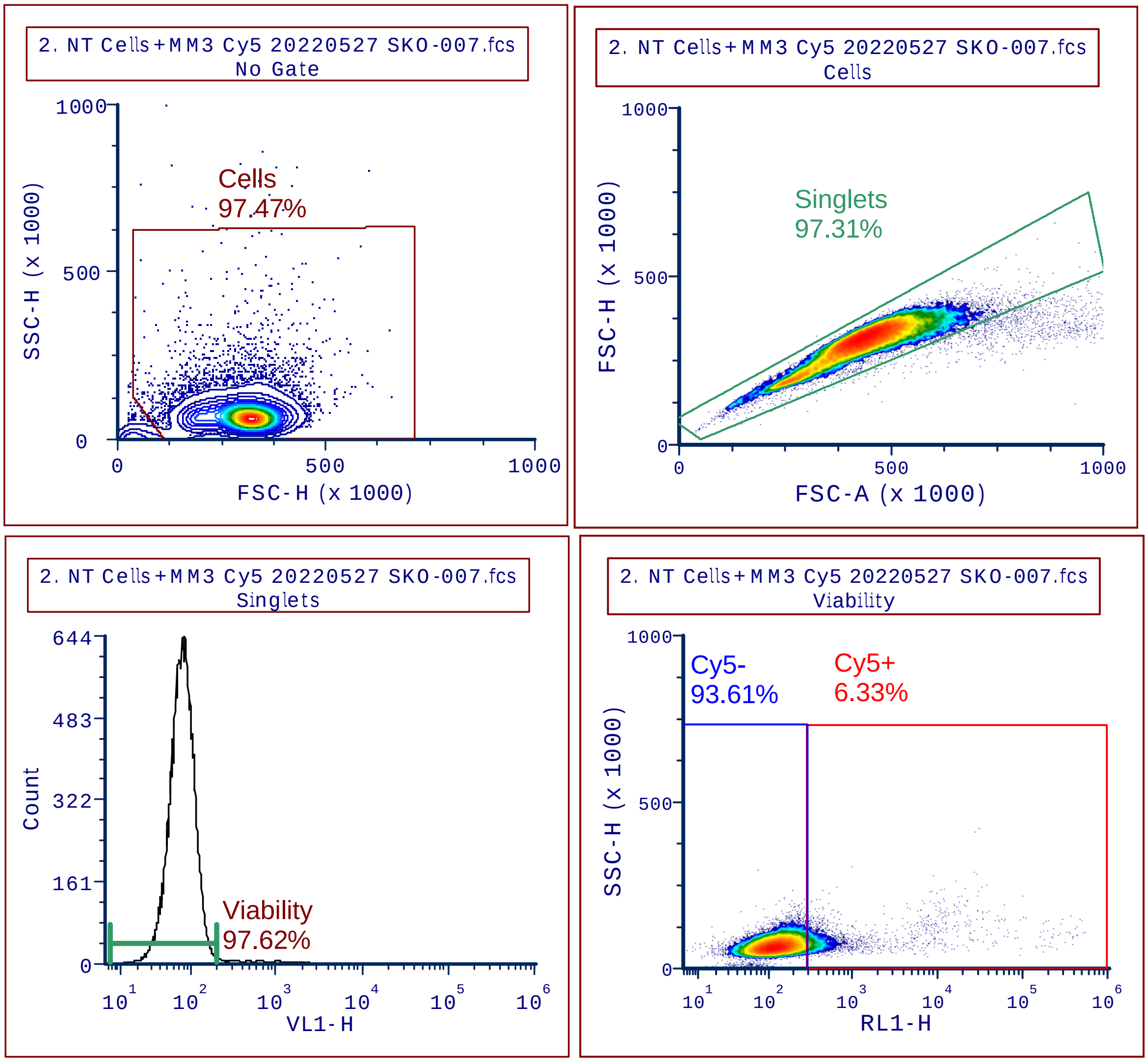

	A	B	C	D	E	F
1	**Filename**	**Gate**	**# of Events**	**% of all cells**	**% of gated cells**	
2	2. NT Cells+M	All Events	46601	100	100	
3	2. NT Cells+M	Cells	45424	97.47	97.47	
4	2. NT Cells+M	Singlets	44204	94.86	97.31	
5	2. NT Cells+M	Viability	43154	92.6	97.62	
6	2. NT Cells+M	Cy5+	2730	5.86	6.33	
7	2. NT Cells+M	Cy5-	40397	86.69	93.61	
8						
9						
10						

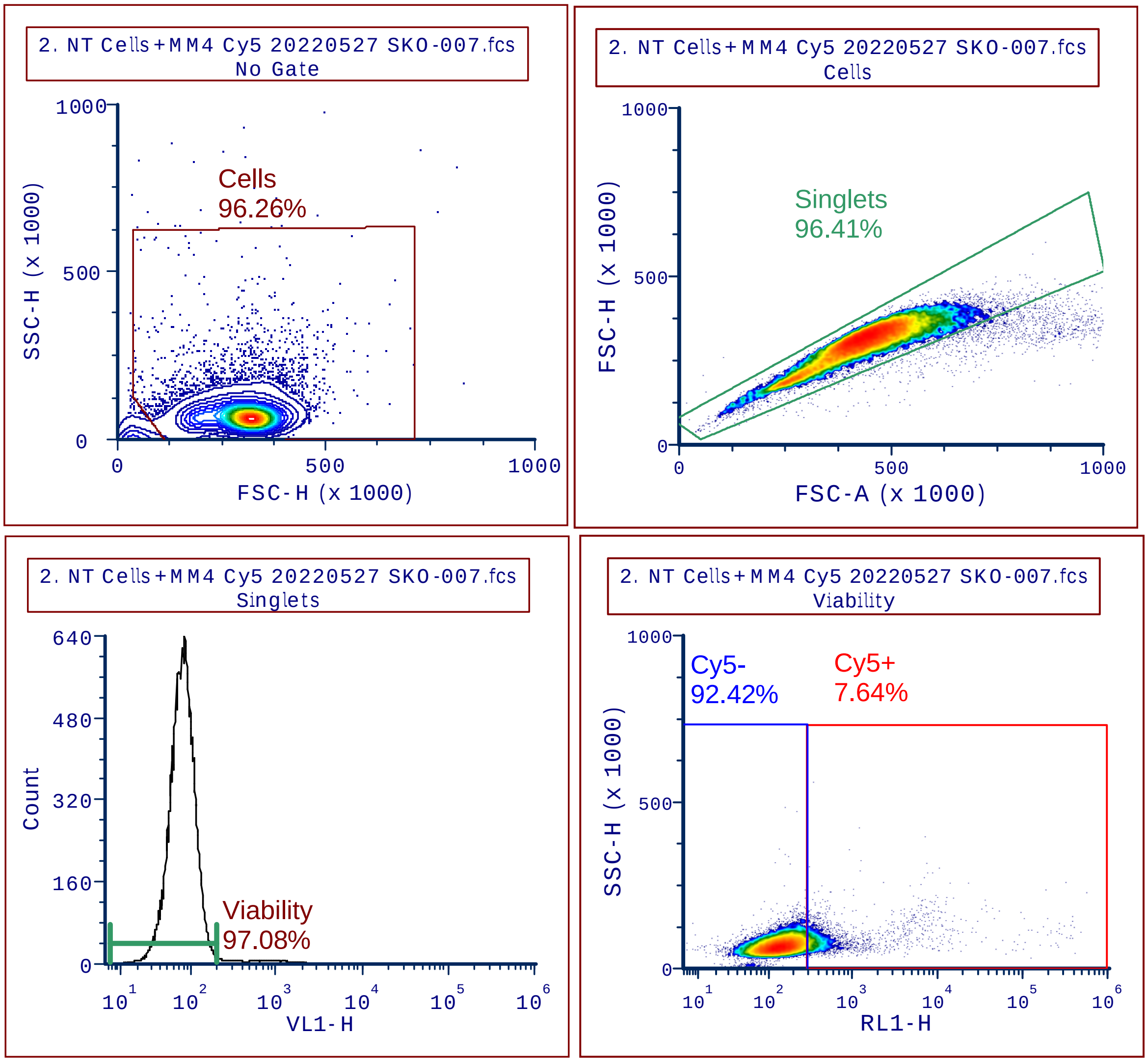

	A	B	C	D	E	F
1	**Filename**	**Gate**	**# of Events**	**% of all cells**	**% of gated cells**	
2	2. NT Cells+M	All Events	46681	100	100	
3	2. NT Cells+M	Cells	44934	96.26	96.26	
4	2. NT Cells+M	Singlets	43322	92.8	96.41	
5	2. NT Cells+M	Viability	42055	90.09	97.08	
6	2. NT Cells+M	Cy5+	3215	6.89	7.64	
7	2. NT Cells+M	Cy5-	38866	83.26	92.42	
8						
9						
10						

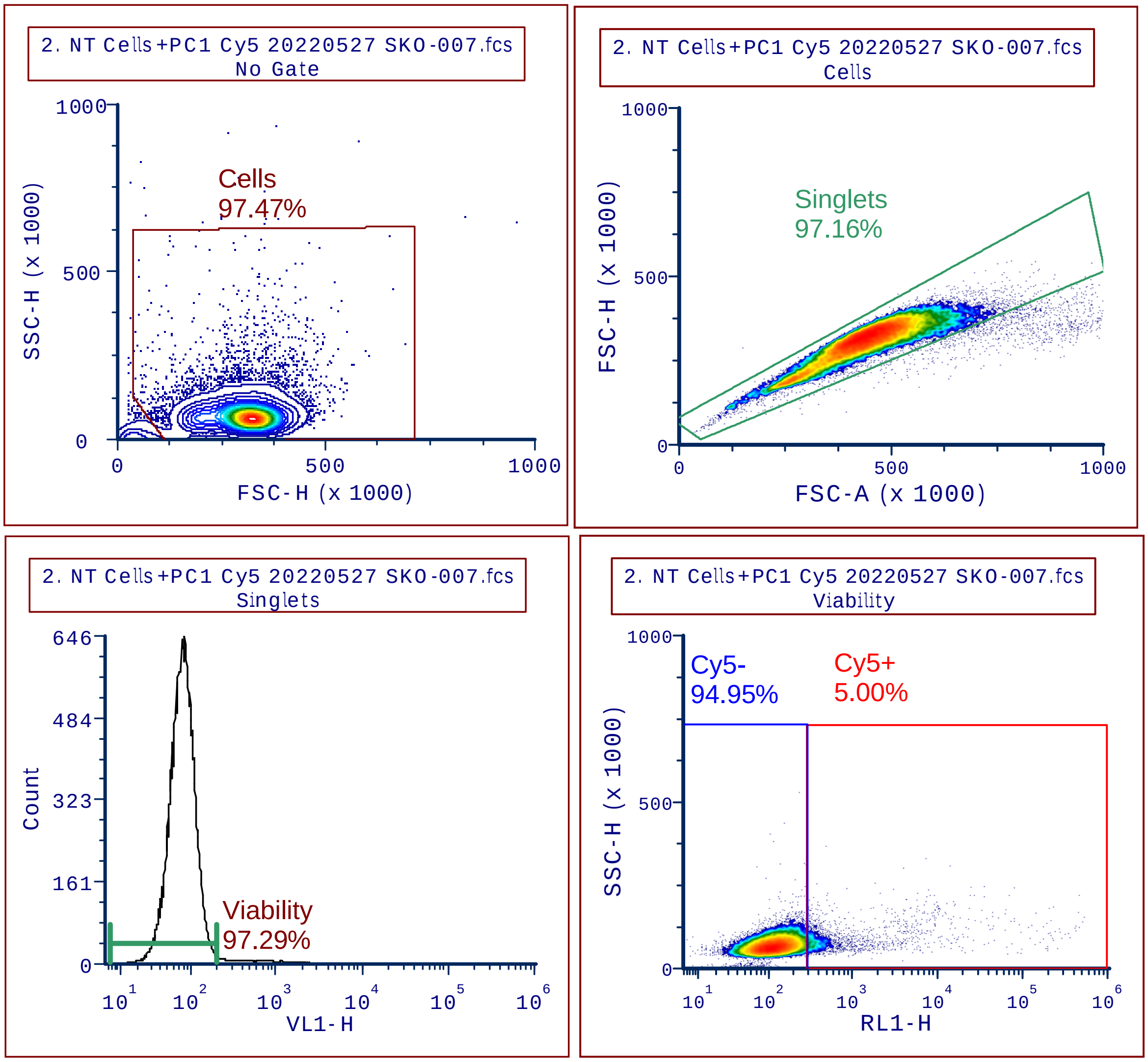

Filename	Gate	# of Events	% of all cells	% of gated cells
2. NT Cells+P	All Events	46659	100	100
2. NT Cells+P	Cells	45477	97.47	97.47
2. NT Cells+P	Singlets	44185	94.7	97.16
2. NT Cells+P	Viability	42989	92.13	97.29
2. NT Cells+P	Cy5+	2150	4.61	5
2. NT Cells+P	Cy5-	40816	87.48	94.95

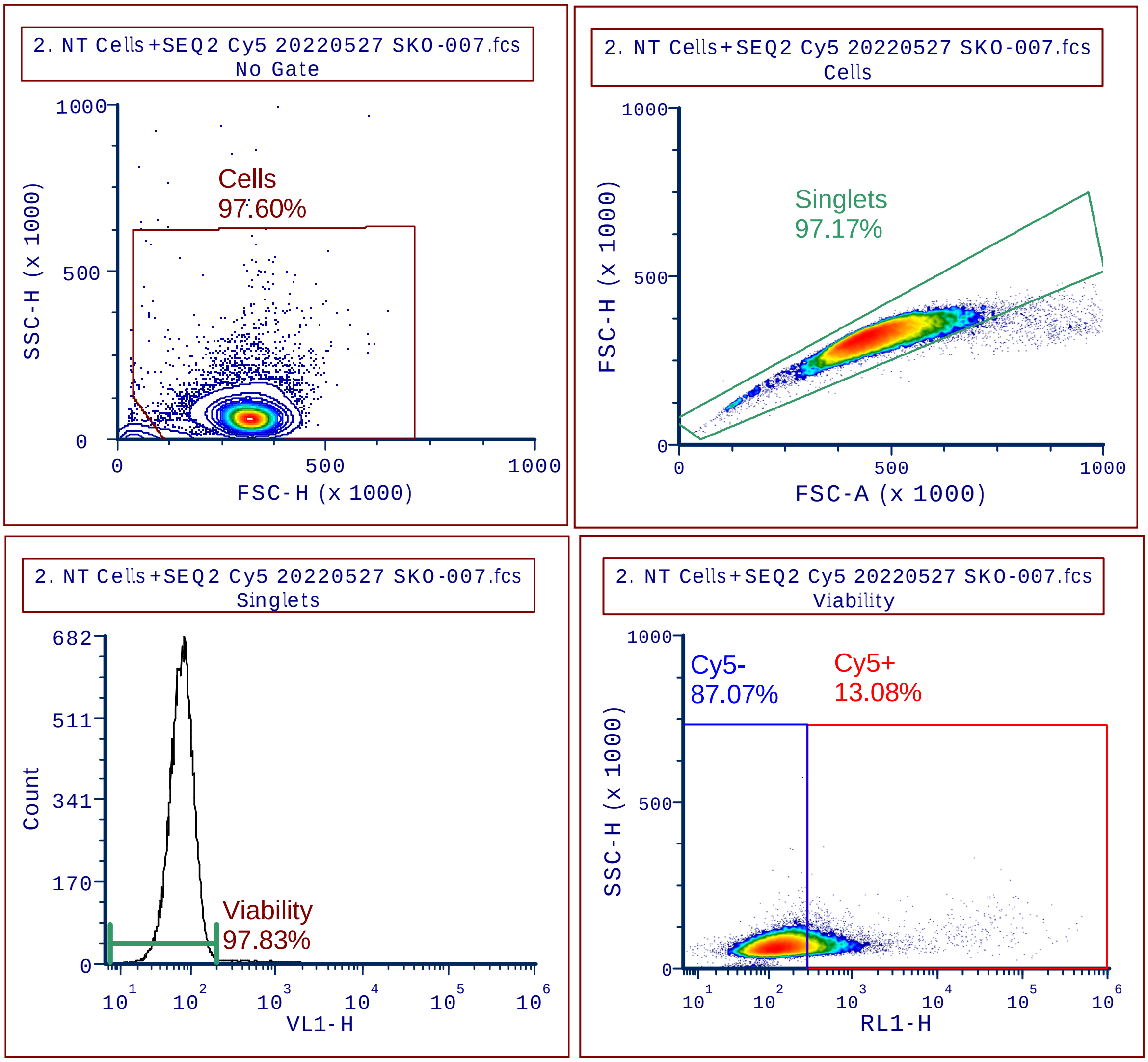

	A	B	C	D	E	F
1	**Filename**	**Gate**	**# of Events**	**% of all cells**	**% of gated cells**	
2	2. NT Cells+SI	All Events	47475	100	100	
3	2. NT Cells+SI	Cells	46337	97.6	97.6	
4	2. NT Cells+SI	Singlets	45024	94.84	97.17	
5	2. NT Cells+SI	Viability	44045	92.78	97.83	
6	2. NT Cells+SI	Cy5+	5759	12.13	13.08	
7	2. NT Cells+SI	Cy5-	38352	80.78	87.07	
8						
9						
10						

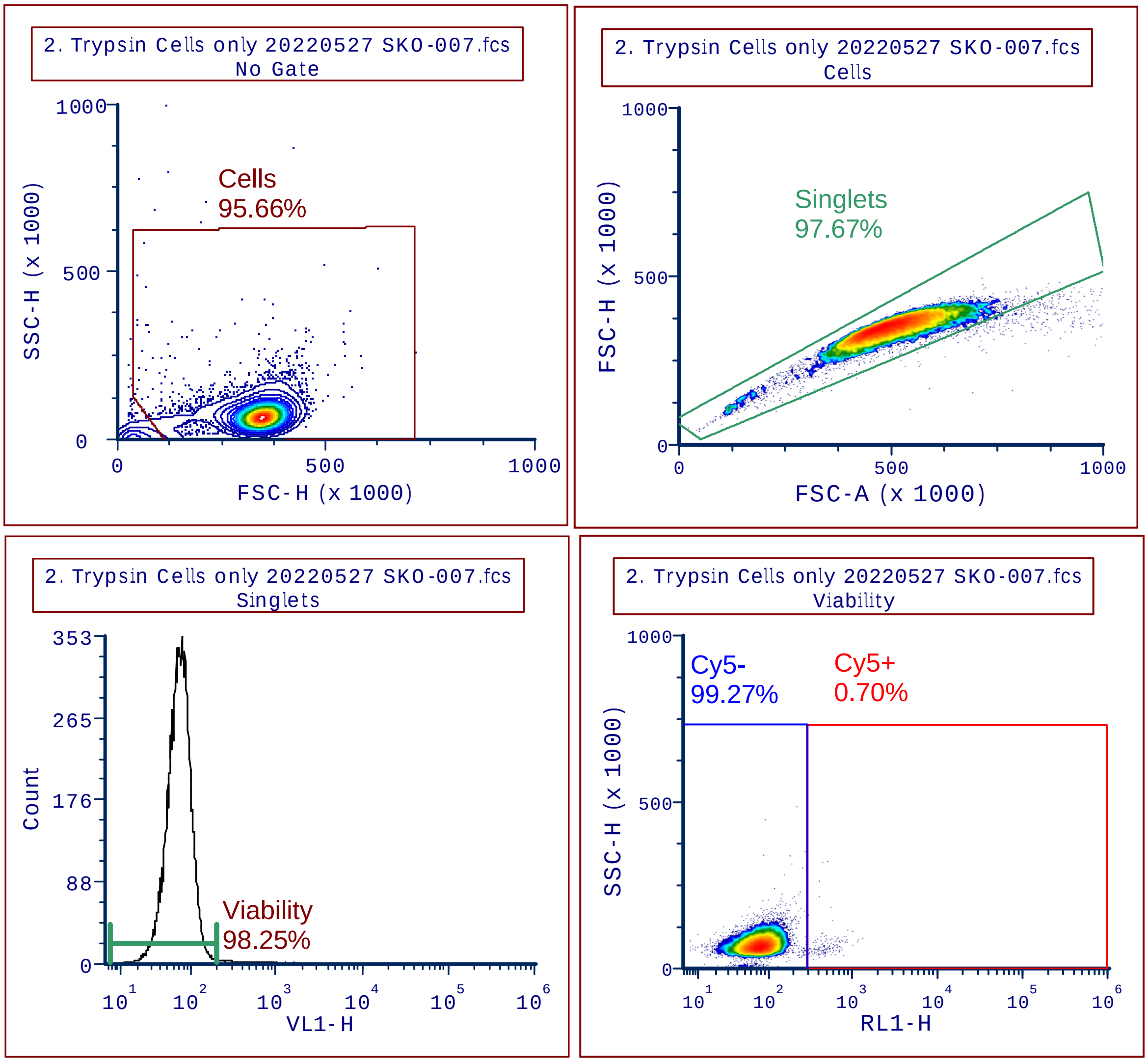

	A	B	C	D	E	F
1	**Filename**	**Gate**	**# of Events**	**% of all cells**	**% of gated cells**	
2	2. Trypsin Cel	All Events	24097	100	100	
3	2. Trypsin Cel	Cells	23050	95.66	95.66	
4	2. Trypsin Cel	Singlets	22512	93.42	97.67	
5	2. Trypsin Cel	Viability	22118	91.79	98.25	
6	2. Trypsin Cel	Cy5+	155	0.64	0.7	
7	2. Trypsin Cel	Cy5-	21957	91.12	99.27	
8						
9						
10						

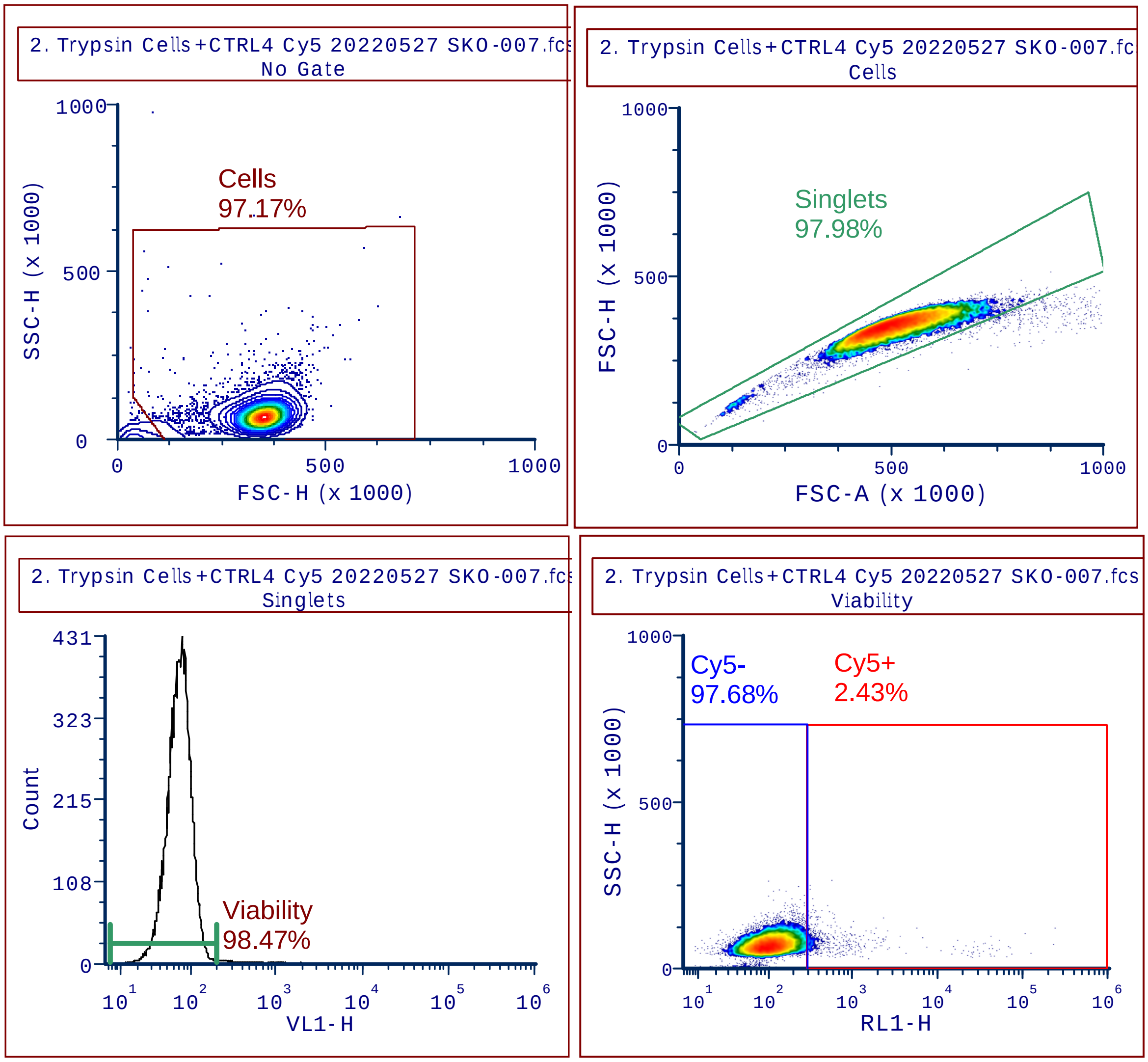

	A	B	C	D	E	F
1	**Filename**	**Gate**	**# of Events**	**% of all cells**	**% of gated cells**	
2	2. Trypsin Cel	All Events	28736	100	100	
3	2. Trypsin Cel	Cells	27922	97.17	97.17	
4	2. Trypsin Cel	Singlets	27357	95.2	97.98	
5	2. Trypsin Cel	Viability	26938	93.74	98.47	
6	2. Trypsin Cel	Cy5+	655	2.28	2.43	
7	2. Trypsin Cel	Cy5-	26312	91.56	97.68	
8						
9						
10						

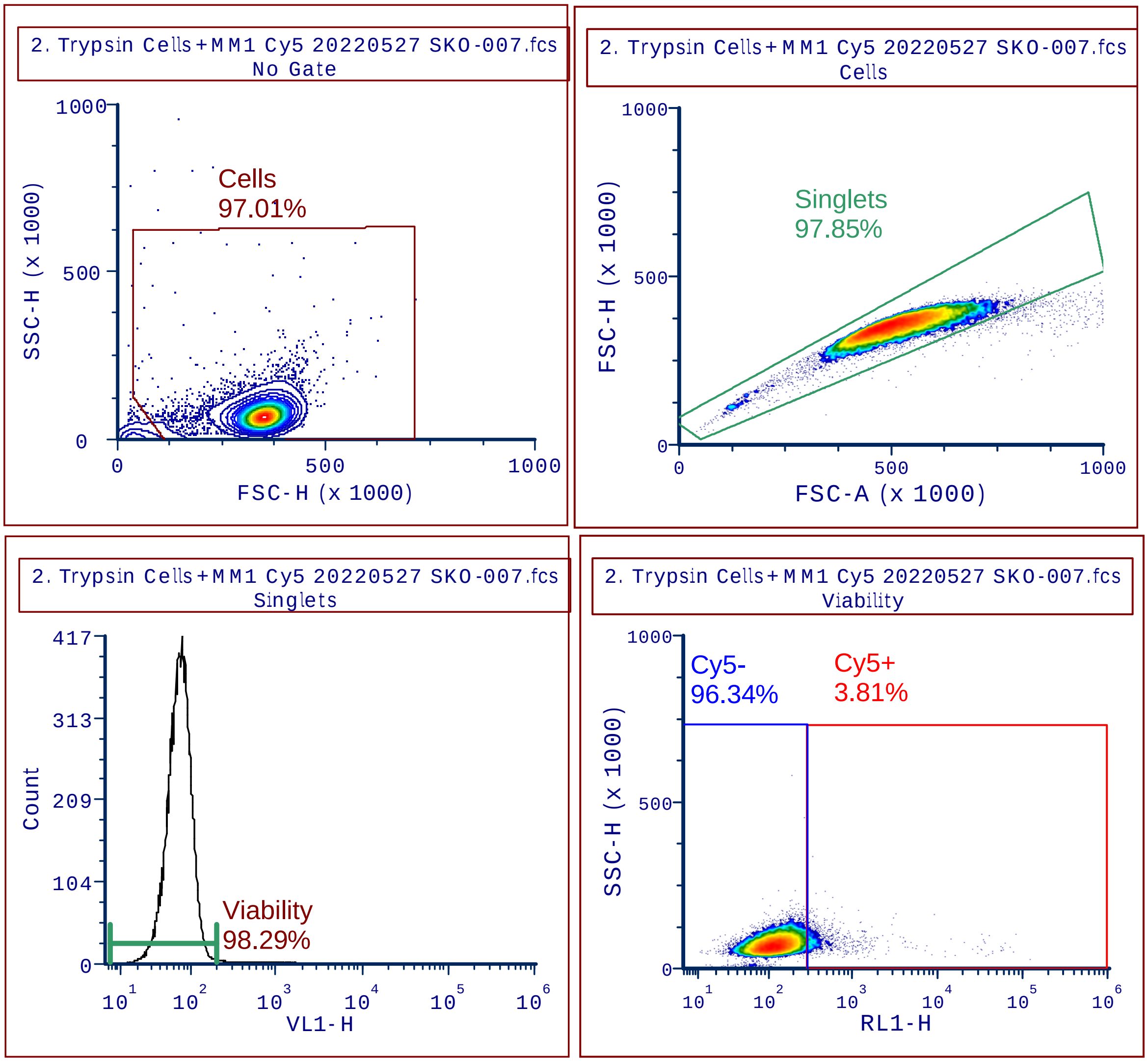

	A	B	C	D	E	F
1	**Filename**	**Gate**	**# of Events**	**% of all cells**	**% of gated cells**	
2	2. Trypsin Cel	All Events	27919	100	100	
3	2. Trypsin Cel	Cells	27083	97.01	97.01	
4	2. Trypsin Cel	Singlets	26500	94.92	97.85	
5	2. Trypsin Cel	Viability	26047	93.29	98.29	
6	2. Trypsin Cel	Cy5+	993	3.56	3.81	
7	2. Trypsin Cel	Cy5-	25093	89.88	96.34	
8						
9						
10						

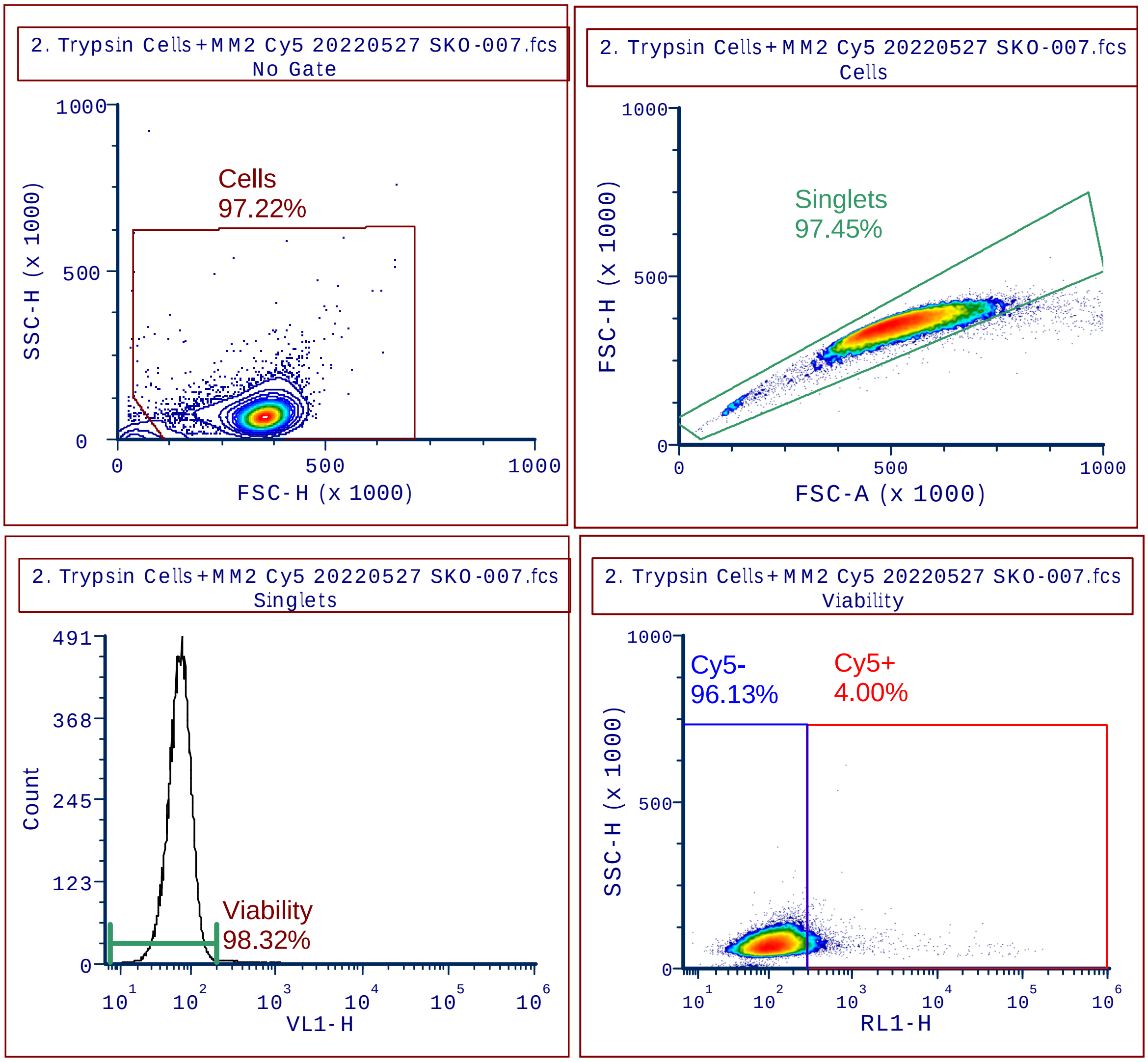

	A	B	C	D	E	F
1	**Filename**	**Gate**	**# of Events**	**% of all cells**	**% of gated cells**	
2	2. Trypsin Cel	All Events	32535	100	100	
3	2. Trypsin Cel	Cells	31630	97.22	97.22	
4	2. Trypsin Cel	Singlets	30823	94.74	97.45	
5	2. Trypsin Cel	Viability	30306	93.15	98.32	
6	2. Trypsin Cel	Cy5+	1212	3.73	4	
7	2. Trypsin Cel	Cy5-	29132	89.54	96.13	
8						
9						
10						

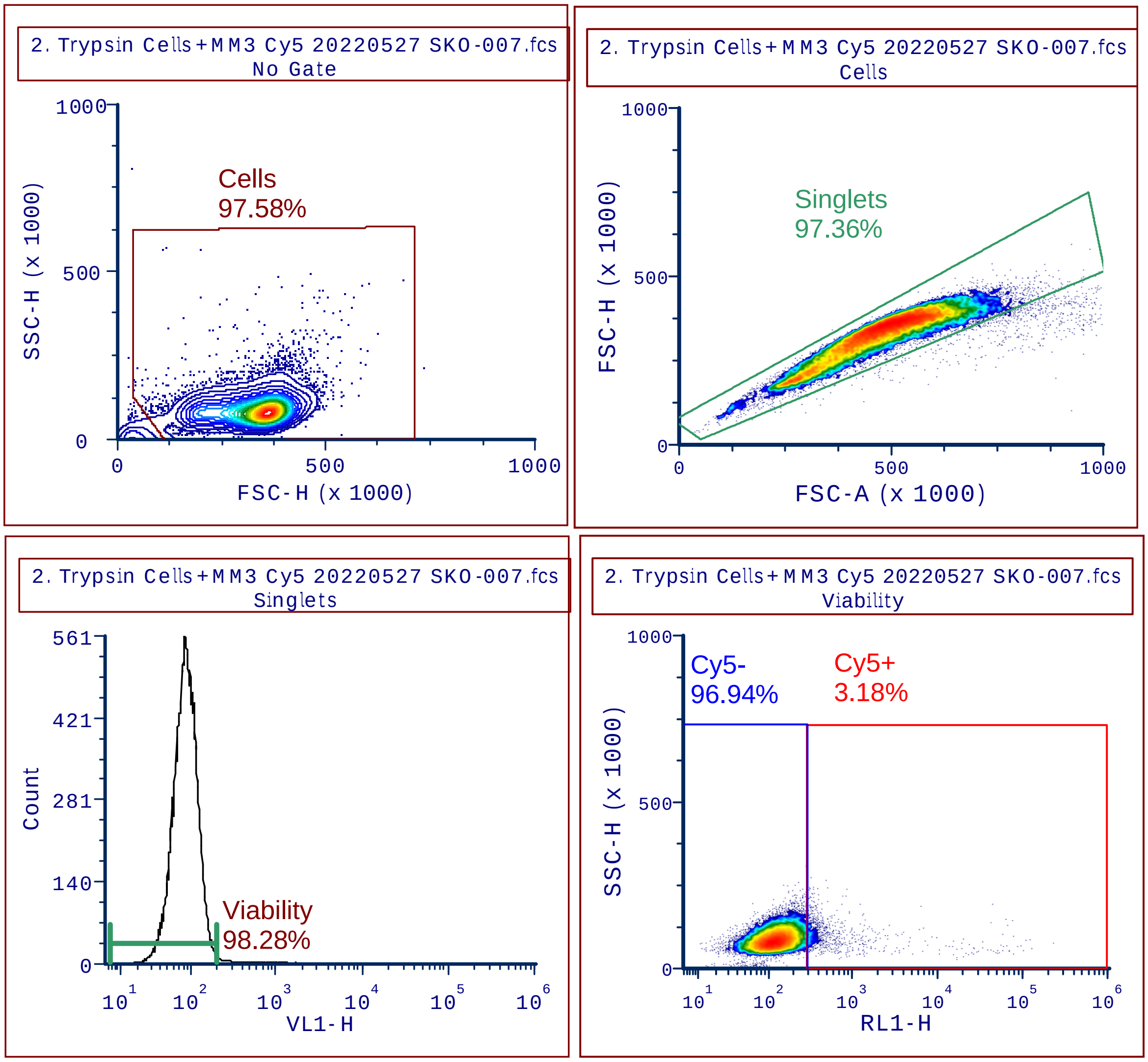

	A	B	C	D	E	F
1	**Filename**	**Gate**	**# of Events**	**% of all cells**	**% of gated cells**	
2	2. Trypsin Cel	All Events	38305	100	100	
3	2. Trypsin Cel	Cells	37379	97.58	97.58	
4	2. Trypsin Cel	Singlets	36394	95.01	97.36	
5	2. Trypsin Cel	Viability	35767	93.37	98.28	
6	2. Trypsin Cel	Cy5+	1137	2.97	3.18	
7	2. Trypsin Cel	Cy5-	34674	90.52	96.94	
8						
9						
10						

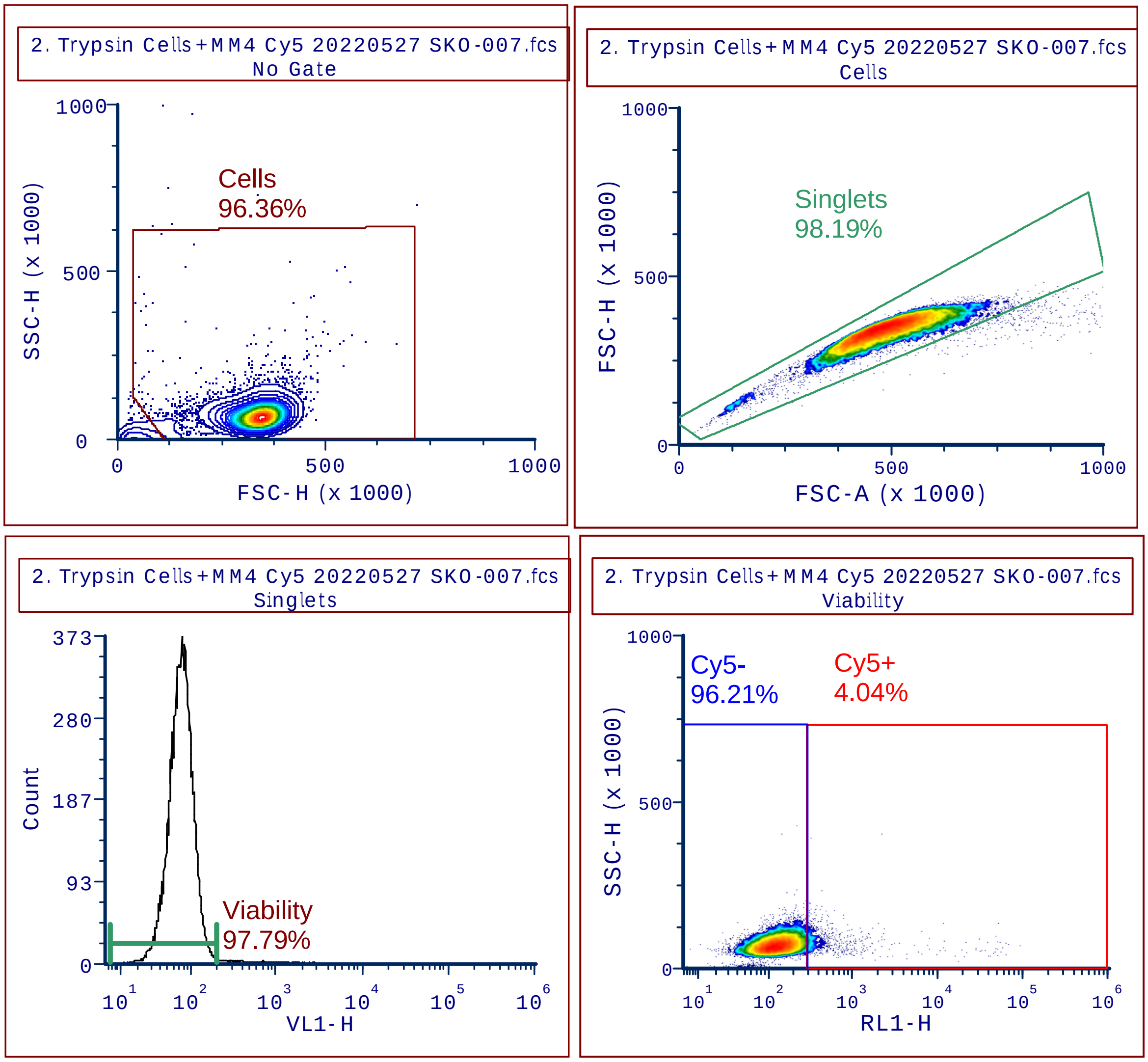

	A	B	C	D	E	F
1	**Filename**	**Gate**	**# of Events**	**% of all cells**	**% of gated cells**	
2	2. Trypsin Cel	All Events	25498	100	100	
3	2. Trypsin Cel	Cells	24569	96.36	96.36	
4	2. Trypsin Cel	Singlets	24124	94.61	98.19	
5	2. Trypsin Cel	Viability	23590	92.52	97.79	
6	2. Trypsin Cel	Cy5+	952	3.73	4.04	
7	2. Trypsin Cel	Cy5-	22696	89.01	96.21	
8						
9						
10						

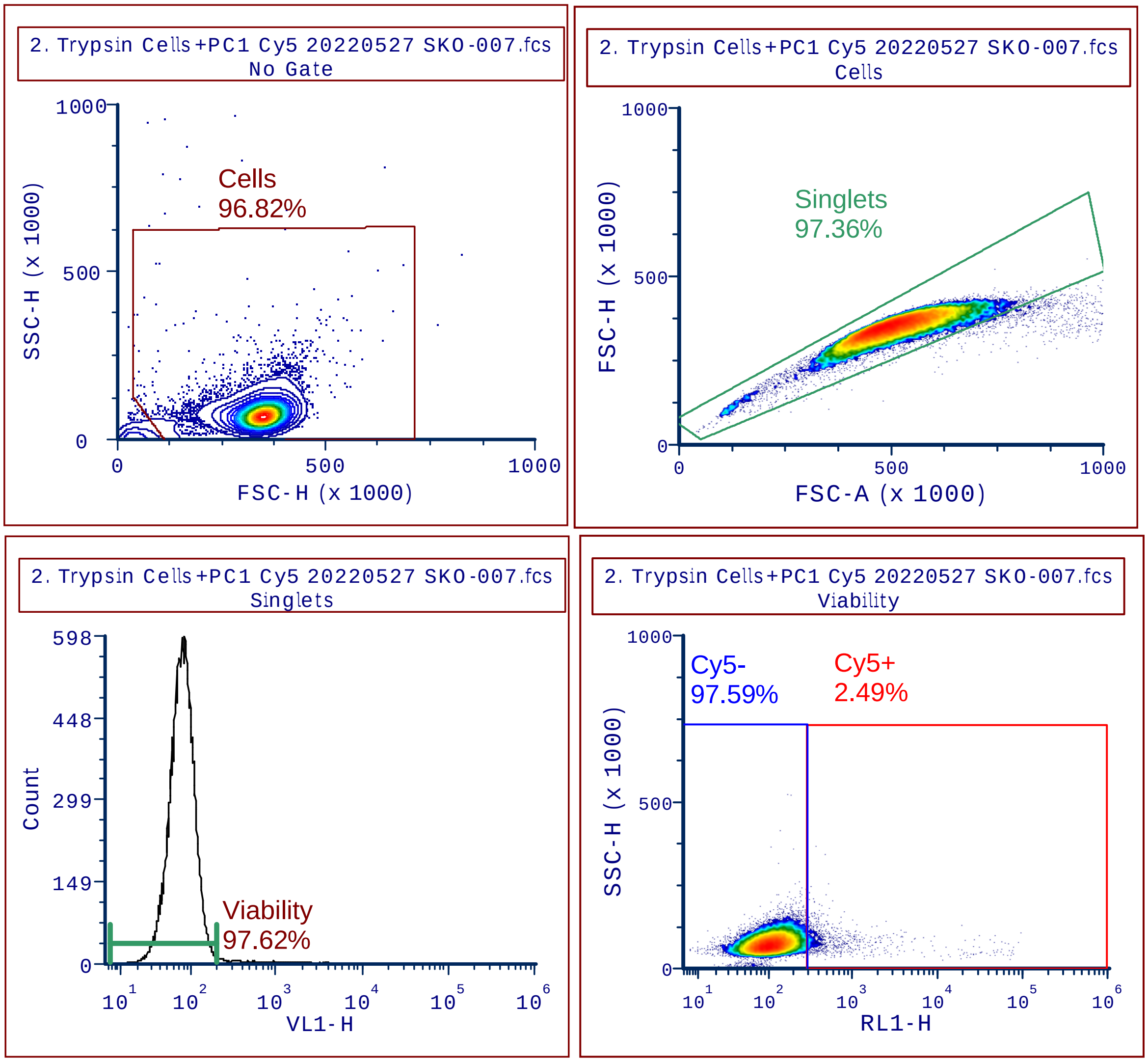

	A	B	C	D	E	F
1	**Filename**	**Gate**	**# of Events**	**% of all cells**	**% of gated cells**	
2	2. Trypsin Cel	All Events	43253	100	100	
3	2. Trypsin Cel	Cells	41878	96.82	96.82	
4	2. Trypsin Cel	Singlets	40771	94.26	97.36	
5	2. Trypsin Cel	Viability	39802	92.02	97.62	
6	2. Trypsin Cel	Cy5+	993	2.3	2.49	
7	2. Trypsin Cel	Cy5-	38843	89.8	97.59	
8						
9						
10						

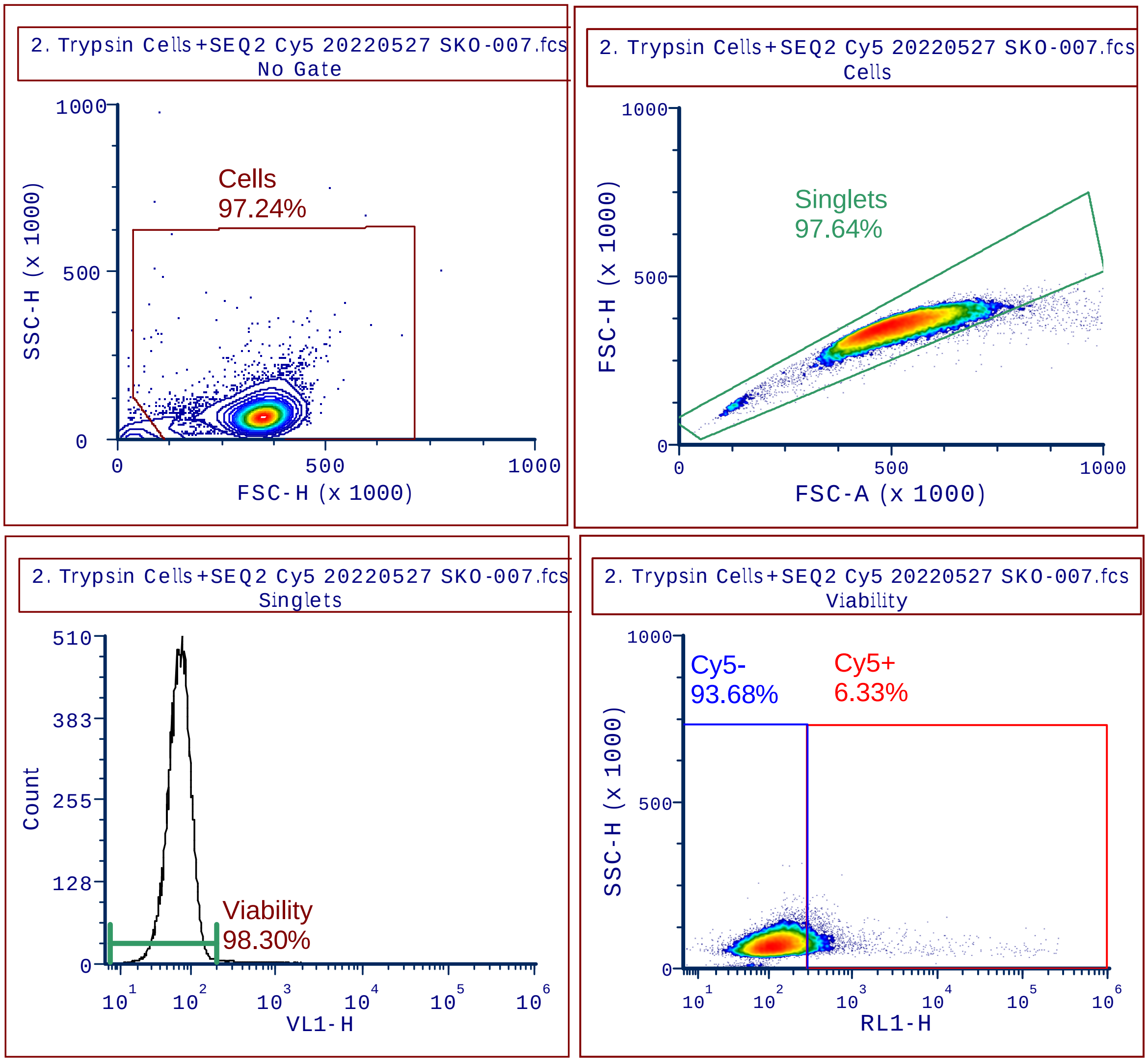

	A	B	C	D	E	F
1	**Filename**	**Gate**	**# of Events**	**% of all cells**	**% of gated cells**	
2	2. Trypsin Cel	All Events	34527	100	100	
3	2. Trypsin Cel	Cells	33575	97.24	97.24	
4	2. Trypsin Cel	Singlets	32783	94.95	97.64	
5	2. Trypsin Cel	Viability	32226	93.34	98.3	
6	2. Trypsin Cel	Cy5+	2039	5.91	6.33	
7	2. Trypsin Cel	Cy5-	30188	87.43	93.68	
8						
9						
10						

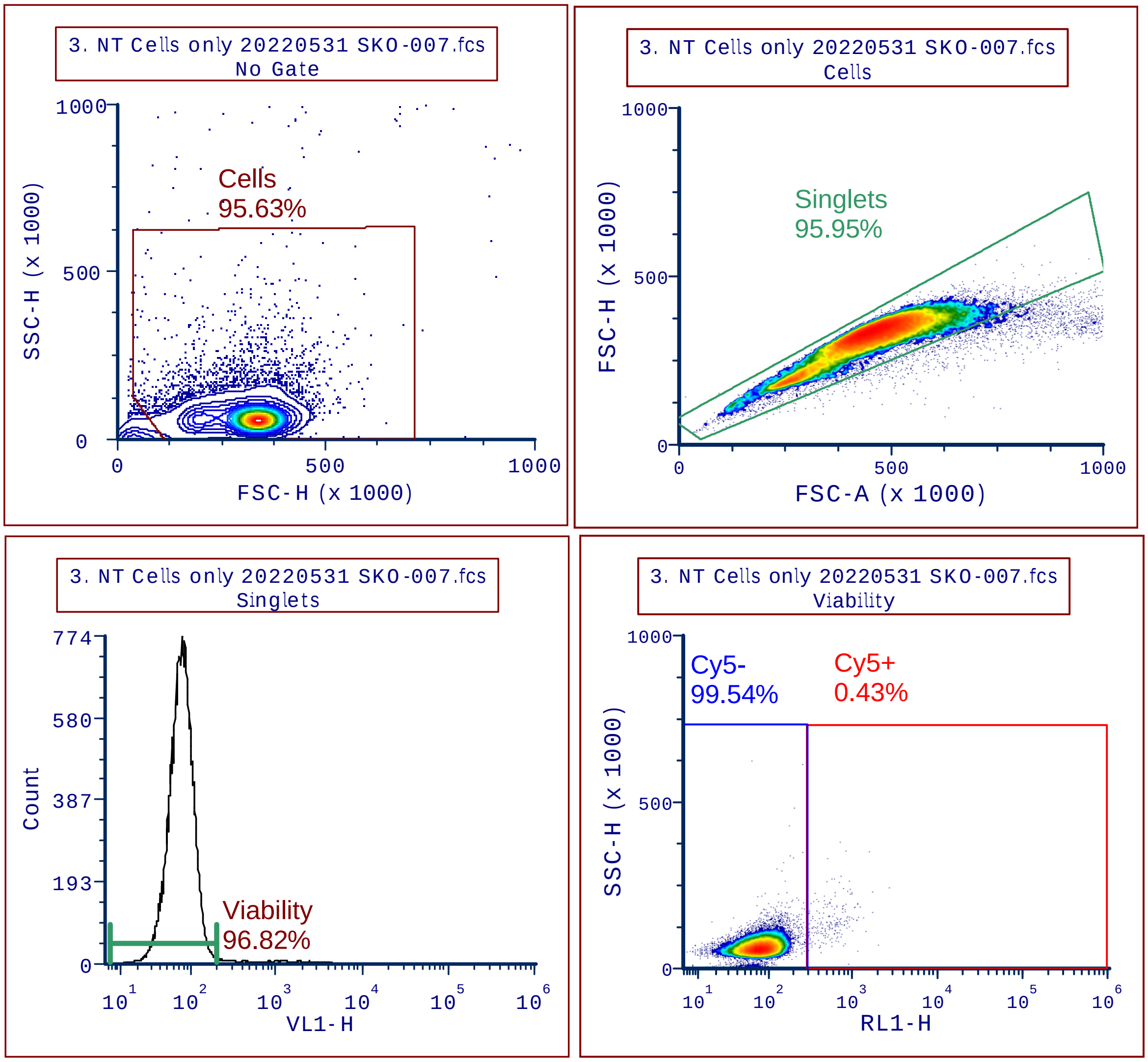

	A	B	C	D	E	F
1	**Filename**	**Gate**	**# of Events**	**% of all cells**	**% of gated cells**	
2	3. NT Cells or	All Events	55865	100	100	
3	3. NT Cells or	Cells	53421	95.63	95.63	
4	3. NT Cells or	Singlets	51257	91.75	95.95	
5	3. NT Cells or	Viability	49629	88.84	96.82	
6	3. NT Cells or	Cy5+	212	0.38	0.43	
7	3. NT Cells or	Cy5-	49400	88.43	99.54	
8						
9						
10						

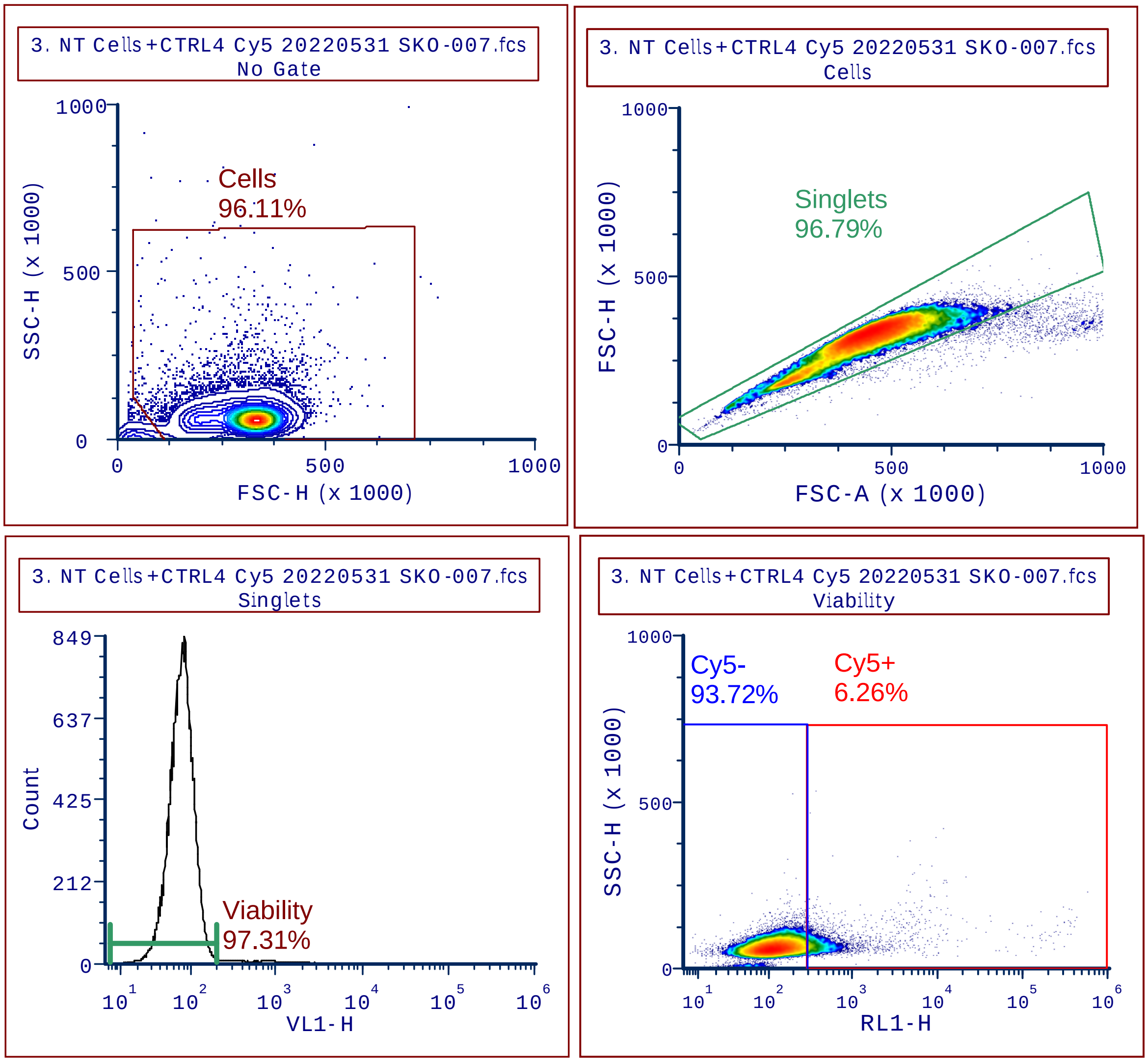

	A	B	C	D	E	F
1	**Filename**	**Gate**	**# of Events**	**% of all cells**	**% of gated cells**	
2	3. NT Cells+C	All Events	59595	100	100	
3	3. NT Cells+C	Cells	57279	96.11	96.11	
4	3. NT Cells+C	Singlets	55440	93.03	96.79	
5	3. NT Cells+C	Viability	53949	90.53	97.31	
6	3. NT Cells+C	Cy5+	3376	5.66	6.26	
7	3. NT Cells+C	Cy5-	50562	84.84	93.72	
8						
9						
10						

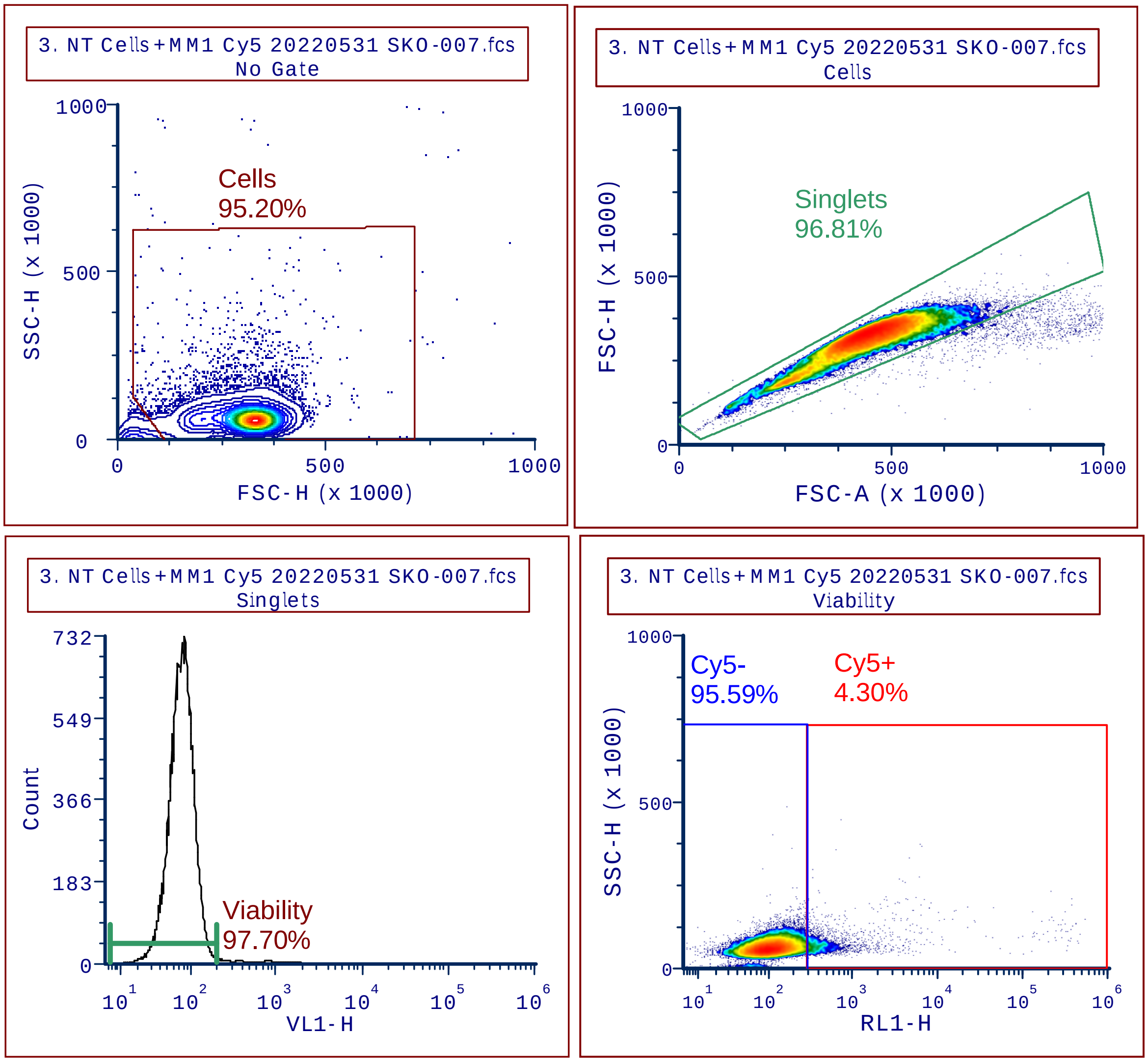

	A	B	C	D	E	F
1	**Filename**	**Gate**	**# of Events**	**% of all cells**	**% of gated cells**	
2	3. NT Cells+M	All Events	53215	100	100	
3	3. NT Cells+M	Cells	50661	95.2	95.2	
4	3. NT Cells+M	Singlets	49044	92.16	96.81	
5	3. NT Cells+M	Viability	47918	90.05	97.7	
6	3. NT Cells+M	Cy5+	2059	3.87	4.3	
7	3. NT Cells+M	Cy5-	45807	86.08	95.59	
8						
9						
10						

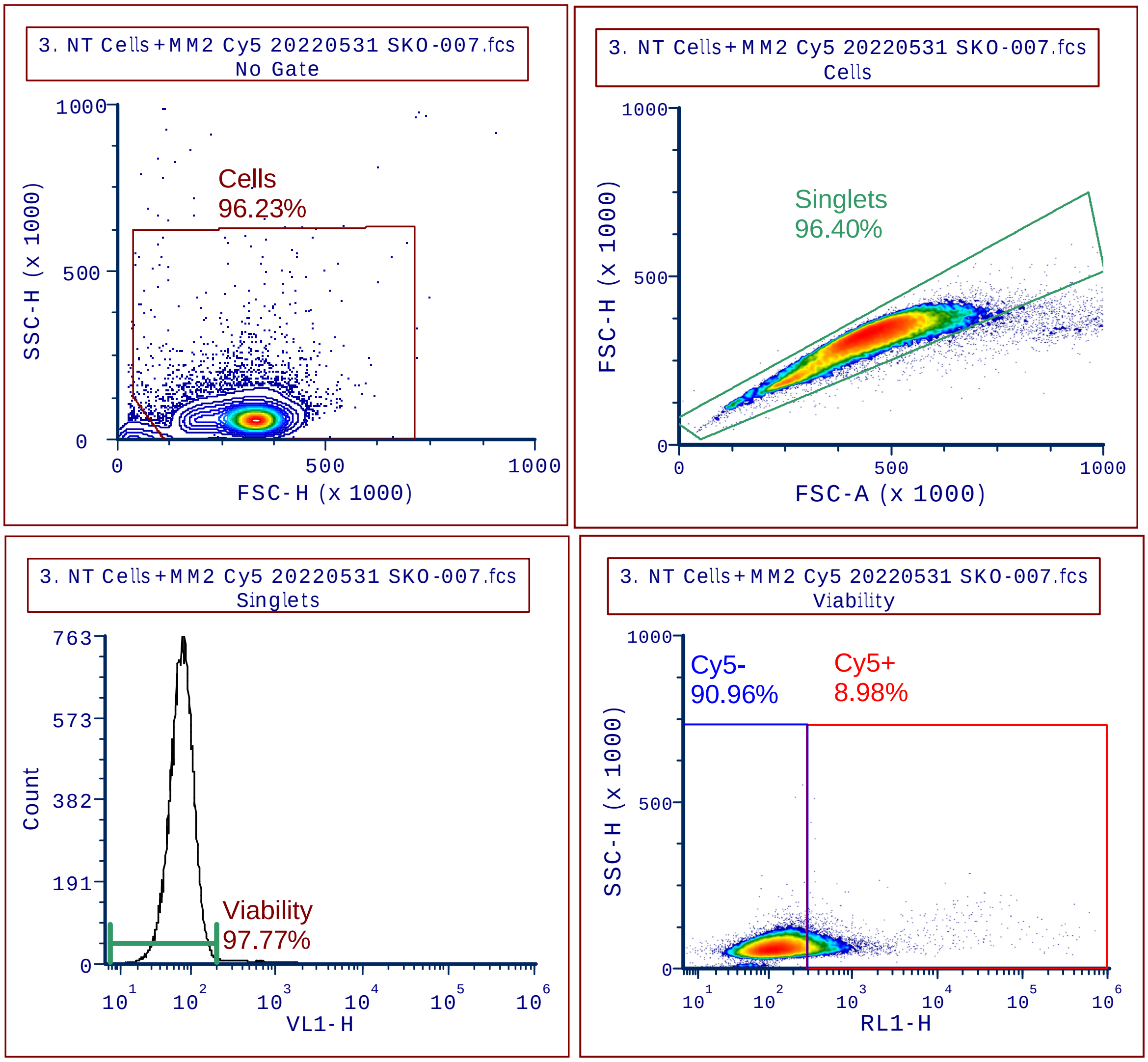

	A	B	C	D	E	F
1	**Filename**	**Gate**	**# of Events**	**% of all cells**	**% of gated cells**	
2	3. NT Cells+M	All Events	55157	100	100	
3	3. NT Cells+M	Cells	53076	96.23	96.23	
4	3. NT Cells+M	Singlets	51166	92.76	96.4	
5	3. NT Cells+M	Viability	50023	90.69	97.77	
6	3. NT Cells+M	Cy5+	4493	8.15	8.98	
7	3. NT Cells+M	Cy5-	45500	82.49	90.96	
8						
9						
10						

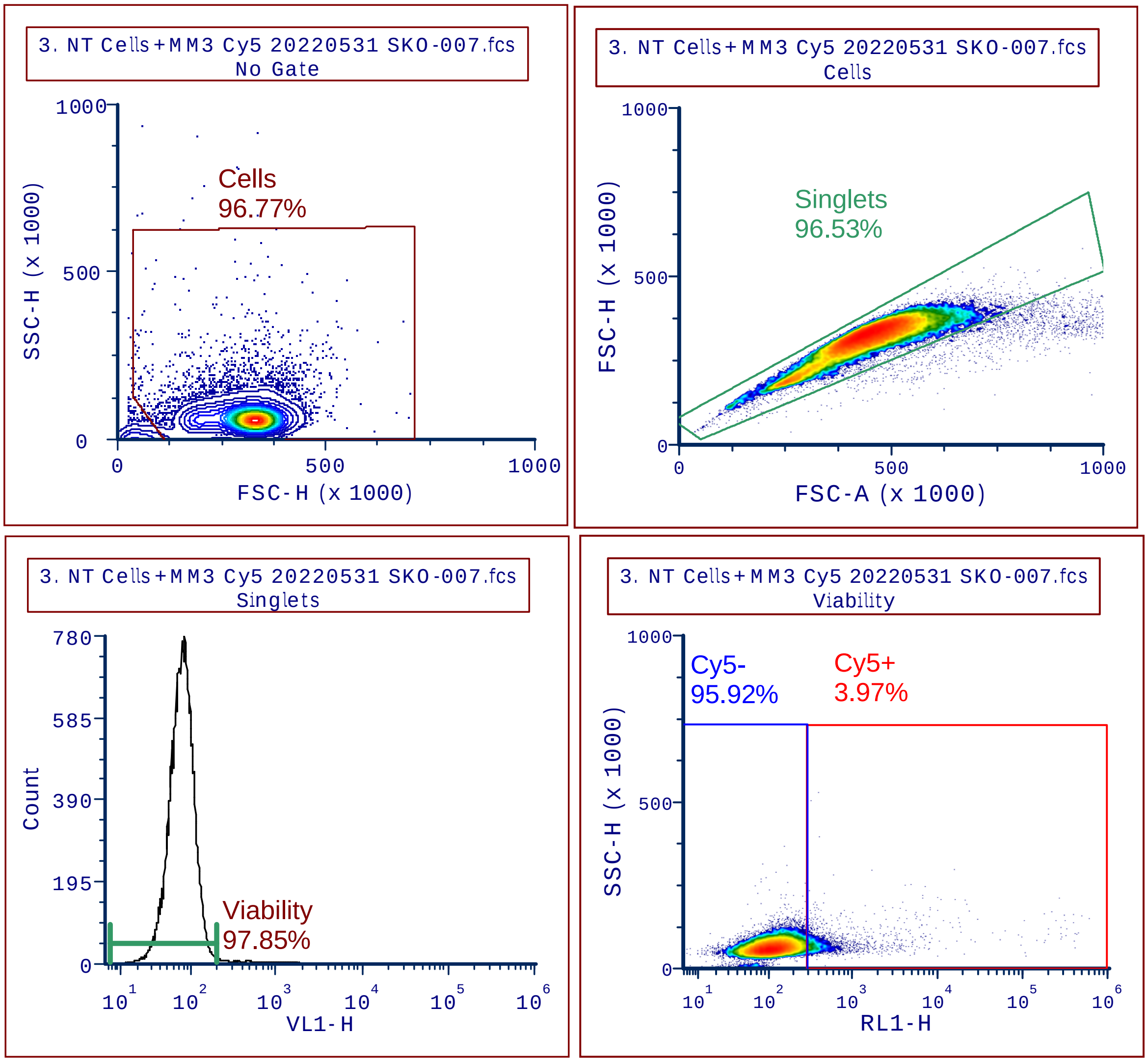

	A	B	C	D	E	F
1	**Filename**	**Gate**	**# of Events**	**% of all cells**	**% of gated cells**	
2	3. NT Cells+N	All Events	55238	100	100	
3	3. NT Cells+N	Cells	53454	96.77	96.77	
4	3. NT Cells+N	Singlets	51597	93.41	96.53	
5	3. NT Cells+N	Viability	50490	91.4	97.85	
6	3. NT Cells+N	Cy5+	2003	3.63	3.97	
7	3. NT Cells+N	Cy5-	48432	87.68	95.92	
8						
9						
10						

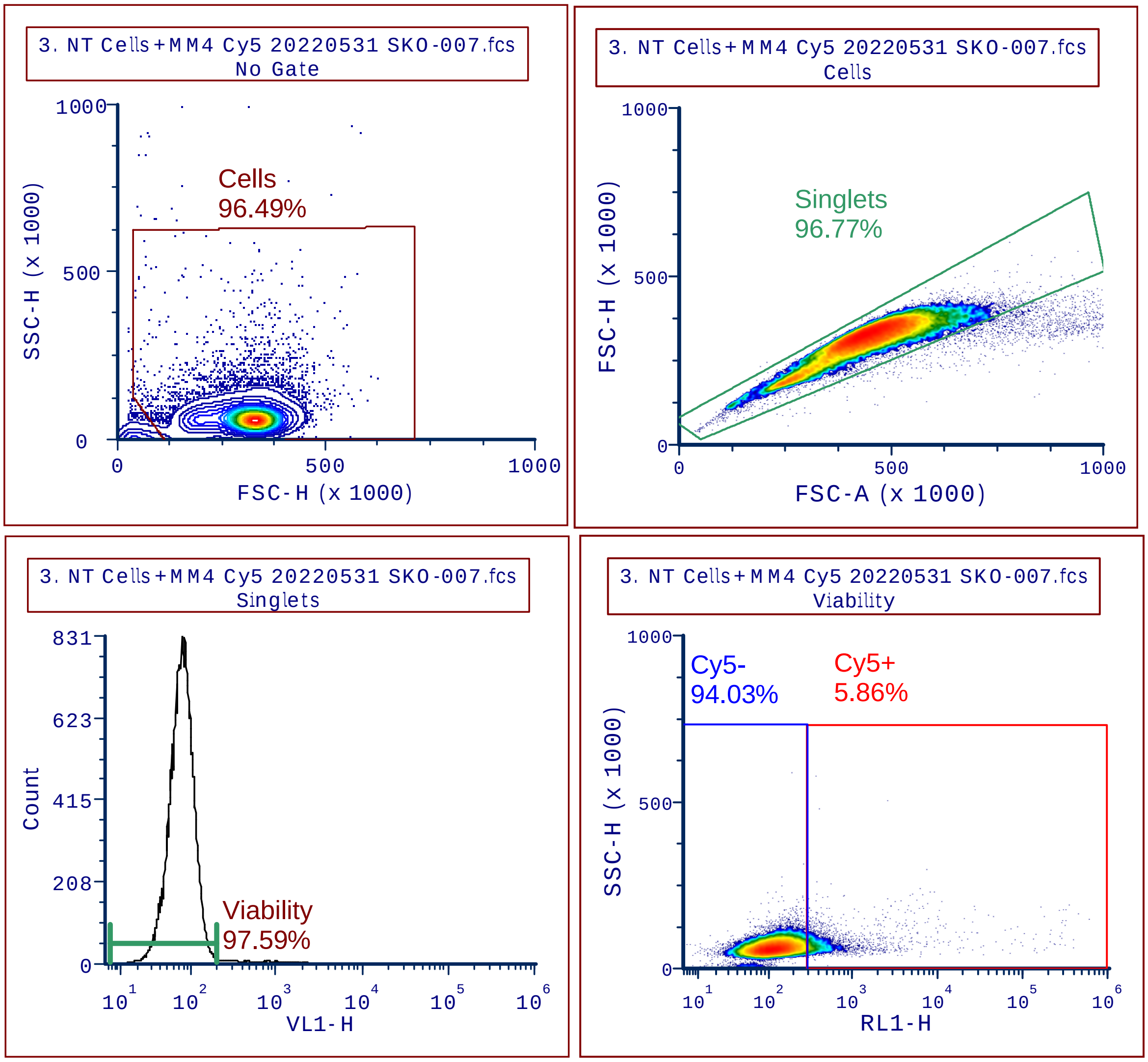

	A	B	C	D	E	F
1	**Filename**	**Gate**	**# of Events**	**% of all cells**	**% of gated cells**	
2	3. NT Cells+N	All Events	58695	100	100	
3	3. NT Cells+N	Cells	56635	96.49	96.49	
4	3. NT Cells+N	Singlets	54807	93.38	96.77	
5	3. NT Cells+N	Viability	53487	91.13	97.59	
6	3. NT Cells+N	Cy5+	3134	5.34	5.86	
7	3. NT Cells+N	Cy5-	50296	85.69	94.03	
8						
9						
10						

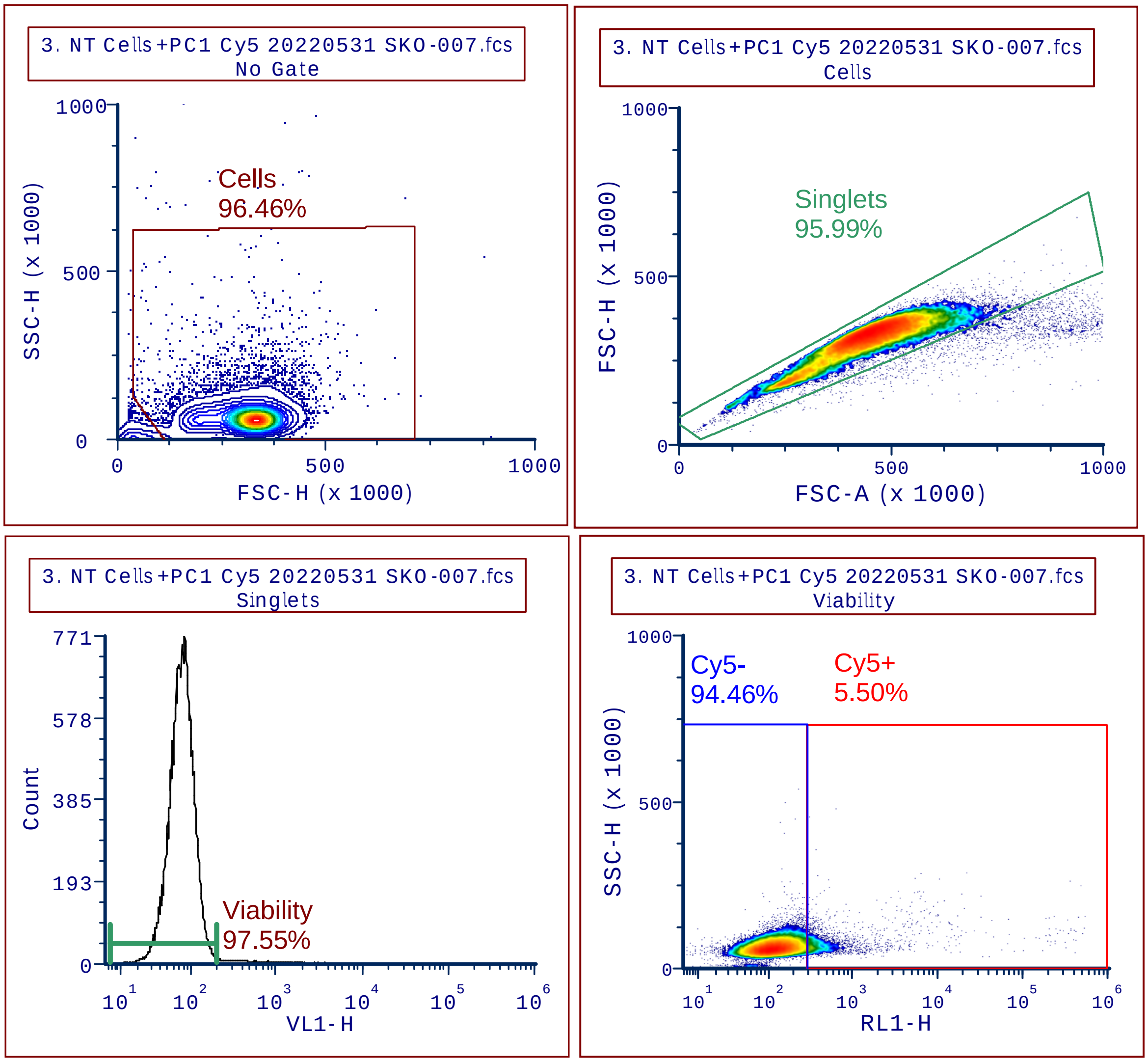

	A	B	C	D	E	F
1	**Filename**	**Gate**	**# of Events**	**% of all cells**	**% of gated cells**	
2	3. NT Cells+P	All Events	55358	100	100	
3	3. NT Cells+P	Cells	53396	96.46	96.46	
4	3. NT Cells+P	Singlets	51257	92.59	95.99	
5	3. NT Cells+P	Viability	50000	90.32	97.55	
6	3. NT Cells+P	Cy5+	2751	4.97	5.5	
7	3. NT Cells+P	Cy5-	47230	85.32	94.46	
8						
9						
10						

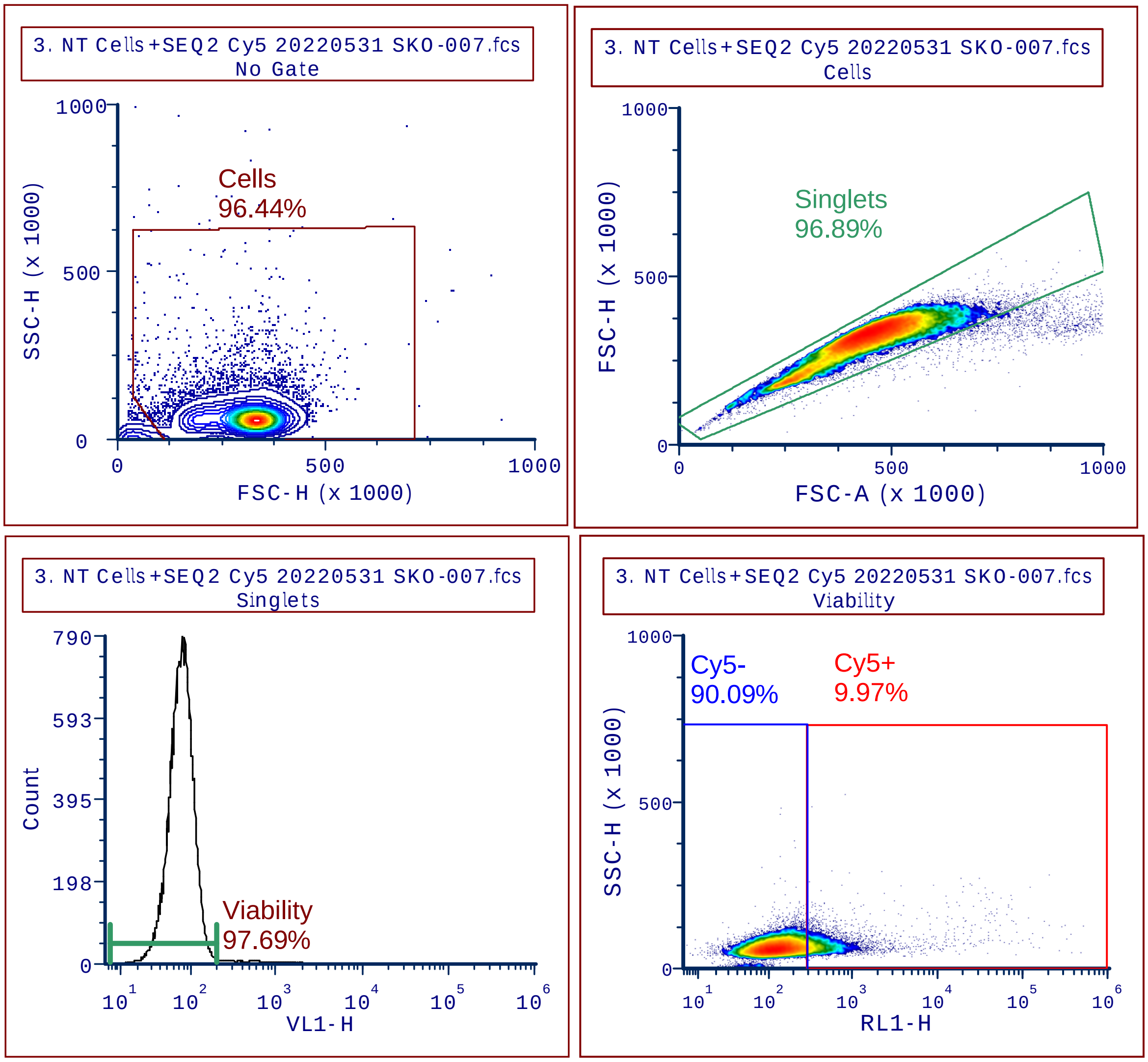

	A	B	C	D	E	F
1	**Filename**	**Gate**	**# of Events**	**% of all cells**	**% of gated cells**	
2	3. NT Cells+SI	All Events	56416	100	100	
3	3. NT Cells+SI	Cells	54410	96.44	96.44	
4	3. NT Cells+SI	Singlets	52720	93.45	96.89	
5	3. NT Cells+SI	Viability	51503	91.29	97.69	
6	3. NT Cells+SI	Cy5+	5133	9.1	9.97	
7	3. NT Cells+SI	Cy5-	46398	82.24	90.09	
8						
9						
10						

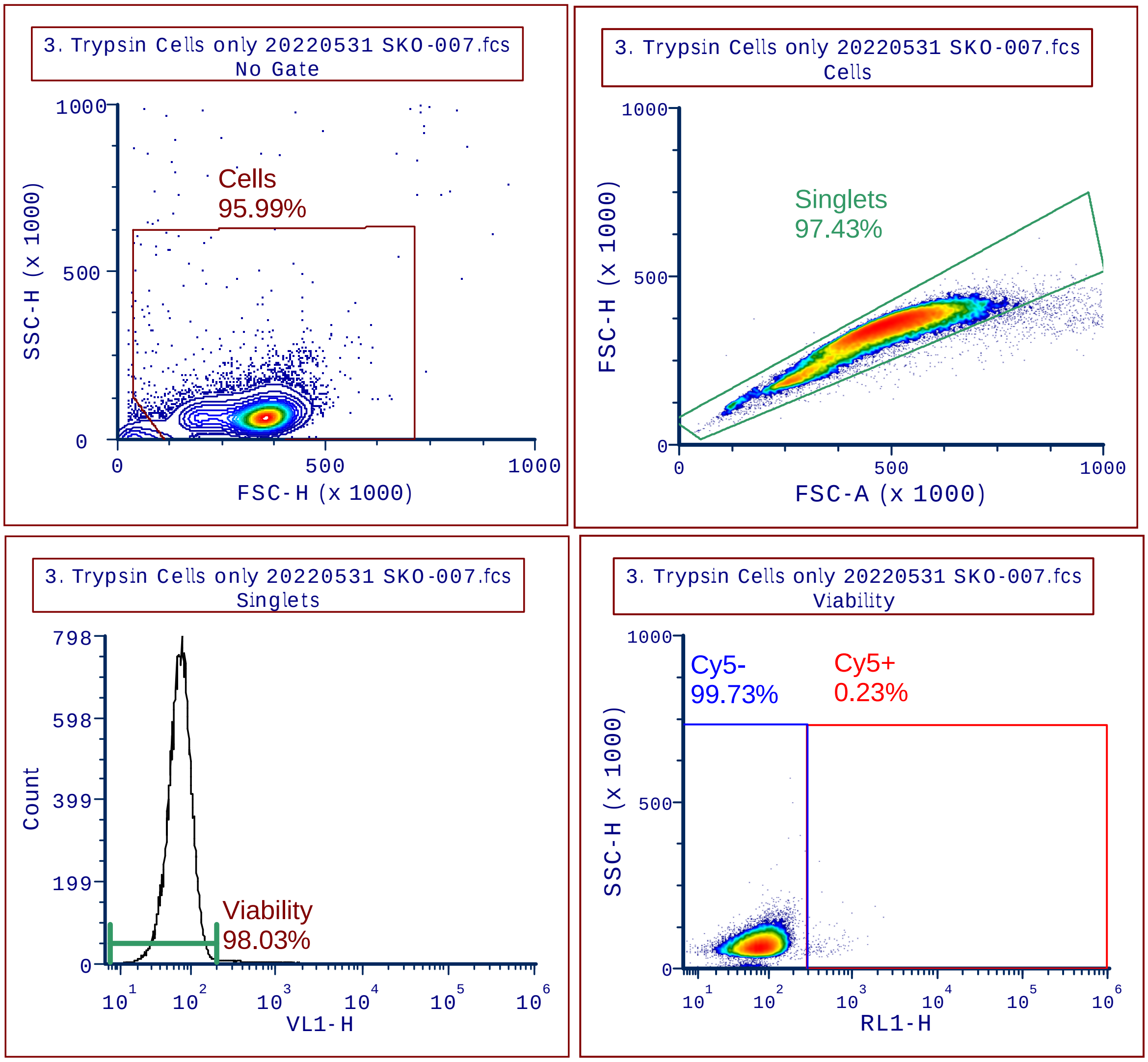

	A	B	C	D	E	F
1	**Filename**	**Gate**	**# of Events**	**% of all cells**	**% of gated cells**	
2	3. Trypsin Cel	All Events	54201	100	100	
3	3. Trypsin Cel	Cells	52025	95.99	95.99	
4	3. Trypsin Cel	Singlets	50690	93.52	97.43	
5	3. Trypsin Cel	Viability	49692	91.68	98.03	
6	3. Trypsin Cel	Cy5+	115	0.21	0.23	
7	3. Trypsin Cel	Cy5-	49559	91.44	99.73	
8						
9						
10						

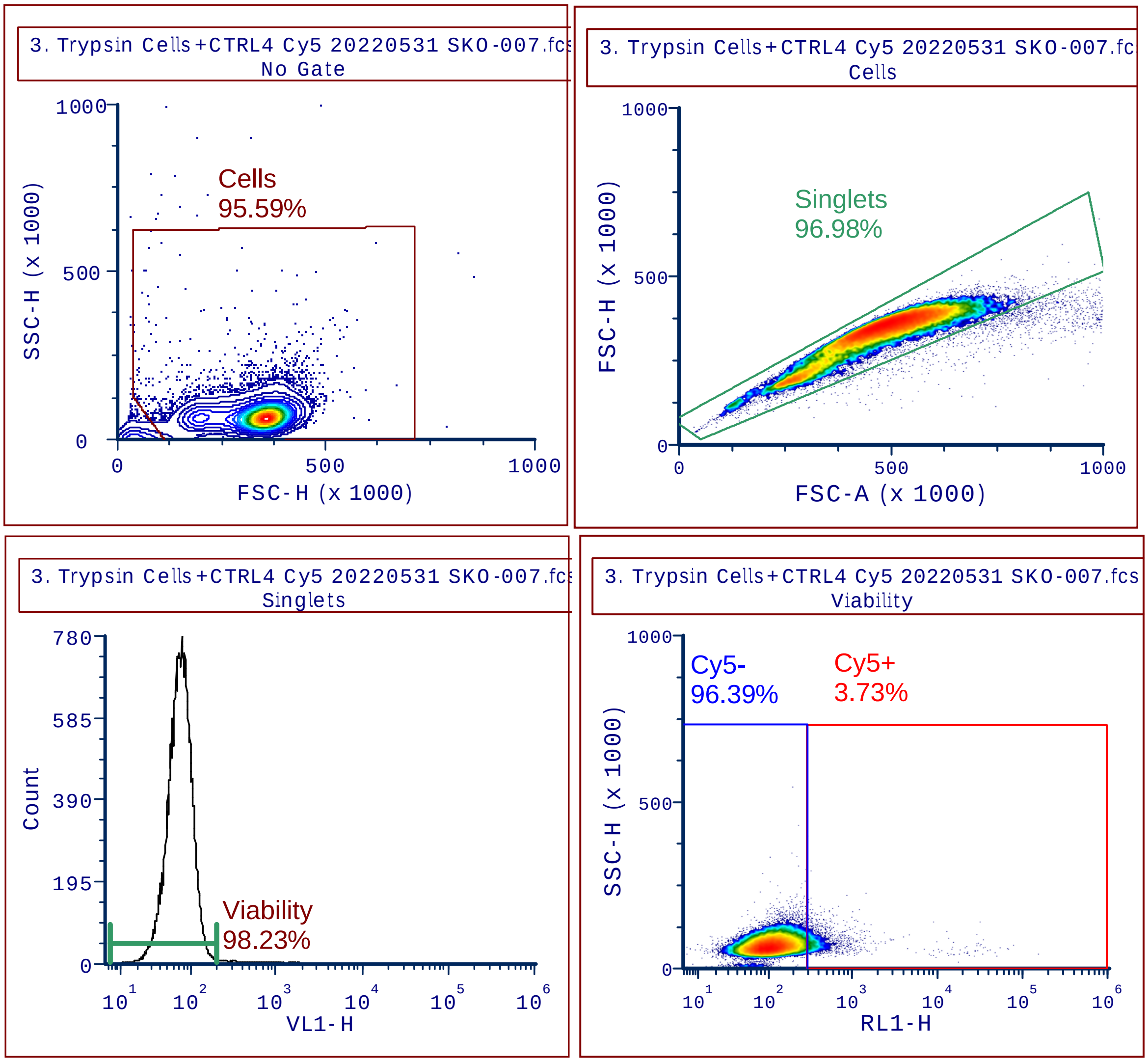

	A	B	C	D	E	F
1	**Filename**	**Gate**	**# of Events**	**% of all cells**	**% of gated cells**	
2	3. Trypsin Cel	All Events	54684	100	100	
3	3. Trypsin Cel	Cells	52270	95.59	95.59	
4	3. Trypsin Cel	Singlets	50690	92.7	96.98	
5	3. Trypsin Cel	Viability	49795	91.06	98.23	
6	3. Trypsin Cel	Cy5+	1855	3.39	3.73	
7	3. Trypsin Cel	Cy5-	47995	87.77	96.39	
8						
9						
10						

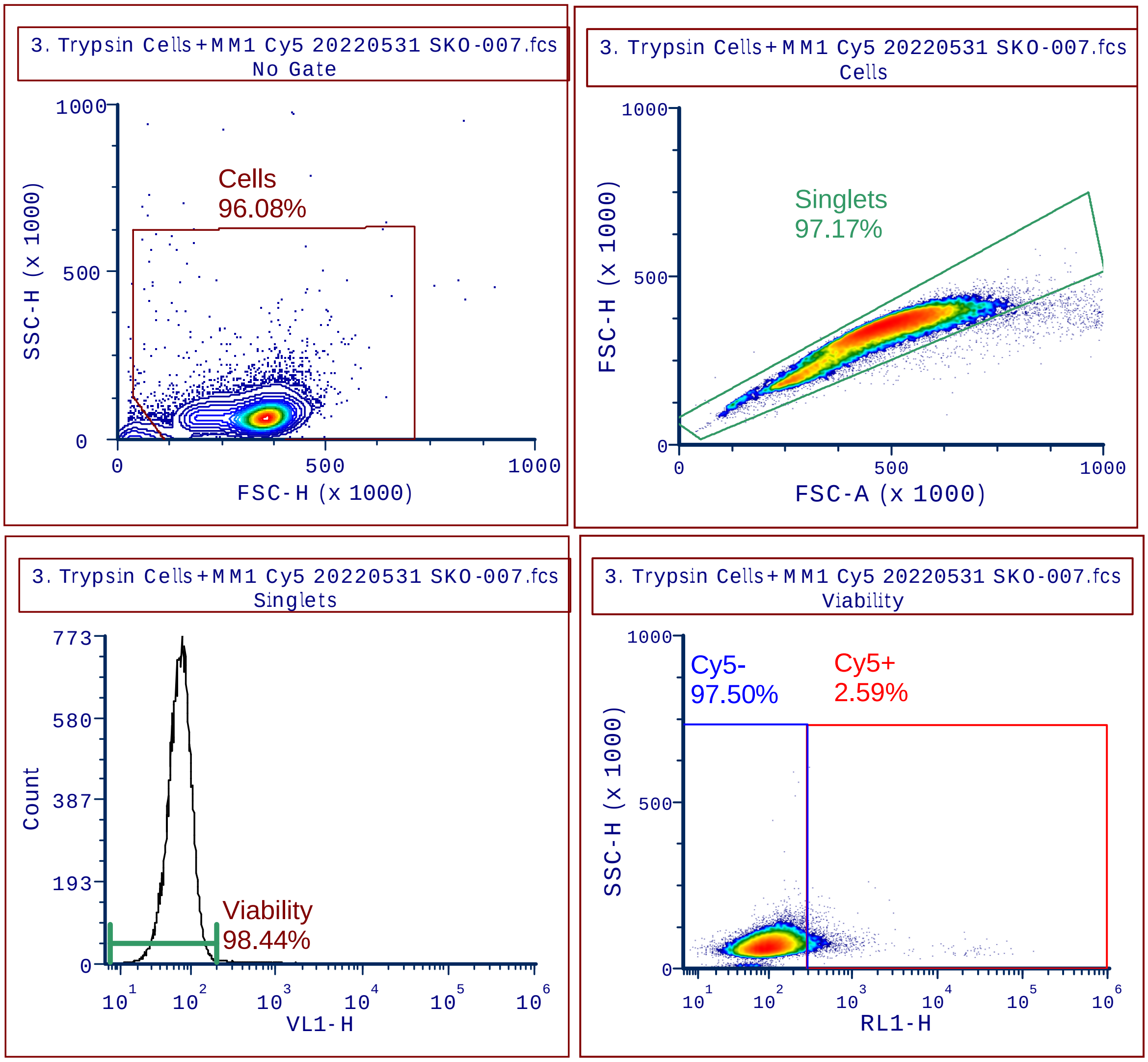

	A	B	C	D	E	F
1	**Filename**	**Gate**	**# of Events**	**% of all cells**	**% of gated cells**	
2	3. Trypsin Cel	All Events	53059	100	100	
3	3. Trypsin Cel	Cells	50979	96.08	96.08	
4	3. Trypsin Cel	Singlets	49538	93.36	97.17	
5	3. Trypsin Cel	Viability	48766	91.91	98.44	
6	3. Trypsin Cel	Cy5+	1265	2.38	2.59	
7	3. Trypsin Cel	Cy5-	47548	89.61	97.5	
8						
9						
10						

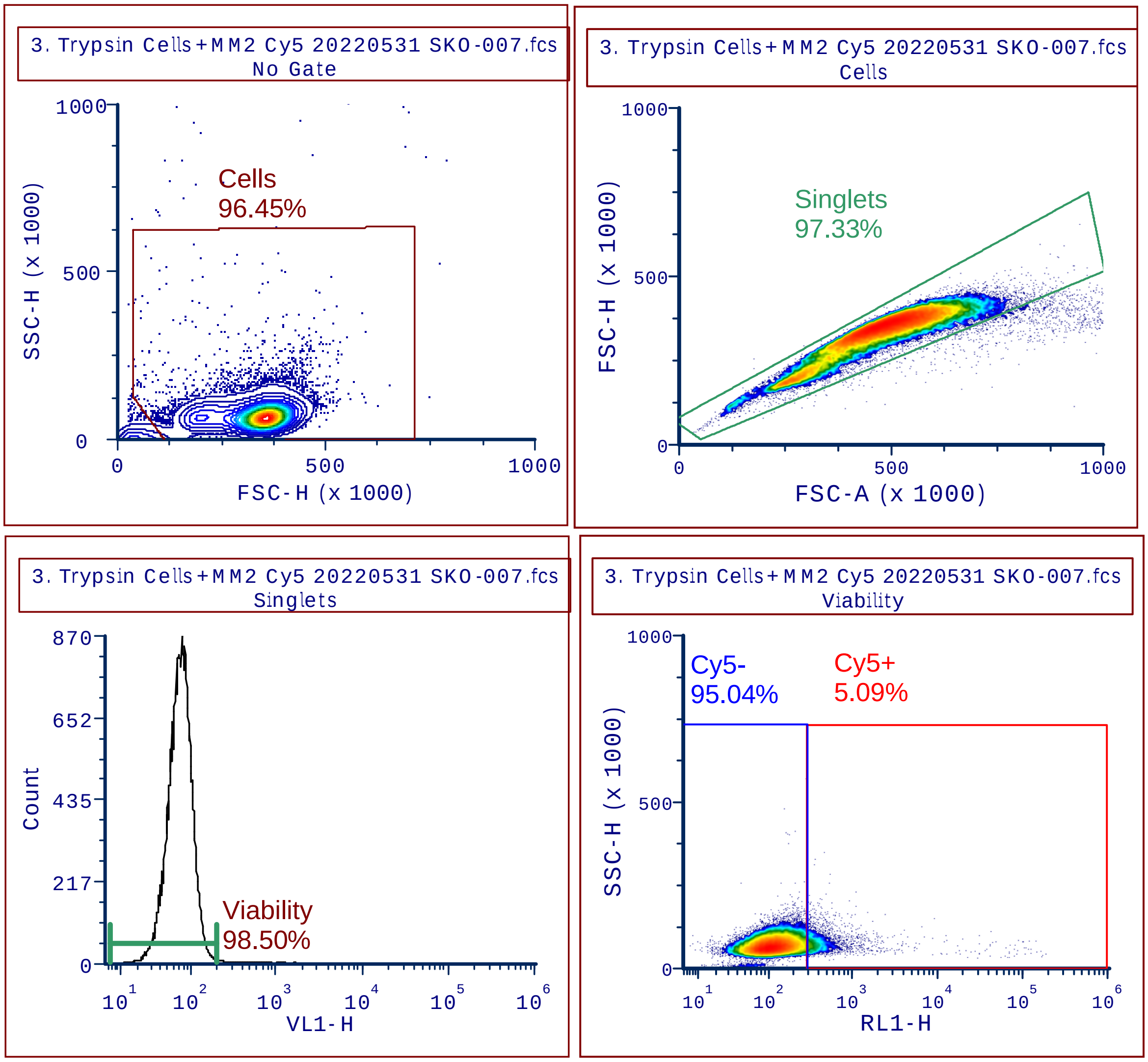

	A	B	C	D	E	F
1	**Filename**	**Gate**	**# of Events**	**% of all cells**	**% of gated cells**	
2	3. Trypsin Cel	All Events	59597	100	100	
3	3. Trypsin Cel	Cells	57482	96.45	96.45	
4	3. Trypsin Cel	Singlets	55948	93.88	97.33	
5	3. Trypsin Cel	Viability	55108	92.47	98.5	
6	3. Trypsin Cel	Cy5+	2806	4.71	5.09	
7	3. Trypsin Cel	Cy5-	52372	87.88	95.04	
8						
9						
10						

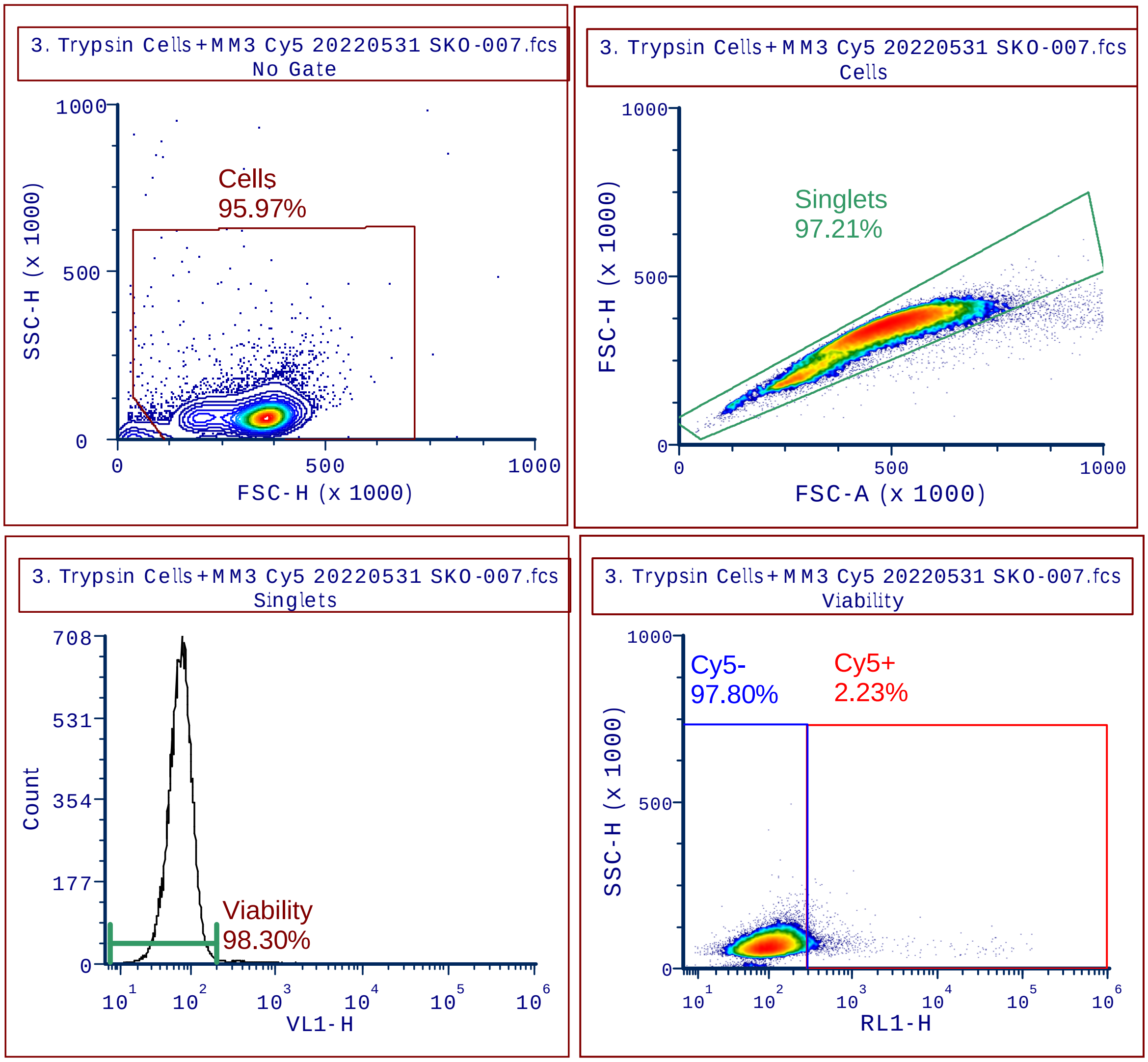

	A	B	C	D	E	F
1	**Filename**	**Gate**	**# of Events**	**% of all cells**	**% of gated cells**	
2	3. Trypsin Cel	All Events	48593	100	100	
3	3. Trypsin Cel	Cells	46634	95.97	95.97	
4	3. Trypsin Cel	Singlets	45334	93.29	97.21	
5	3. Trypsin Cel	Viability	44565	91.71	98.3	
6	3. Trypsin Cel	Cy5+	993	2.04	2.23	
7	3. Trypsin Cel	Cy5-	43585	89.69	97.8	
8						
9						
10						

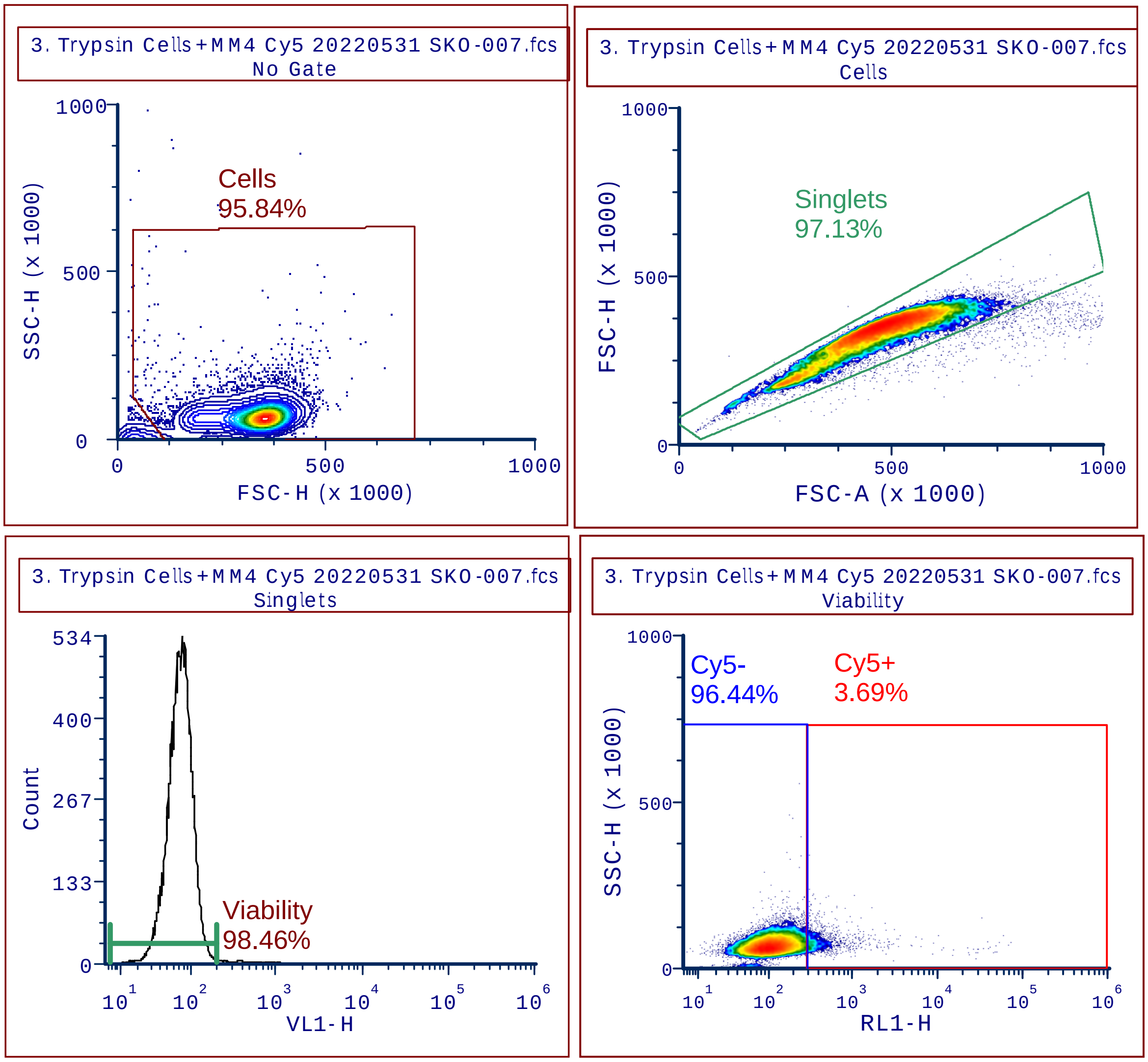

	A	B	C	D	E	F
1	**Filename**	**Gate**	**# of Events**	**% of all cells**	**% of gated cells**	
2	3. Trypsin Cel	All Events	37656	100	100	
3	3. Trypsin Cel	Cells	36090	95.84	95.84	
4	3. Trypsin Cel	Singlets	35054	93.09	97.13	
5	3. Trypsin Cel	Viability	34513	91.65	98.46	
6	3. Trypsin Cel	Cy5+	1272	3.38	3.69	
7	3. Trypsin Cel	Cy5-	33285	88.39	96.44	
8						
9						
10						

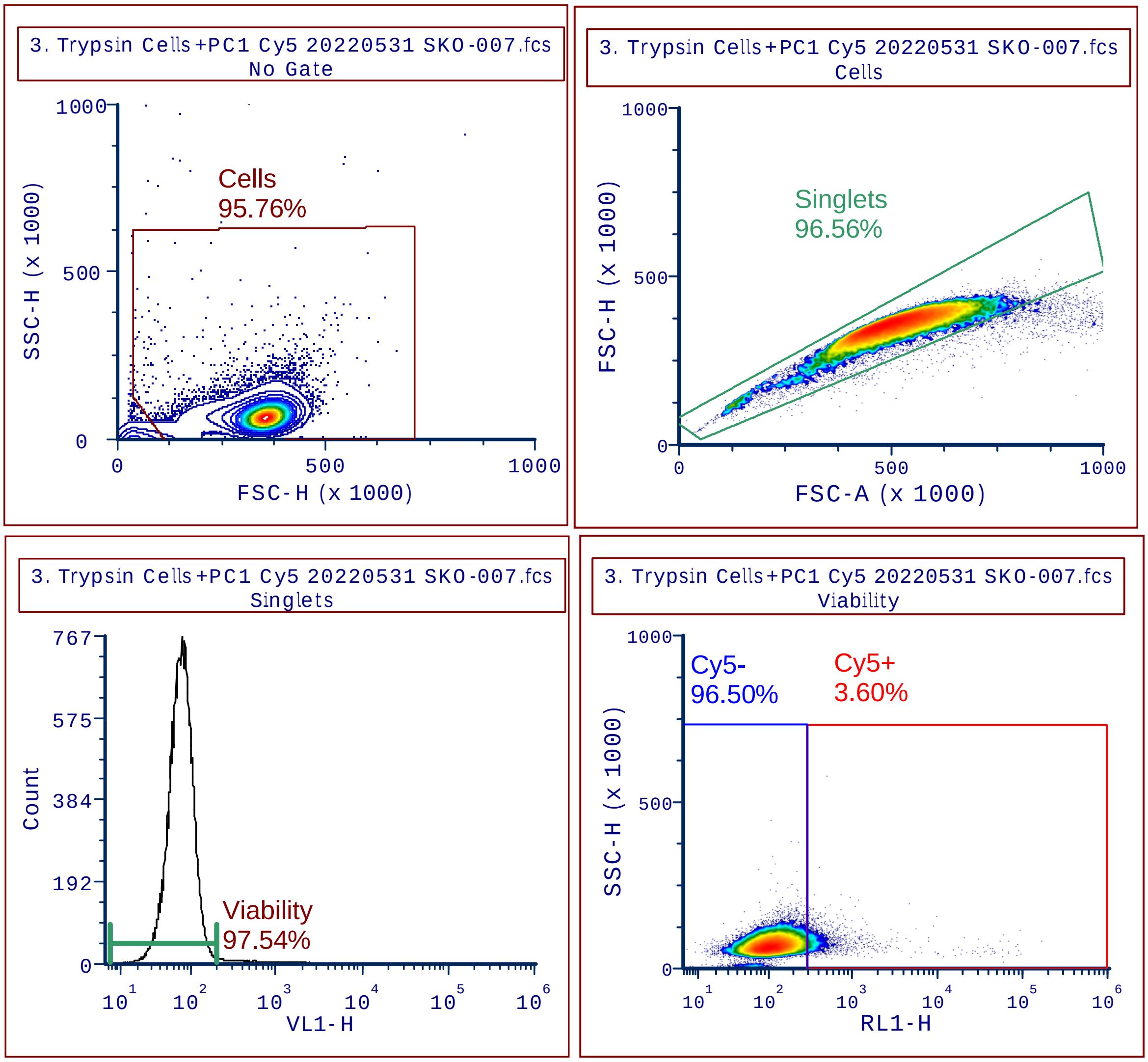

	A	B	C	D	E	F
1	**Filename**	**Gate**	**# of Events**	**% of all cells**	**% of gated cells**	
2	3. Trypsin Cel	All Events	55268	100	100	
3	3. Trypsin Cel	Cells	52926	95.76	95.76	
4	3. Trypsin Cel	Singlets	51104	92.47	96.56	
5	3. Trypsin Cel	Viability	49848	90.19	97.54	
6	3. Trypsin Cel	Cy5+	1797	3.25	3.6	
7	3. Trypsin Cel	Cy5-	48105	87.04	96.5	
8						
9						
10						

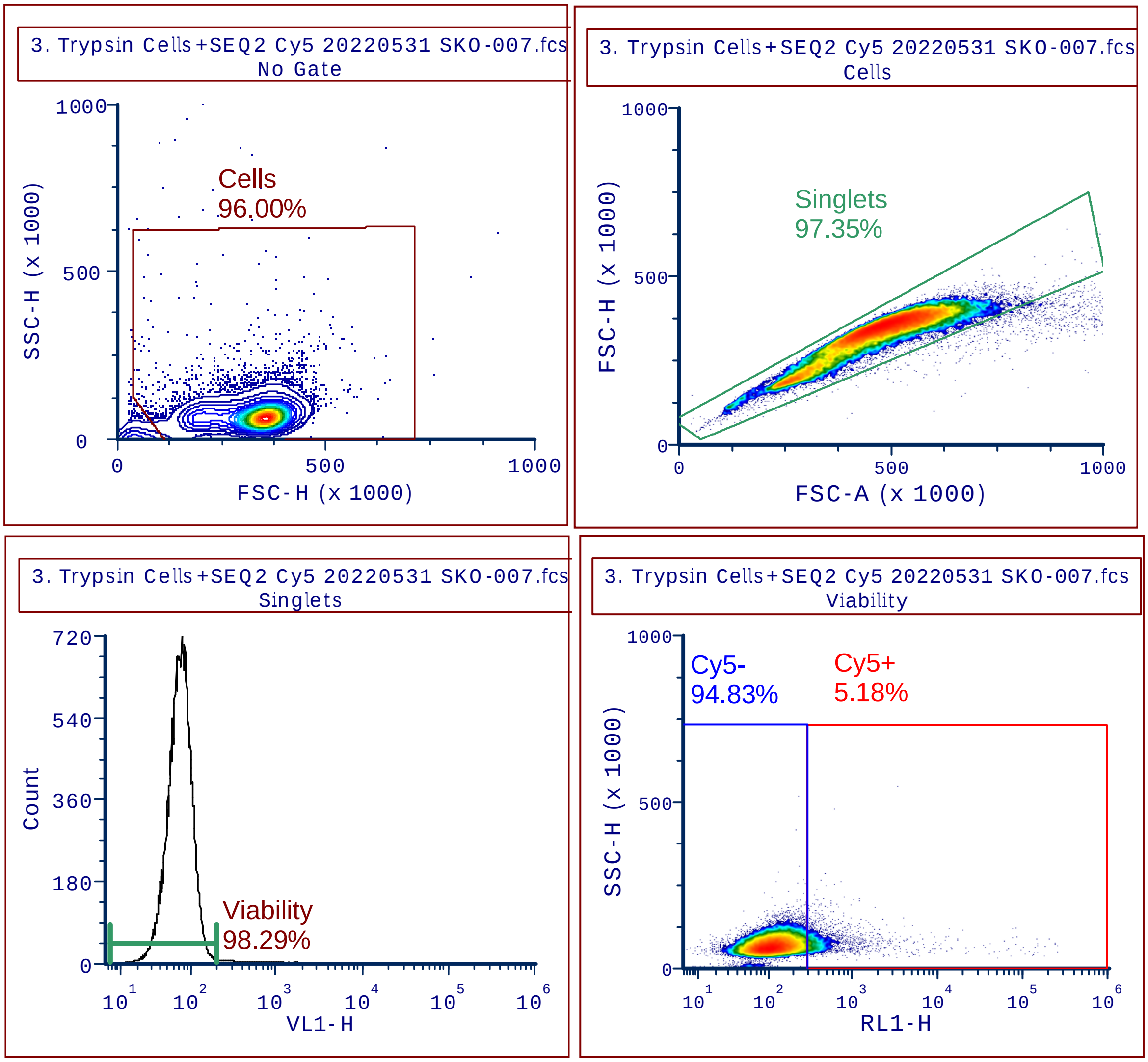

	A	B	C	D	E	F
1	**Filename**	**Gate**	**# of Events**	**% of all cells**	**% of gated cells**	
2	3. Trypsin Cel	All Events	49868	100	100	
3	3. Trypsin Cel	Cells	47873	96	96	
4	3. Trypsin Cel	Singlets	46605	93.46	97.35	
5	3. Trypsin Cel	Viability	45808	91.86	98.29	
6	3. Trypsin Cel	Cy5+	2374	4.76	5.18	
7	3. Trypsin Cel	Cy5-	43438	87.11	94.83	
8						
9						
10						
